# Mendelian randomization analysis demonstrates the causal effects of IGF family members in diabetes

**DOI:** 10.3389/fmed.2024.1332162

**Published:** 2024-02-05

**Authors:** Xing Li, Jie Tang, Sha Lin, Xuwei Liu, Yifei Li

**Affiliations:** Key Laboratory of Birth Defects and Related Diseases of Women and Children of MOE, Department of Pediatrics, West China Second University Hospital, Sichuan University, Chengdu, China

**Keywords:** IGF family, diabetes, GWAS, development, mendelian randomization study

## Abstract

**Background:**

Observational studies have consistently shown significant associations between the IGF family and metabolic diseases, including diabetes. However, these associations can be influenced by confounding factors and reverse causation. This study aimed to assess the causal relationship between the IGF family and diabetes using Mendelian randomization (MR).

**Methods:**

We conducted a two-sample MR analysis to investigate the causal effects of the IGF family on diabetes. Instrumental variables for the IGF family and diabetes were derived from summary-level statistics obtained from genome-wide association studies. Horizontal pleiotropy was assessed using MR-Egger regression and the weighted median method. We applied the inverse-variance weighted method as part of the conventional MR analysis to evaluate the causal impact of the IGF family on diabetes risk. To test the robustness of the results, we also employed MR-Egger regression, the weighted median method, and a leave-one-out analysis.

**Results:**

Our study revealed that IGF-1 causally increases the risk of Type 2 Diabetes (T2D), while IGFBP-6, adiponectin and INSR decreases the risk (IGF-1, OR 1.02 [95% CI 1–1.03], *p* = 0.01; IGFBP-6, OR 0.92 [95% CI 0.87–0.98], *p* = 0.01; Adiponectin, OR 0.837 [95% CI 0.721–0.970], *p* = 0.018; INSR, OR 0.910 [95% CI 0.872–0.950], *p* = 1.52 × 10–5). Additionally, genetically lower levels of IGF-1 and IGFBP-5, along with higher levels of IGFBP-7, were associated with an increased risk of Type 1 Diabetes (T1D) (IGF-1, OR 0.981 [95% CI 0.963–0.999], *p* = 0.037; IGFBP-5, OR 0.882 [95% CI 0.778–0.999], *p* = 0.049; IGFBP-7, OR 1.103 [95% CI 1.008–1.206], *p* = 0.033).

**Conclusion:**

In summary, our investigation has unveiled causal relationships between specific IGF family members and T1D and T2D through MR analysis. Generally, the IGF family appears to reduce the risk of T1D, but it presents a more complex and controversial role in the context of T2D. These findings provide compelling evidence that T2D is intricately linked with developmental impairment. Our study results offer fresh insights into the pathogenesis and the significance of serum IGF family member concentrations in assessing diabetes risk.

## Introduction

Diabetes is one of the most common endocrine diseases (1). According to the different causes, diabetes is divided into the several types, including type I diabetes mellitus (T1DM), type 2 diabetes mellitus (T2DM), gestational diabetes mellitus (GDM), and maturity onset diabetes of the young (MODy) (1, 2). Diabetes management is a complex and ongoing process that involves addressing a range of challenges and difficulties associated with this metabolic disorder. he foundation for the risk of diabetes can be established during fetal development. Poor maternal nutrition, maternal obesity, gestational diabetes, and other factors during pregnancy can influence the baby’s future risk of diabetes. Our previous researches demonstrated the early adverse environmental exposure would lead to programmed metabolic dysfunction. Besides, infants and young children exposed to an obesogenic environment or excessive calorie intake may be more prone to developing obesity and insulin resistance, which are risk factors for diabetes. Importantly, Low birth weight, often associated with poor maternal nutrition, has been linked to an increased risk of T2D later in life. Some individuals who experience low birth weight may undergo rapid catch-up growth in early childhood, which can exacerbate this risk. According to our clinical investigation that the insulin-like growth factor (IGF) family was widely involved in normal children development and catch-up group. So that, it is important to address the cause effects of IGF family members in the origins of diabetes.

Beyond genetic background, the developmental influences had been identified in kinds of endocrine and metabolic disorders. The IGF family serves as an important role in regulating cell growth and differentiation, proliferation, and survival, including IGF binding proteins (IGFBPs), IGF ligands, IGF receptors, and IGF modulators (3). Previous researches demonstrated that IGF family widely participated in regulation of metabolic function, such as glucose transportation and glycolysis. And IGF family members had been involved in immune regulation and can serve as a potential target for T1D. Studies have shown that IGFs may prevent existing damages in the pancreatic tissue and inhibiting the activated immune responses in T1D, revealing a promising role in T1D management (4). Recent research has provided insights into the roles of IGF-1 and IGF-2 in glucose metabolism and insulin sensitivity. Altered IGF signaling pathways have been implicated in insulin resistance and β-cell dysfunction, key features of T2D. Additionally, changes in IGF binding proteins and their interactions with IGFs have been associated with the risk of developing diabetes. Emerging evidence also suggests a potential role of the IGF family in the complications of diabetes, such as diabetic nephropathy and retinopathy. Modulation of the IGF axis presents a promising avenue for future therapeutic strategies. A review has shown that the high IGF-1 concentrations can prevent or delay the inception of diabetes-related complications in people with diabetes (5). According to a targeted serum proteomics study, lower levels of IGF1 and IGFBP3 and elevated IGFBP1 level were detected in the sera of T1D youth (6). There were additionally studies confirmed that adiponectin and INSR were crucial in regulating glucose metabolism and insulin sensitivity (7, 8). Moreover, the diabetes-related histological and functional changes, especially fibro-genesis, could be attenuated by IGF-1/IGF-1R inhibitors (9). The diabetes-related histological and functional changes, as well as fibrogenesis, can be attenuated by IGF-1/IGF-1R inhibition (9). To date, A small prospective observational study indicated that low IGF-1 levels were associated with the increased risk of T2D. At the same time, this study suggested that IGFBP-1 levels might alter the regulation of IGF-1 in glucose tolerance (3, 10).

However, observational studies are susceptible to contingencies, inverse causality, and residual or unmeasured confounding. Therefore, it is incapable of confirming whether these associations are causal. Mendelian randomization (MR) is a method that uses genetic variation data to unbiased test or estimate causal relationships between exposure and associated outcomes. As the gold standard for causal inference in epidemiological studies, randomized controlled trials are sometimes difficult to conduct because of ethical limitations and high costs (11). Mendelian randomization (MR) is a method that uses genetic variation data to unbiased test or estimate causal relationships between exposure and associated outcomes. The selected SNPs are called instrumental variances (IVs) (12). This approach is less prone to reverse causality and confusion, and the comparison of genetically defined groups of individuals is equivalent to a random comparison (13). Herein, we used MR strategy to underline the association between IGF family members’ levels and the onsets of diabetes. In this study, two-sample MR with a large sample size had been carried out to determine whether there was a causal relationship between IGF and diabetes.

## Methods

### Study design

This study was design to assess the causal effects of IGF family members in the risk of T2DM/T1DM. The related traits of IGF family members had been identified, and 14 IGF family members traits included: IGF1, IGF1-sR, IGF-IIR, IGFBP1, IGFBP2, IGFBP3, IGFBP4, IGFBP5, IGFBP6, IGFBP7, IGF-LR1, CTGF, WISP1, and CYR61. As the crucial correlation between IGF1 and cytokine including adiponectin and INSR, we also put them into analysis to estimate the genetic effect in pathology of diabetes. Besides, three traits had been retrieved for genetic association of T1D and T2D, including finn-b-E4_DM1NOCOMP for T1D, and finn-b-T2D for T2D. First, the effects of 14 IGF family members and their serum concentration were evaluated to identify the potential Single nucleotide polymorphisms (SNPs) as one sample MR analysis. Then two-sample MR analysis had been completed among diabetes traits to measure the causal effects of IGF family members in T1D and T2D origins.

### GWAS summary data of diabetes and IGF family

We acquired the GWAS summary data for diabetes from a comprehensive combination of sources, including the FinnGen Biobank, and the UK Biobank resource (14). This dataset encompassed a total of 4,918 T1D cases without complications and 183,185 control participants. While the dataset encompassed a total of 29,193 general T2D cases and 183,573 control participants. The identification of diabetes events within the summary dataset was based on diagnostic codes, self-reports, operation codes, or causes of death. Additionally, we utilized GWAS summary datasets from the FinnGen Biobank and the UK Biobank as duplications.

To identify SNPs associated with IGF family members, adiponectin and INSR, we extracted and selected data from the latest and largest genome-wide association studies (GWAS) available in the UK Biobank resource, the KORA cohorts (15), and the INTERVAL study (16). These genetic associations were adjusted for age, sex, and body mass index. All the GWAS datasets we selected are presented in Table 1.

**Table 1:** All GWAS datasets selected in this article.

ID	Trait	Year	No. of Variants
ukb-d-30770_raw	IGF-1	2018	13,586,000
prot-c-2771_35_2	IGFBP-1	2019	501,428
prot-c-2570_72_5	IGFBP-2	2019	501,428
prot-c-2571_12_3	IGFBP-3	2019	501,428
prot-c-2950_57_2	IGFBP-4	2019	501,428
prot-c-2685_21_2	IGFBP-5	2019	501,428
prot-c-2686_67_2	IGFBP-6	2019	501,428
prot-c-3320_49_2	IGFBP-7	2019	501,428
prot-a-1455	IGFLR1	2018	10,534,735
prot-c-4232_19_2	IGF-I Sr	2019	501,428
prot-c-3676_15_3	IGF-IIR	2019	501,428
prot-c-2975_19_2	CTGF	2019	501,428
prot-c-3057_55_1	WISP-1	2019	501,428
prot-a-758	CYR61	2018	10,534,735
ieu-a-1	Adiponectin	2012	2,675,209
prot-a-1564	ISNR	2018	10,534,735
finn-b-T2D	T2DM	2021	16,380,433
finn-b-E4_DM1NOCOMP	T1DM	2021	16,379,879

### Genetic correlation analysis

We utilized LDSC (v1.0.1^1^) software to assess the genetic correlations between diabetes and each member of the IGF family, adiponectin and INSR. LDSC is a robust approach for conducting genetic correlation analyses of complex diseases or traits. It allows for the discrimination between true polygenetic effects and potential mixed biases, encompassing implicit associations and demographic stratification. When a genetic association demonstrates both statistical and quantitative significance, it provides confirmation that the overall phenotypic association is not solely attributable to environmental confounding factors. In this study, we examined the linkage disequilibrium (LD) between diabetes and each IGF family member, adiponectin and INSR, employing the European 1,000 G reference panel as the reference dataset. To establish statistical significance, we applied a stringent Bonferroni correction, setting the significant association threshold at *p* > 0.00357 (0.05/14). *p*-values falling within the range of 0.00357–0.05 were considered suggestive of significance (17).

### Mendelian randomization analysis

In the present study, we employed MR analysis to assess the potential causal relationship between each member of the IGF family and diabetes. We conducted the analysis using the inverse variance weighted (IVW) method and initially identified significant IGF family members through LDSC analysis, which were subsequently included in further analyses. For each IGF family member, we selected SNPs strongly predictive of exposure at the genome-wide significance level (*p* < 5 × 10^−8^). To minimize potential pleiotropy, we excluded SNPs associated with multiple cytokines. Additionally, we retained SNPs with low linkage disequilibrium (*r*^2^ < 0.1) to avoid the confounding effects of correlated SNPs. However, it should be noted that despite these efforts, none of the SNPs associated with IGF family members showed significant associations with diabetes in the harmonized GWAS datasets. Consequently, we adopted a more stringent cutoff (*p* < 1 × 10^−5^) to select SNPs predicting IGF family members. We reported the number of included SNPs, along with effect estimates, confidence intervals, and *p*-values (18).

MR estimates were derived using the inverse-variance weighted (IVW) method and the MR-Egger method, both implemented under a random-effects model. To assess the robustness of our IVW results, we conducted tests for heterogeneity, multiple validity tests, and sensitivity analyses using weighted median estimation and MR–Egger regression. The TwoSampleMR packages (18) (version 0.5.6) in R (version 4.0.4) were utilized for performing the MR analysis. The statistical significance level was set at *p* < 0.05. We used the IVW method and MR-Egger regression to detect heterogeneity. The heterogeneities were quantified by Cochran Q statistic; a *p* value of < 0.05 would be regarded as significant heterogeneity. Additionally, to identify potentially influential SNPs, we performed a “leave-one-out” sensitivity analysis to where the MR is performed again but leaving out each SNP in turn.

## Results

### Causal effects of serum IGF family on the risk of T1D

In accordance with our study design strategy, we investigated the potential causal effects of serum IGF family members’ concentrations on the risk of T1D. We included 14 molecules in the initial one-sample MR analysis to identify SNPs that might influence their serum concentrations. These molecules were IGF1 (prot-c-2952_75_2), IGF1-sR (prot-c-4232_19_2), IGF-IIR (prot-c-3676_15_3), IGFBP1 (prot-c-2771_35_2), IGFBP2 (prot-c-2570_72_5), IGFBP3 (prot-c-2571_12_3), IGFBP4 (prot-c-2950_57_2), IGFBP5 (prot-c-2685_21_2), IGFBP6 (prot-c-2686_67_2), IGFBP7 (prot-c-3320_49_2), IGF-LR1 (prot-a-1455), CTGF (prot-c-2975_19_2), WISP1 (prot-c-3057_55_1), and CYR61 (prot-a-758).

Out of these 14 serum concentration traits related to IGF family members, which had previously been substantiated in published studies, 13 of them displayed more than one genome-wide significant SNP site. Further details, including the outcomes of the clumping process for LD-independent SNPs related to the exposure, are provided in the Supplementary figures. Notably, all calculated F-statistics exceeded a value of ten, indicating that the results were less susceptible to the bias associated with weak instruments.

**Figure 1:**
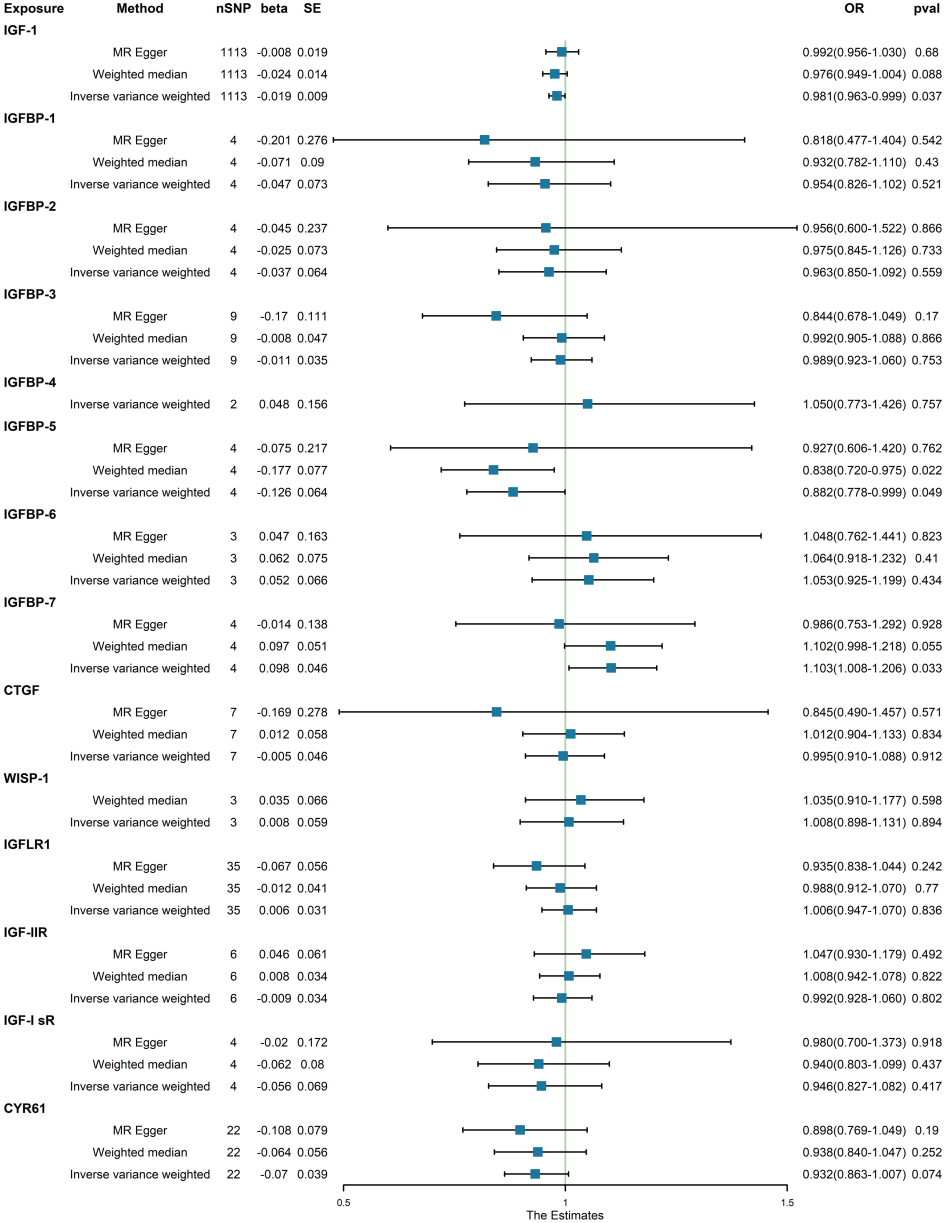
Associations between genetically cause of IGFs levels and T1D.

**Figure 2:**
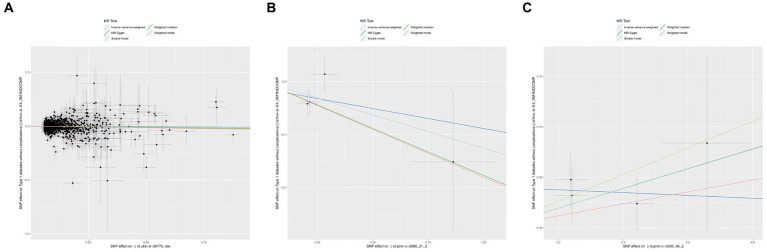
Scatter plots for MR analyses of the causal effect of IGFs on T1D. **(A)** IGF-1. **(B)** IGFBP-5. **(C)** IGFBP-7. Analyses were conducted using the conventional IVW, MBE, WMM, MR-Egger, and MR.RAPS methods. The slope of each line corresponding to the estimated MR effect per method.

In the initial one-step MR analysis, we employed both the MR-Egger and IVW methods. Subsequently, we identified multiple SNPs that reached genome-wide significance (*p* < 1 × 10–5) among the 14 IGF family molecules, which were employed to assess their causal effects on T1D. Upon pooling the data, three IGF family molecules were found to be associated with T1D. Specifically, the level of circulating IGF1 was associated with a reduced risk of T1D onset (OR = 0.981, 95%CI = 0.963–0.990, *p* = 0.037, IVW method). Notably, IGFBP-5 also exhibited a negative correlation with T1D prevalence, as evidenced in both the WM analysis (OR = 0.838, 95%CI = 0.720–0.975, *p* = 0.022) and IVW analysis (OR = 0.882, 95%CI = 0.778–0.999, *p* = 0.049). In contrast, a higher serum concentration of IGFBP-7 was positively correlated with the pathogenesis of T1D, as indicated by the IVW approach (OR = 1.103, 95%CI = 1.008–1.206, *p* = 0.033). However, the remaining molecules did not provide compelling evidence for positive effects on assessing the causal influence of IGF family members on the risk of T1D (Figure 1). Scatter plots depicting the MR analyses of the causal effects of IGFs on T1D with statistical significance are presented in Figure 2 (A for IGF-1, B for IGFBP-5, and C for IGFBP-7, respectively). All the involved funnel plots, scatter plots and “leave-one out analysis” plots in assessing the association between IGFs family and T1D were shown in Supplementary file 2.

### Causal effects of serum IGF family on the risk of T2D

**Figure 3:**
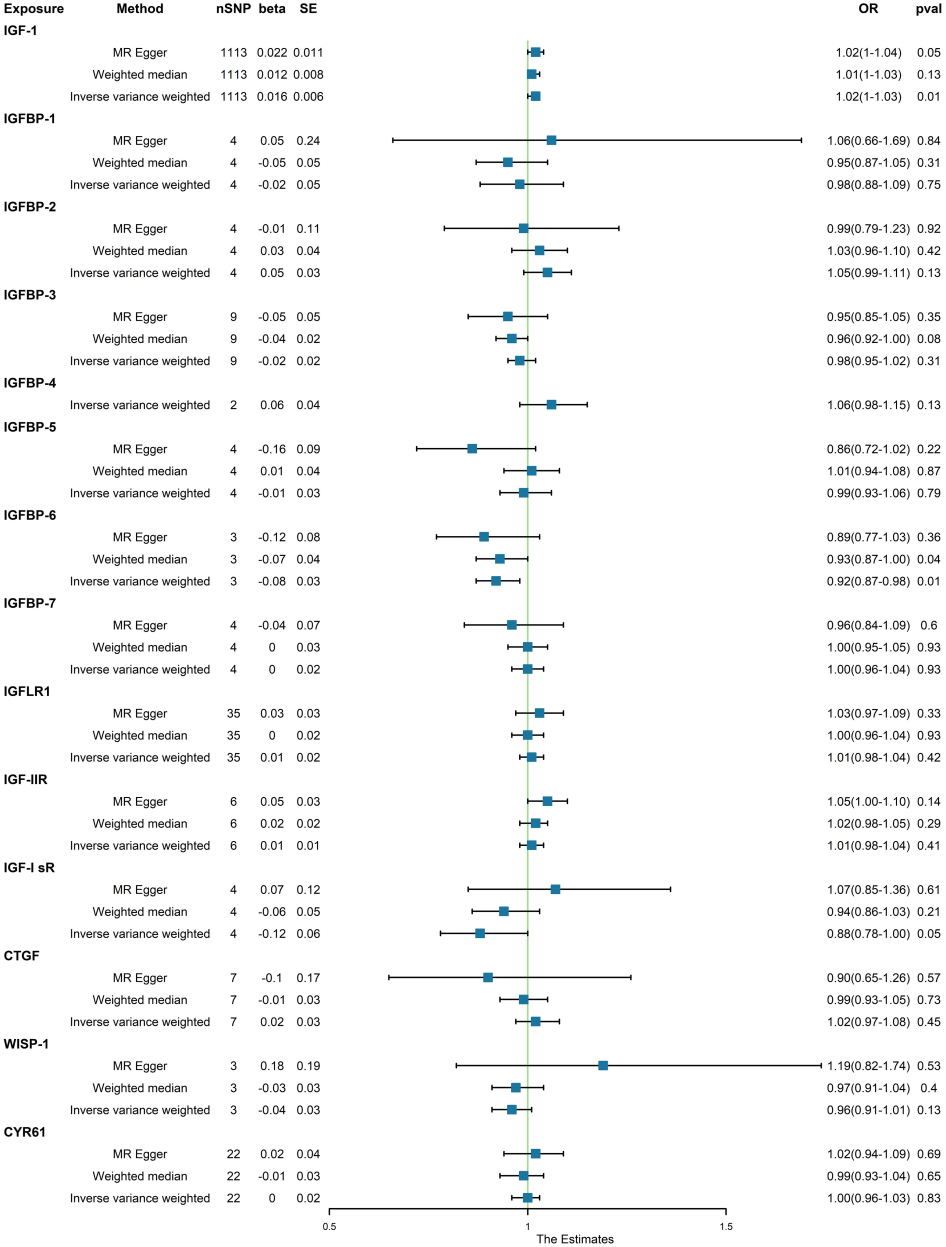
Associations between genetically cause of IGFs levels and T2D.

**Figure 4:**
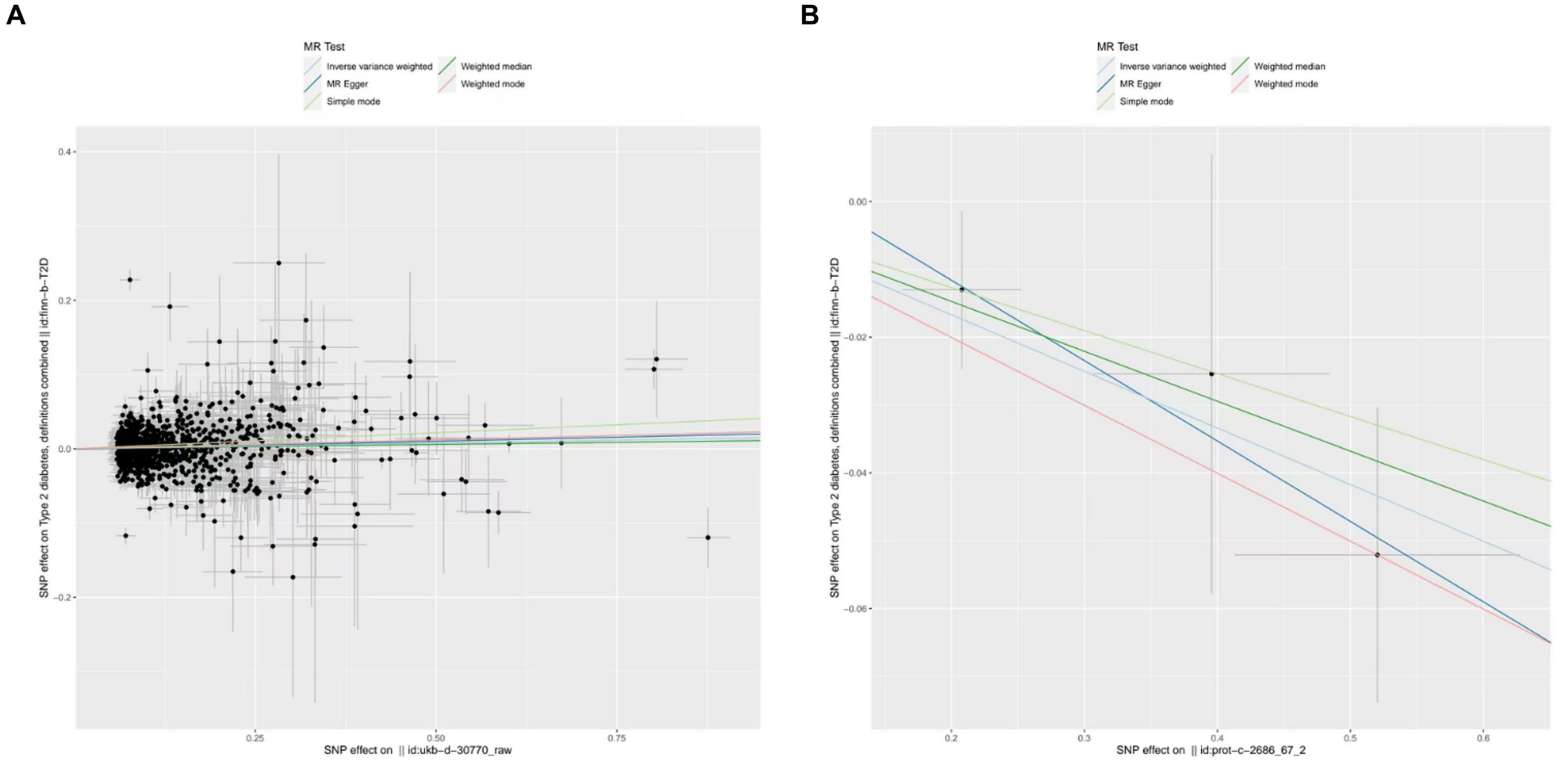
Scatter plots for MR analyses of the causal effect of IGFs on T2D. **(A)** IGF-1. **(B)** IGFBP-6. Analyses were conducted using the conventional IVW, MBE, WMM, MR-Egger, and MR.RAPS methods. The slope of each line corresponding to the estimated MR effect per method.

Upon identifying the 14 serum concentration traits associated with IGF family members and retrieving specific SNPs influencing the expression regulation of IGFs, we extended our analysis to include the trait of T2D, specifically finn-b-T2D. Interestingly, the IGF family displayed a contrasting impact on the regulation of T1D and T2D. After consolidating the data, we found that two IGF family molecules were linked to T2D. Notably, an elevated level of circulating IGF1 was associated with an increased risk of T2D onset (OR = 1.02, 95%CI = 1.000–1.030, *p* = 0.01, IVW method). In contrast, a higher serum concentration of IGFBP-6 exhibited a positive correlation with a reduced risk of T2D, as demonstrated by both the IVW approach (OR = 0.92, 95%CI = 0.87–0.98, *p* = 0.01) and the WM method (OR = 0.93, 95%CI = 0.87–1.00, *p* = 0.04). However, the remaining molecules did not provide compelling evidence to support a positive impact in assessing the causal influence of IGF family members on the risk of T2D (Figure 3). Scatter plots illustrating the MR analyses of the causal effects of IGFs on T1D with statistical significance are presented in Figure 4 (A for IGF-1 and B for IGFBP-6, respectively). MR Egger regression tests suggested no significant horizontal pleiotropy in this part. All the involved funnel plots, scatter plots and “leave-one out analysis” plots in assessing the association between IGFs family and T2D were shown in Supplementary file 2.

### Causal effects of adiponectin and INSR on the risk of diabetes

**Figure 5:**
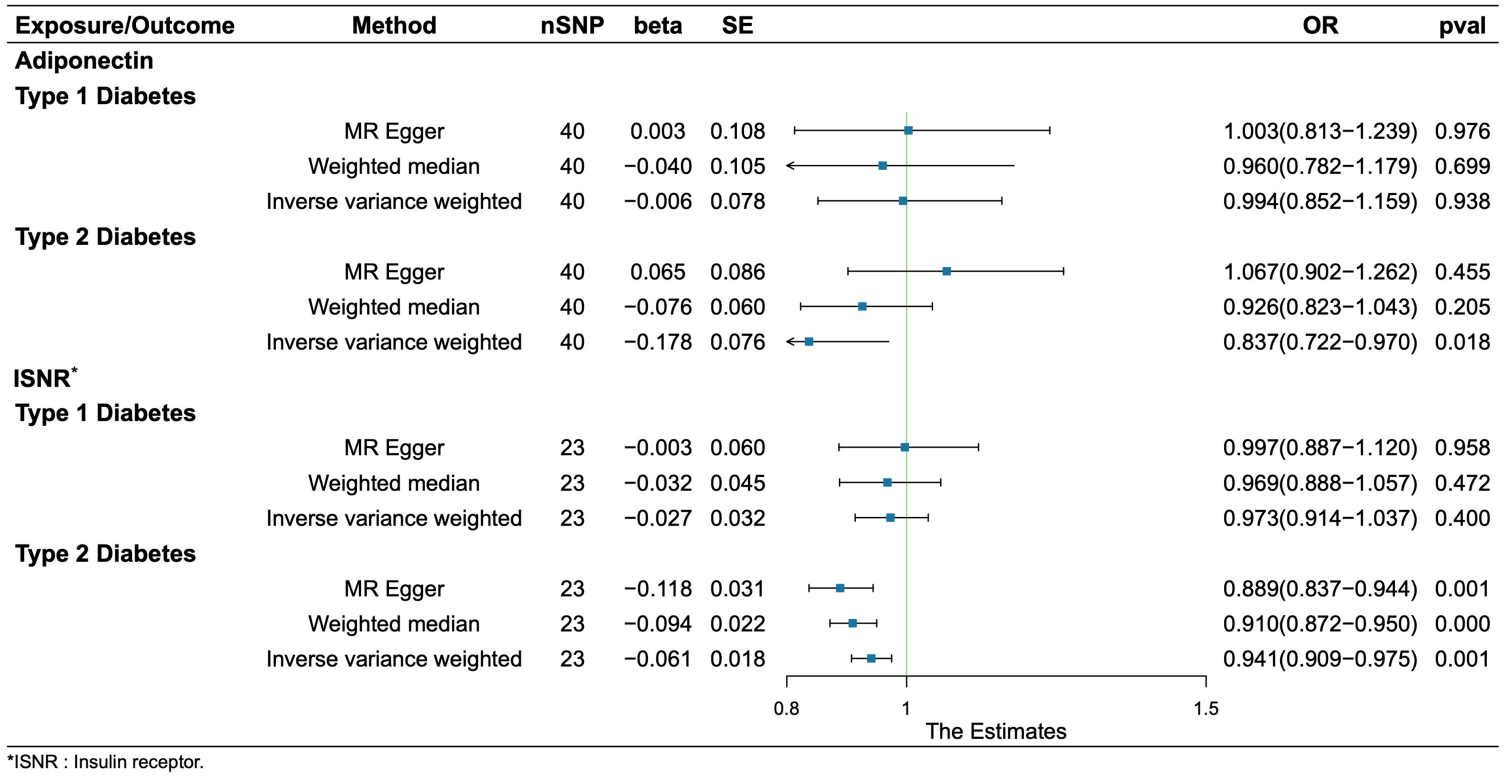
Associations between genetically cause of adiponectin/INSR levels and diabetes.

**Figure 6:**
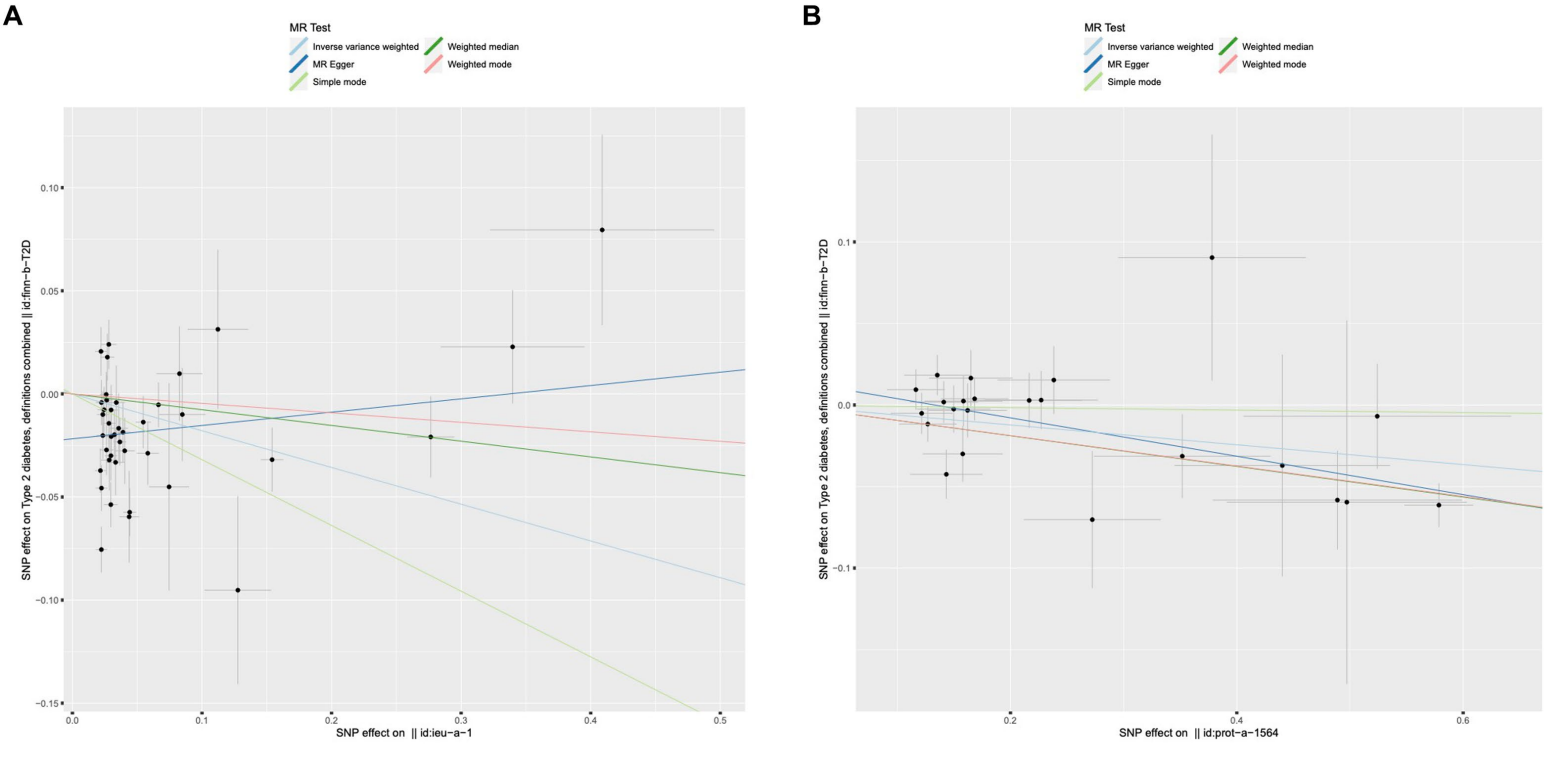
Scatter plots for MR analyses of the causal effect of adiponectin and INSR on T2D. **(A)** Adiponectin. **(B)** INSR. Analyses were conducted using the conventional IVW, MBE, WMM, MR-Egger, and MR.RAPS methods. The slope of each line corresponding to the estimated MR effect per method.

Regretfully, our analysis shows no evidence of causality from adiponectin and INSR to T1D. We found that adiponectin and INSR were associated with T2D. A higher serum Genetically lower levels of adiponectin was associated with an increased risk of Type 2 Diabetes (OR = 0.837, 95%CI = 0.721–0.970, *p* = 0.018, IVW method). Notably, INSR also demonstrated a negative correlation with T2D prevalence, as evidenced in among the MR Egger analysis (OR = 0.889, 95%CI = 0.837–0.944, *p* = 0.001), the WM analysis (OR = 0.910, 95%CI = 0.872–0.950, *p* = 1.52 × 10–5), and IVW analysis (OR = 0.941, 95%CI = 0.908–0.975, *p* = 6.68 × 10–4) (Figure 5). Scatter plots illustrating the analyses of the causal effects of adiponectin and INSR on T2D with statistical significance are presented in Figure 6. MR Egger regression tests suggested no significant horizontal pleiotropy in this part. All the involved funnel plots, scatter plots and “leave-one out analysis” plots in assessing the association between Adiponectin/INSR and T1D/T2D were shown in Supplementary files 3, 4.

## Discussion

In this study, we conducted two-sample MR analyses using multiple GWAS datasets to assess the relationship between individual IGF family members, adiponectin, INSR and diabetes. Our findings indicate that a genetically determined IGF1, IGFBP-5 and IGFBP-7 would reduce the risk of T1D. However, the IGF1 had been proved to be positively associated withT2D, while the level of IGFBP-6, adiponectin and INSR still decrease the possibility of T2D. To the best of our knowledge, our study represents the first comprehensive MR analysis systematically examining the associations between multiple IGF family members and T1D, T2D.

The IGF family comprises IGF-1, IGF-2, IGF receptors, and IGFBPs, playing a pivotal role in regulating growth, development, and various physiological processes. IGF-1 and IGF-2 serve as potent growth factors, stimulating cell growth and division while fostering the development of diverse tissues, including bone, muscle, and organs. Furthermore, IGF-1, which bears structural similarities to insulin, acts in an insulin-like manner, regulating glucose metabolism and enhancing glucose uptake in muscle and adipose tissue through downstream signaling via the PI3K-AKT and MAPK pathways. These pathways, in turn, govern cell survival, proliferation, and differentiation. IGFs can function both as endocrine hormones, originating from the liver and influencing distant tissues, and as paracrine/autocrine factors, produced locally and influencing neighboring cells.

Several strengths underpin our multiple MR analyses in this study. Initially, we leveraged a large-scale dataset encompassing diabetes and IGF family GWAS, a strategy that mitigates the impact of population stratification. Additionally, our study employed three distinct MR methods, enhancing the robustness of our results while guarding against reverse causal bias. We conducted several pleiotropic analyses to reduce the potential influence of pleiotropic factors on our MR results. Furthermore, we conducted sensitivity analyses utilizing the leave-one-out method, ensuring the stability of our MR findings (19).

In our study, we utilized summary statistics from GWAS to elucidate causal connections between the IGF family and diabetes, encompassing both T1D and T2D. Our findings indicated that IGF-1 exhibited a causal association with an increased risk of T2D, while IGFBP-6 displayed a tendency to decrease the risk of T2D. Simultaneously, our study unveiled that genetically lower levels of IGF-1 and IGFBP-5, as well as higher levels of IGFBP-7, were linked to an elevated susceptibility to T1D. More recently, Susanna C. Larsson and her colleagues conducted a Mendelian randomization study, incorporating data from 416 SNPs and 358,072 individuals, to investigate the association between IGF-1 levels and T2D in the UK Biobank, consisting of 74,124 T2D cases and 824,006 controls. Their research yielded evidence suggesting that increased IGF-1 levels might be causally associated with a higher risk of T2D (20). Another study by Wang et al. employed cluster Mendelian randomization analysis to identify distinct and opposing pathways of genetic influence between IGF-1 and T2D. Their investigation revealed that a higher IGF-1 level was associated with a reduced risk of T2D within specific clusters linked to genes in the growth hormone signaling pathway. Conversely, it was linked to an increased risk of T2D within clusters associated with genes involved in amino acid metabolism and genomic integrity (21). Besides, cytokine including adiponectin and INSR presented interesting association with diabetes due to the crucial correlation with IGF1 in our study. It was that adiponectin and INSR were negative associated with T2D, which reconfirmed the conclusion in previous studies and enhanced the relevance of IGF1 and diabetes. Adiponectin, the most prevalent peptide released by adipocytes, plays a prominent role in the intricate connection between adiposity and insulin resistance. Both animal and experimental research have demonstrated that adiponectin enhances insulin sensitivity, suggesting that it may serve as a preventive measure against the onset of T2DM (7, 22, 23). An observational study exploring the relationship between protein, specifically IGFBP2, and diabetes suggested an inverse association. However, a Mendelian randomization study failed to uncover any significant causal relationship between this protein and diabetes in either direction (24).

In the field of molecular epidemiology over the past few decades, obesity has been strongly linked to metabolic syndrome, including diabetes. Xu and colleagues, in their research, extracted instrumental variables for body mass index (BMI), waist-hip ratio (WHR), and BMI-adjusted WHR (WHRadjBMI) based on pooled statistics from genome-wide association studies. They successfully confirmed the causal effects of both overall and abdominal obesity on the risk of T2D and insulin resistance using a two-sample MR design (25, 26). The insulin/IGF-1 axis has emerged as a pivotal mediator in the connection between obesity and the risk of diabetes (25). Exploring therapeutic potential in metabolic disorders, researchers have identified several components within the IGF-IGFBP system. Notably, both IGFBP-1 and IGFBP-2 have demonstrated significant associations with insulin sensitivity in humans (10). Previous studies have measured serum levels of IGF-1, IGF-2, and IGFBP in an age-matched cross-sectional cohort of 305 pediatric and adolescent participants with varying degrees of T1D risk. This research revealed that lower levels of IGF1 and IGF2 were associated with a higher incidence of T1DM, aligning with our findings (20, 27, 28). During fetal development and childhood, IGFs play a critical role in promoting the normal growth and development of various tissues and organs. The distinctive biological features between T1D and T2D imply that different mechanisms contribute to the controversial regulation of IGF-1 in these two types of diabetes.

Consequently, our results underscore the notion that excessive growth during childhood and adolescence can lead to long-term programmed metabolic and inflammatory disorders, elevating the risk of T2D. This validates the hypothesis that early-life exposure to adverse environmental factors contributes to metabolic diseases in adulthood. Thus, the importance of glucose monitoring in whom with alterations of relevant IGF family members was alerted. Moreover, the identified proteins were promising for development of early screening tools for diabetes, while they might be appealing targets for treatment. Future studies are needed to validate these cytokines as predictive biomarkers in longitudinal case–control diabetes cohorts. However, such studies are often strongly influenced by confounding and reverse causation (29).

## Conclusion

In summary, our investigation, utilizing GWAS summary datasets related to diabetes and circulating IGF family members, has unveiled causal relationships between specific IGF family members and T1D and T2D through MR analysis. Generally, the IGF family appears to reduce the risk of T1D, but it presents a more complex and controversial role in the context of T2D. These findings provide compelling evidence that T2D is intricately linked with developmental impairment. Our findings have suggested a correlation between the variations of relevant cytokines especially IGF1 in earlier period and pathogenesis of diabetes, furthermore, have alerted the importance of glucose monitoring in whom with alterations of relevant IGF family members and provided potential targets for early treatment such as IGF-1/IGF-1R inhibitors in clinical practice. However, further exploration of the molecular mechanism involved in this study was needed in this field. They also underscore the potential involvement of developmental pathological effects in the onset of diabetes, emphasizing the need for further observational and experimental studies in this field.

## Data availability statement

The original contributions presented in the study are included in the article/Supplementary material, further inquiries can be directed to the corresponding author.

## Author contributions

YL: Conceptualization, Formal analysis, Funding acquisition, Investigation, Methodology, Project administration, Supervision, Validation, Visualization, Writing – review & editing. XiL: Data curation, Formal analysis, Investigation, Methodology, Software, Writing – original draft. JT: Data curation, Formal analysis, Investigation, Methodology, Software, Writing – original draft. SL: Formal analysis, Investigation, Methodology, Software, Supervision, Validation, Writing – original draft. XuL: Conceptualization, Formal analysis, Investigation, Project administration, Supervision, Validation, Visualization, Writing – review & editing.

## Footnotes

^1^https://github.com/bulik/ldsc
