# Supplementary material for: Mendelian randomization analysis demonstrates the causal effects of IGF family members in diabetes
Source: Front Med (Lausanne). 2024 Feb 5;11:1332162. doi: 10.3389/fmed.2024.1332162 (PMC10875044; doi:10.3389/fmed.2024.1332162)
Supplement: Supplementary file 1 [file Data_Sheet_1.PDF]

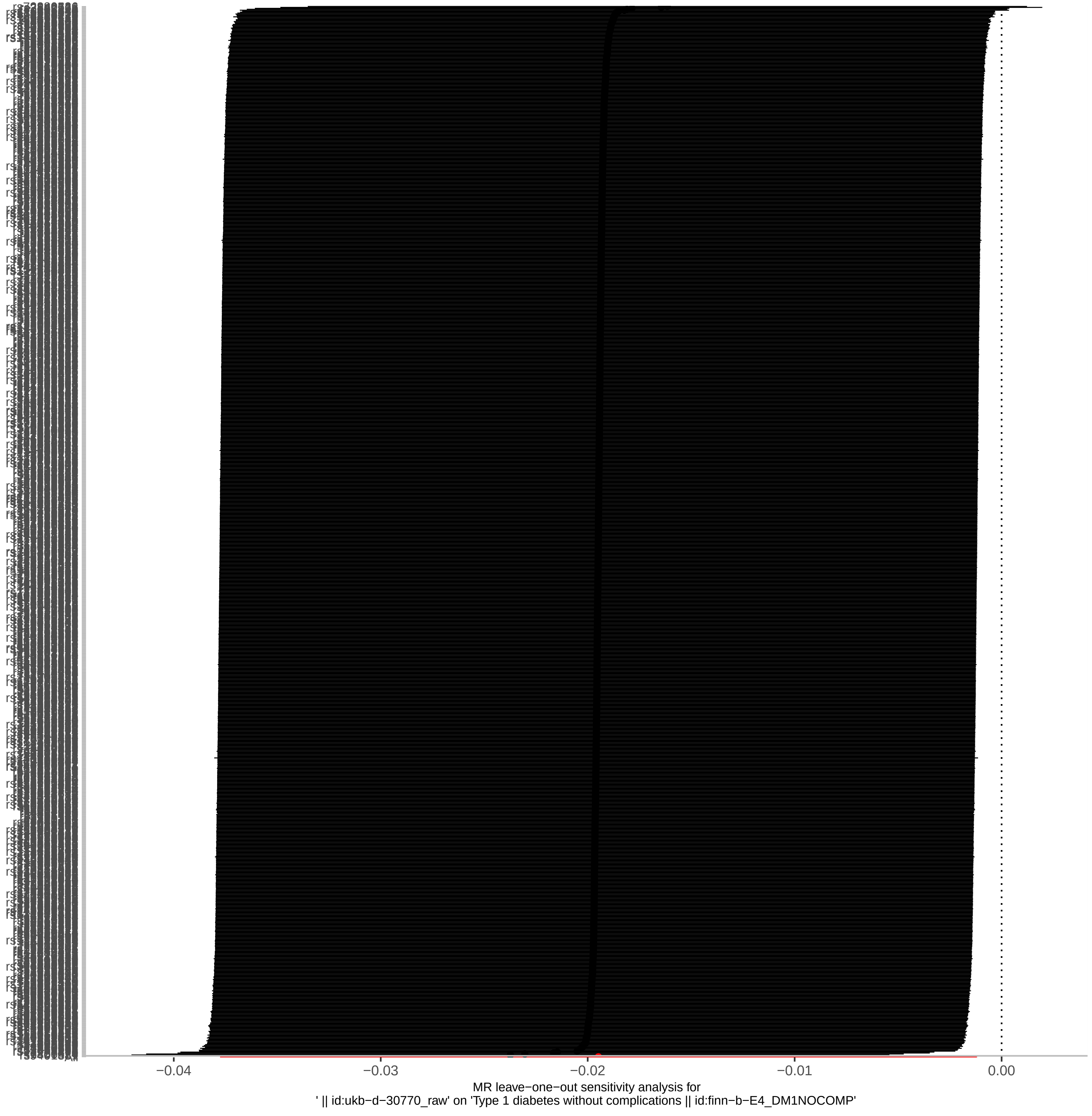
−0.04
−0.03
−0.02
−0.01
0.00
MR leave−one−out sensitivity analysis for
' || id:ukb−d−30770_raw' on 'Type 1 diabetes without complications || id:finn−b−E4_DM1NOCOMP'

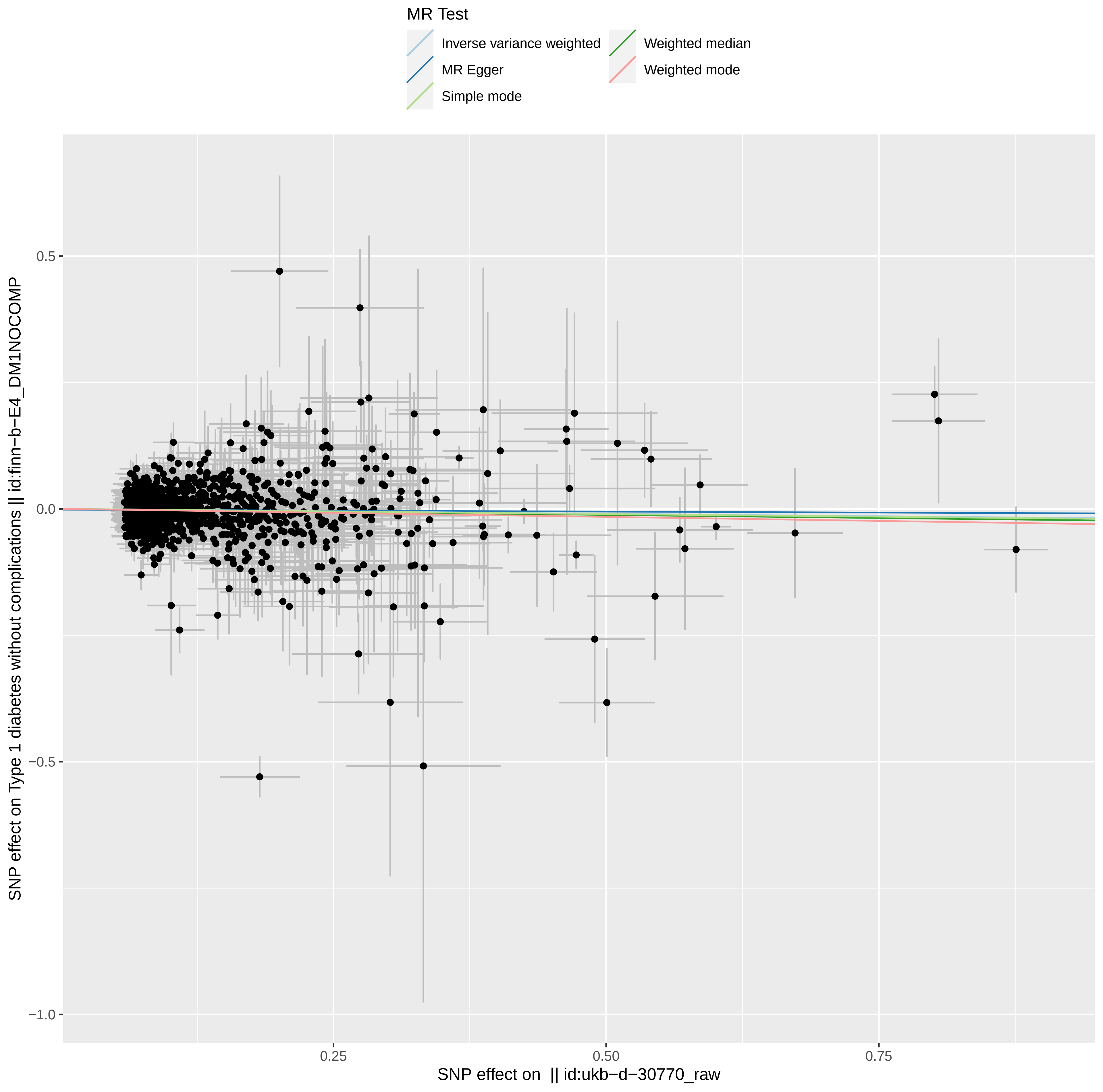
MR Test
Inverse variance weighted
MR Egger
Simple mode
Weighted median
Weighted mode
SNP effect on Type 1 diabetes without complications || id:finn-b-E4_DM1NOCOMP
0.5
0.0
−0.5
−1.0
0.25
0.50
0.75
SNP effect on || id:ukb-d-30770_raw

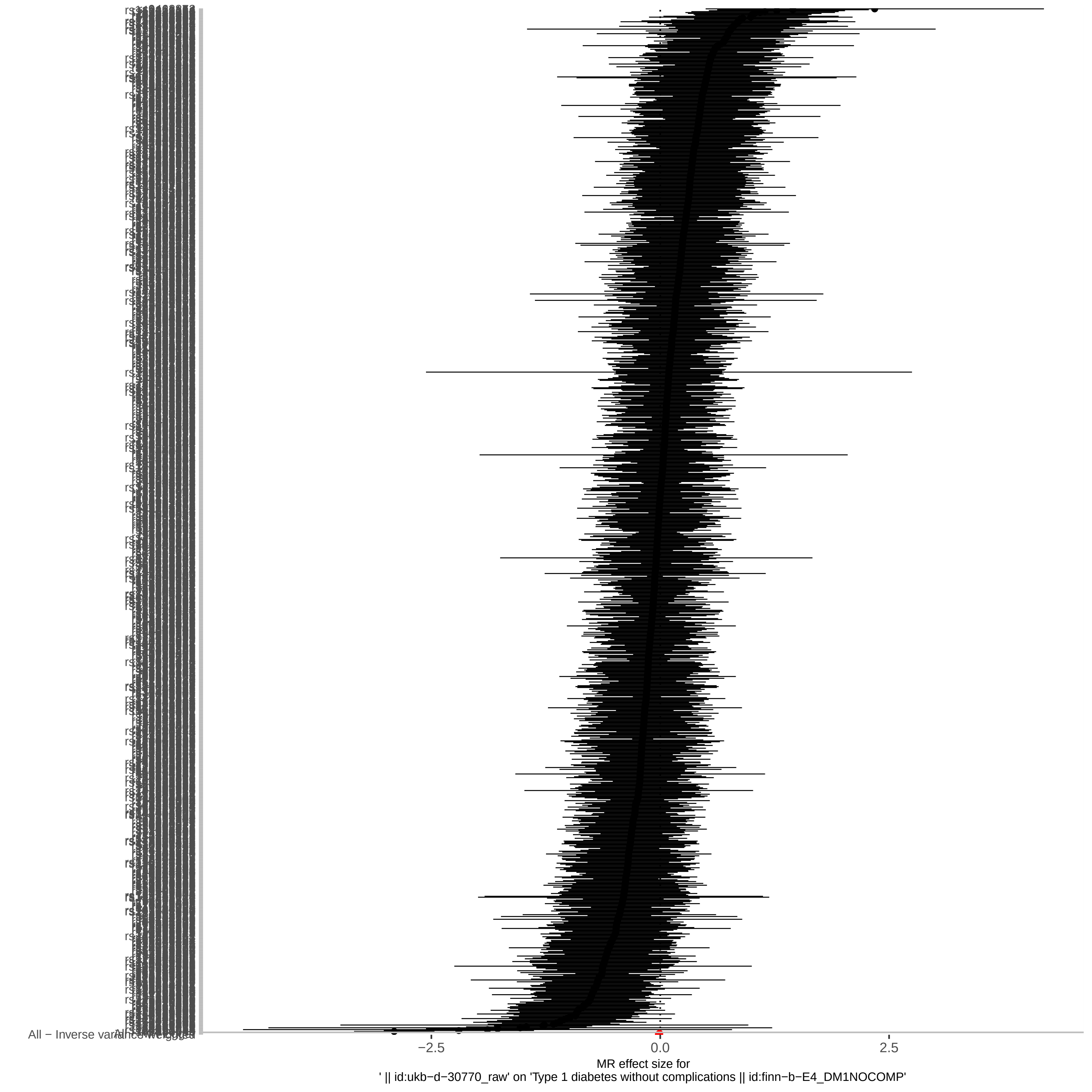
All − Inverse variance weighted
−2.5
0.0
2.5
MR effect size for
' || id:ukb−d−30770_raw' on 'Type 1 diabetes without complications || id:finn−b−E4_DM1NOCOMP'

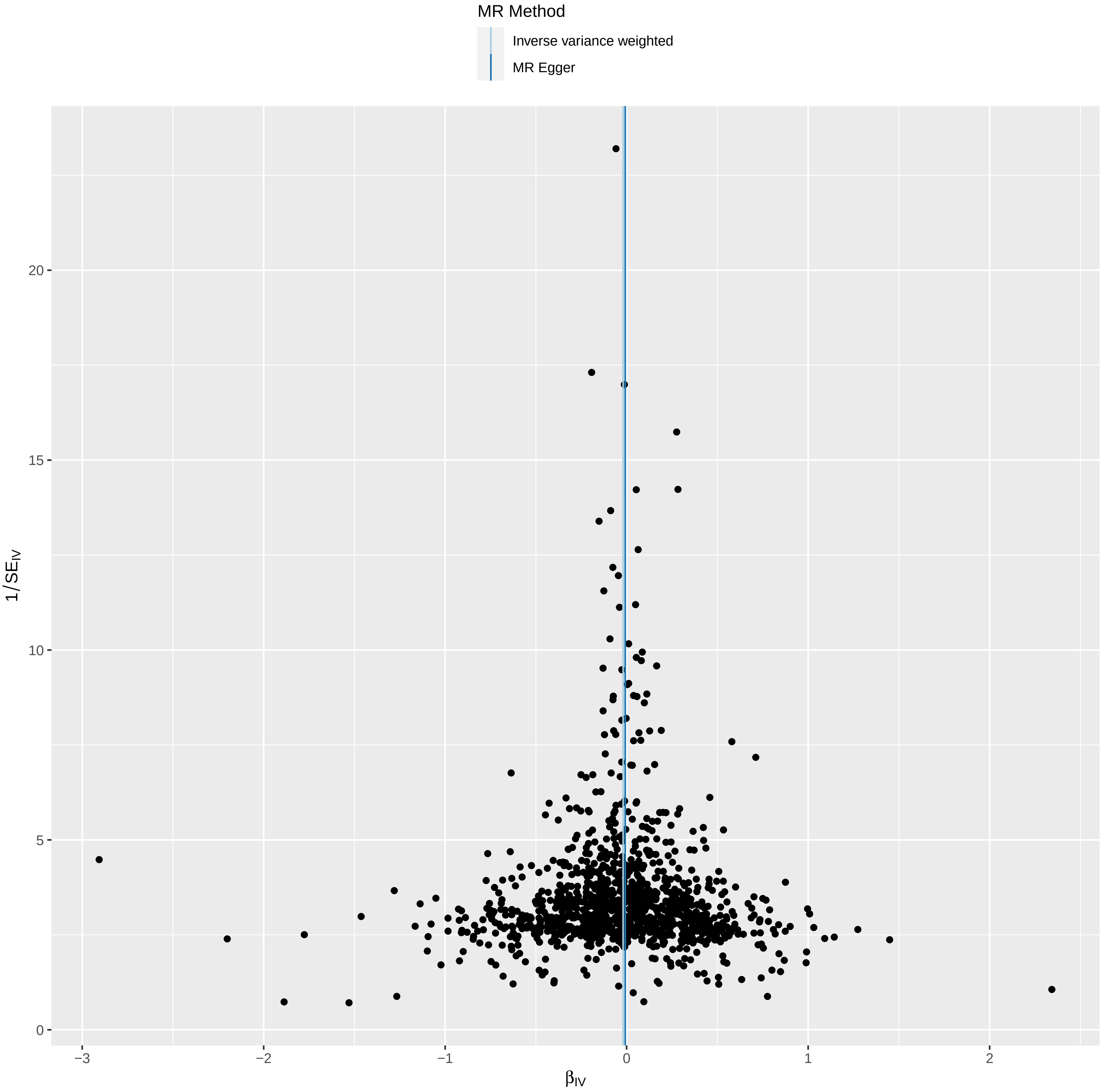
MR Method
Inverse variance weighted
MR Egger
1/SE_IV
β_IV
20
15
10
5
0
−3
−2
−1
0
1
2

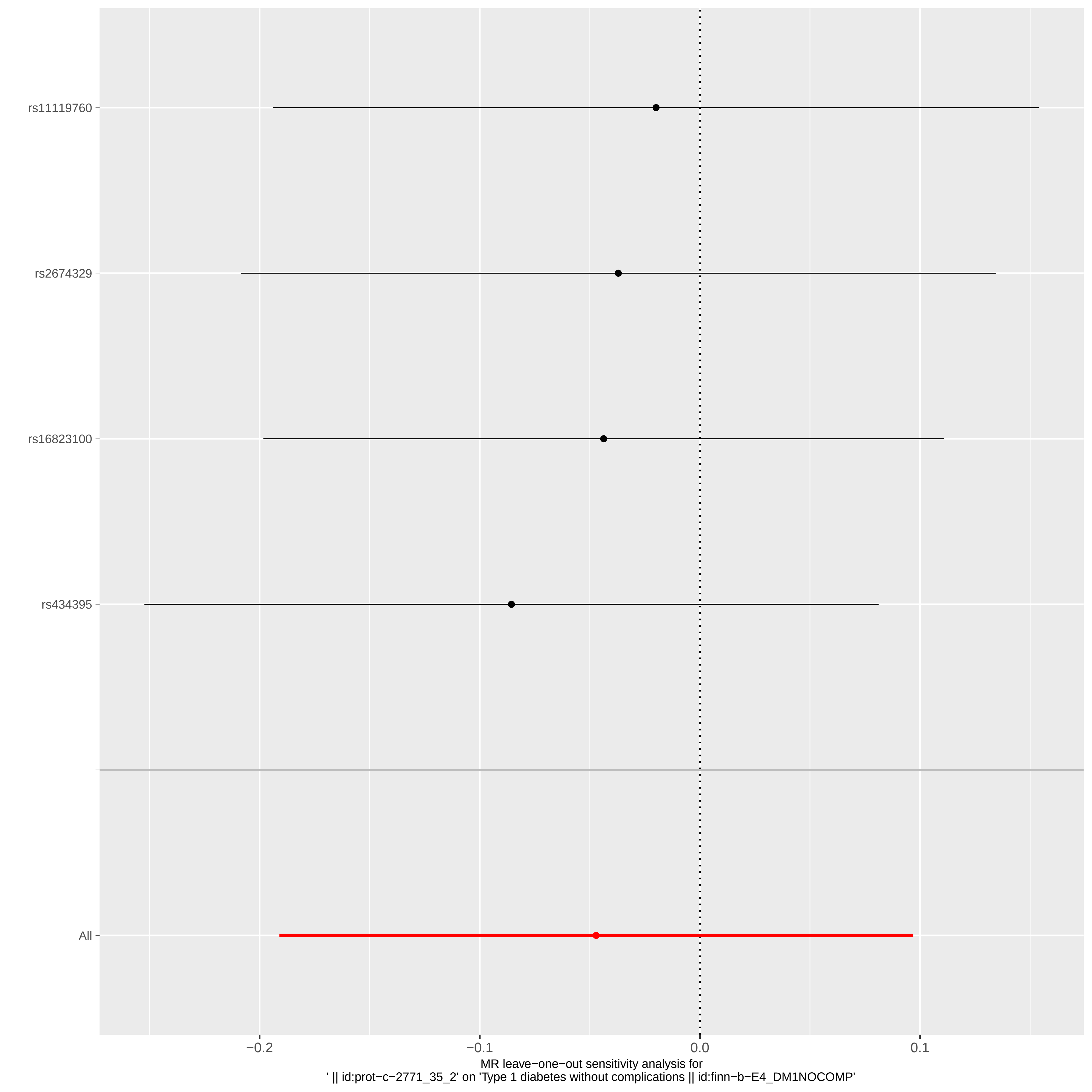

rs11119760
rs2674329
rs16823100
rs434395
All
−0.2
−0.1
0.0
0.1
MR leave−one−out sensitivity analysis for
' || id:prot−c−2771_35_2' on 'Type 1 diabetes without complications || id:finn−b−E4_DM1NOCOMP'

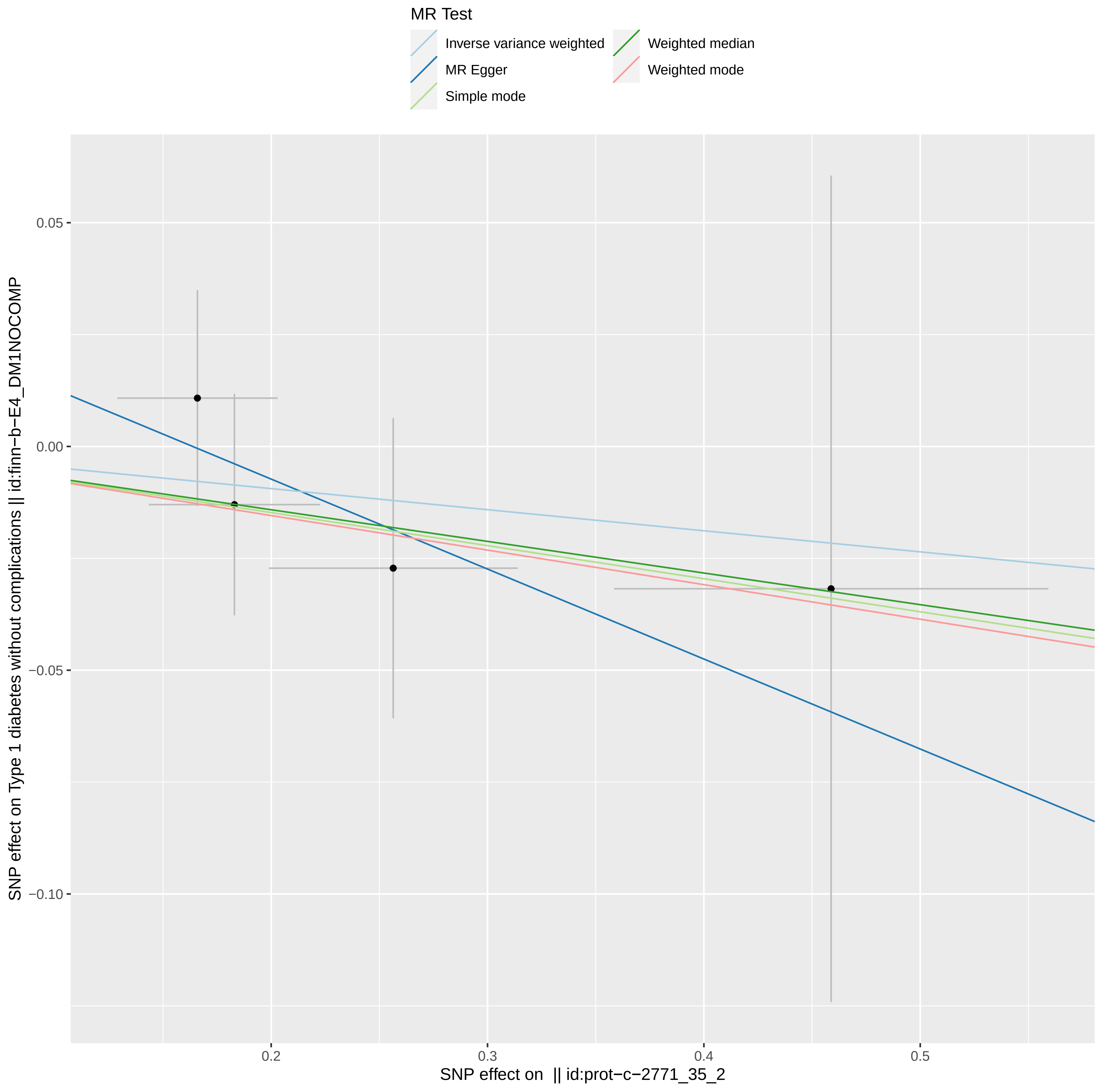
MR Test
Inverse variance weighted
MR Egger
Simple mode
Weighted median
Weighted mode
0.05
0.00
−0.05
−0.10
SNP effect on Type 1 diabetes without complications || id:finn−b−E4_DM1NOCOMP
0.2
0.3
0.4
0.5
SNP effect on || id:prot−c−2771_35_2

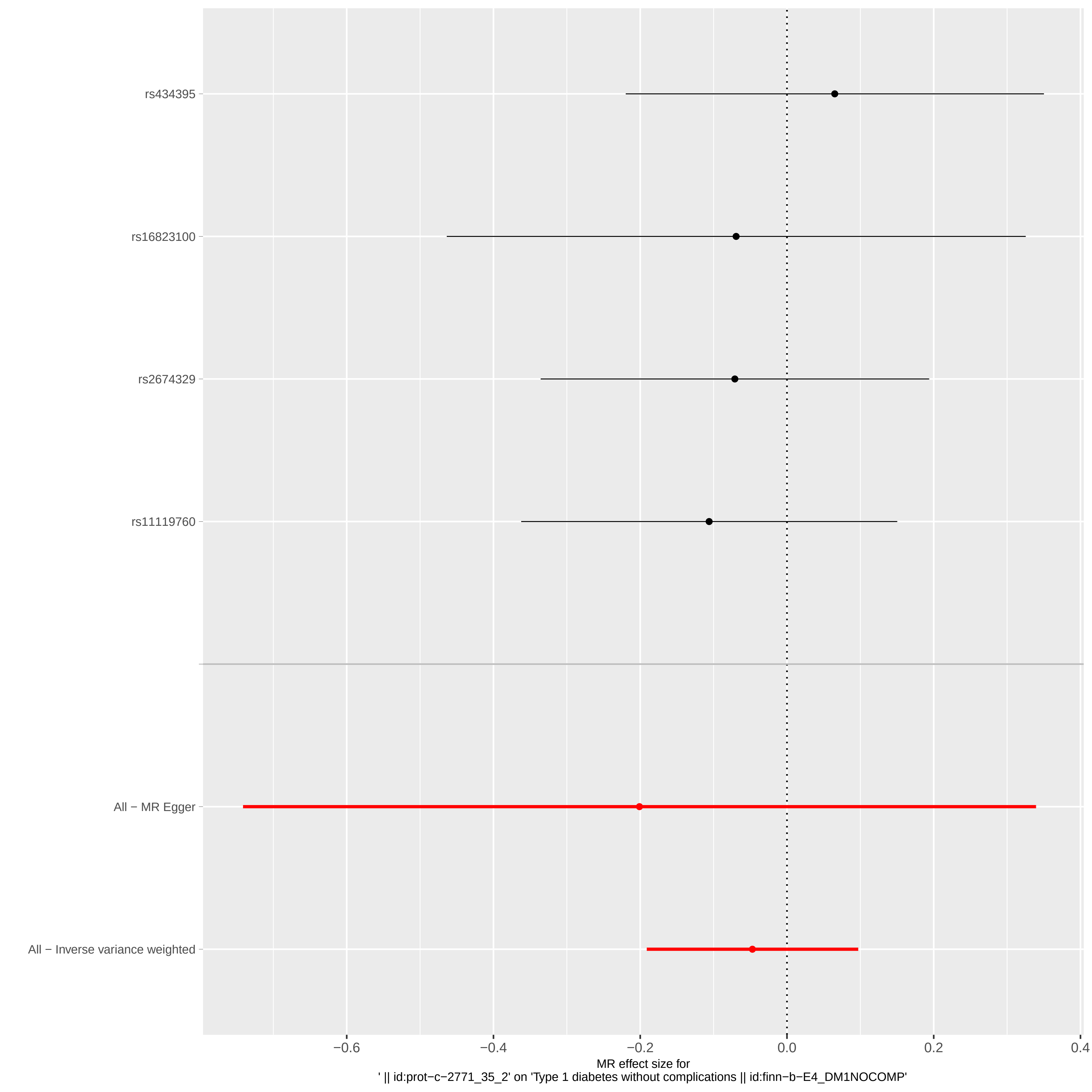

rs434395
rs16823100
rs2674329
rs11119760
All − MR Egger
All − Inverse variance weighted
−0.6
−0.4
−0.2
0.0
0.2
0.4
MR effect size for
' || id:prot−c−2771_35_2' on 'Type 1 diabetes without complications || id:finn−b−E4_DM1NOCOMP'

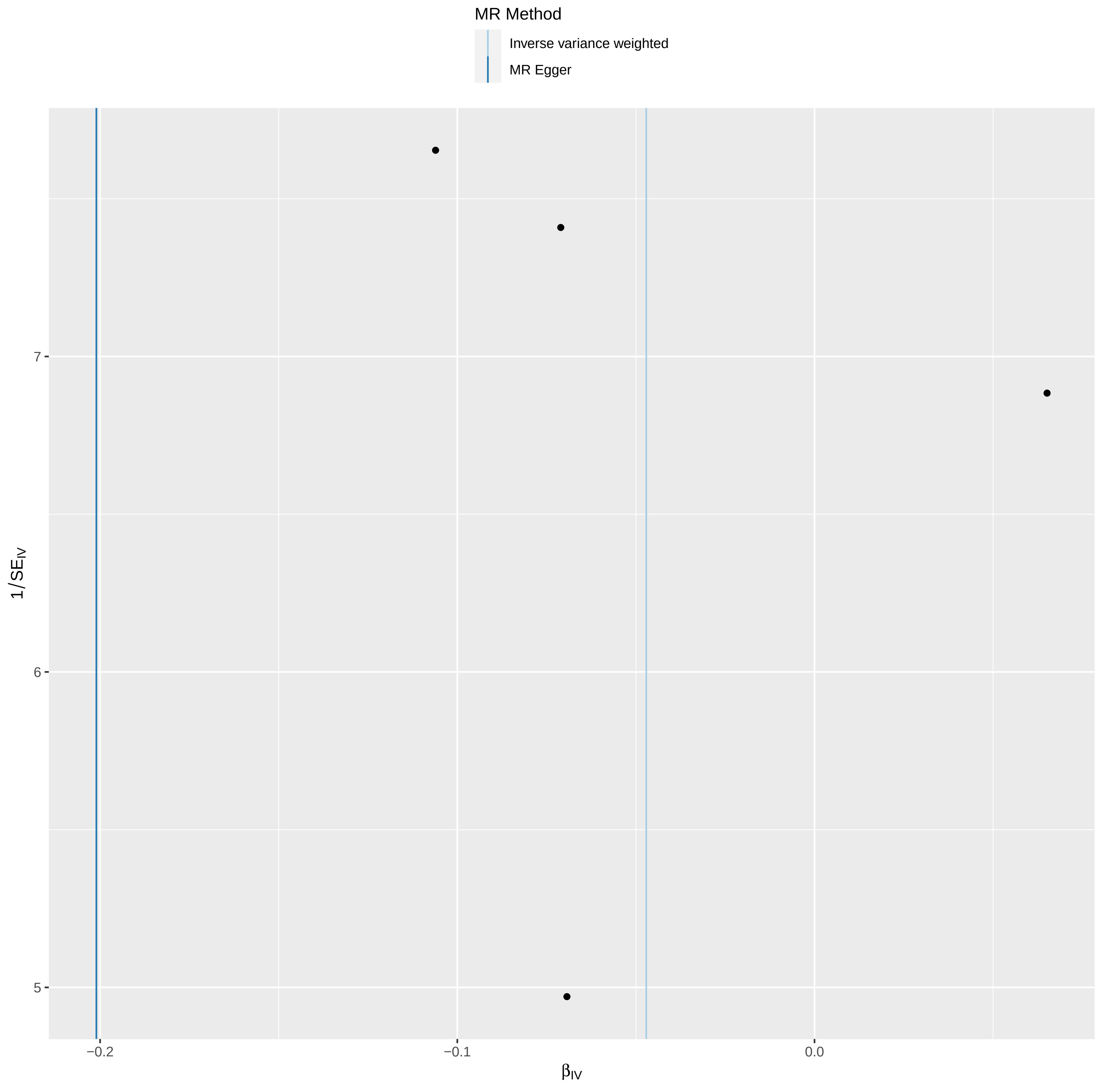

MR Method
Inverse variance weighted
MR Egger
7
6
5
1/SE_IV
−0.2
−0.1
0.0
β_IV

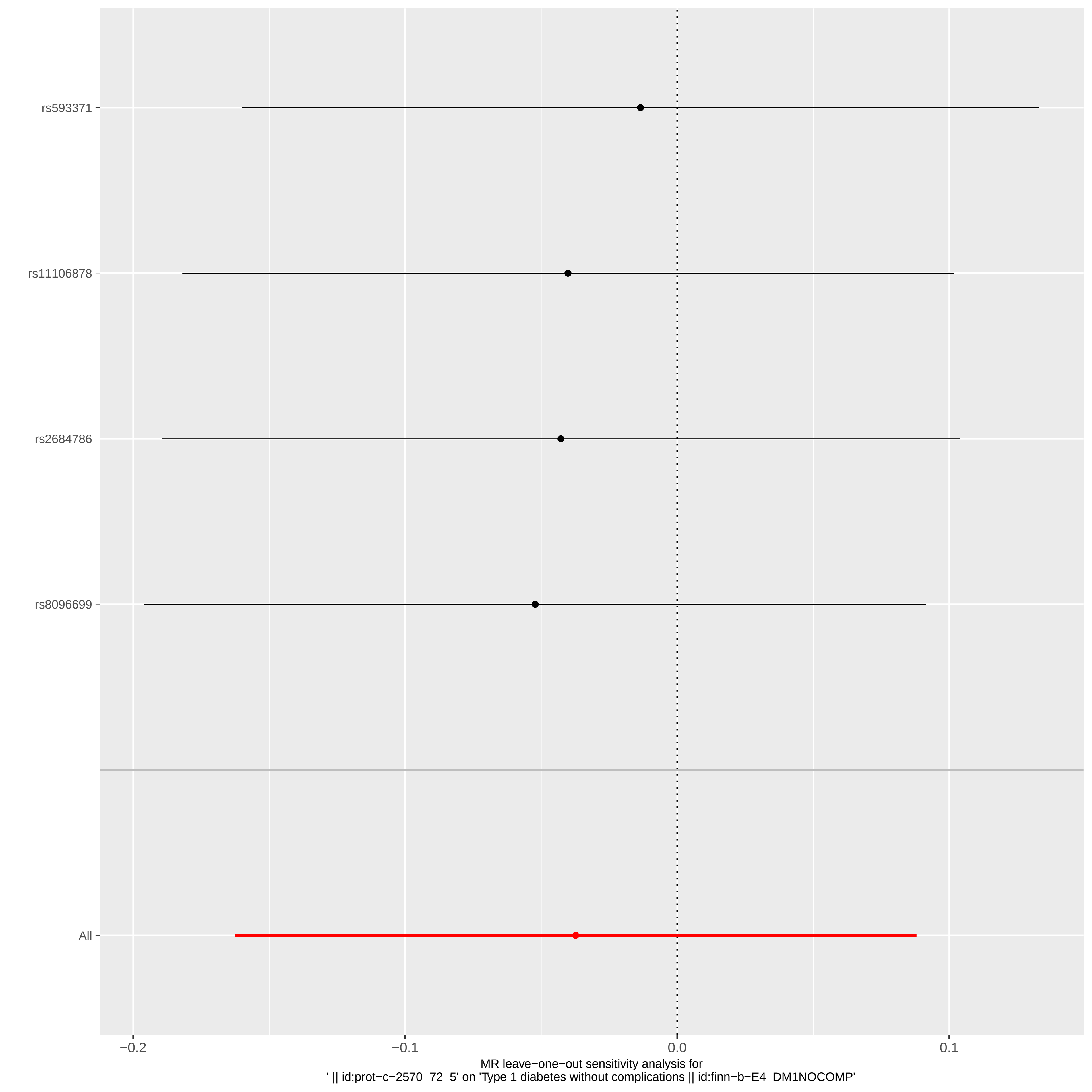
rs593371
rs11106878
rs2684786
rs8096699
All
−0.2
−0.1
0.0
0.1
MR leave−one−out sensitivity analysis for
' || id:prot−c−2570_72_5' on 'Type 1 diabetes without complications || id:finn−b−E4_DM1NOCOMP'

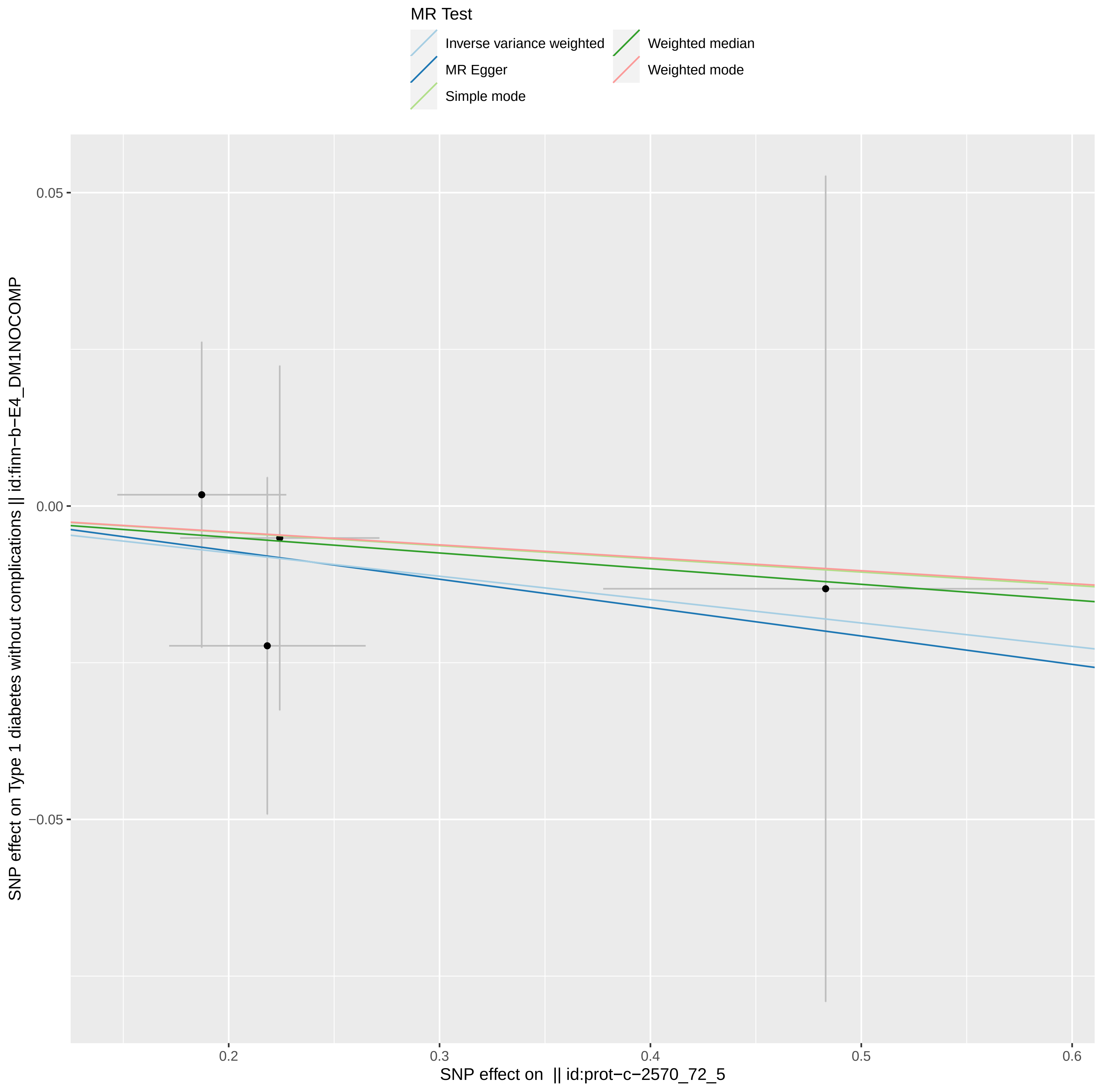

MR Test
Inverse variance weighted
MR Egger
Simple mode
Weighted median
Weighted mode
0.05
0.00
−0.05
0.2
0.3
0.4
0.5
0.6
SNP effect on Type 1 diabetes without complications || id:finn−b−E4_DM1NOCOMP
SNP effect on || id:prot−c−2570_72_5

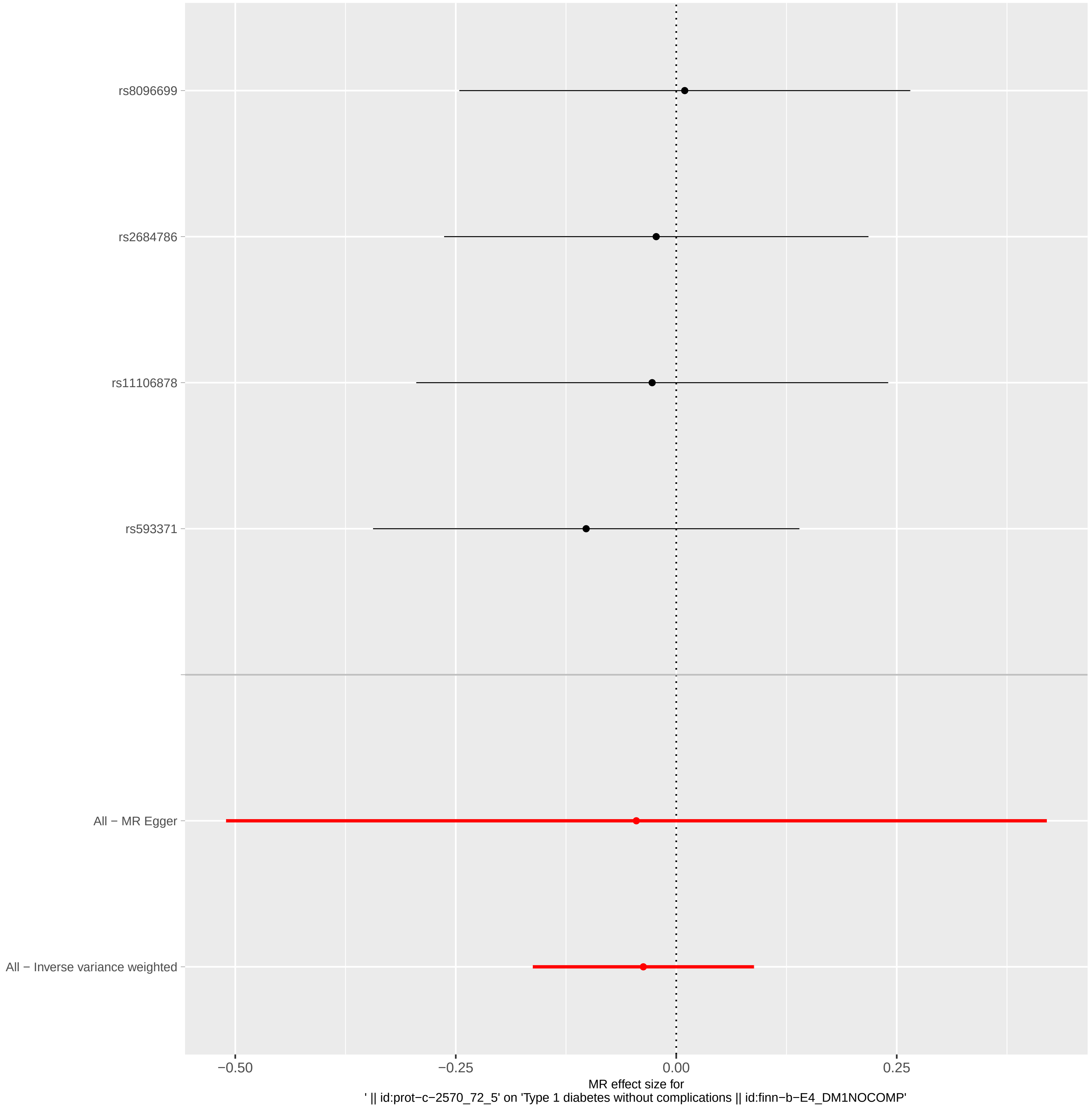
rs8096699
rs2684786
rs11106878
rs593371
All − MR Egger
All − Inverse variance weighted
−0.50
−0.25
0.00
0.25
MR effect size for
' || id:prot−c−2570_72_5' on 'Type 1 diabetes without complications || id:finn−b−E4_DM1NOCOMP'

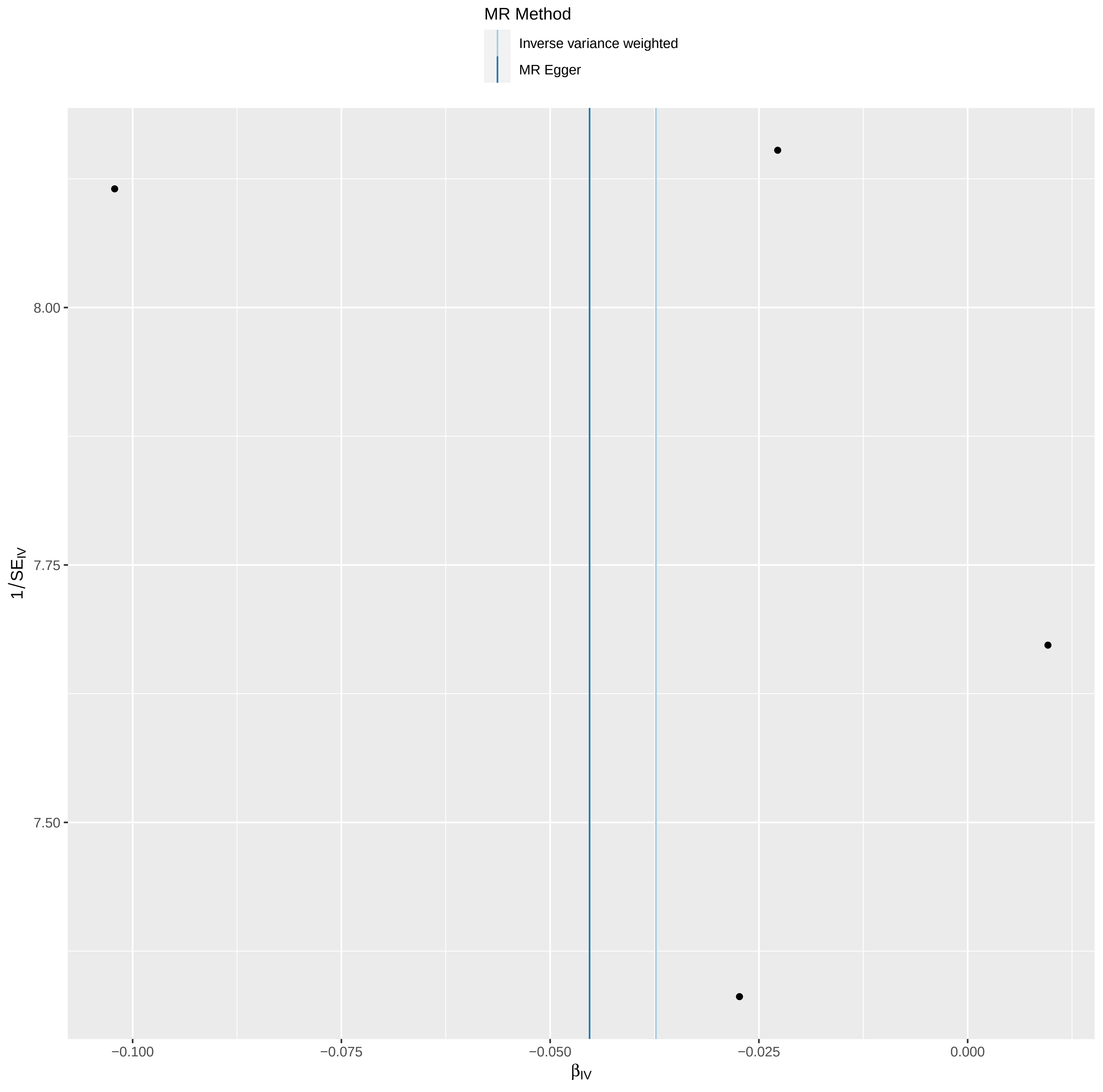

MR Method
Inverse variance weighted
MR Egger
8.00
7.75
7.50
1/SE$_{IV}$
−0.100
−0.075
−0.050
−0.025
0.000
β$_{IV}$

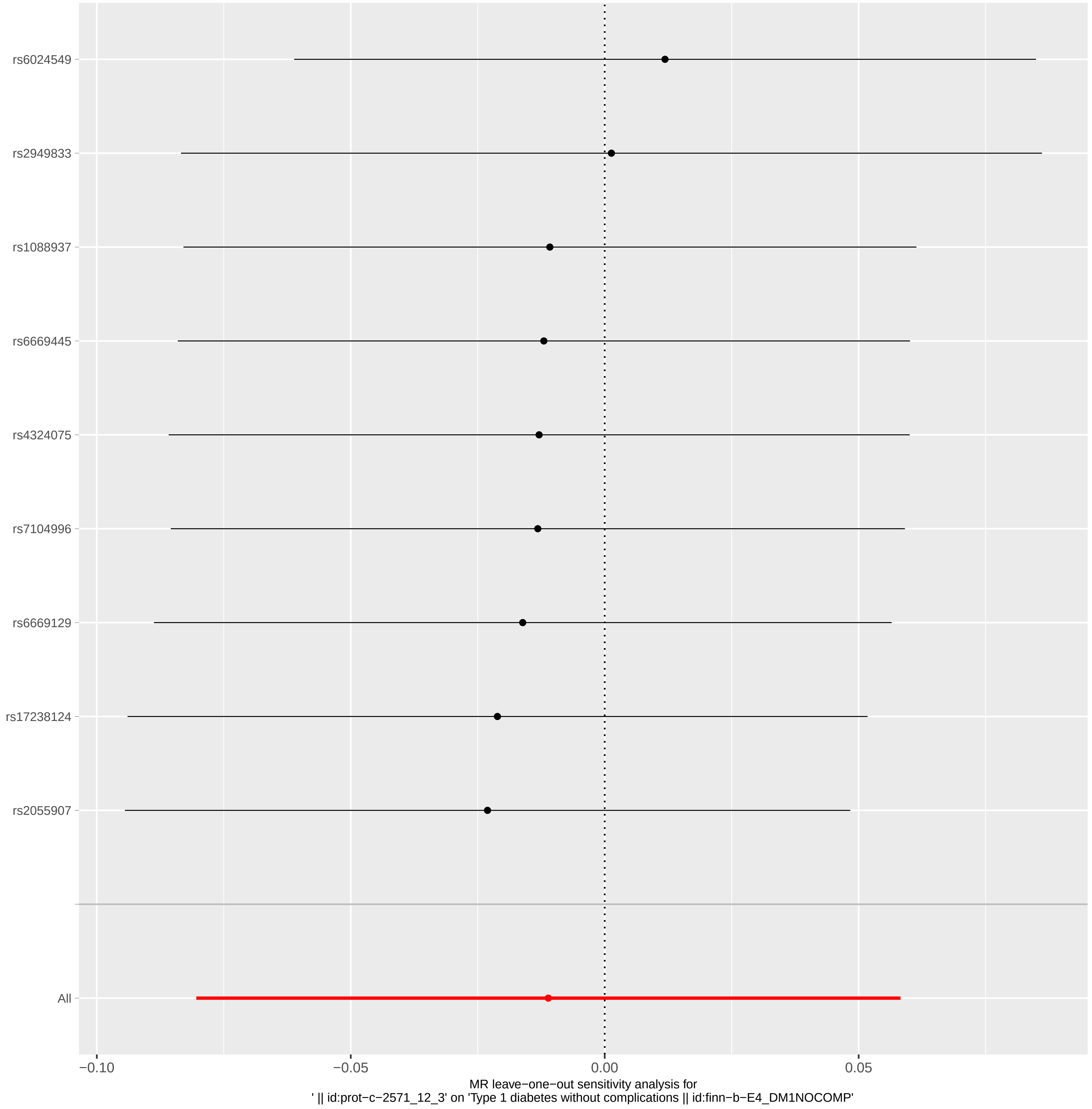
rs6024549
rs2949833
rs1088937
rs6669445
rs4324075
rs7104996
rs6669129
rs17238124
rs2055907
All
−0.10
−0.05
0.00
0.05
MR leave−one−out sensitivity analysis for
' || id:prot−c−2571_12_3' on 'Type 1 diabetes without complications || id:finn−b−E4_DM1NOCOMP'

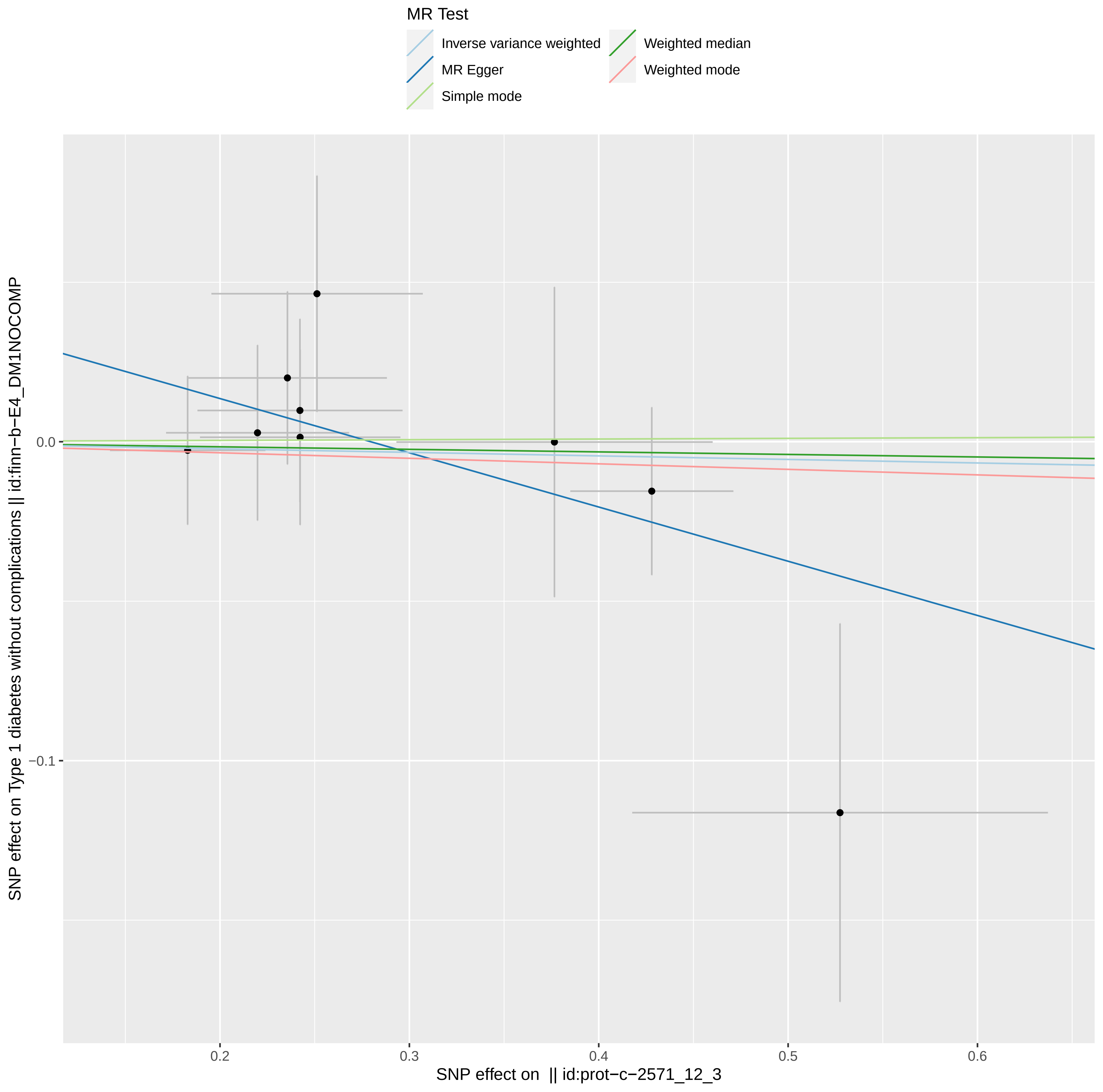
MR Test
Inverse variance weighted
MR Egger
Simple mode
Weighted median
Weighted mode
SNP effect on Type 1 diabetes without complications || id:finn−b−E4_DM1NOCOMP
0.0
−0.1
0.2
0.3
0.4
0.5
0.6
SNP effect on || id:prot−c−2571_12_3

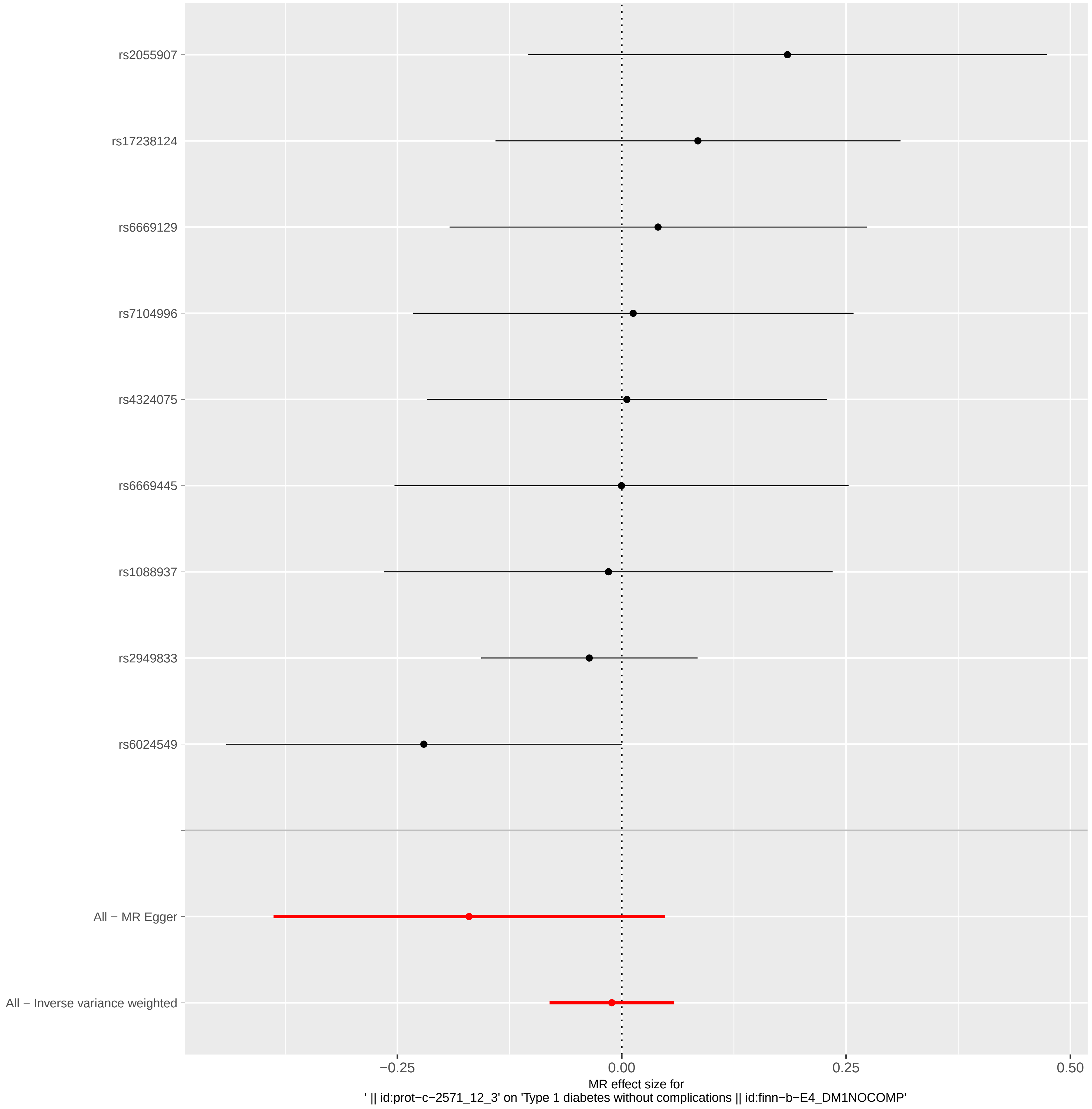

rs2055907
rs17238124
rs6669129
rs7104996
rs4324075
rs6669445
rs1088937
rs2949833
rs6024549
All − MR Egger
All − Inverse variance weighted
−0.25
0.00
0.25
0.50
MR effect size for
' || id:prot−c−2571_12_3' on 'Type 1 diabetes without complications || id:finn−b−E4_DM1NOCOMP'

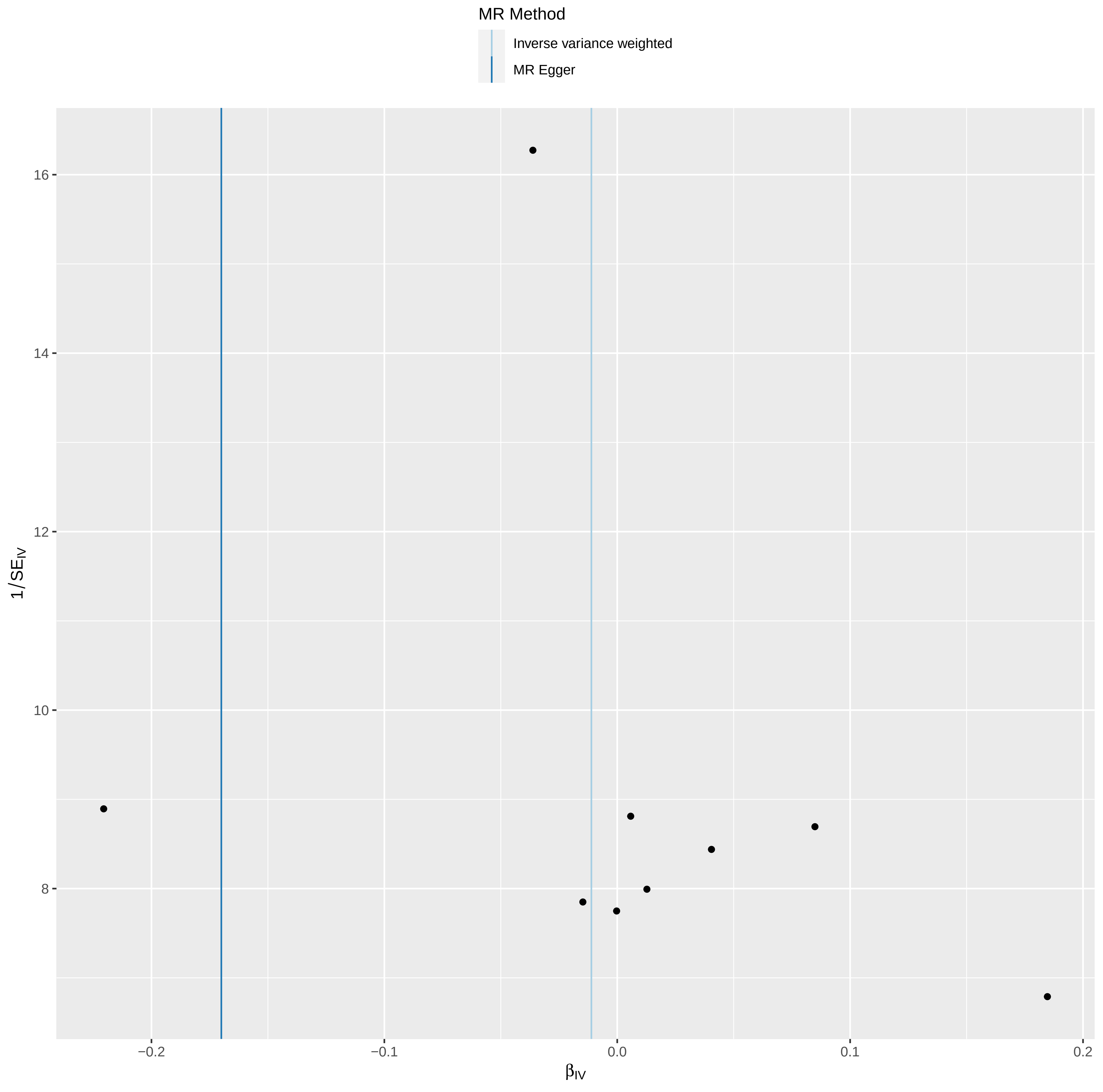

MR Method
Inverse variance weighted
MR Egger
16
14
12
10
8
1/SE$_{IV}$
−0.2
−0.1
0.0
0.1
0.2
β$_{IV}$

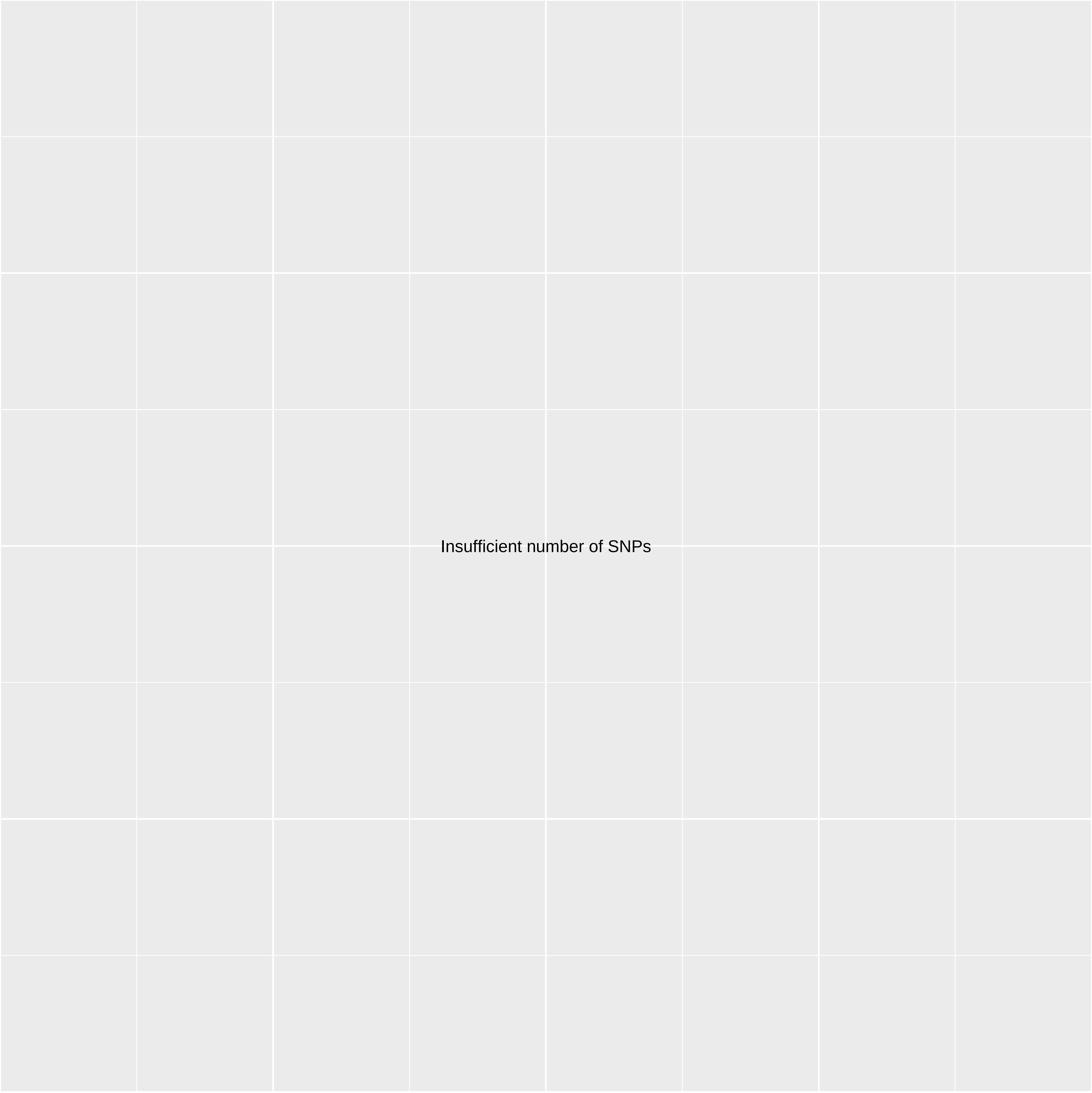
Insufficient number of SNPs

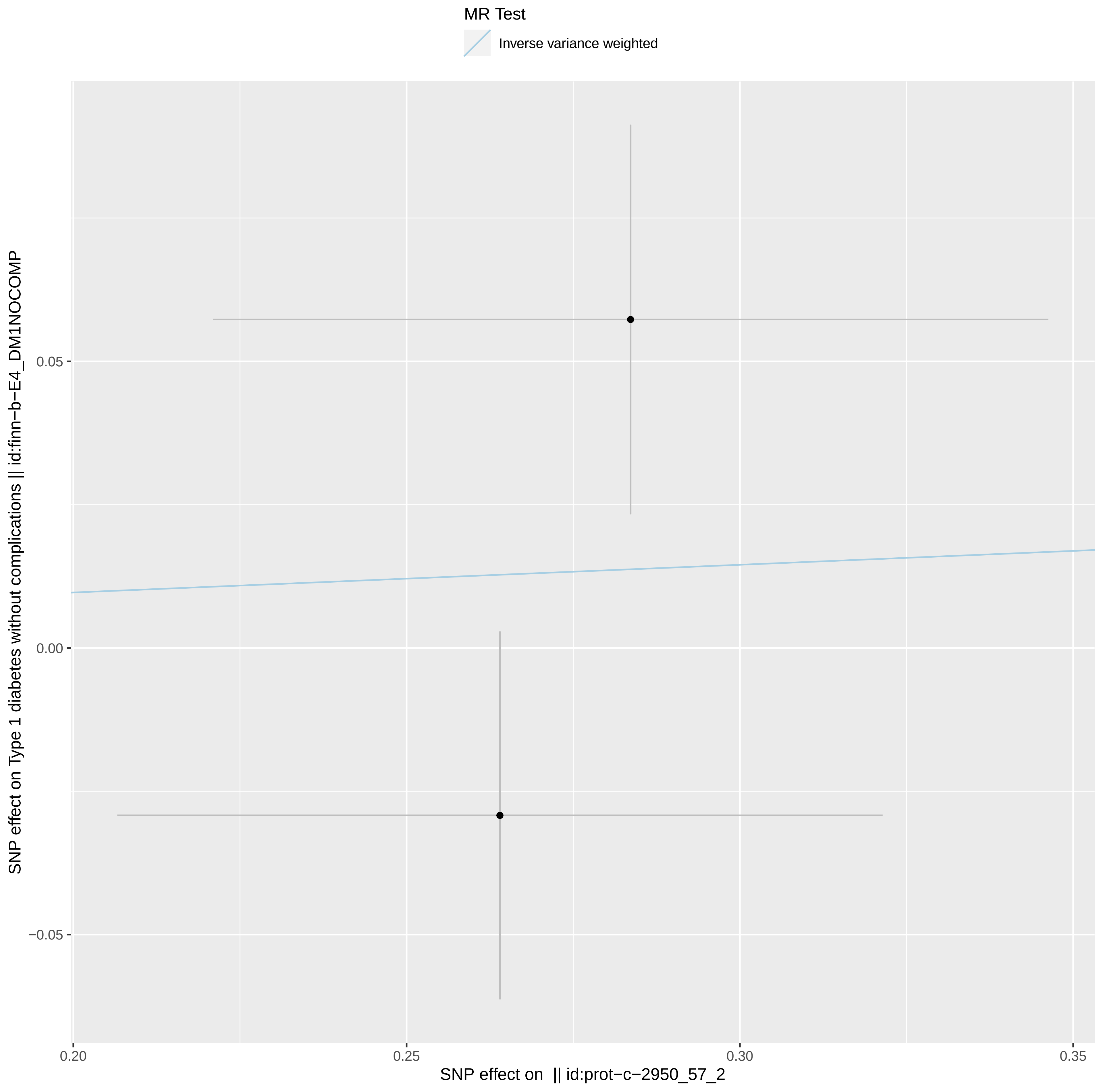

MR Test
Inverse variance weighted
SNP effect on Type 1 diabetes without complications || id:finn−b−E4_DM1NOCOMP
0.05
0.00
−0.05
0.20
0.25
0.30
0.35
SNP effect on || id:prot−c−2950_57_2

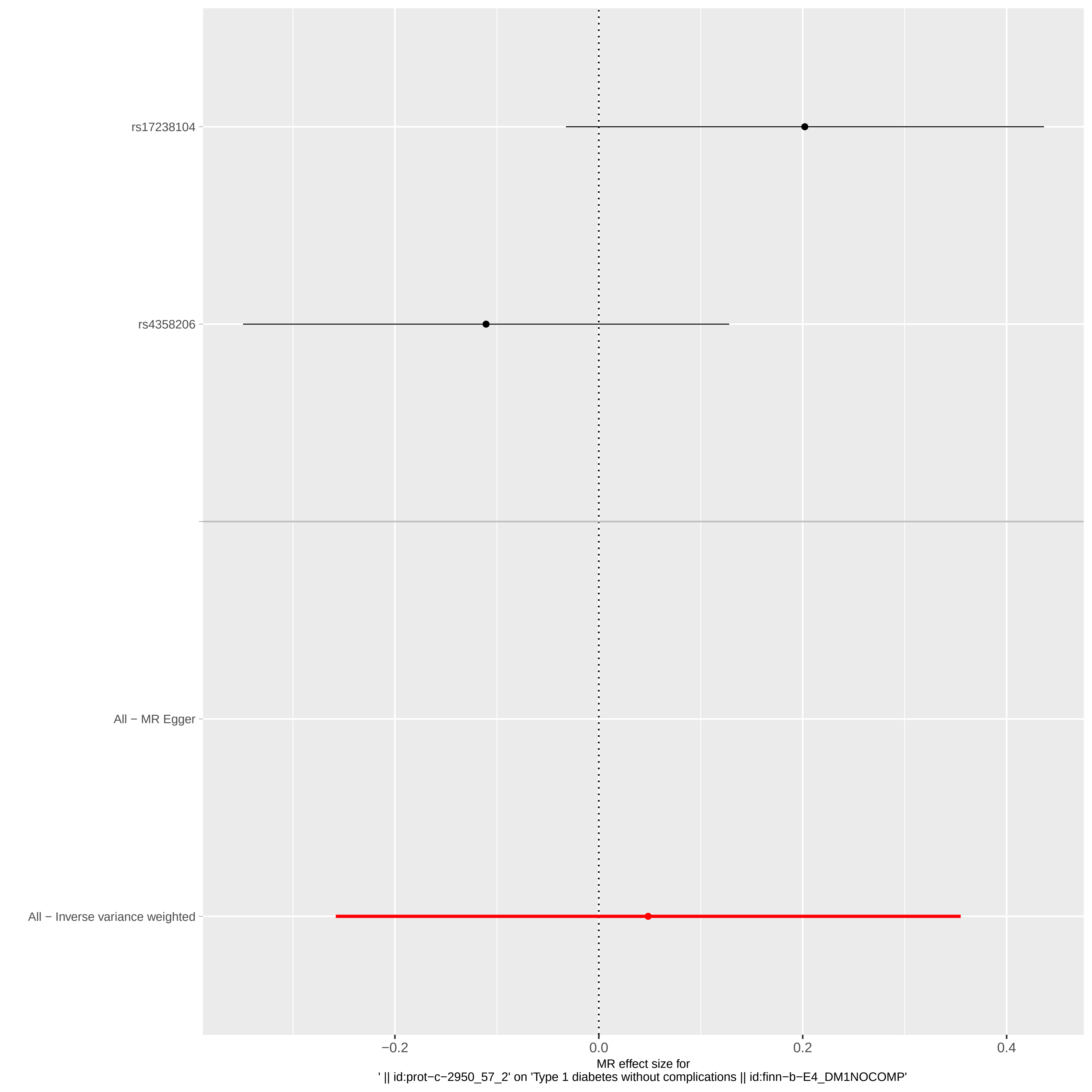
rs17238104
rs4358206
All − MR Egger
All − Inverse variance weighted
−0.2
0.0
0.2
0.4
MR effect size for
' || id:prot−c−2950_57_2' on 'Type 1 diabetes without complications || id:finn−b−E4_DM1NOCOMP'

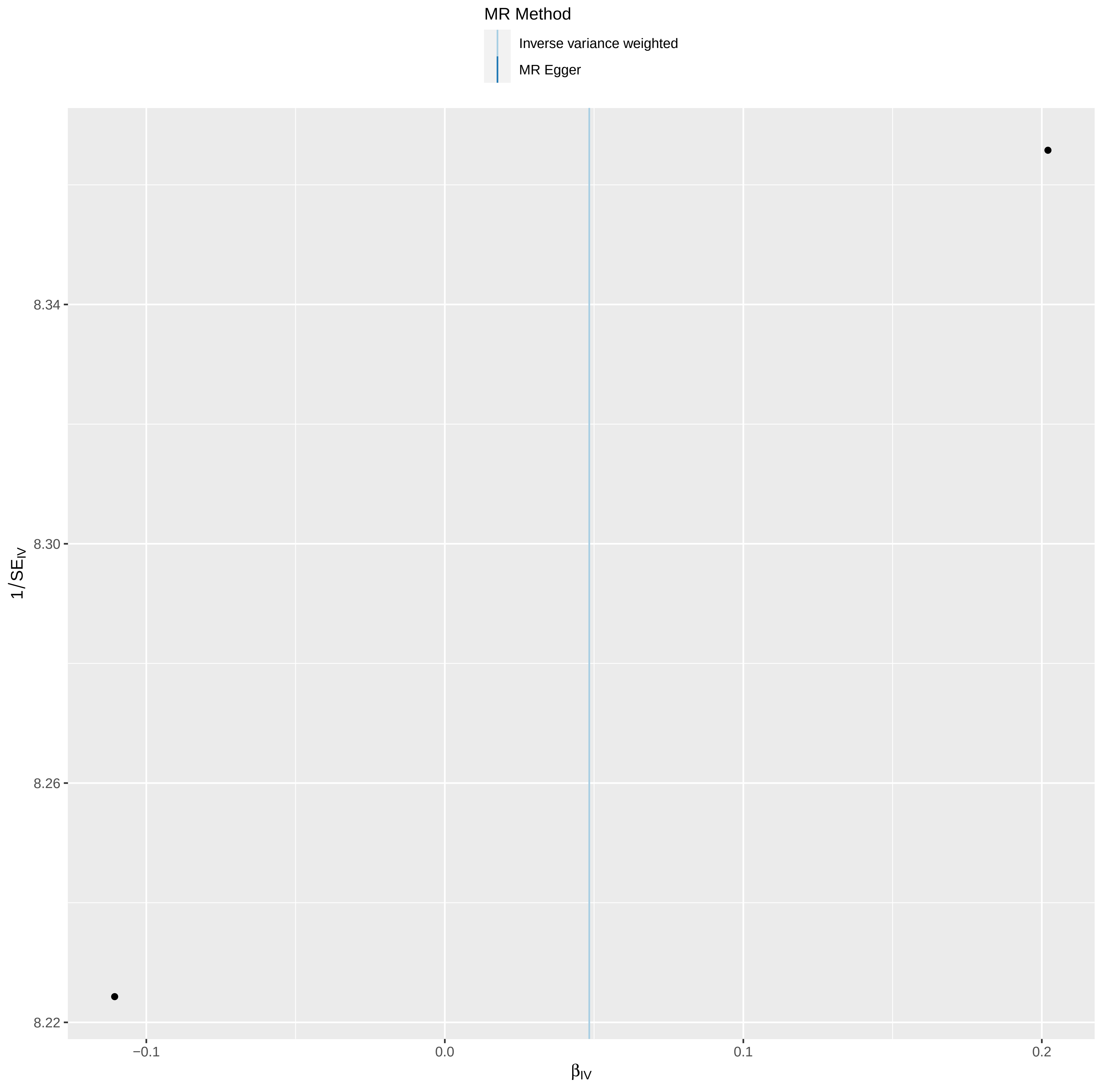
MR Method
Inverse variance weighted
MR Egger
8.34
8.30
8.26
8.22
$1/SE_{IV}$
−0.1
0.0
0.1
0.2
β_{IV}

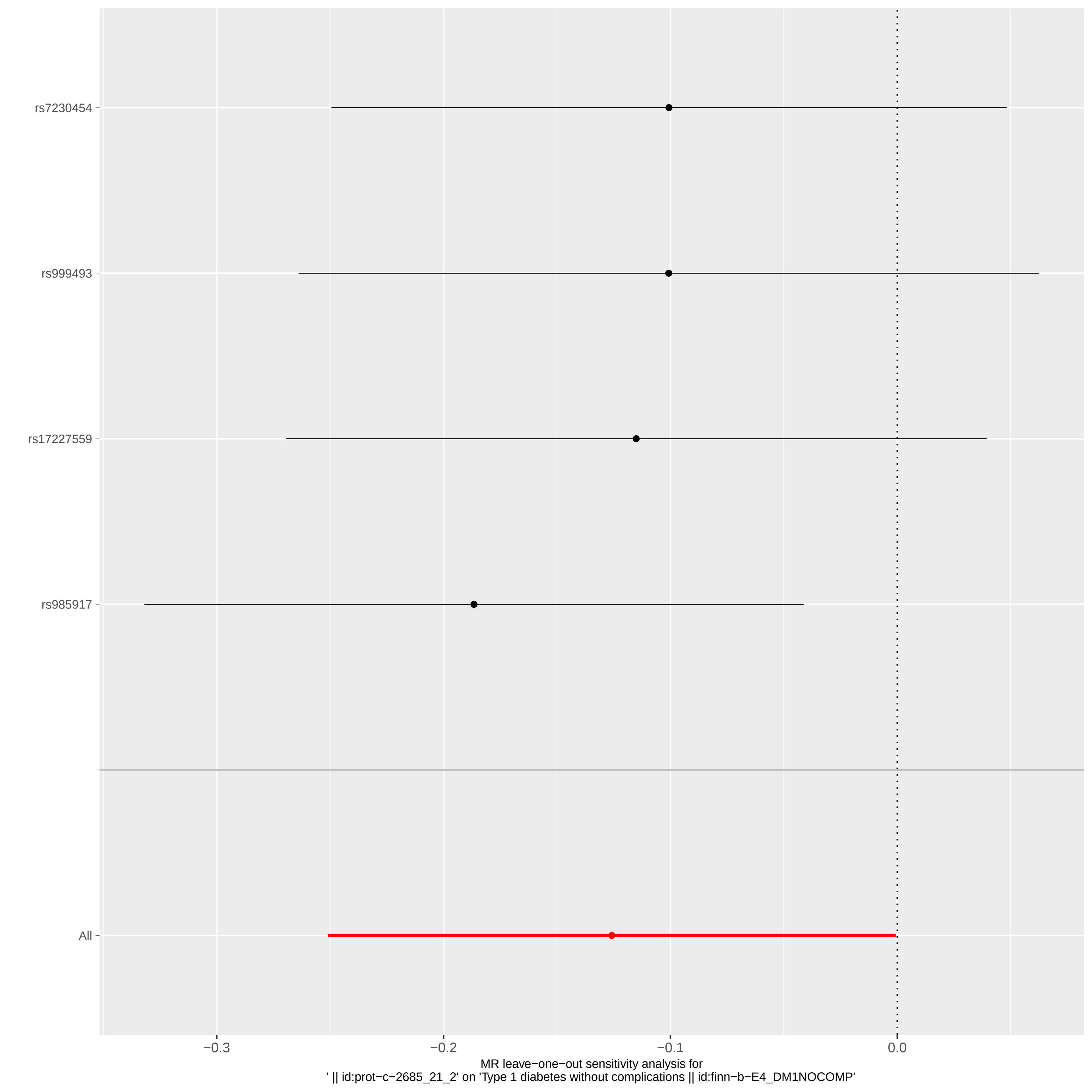
rs7230454
rs999493
rs17227559
rs985917
All
−0.3
−0.2
−0.1
0.0
MR leave−one−out sensitivity analysis for
' || id:prot−c−2685_21_2' on 'Type 1 diabetes without complications || id:finn−b−E4_DM1NOCOMP'

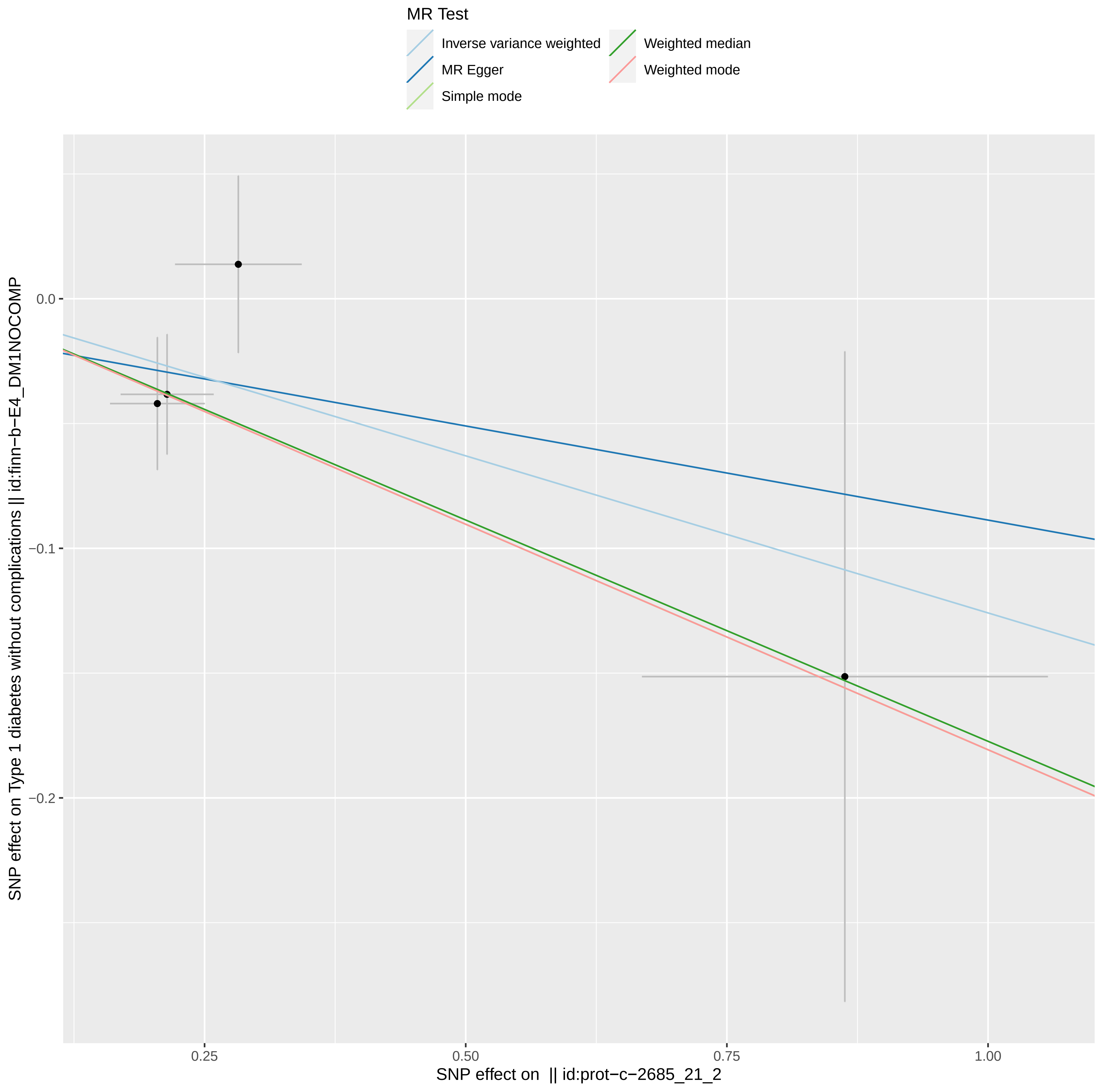
MR Test
Inverse variance weighted
MR Egger
Simple mode
Weighted median
Weighted mode
0.0
−0.1
−0.2
0.25
0.50
0.75
1.00
SNP effect on Type 1 diabetes without complications || id:finn−b−E4_DM1NOCOMP
SNP effect on || id:prot−c−2685_21_2

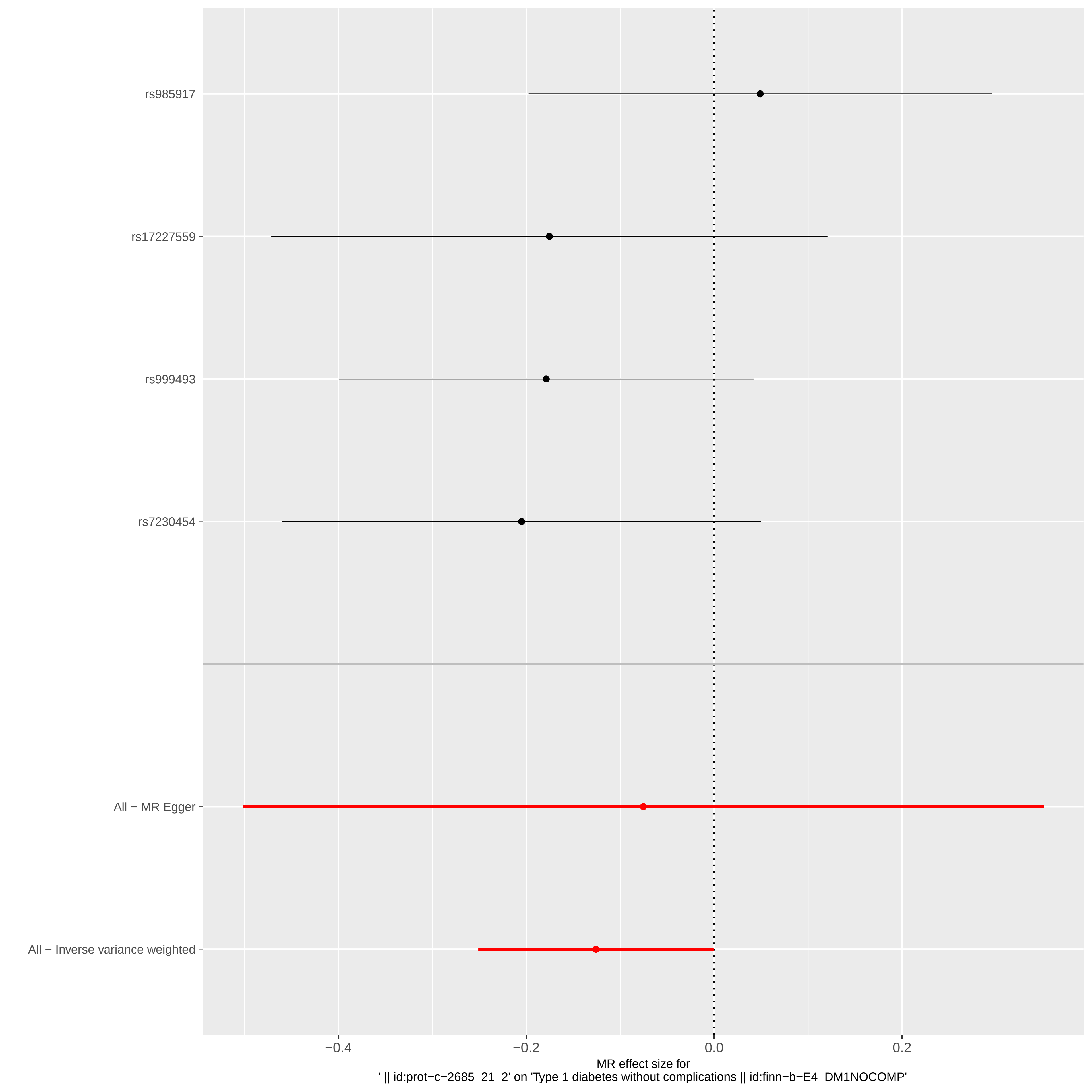

rs985917
rs17227559
rs999493
rs7230454
All − MR Egger
All − Inverse variance weighted
−0.4
−0.2
0.0
0.2
MR effect size for
' || id:prot−c−2685_21_2' on 'Type 1 diabetes without complications || id:finn−b−E4_DM1NOCOMP'

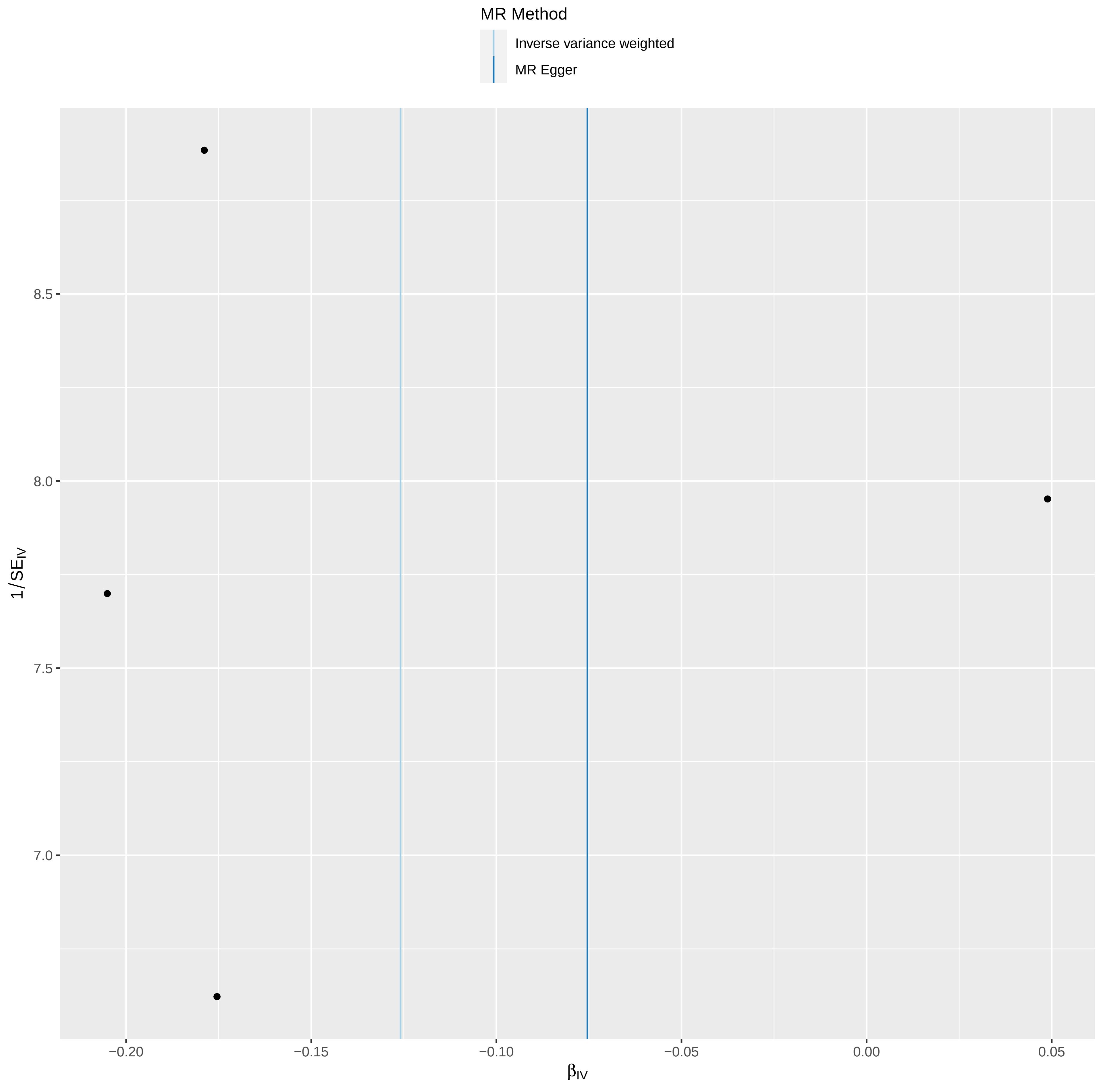

MR Method
Inverse variance weighted
MR Egger
8.5
8.0
7.5
7.0
1/SE_IV
−0.20
−0.15
−0.10
−0.05
0.00
0.05
β_IV

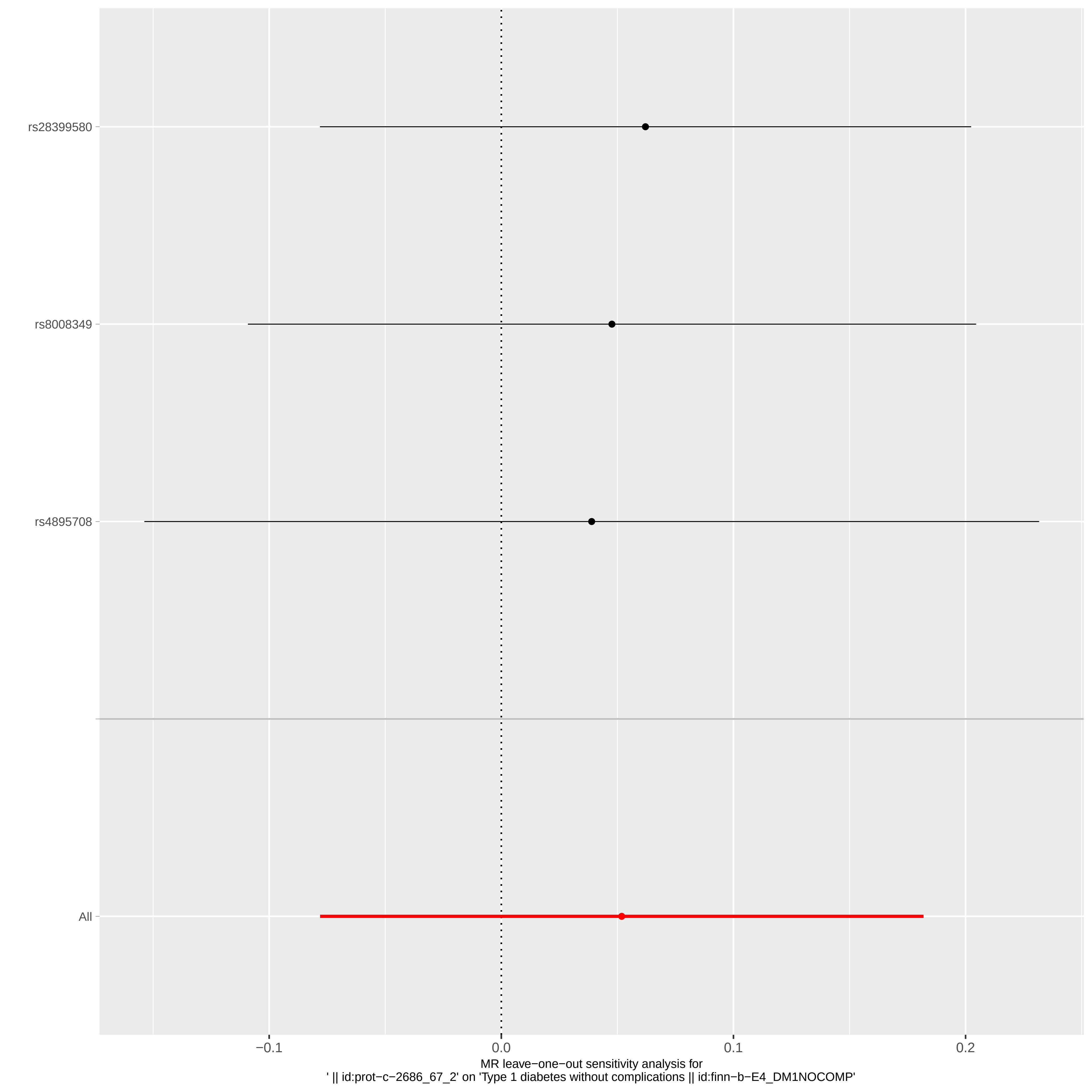
rs28399580
rs8008349
rs4895708
All
−0.1
0.0
0.1
0.2
MR leave−one−out sensitivity analysis for
' || id:prot−c−2686_67_2' on 'Type 1 diabetes without complications || id:finn−b−E4_DM1NOCOMP'

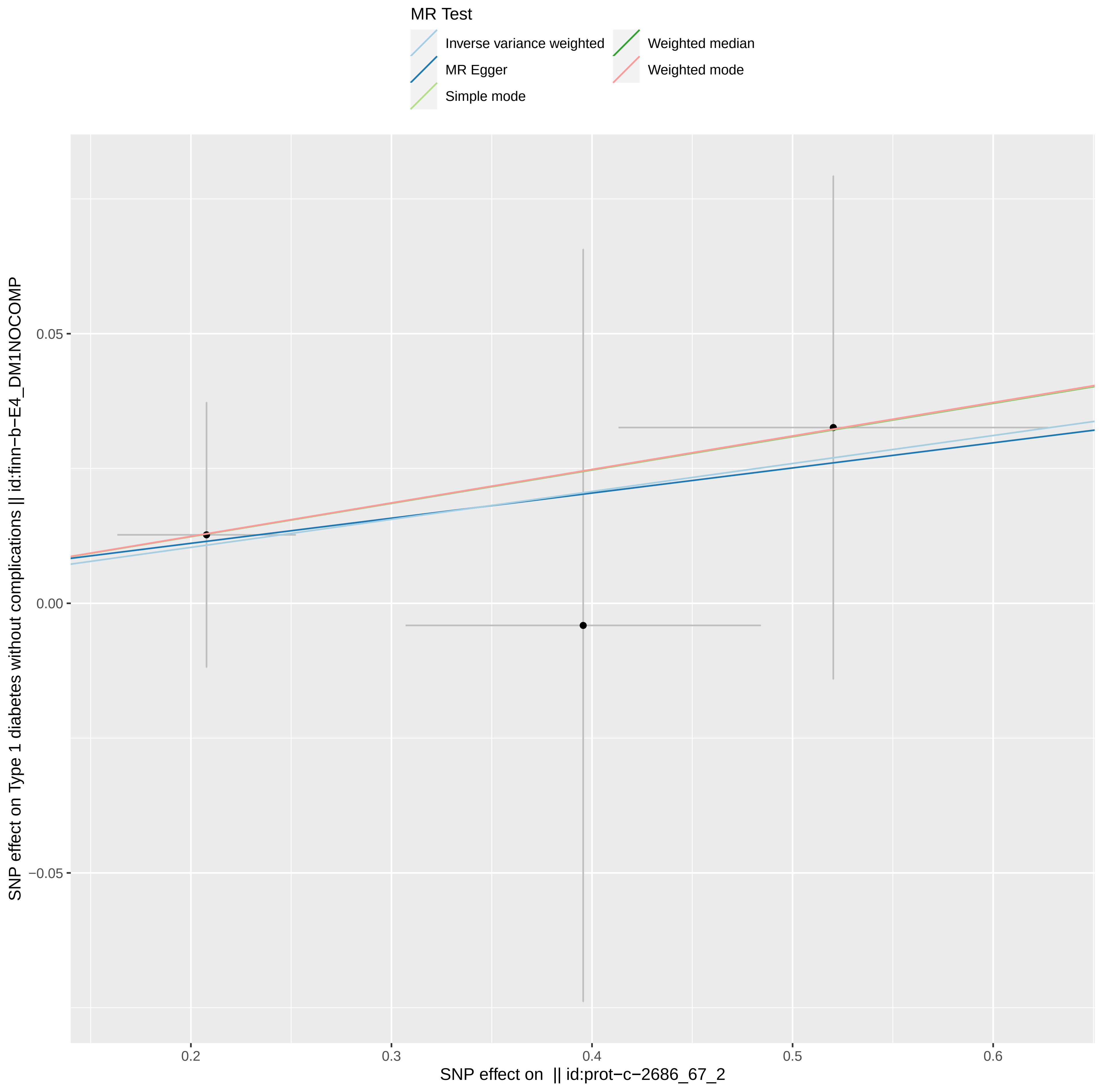
MR Test
Inverse variance weighted
MR Egger
Simple mode
Weighted median
Weighted mode
SNP effect on Type 1 diabetes without complications || id:finn−b−E4_DM1NOCOMP
0.05
0.00
−0.05
0.2
0.3
0.4
0.5
0.6
SNP effect on || id:prot−c−2686_67_2

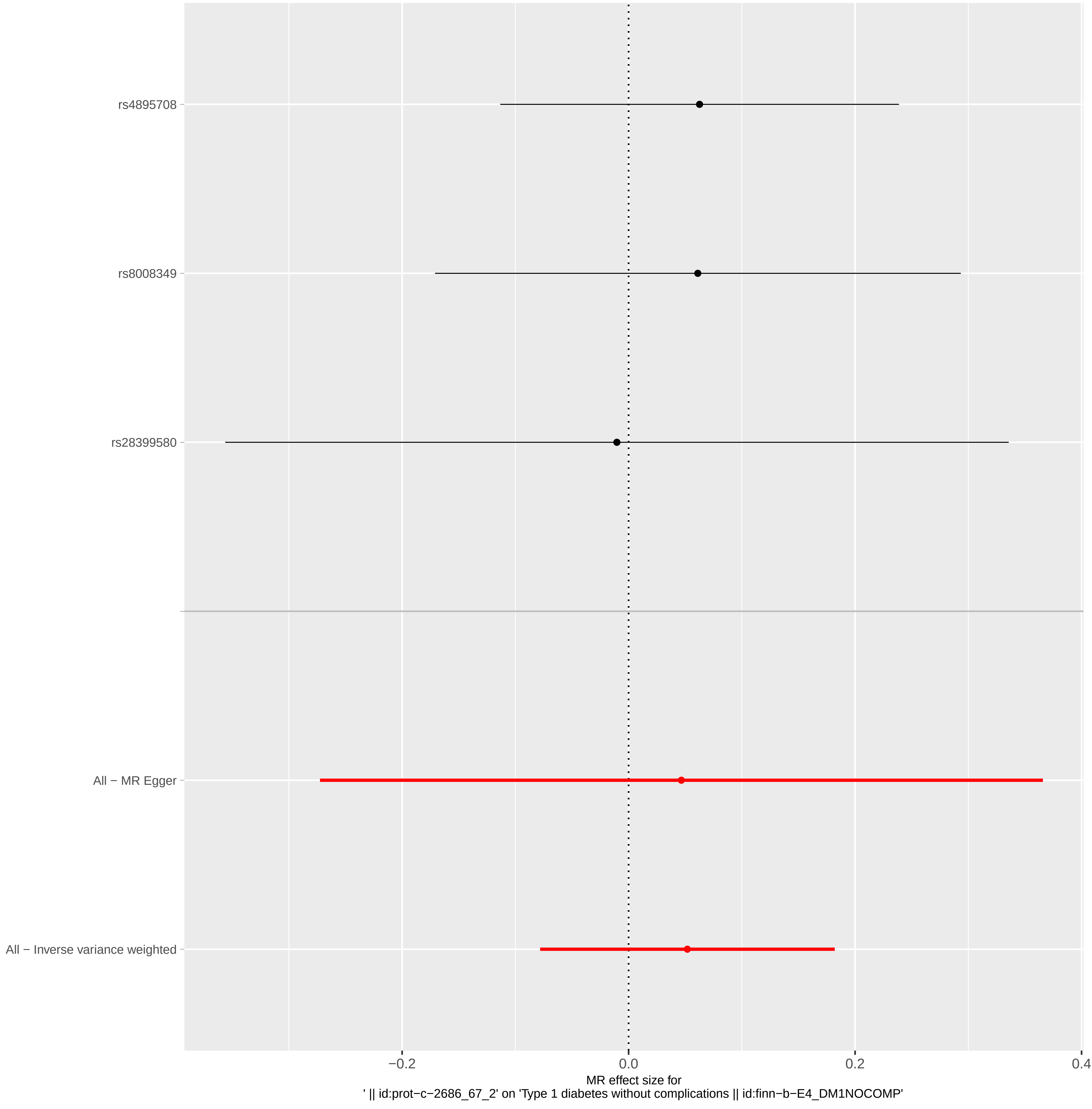

rs4895708
rs8008349
rs28399580
All − MR Egger
All − Inverse variance weighted
−0.2
0.0
0.2
0.4
MR effect size for
' || id:prot−c−2686_67_2' on 'Type 1 diabetes without complications || id:finn−b−E4_DM1NOCOMP'

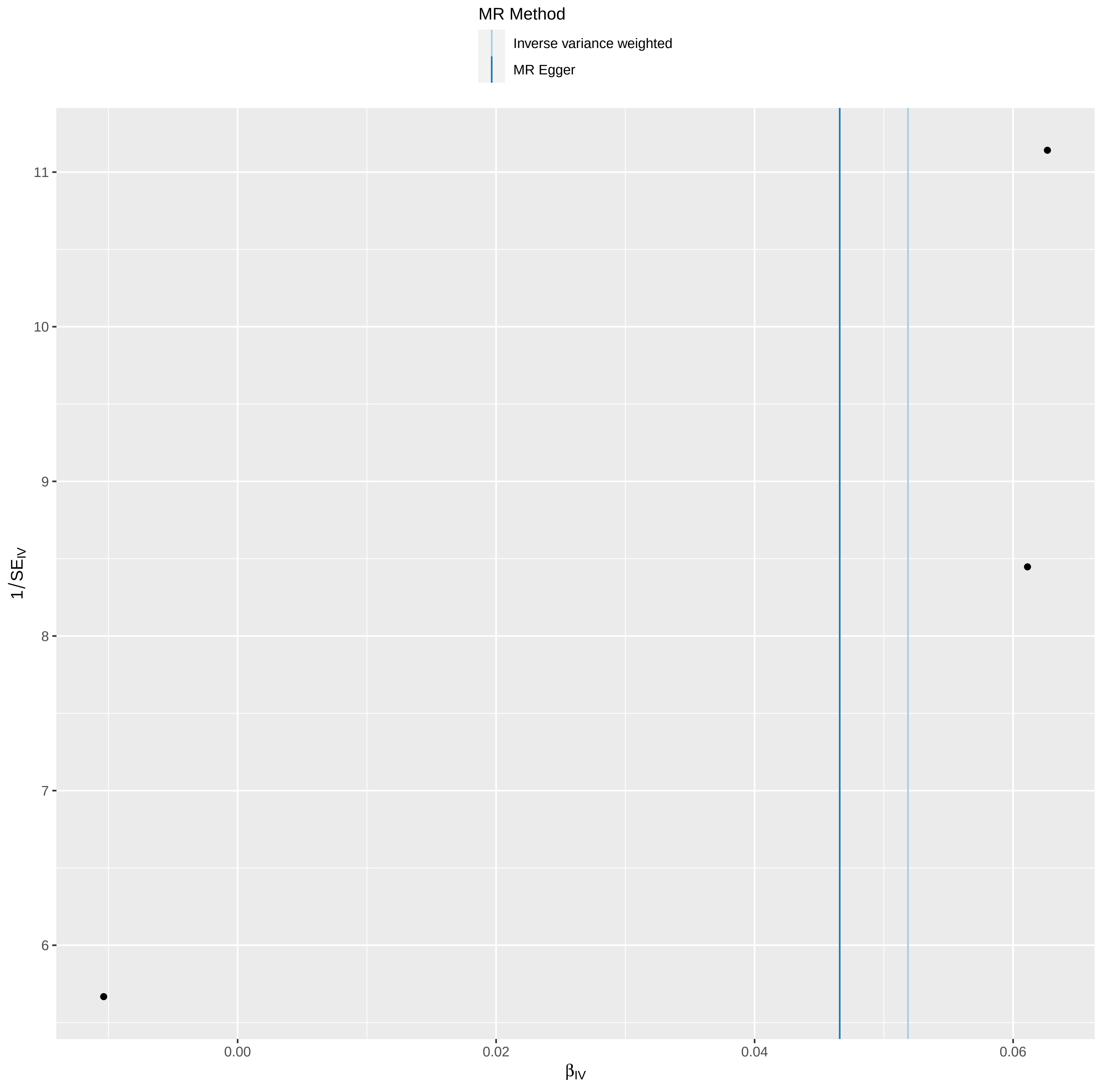

MR Method
Inverse variance weighted
MR Egger
11
10
9
8
7
6
1/SE$_{IV}$
0.00
0.02
0.04
0.06
β$_{IV}$

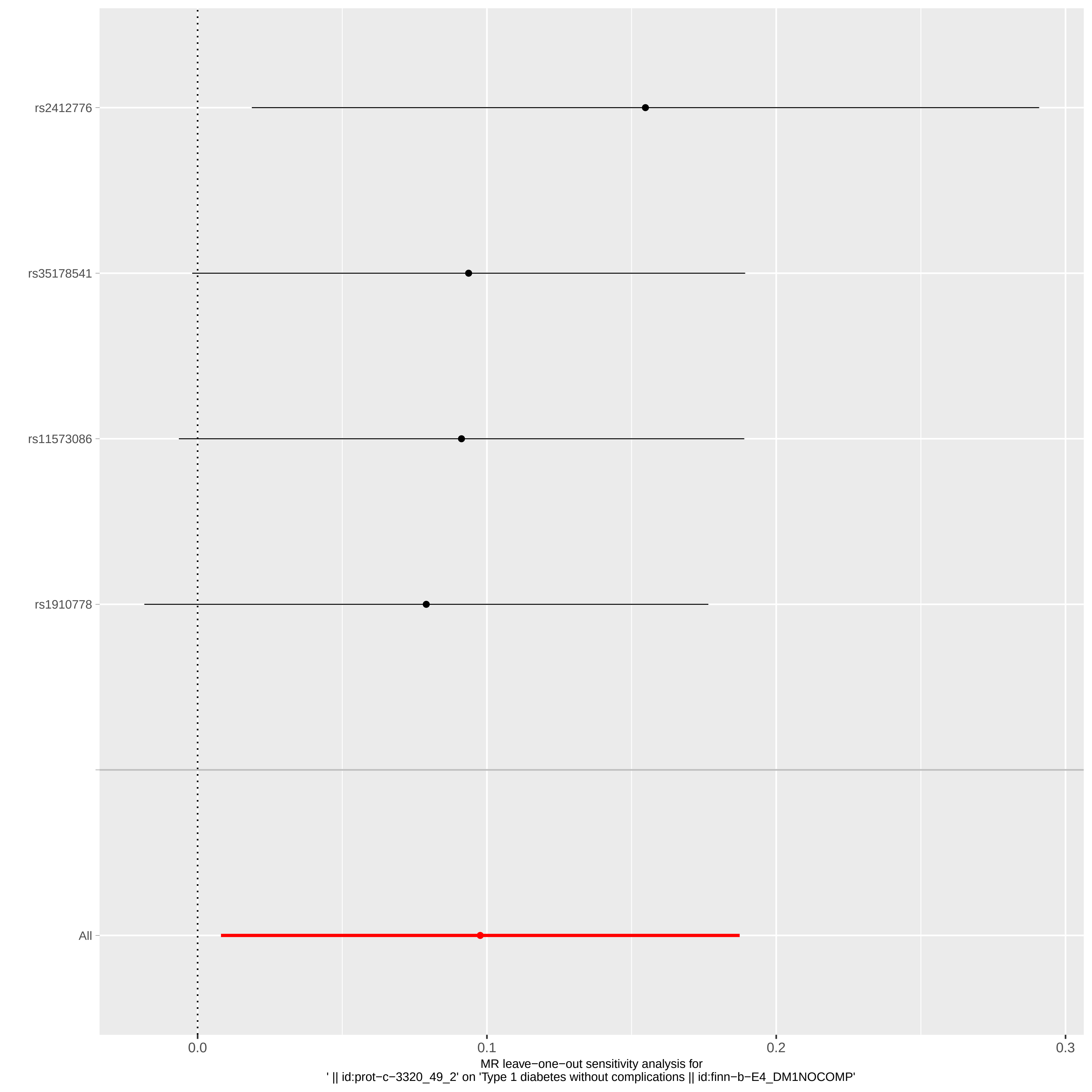

rs2412776
rs35178541
rs11573086
rs1910778
All
0.0
0.1
0.2
0.3
MR leave−one−out sensitivity analysis for
' || id:prot−c−3320_49_2' on 'Type 1 diabetes without complications || id:finn−b−E4_DM1NOCOMP'

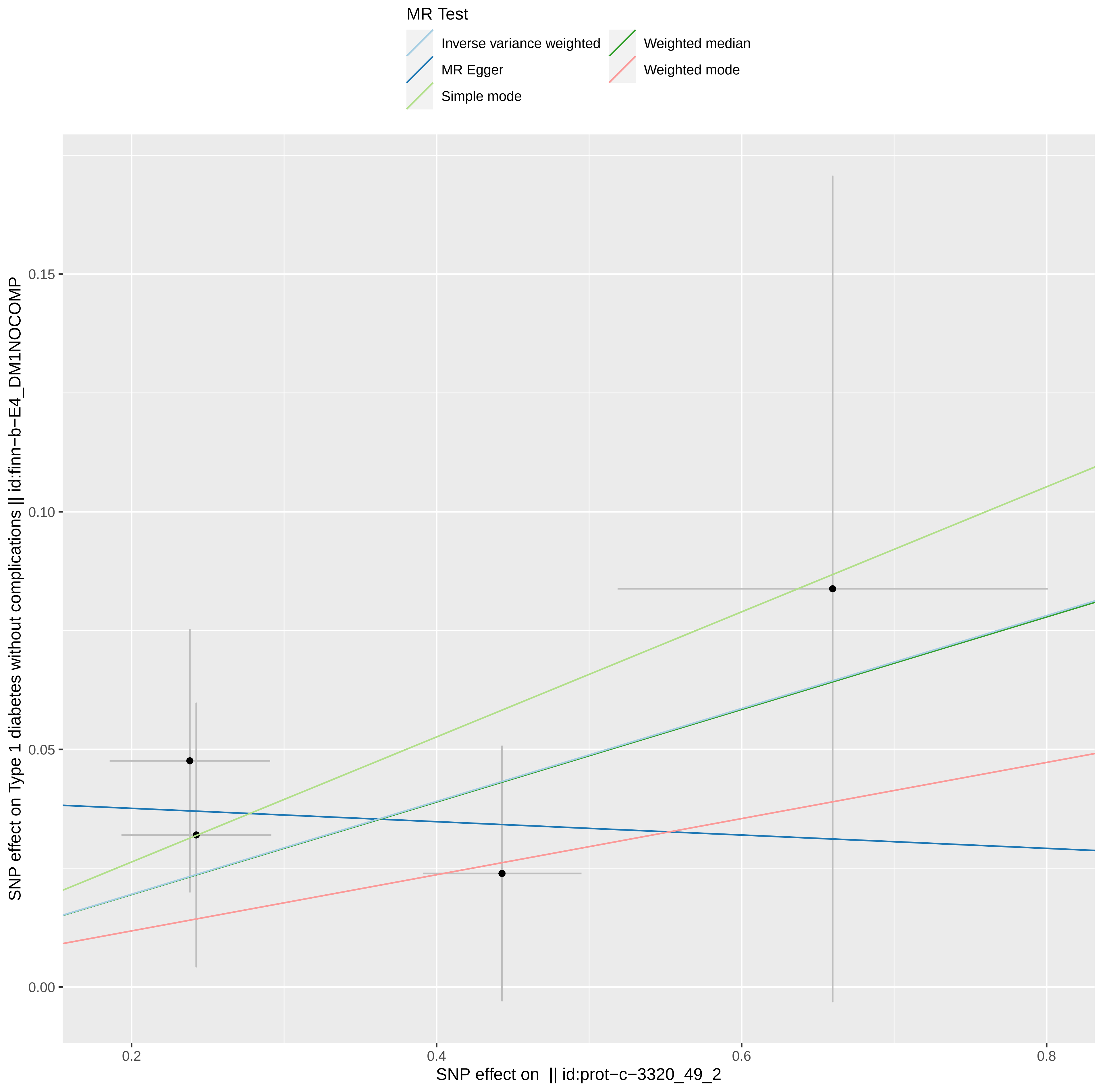

MR Test
Inverse variance weighted
MR Egger
Simple mode
Weighted median
Weighted mode
0.15
0.10
0.05
0.00
0.2
0.4
0.6
0.8
SNP effect on Type 1 diabetes without complications || id:finn−b−E4_DM1NOCOMP
SNP effect on || id:prot−c−3320_49_2

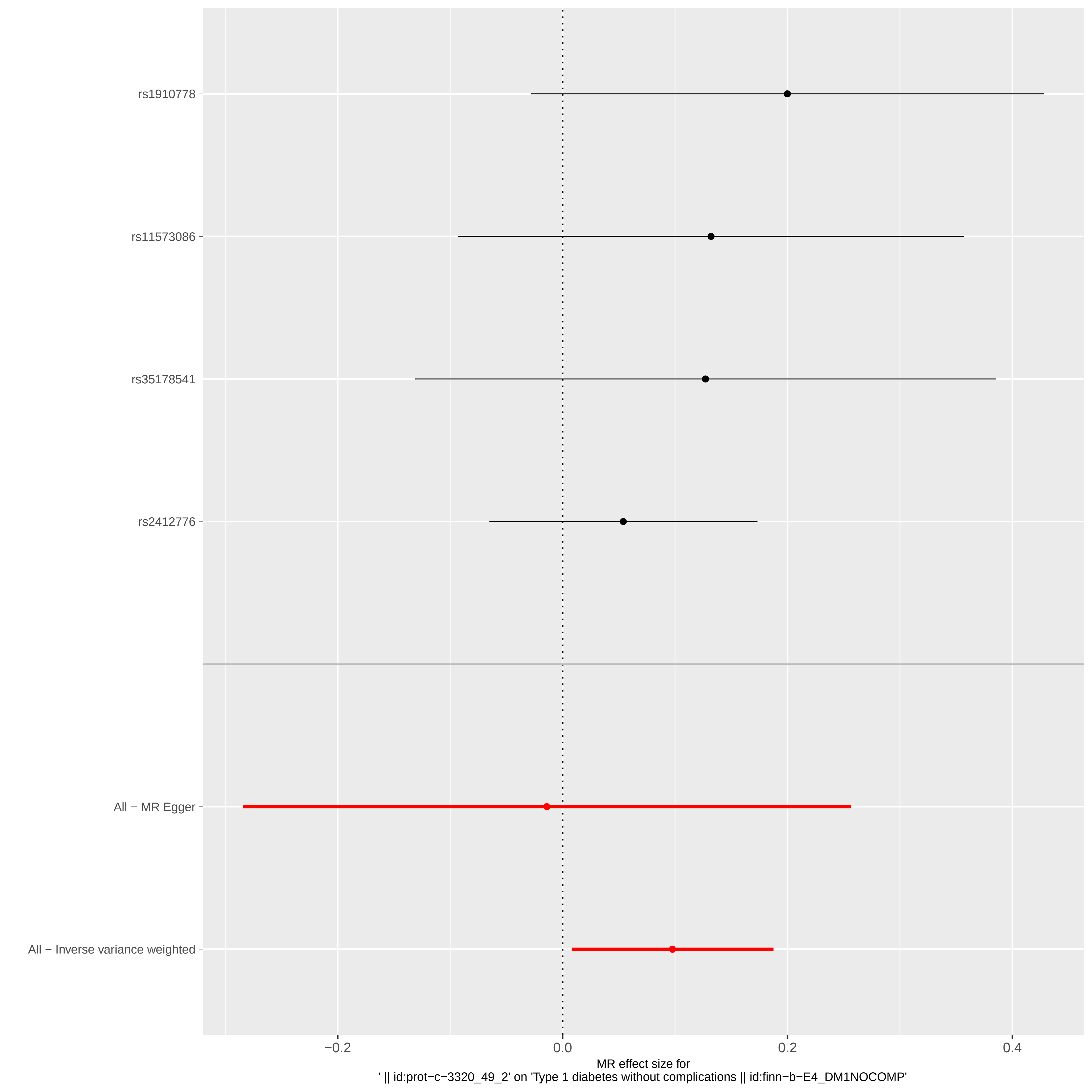
rs1910778
rs11573086
rs35178541
rs2412776
All − MR Egger
All − Inverse variance weighted
−0.2
0.0
0.2
0.4
MR effect size for
' || id:prot−c−3320_49_2' on 'Type 1 diabetes without complications || id:finn−b−E4_DM1NOCOMP'

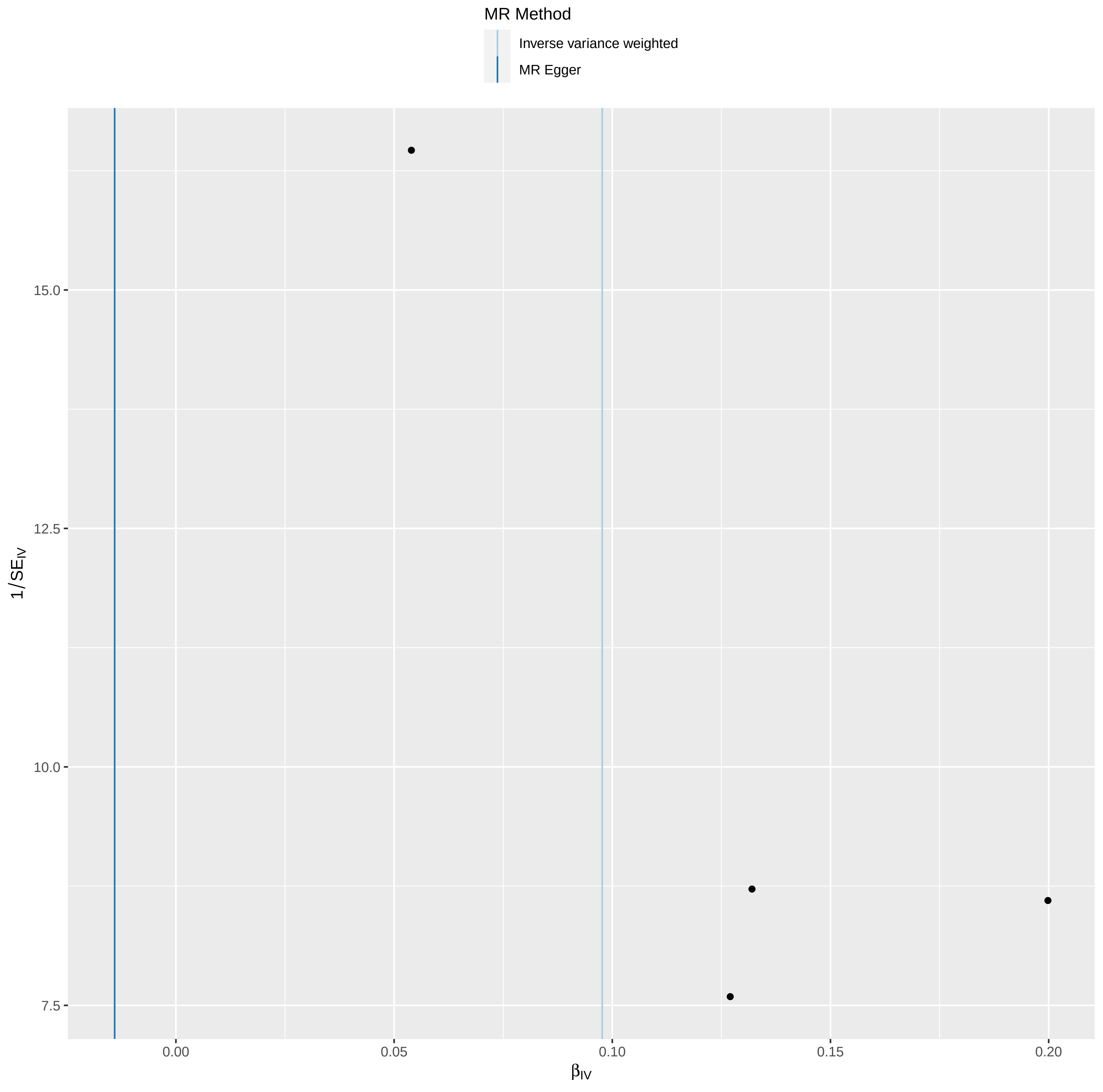

MR Method
Inverse variance weighted
MR Egger
15.0
12.5
10.0
7.5
1/SE_IV
0.00
0.05
0.10
0.15
0.20
β_IV

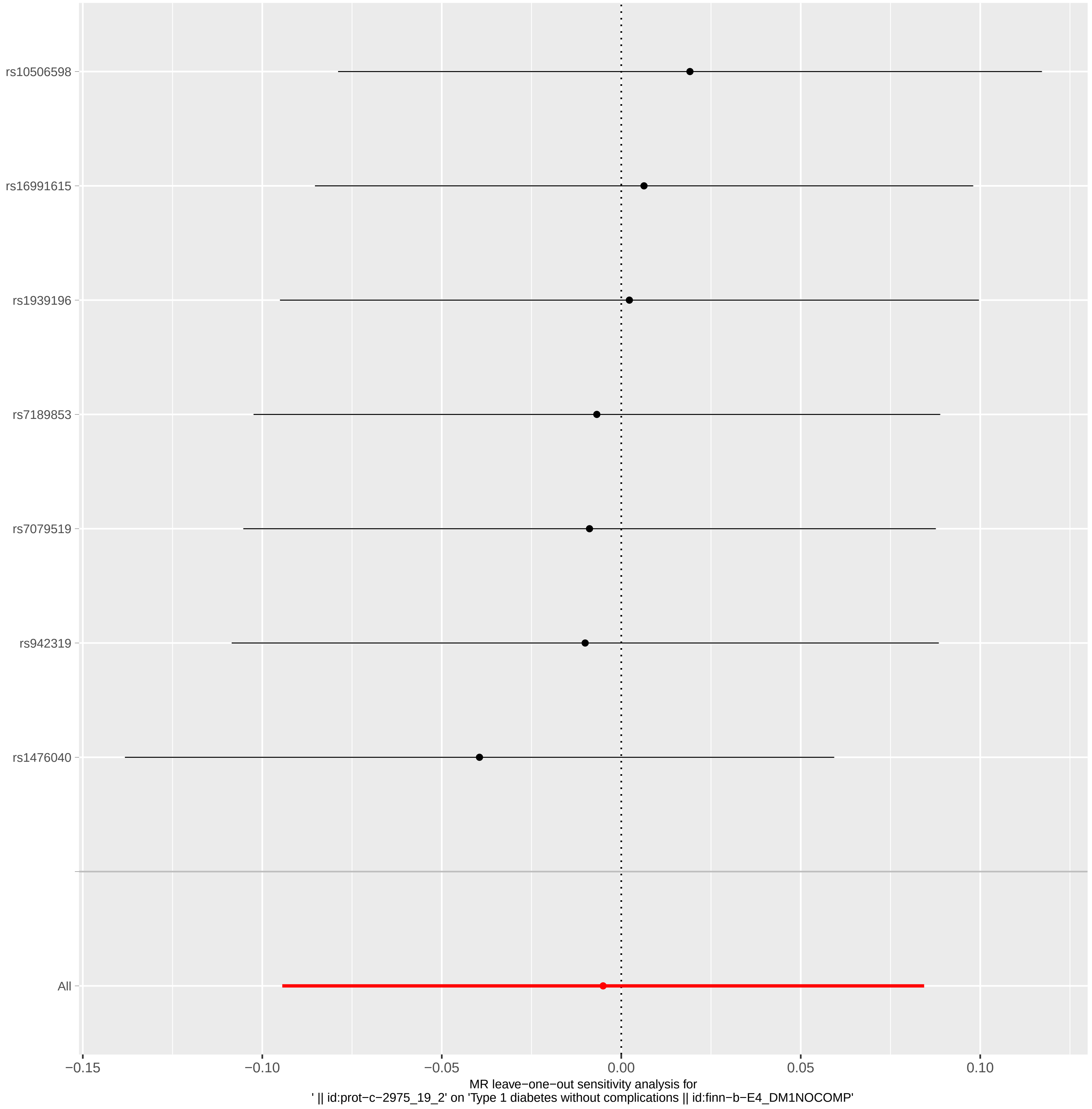

rs10506598
rs16991615
rs1939196
rs7189853
rs7079519
rs942319
rs1476040
All
−0.15
−0.10
−0.05
0.00
0.05
0.10
MR leave−one−out sensitivity analysis for
' || id:prot−c−2975_19_2' on 'Type 1 diabetes without complications || id:finn−b−E4_DM1NOCOMP'

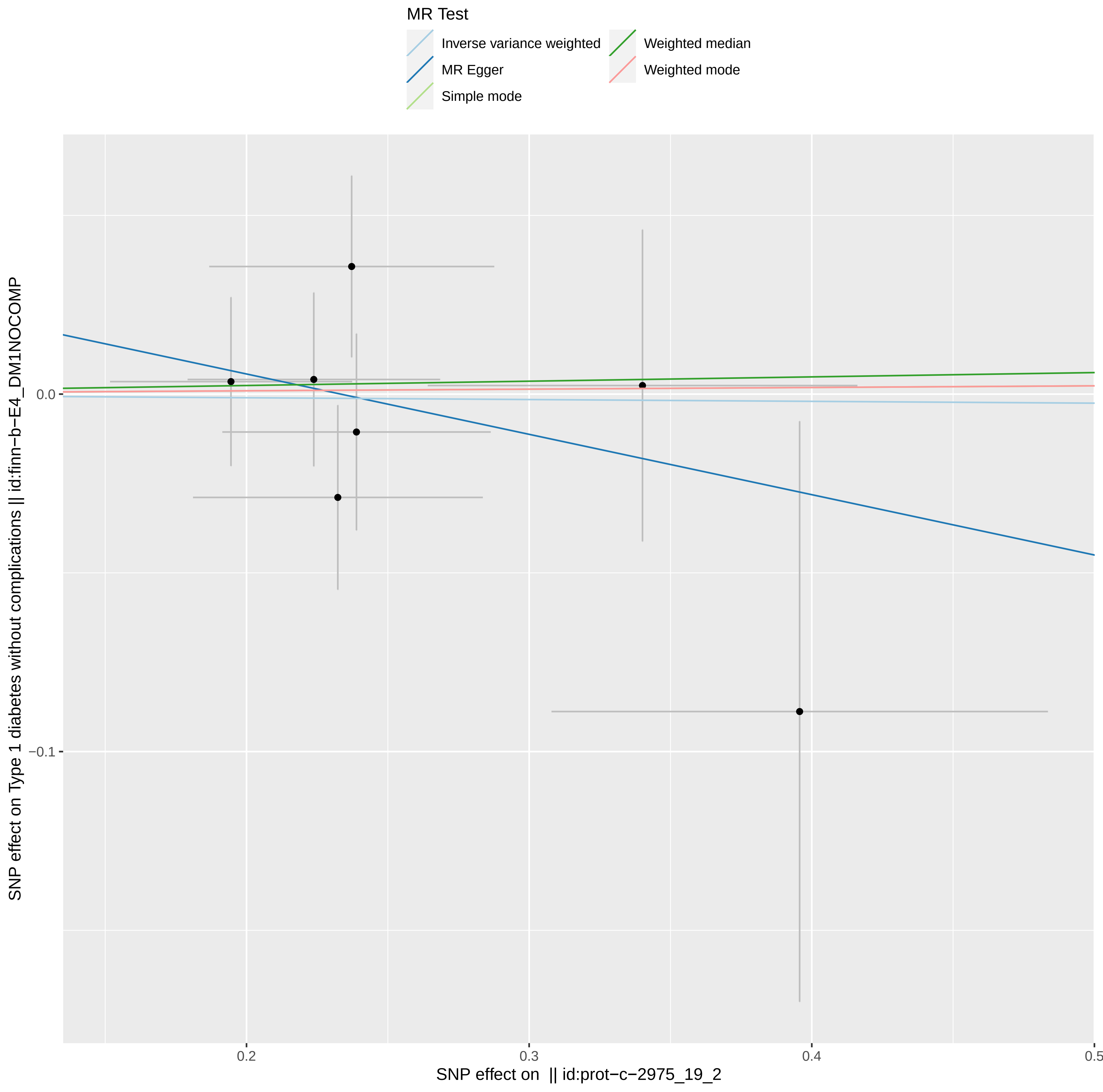

MR Test
Inverse variance weighted
MR Egger
Simple mode
Weighted median
Weighted mode
0.0
−0.1
0.2
0.3
0.4
0.5
SNP effect on Type 1 diabetes without complications || id:finn-b-E4_DM1NOCOMP
SNP effect on || id:prot−c−2975_19_2

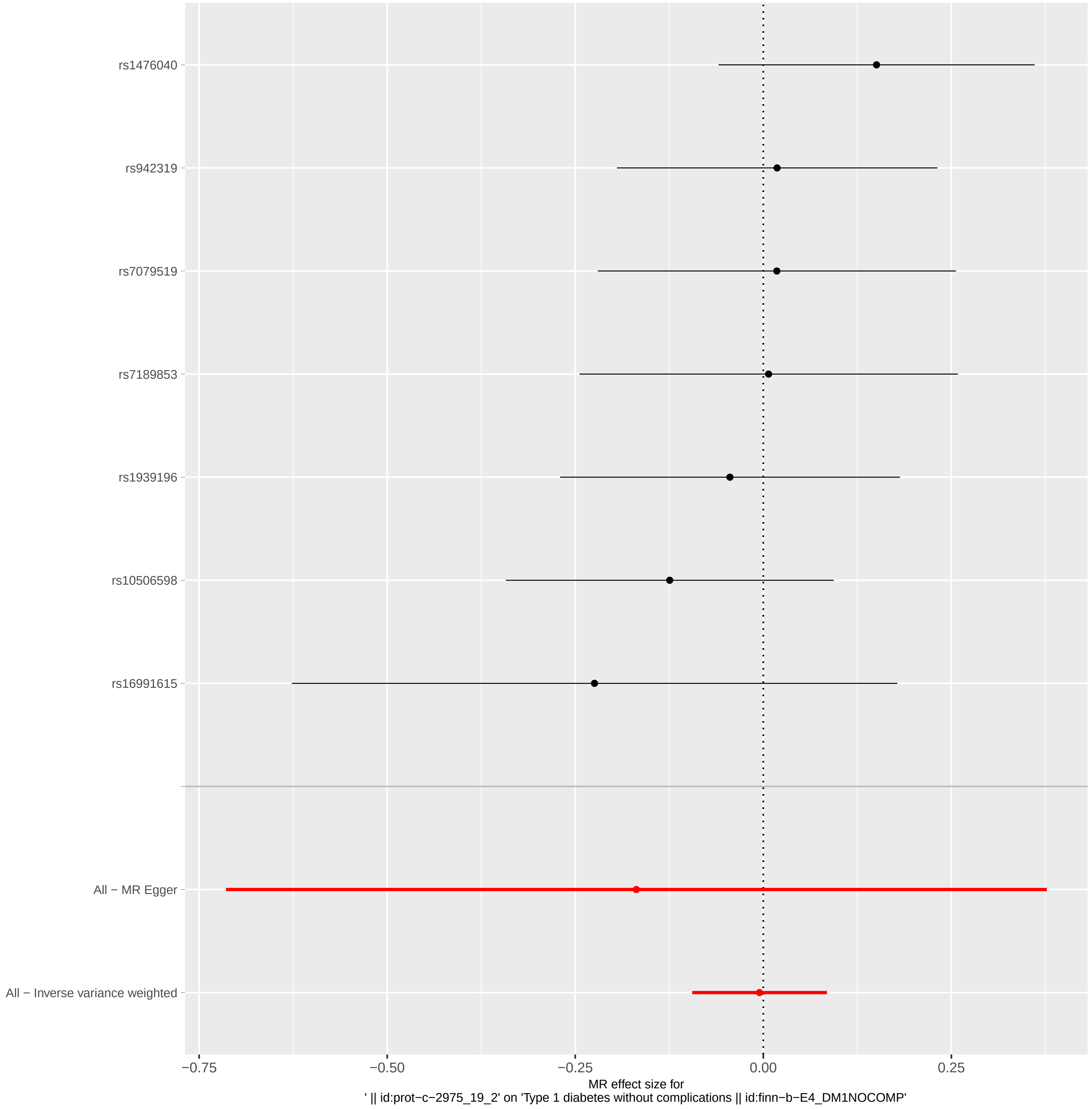
rs1476040
rs942319
rs7079519
rs7189853
rs1939196
rs10506598
rs16991615
All − MR Egger
All − Inverse variance weighted
−0.75
−0.50
−0.25
0.00
0.25
MR effect size for
' || id:prot−c−2975_19_2' on 'Type 1 diabetes without complications || id:finn−b−E4_DM1NOCOMP'

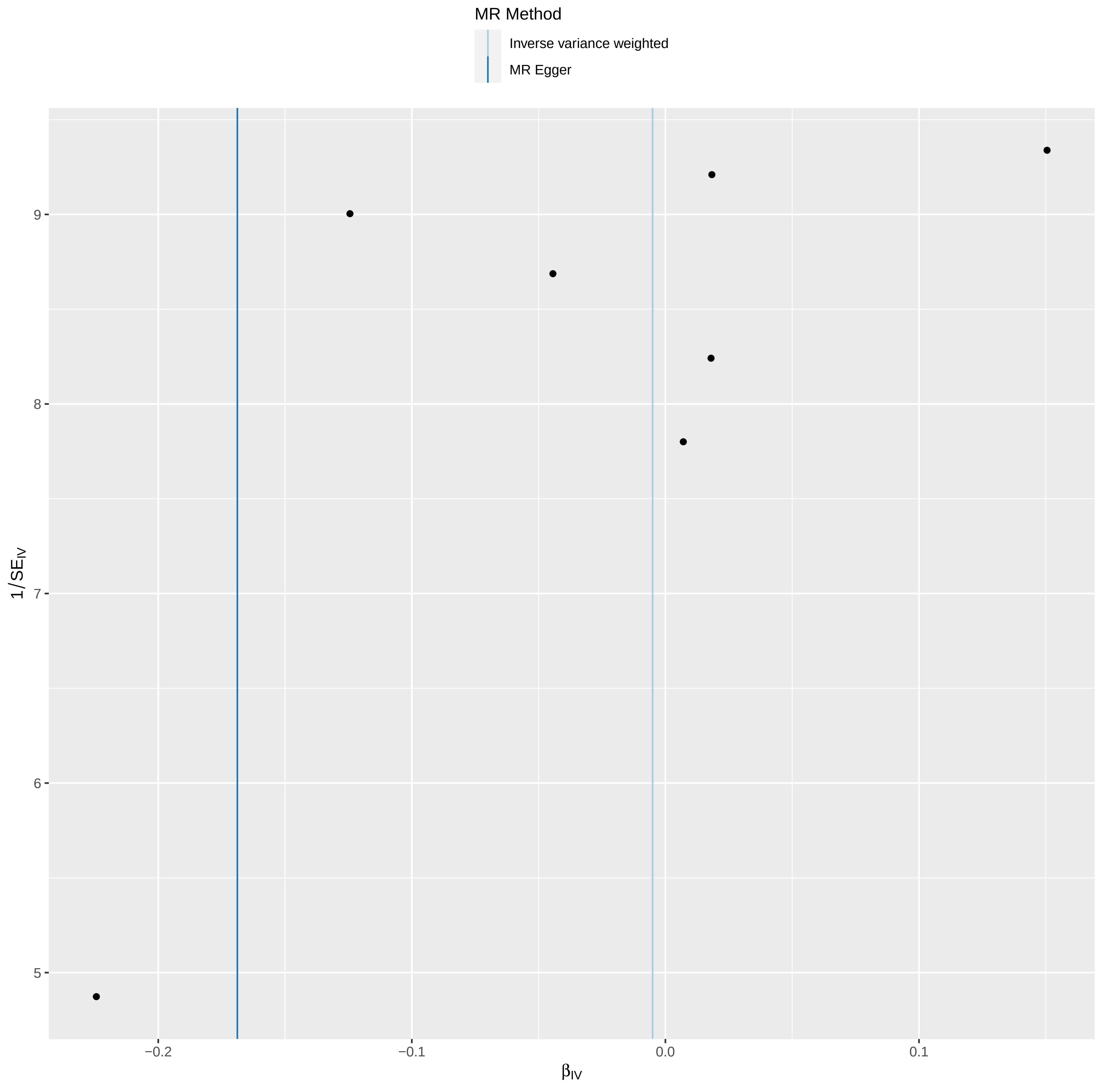
MR Method
Inverse variance weighted
MR Egger
1/SE_IV
β_IV
9
8
7
6
5
−0.2
−0.1
0.0
0.1

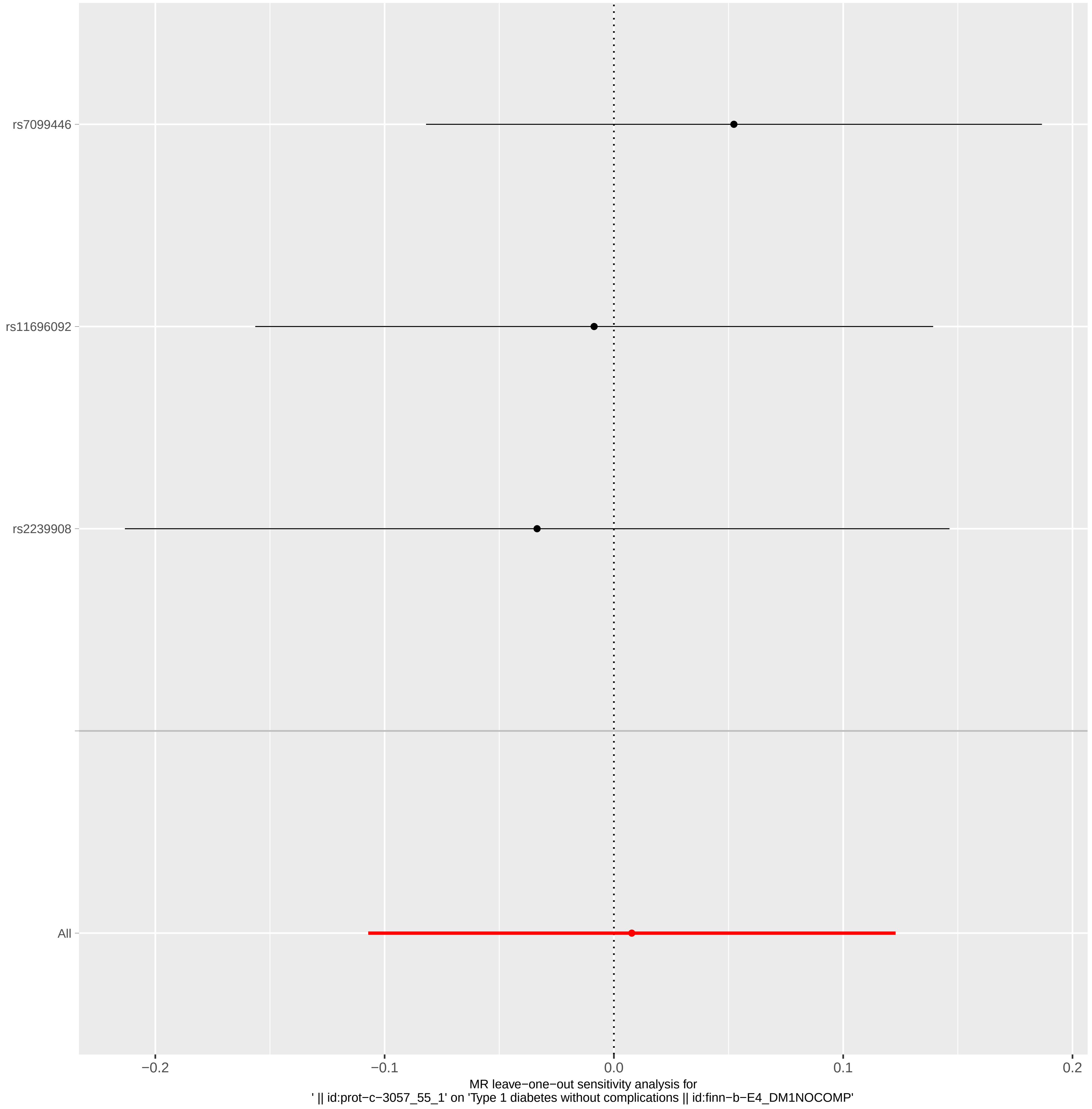
rs7099446
rs11696092
rs2239908
All
−0.2
−0.1
0.0
0.1
0.2
MR leave−one−out sensitivity analysis for
' || id:prot−c−3057_55_1' on 'Type 1 diabetes without complications || id:finn−b−E4_DM1NOCOMP'

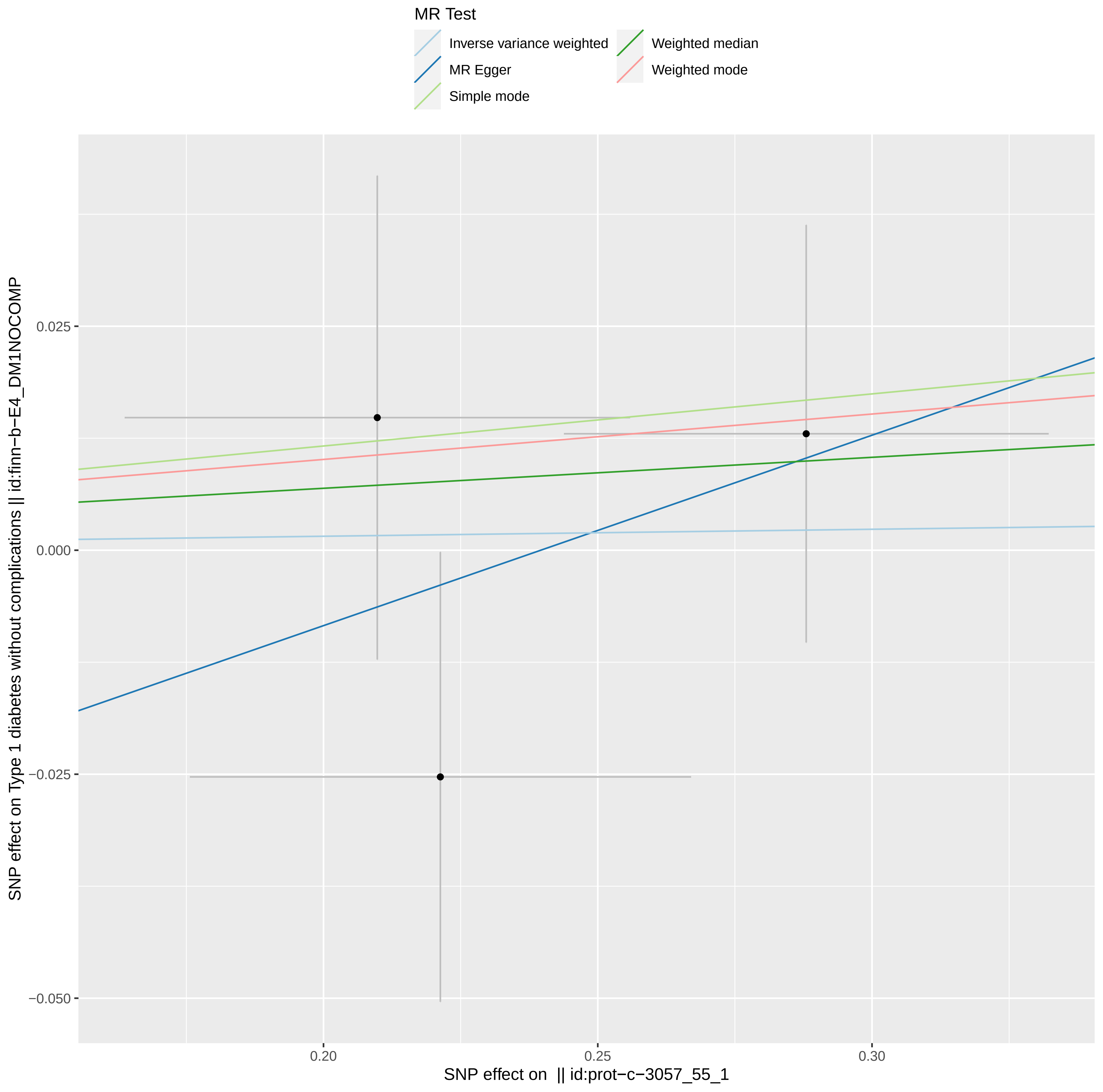
MR Test
Inverse variance weighted
MR Egger
Simple mode
Weighted median
Weighted mode
0.025
0.000
−0.025
−0.050
0.20
0.25
0.30
SNP effect on Type 1 diabetes without complications || id:finn−b−E4_DM1NOCOMP
SNP effect on || id:prot−c−3057_55_1

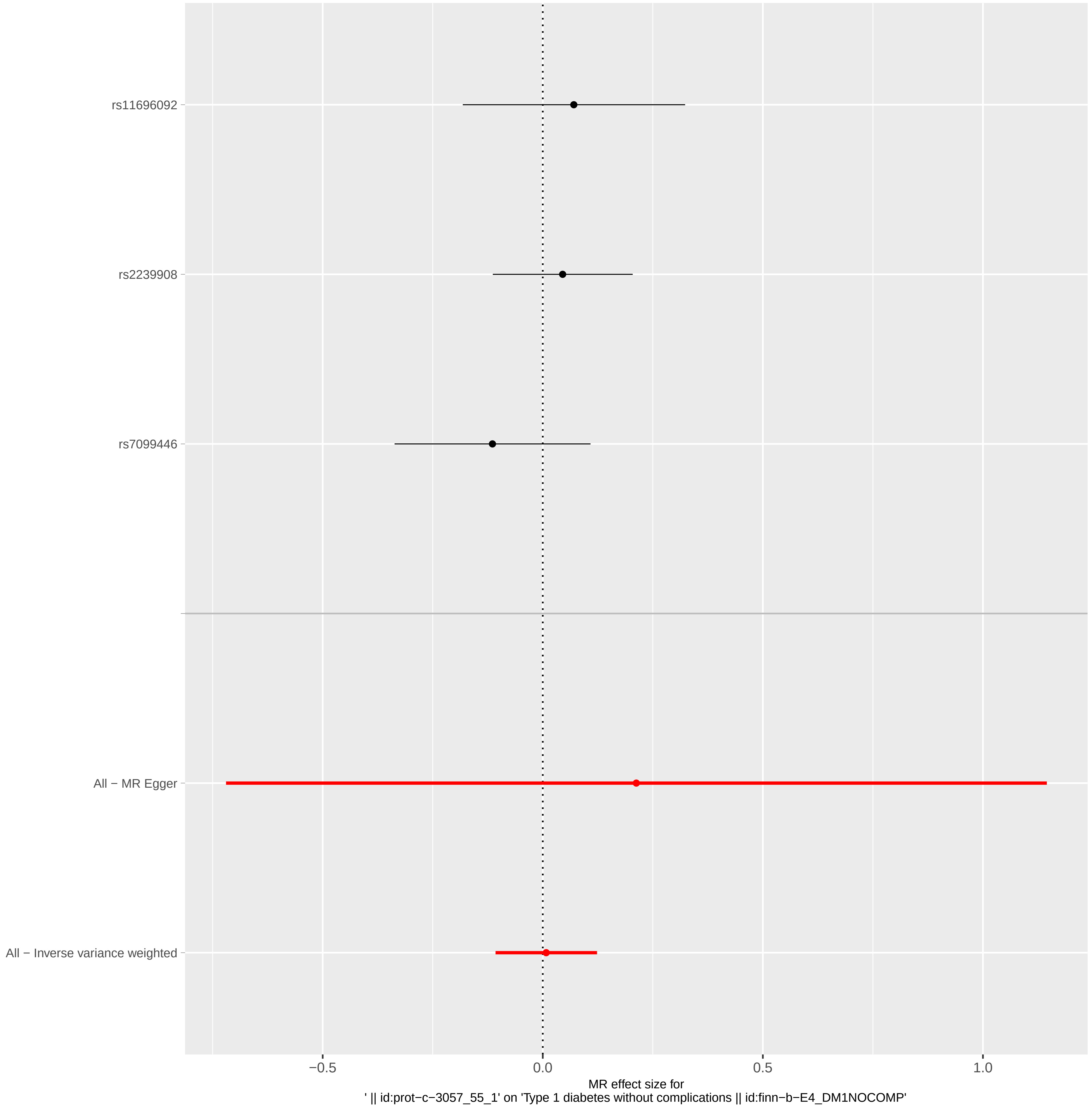
rs11696092
rs2239908
rs7099446
All − MR Egger
All − Inverse variance weighted
−0.5
0.0
0.5
1.0
MR effect size for
' || id:prot−c−3057_55_1' on 'Type 1 diabetes without complications || id:finn−b−E4_DM1NOCOMP'

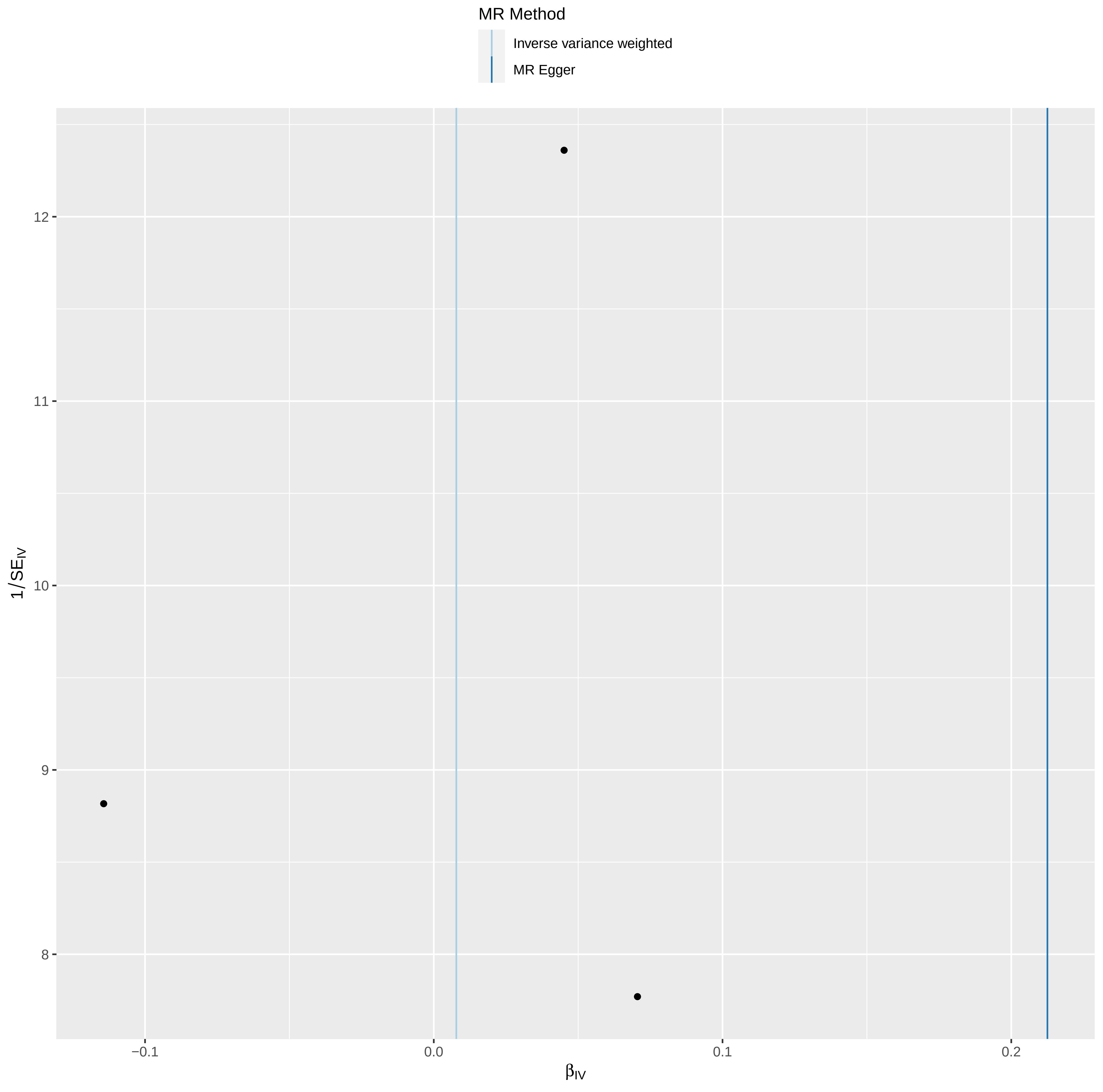

MR Method
Inverse variance weighted
MR Egger
12
11
10
9
8
$1/SE_{IV}$
−0.1
0.0
0.1
0.2
β_{IV}

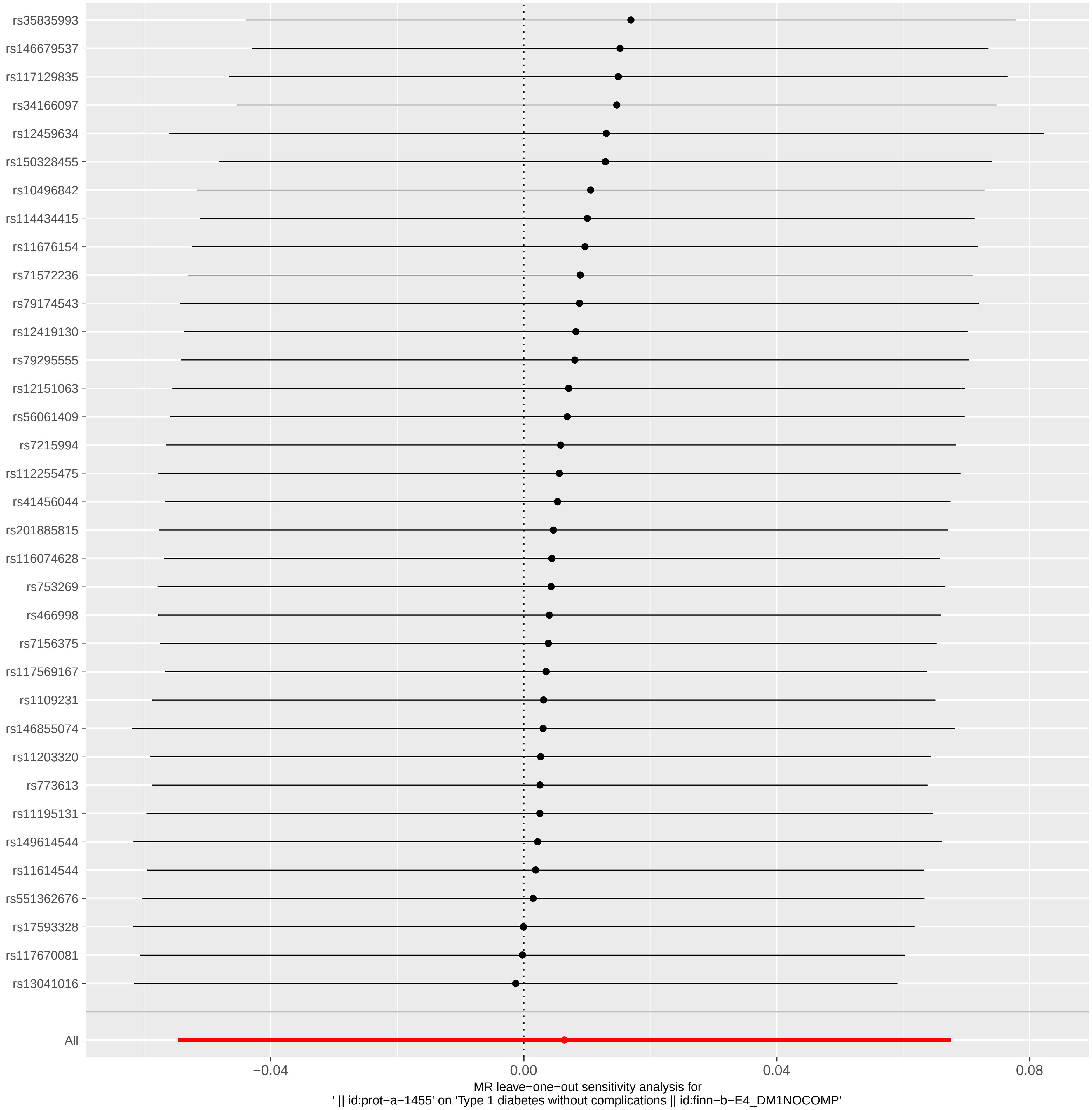
rs35835993
rs146679537
rs117129835
rs34166097
rs12459634
rs150328455
rs10496842
rs114434415
rs11676154
rs71572236
rs79174543
rs12419130
rs79295555
rs12151063
rs56061409
rs7215994
rs112255475
rs41456044
rs201885815
rs116074628
rs753269
rs466998
rs7156375
rs117569167
rs1109231
rs146855074
rs11203320
rs773613
rs11195131
rs149614544
rs11614544
rs551362676
rs17593328
rs117670081
rs13041016
All
−0.04
0.00
0.04
0.08
MR leave−one−out sensitivity analysis for
' || id:prot−a−1455' on 'Type 1 diabetes without complications || id:finn−b−E4_DM1NOCOMP'

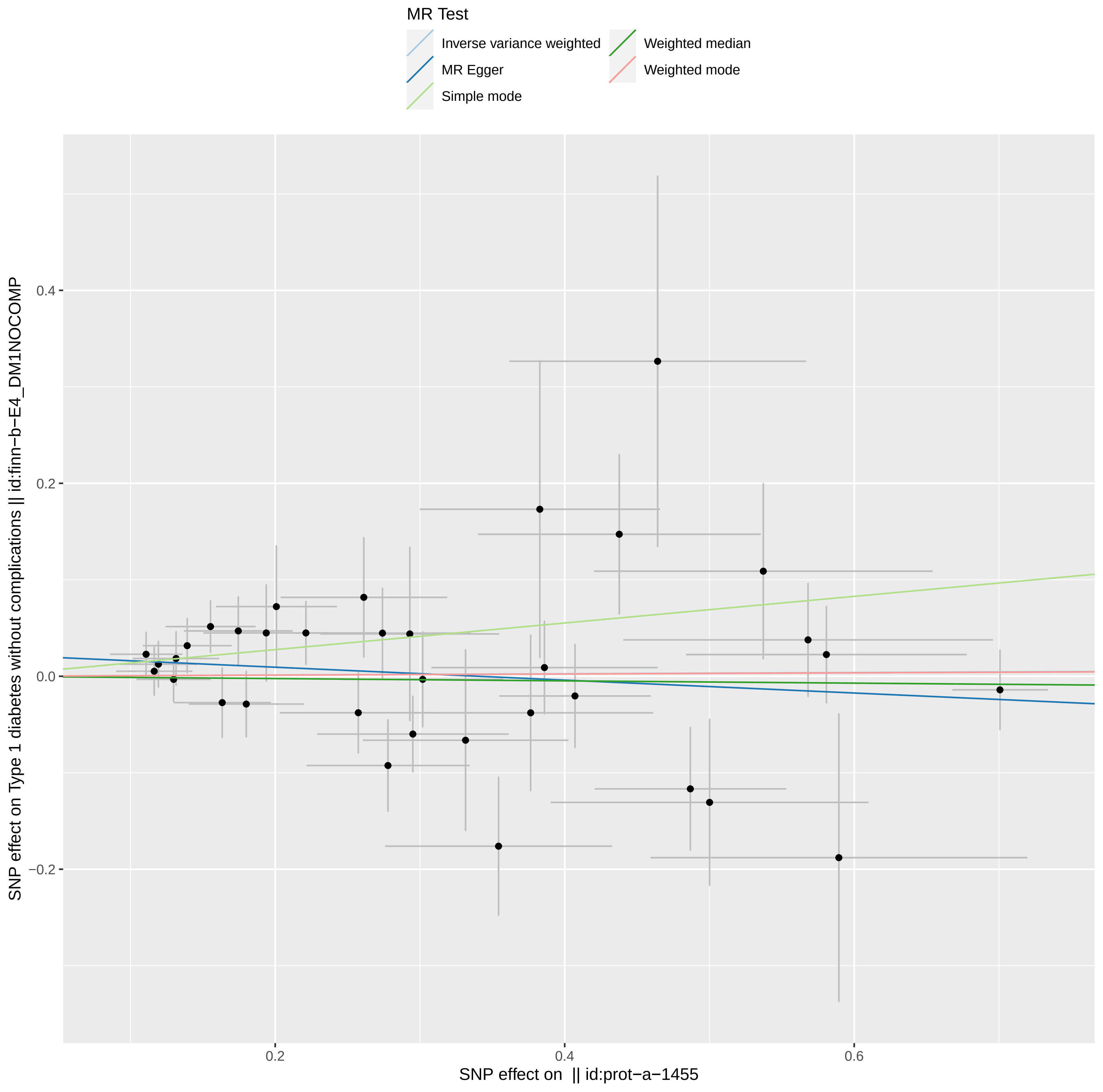

MR Test
Inverse variance weighted
MR Egger
Simple mode
Weighted median
Weighted mode
SNP effect on Type 1 diabetes without complications || id:finn−b−E4_DM1NOCOMP
0.4
0.2
0.0
−0.2
0.2
0.4
0.6
SNP effect on || id:prot−a−1455

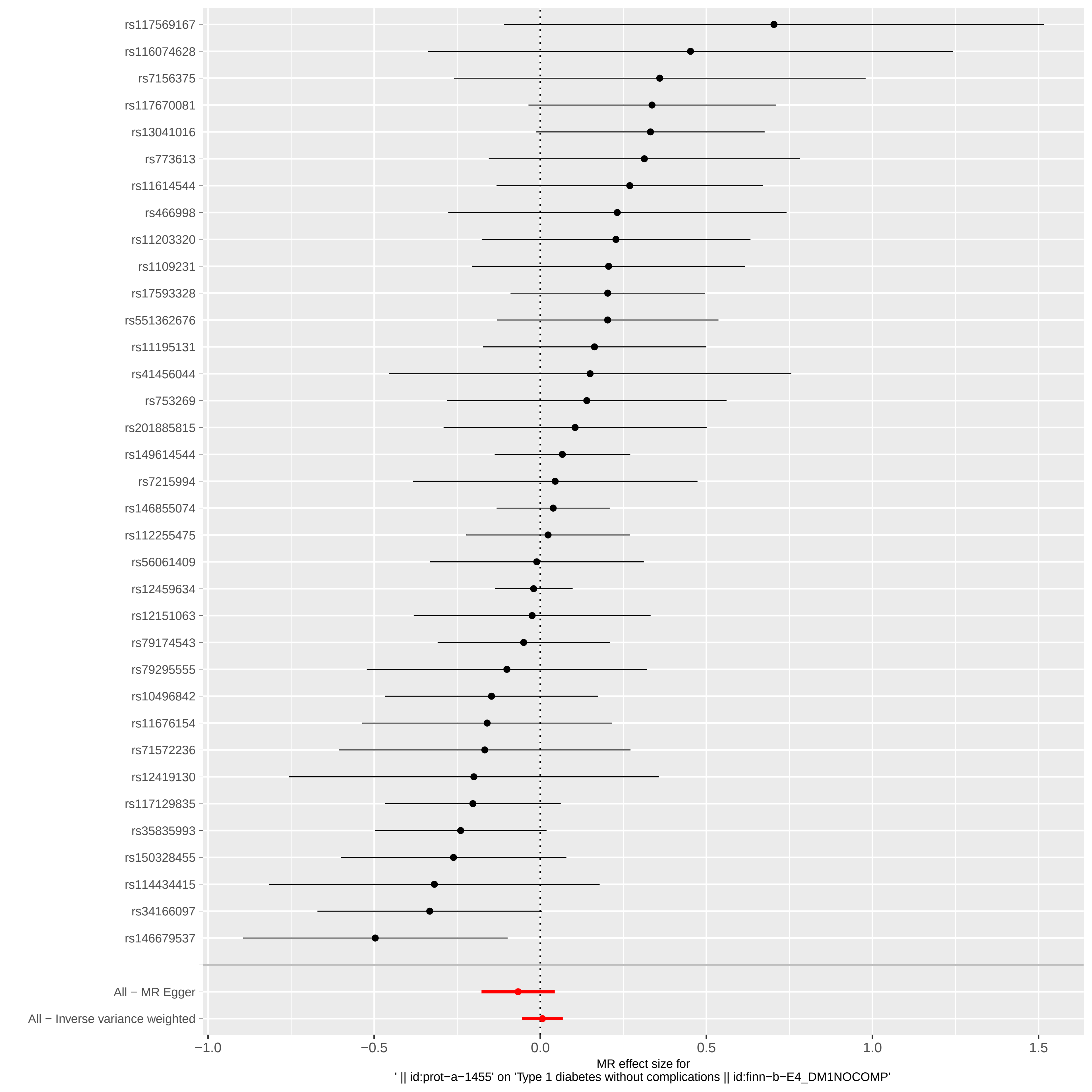
rs117569167
rs116074628
rs7156375
rs117670081
rs13041016
rs773613
rs11614544
rs466998
rs11203320
rs1109231
rs17593328
rs551362676
rs11195131
rs41456044
rs753269
rs201885815
rs149614544
rs7215994
rs146855074
rs112255475
rs56061409
rs12459634
rs12151063
rs79174543
rs79295555
rs10496842
rs11676154
rs71572236
rs12419130
rs117129835
rs35835993
rs150328455
rs114434415
rs34166097
rs146679537
All − MR Egger
All − Inverse variance weighted
−1.0
−0.5
0.0
0.5
1.0
1.5
MR effect size for
' || id:prot−a−1455' on 'Type 1 diabetes without complications || id:finn−b−E4_DM1NOCOMP'

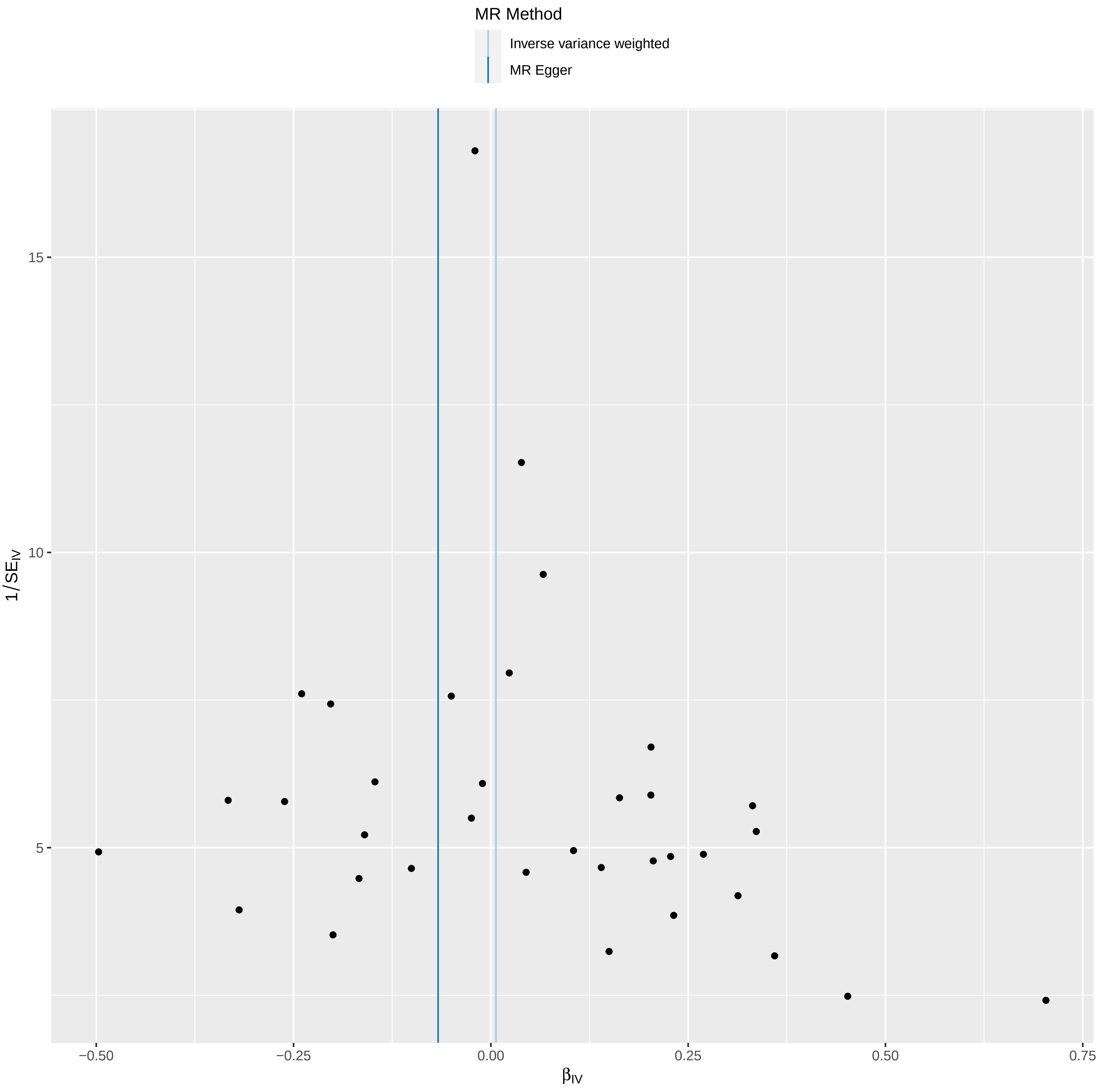
MR Method
Inverse variance weighted
MR Egger
1/SE$_{IV}$
15
10
5
−0.50
−0.25
0.00
0.25
0.50
0.75
β$_{IV}$

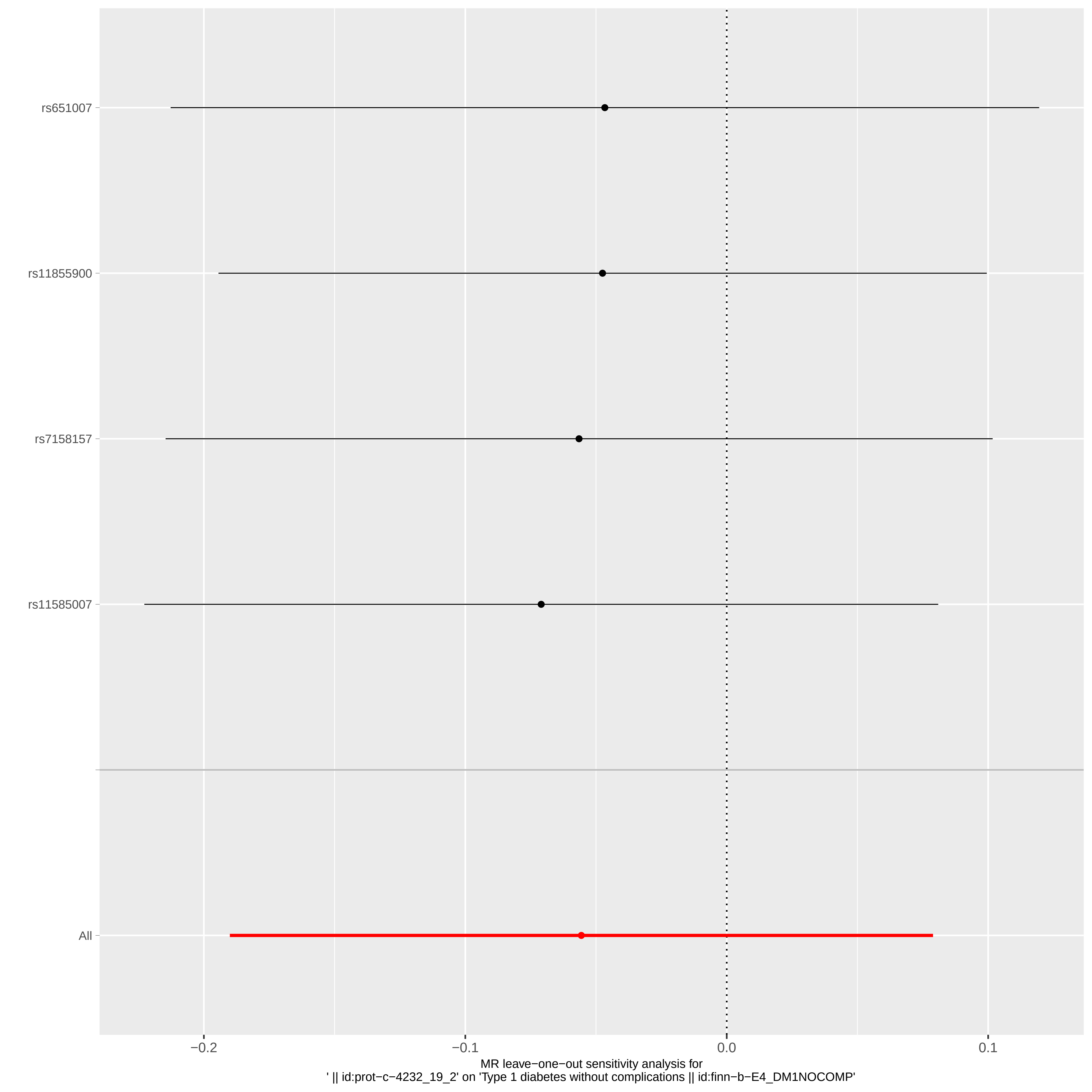

rs651007
rs11855900
rs7158157
rs11585007
All
−0.2
−0.1
0.0
0.1
MR leave−one−out sensitivity analysis for
' || id:prot−c−4232_19_2' on 'Type 1 diabetes without complications || id:finn−b−E4_DM1NOCOMP'

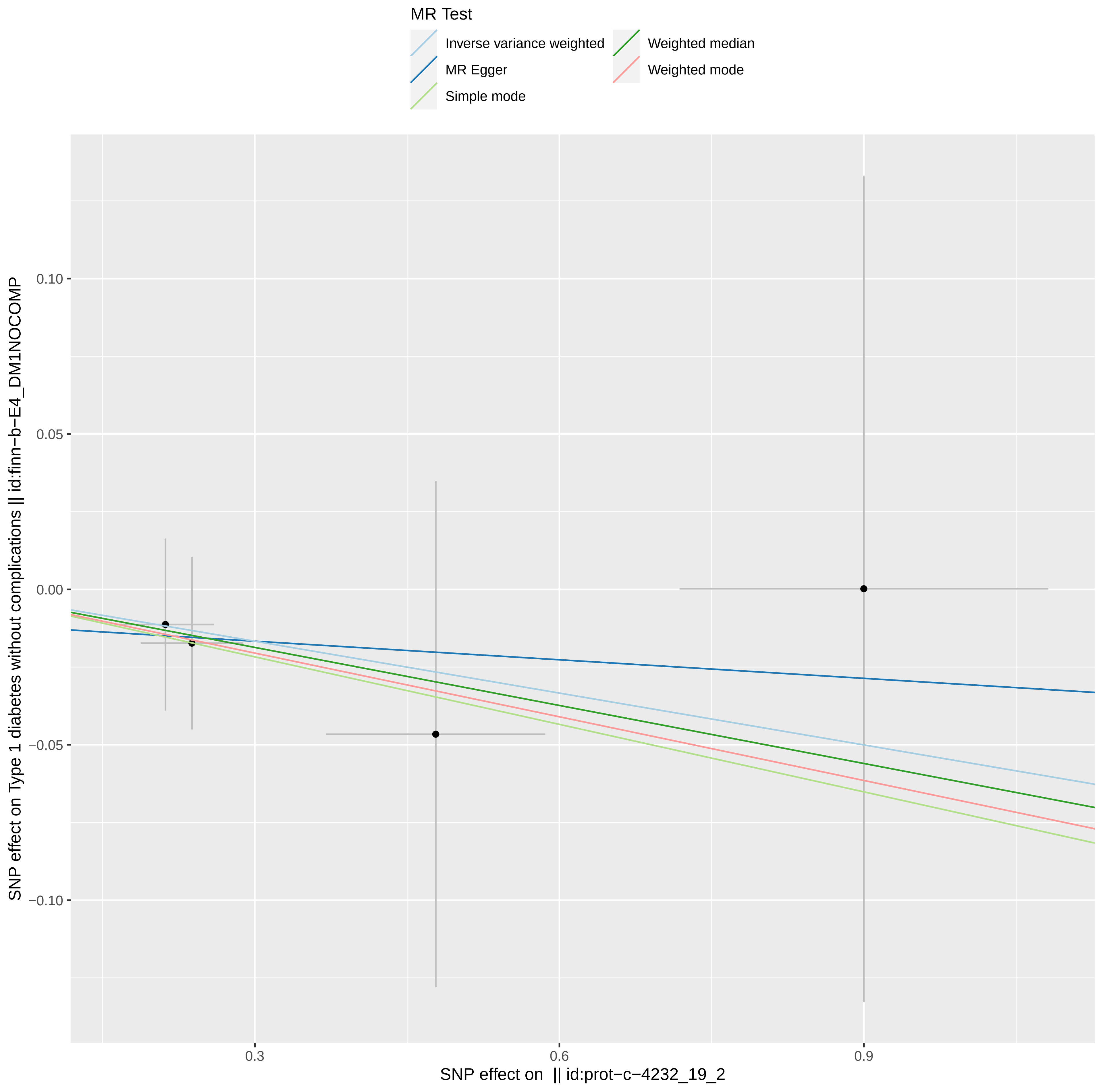
MR Test
Inverse variance weighted
MR Egger
Simple mode
Weighted median
Weighted mode
0.10
0.05
0.00
−0.05
−0.10
0.3
0.6
0.9
SNP effect on Type 1 diabetes without complications || id:finn−b−E4_DM1NOCOMP
SNP effect on || id:prot−c−4232_19_2

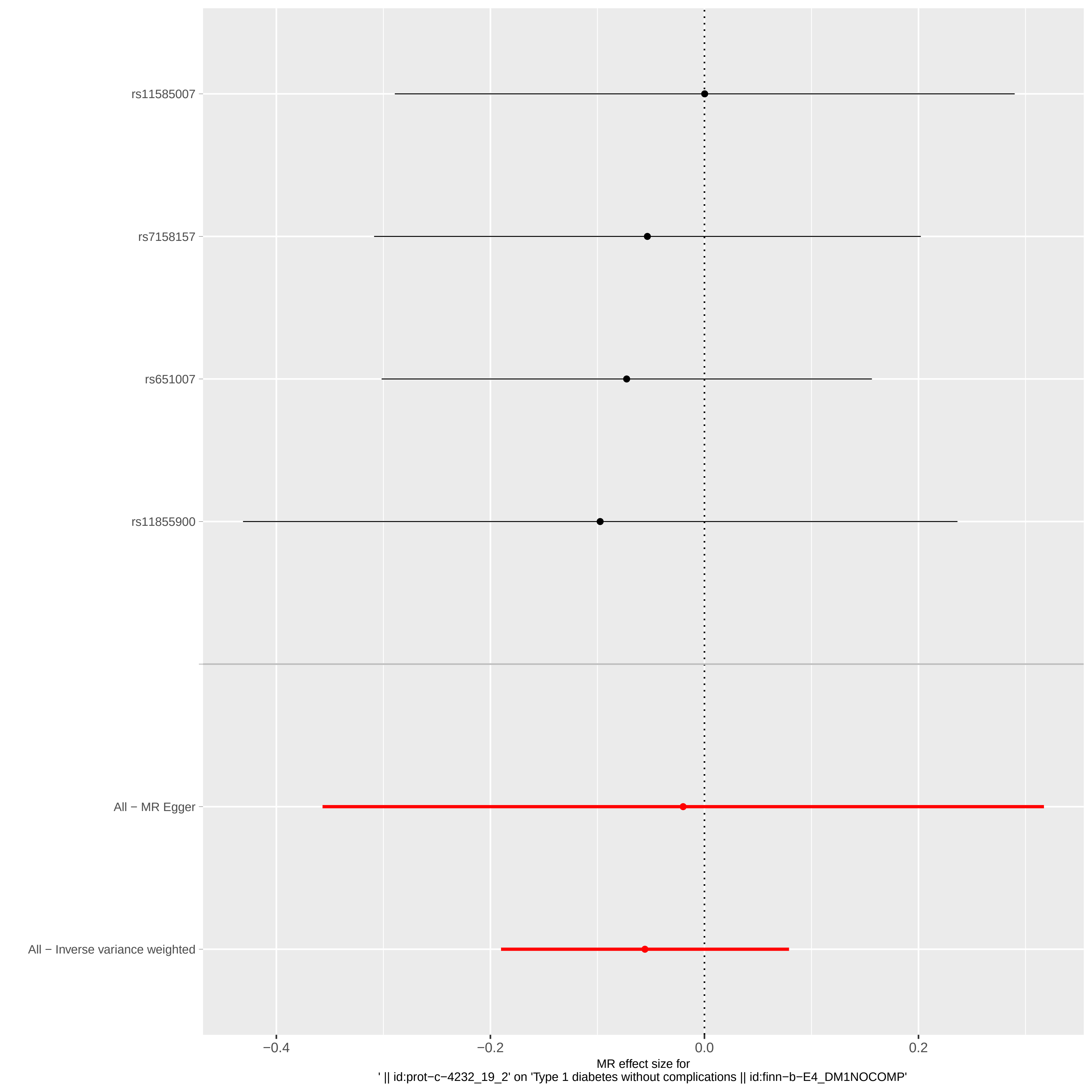
rs11585007
rs7158157
rs651007
rs11855900
All − MR Egger
All − Inverse variance weighted
−0.4
−0.2
0.0
0.2
MR effect size for
' || id:prot−c−4232_19_2' on 'Type 1 diabetes without complications || id:finn−b−E4_DM1NOCOMP'

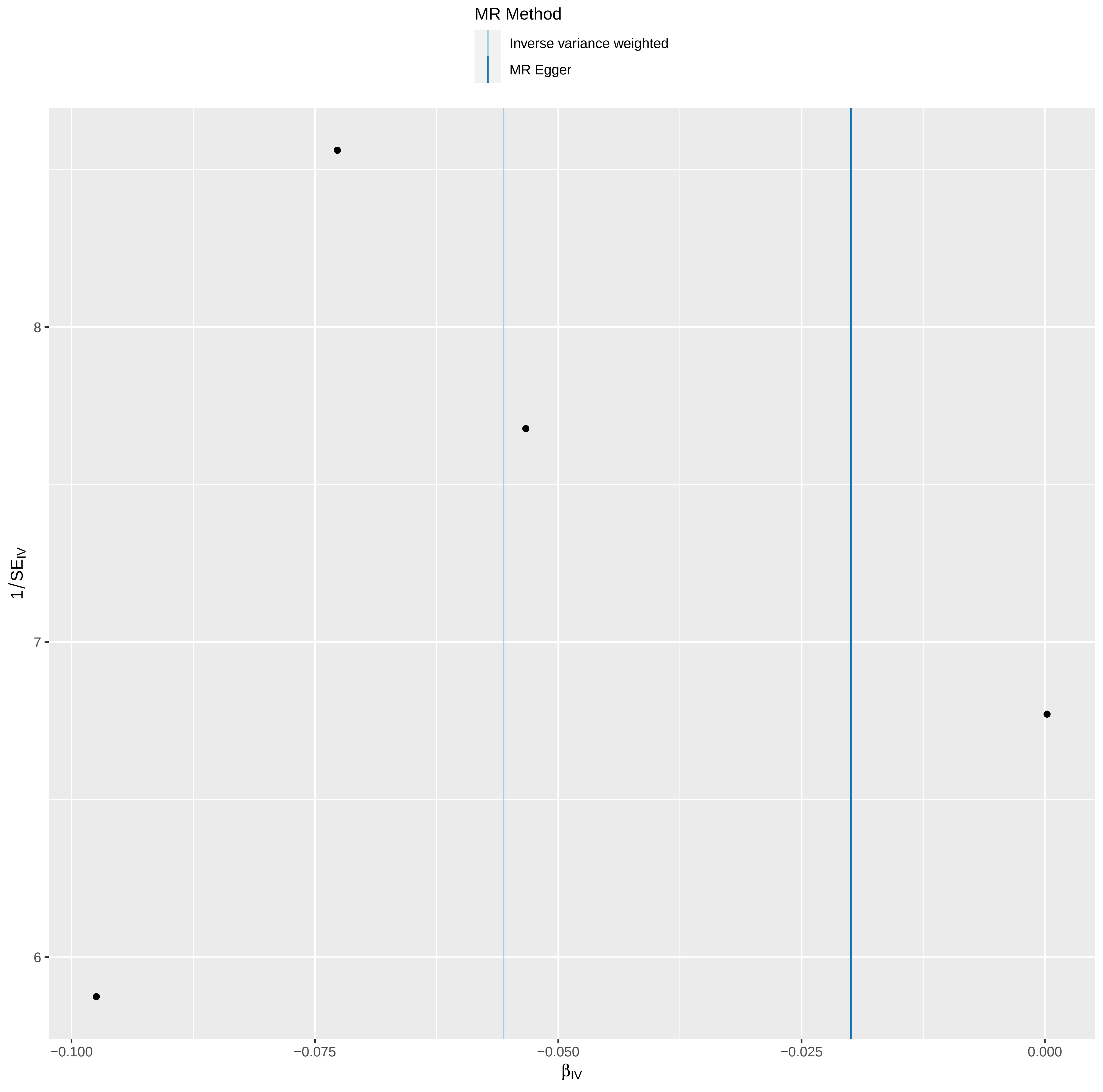
MR Method
Inverse variance weighted
MR Egger
8
7
6
$1/SE_{IV}$
−0.100
−0.075
−0.050
−0.025
0.000
β_{IV}

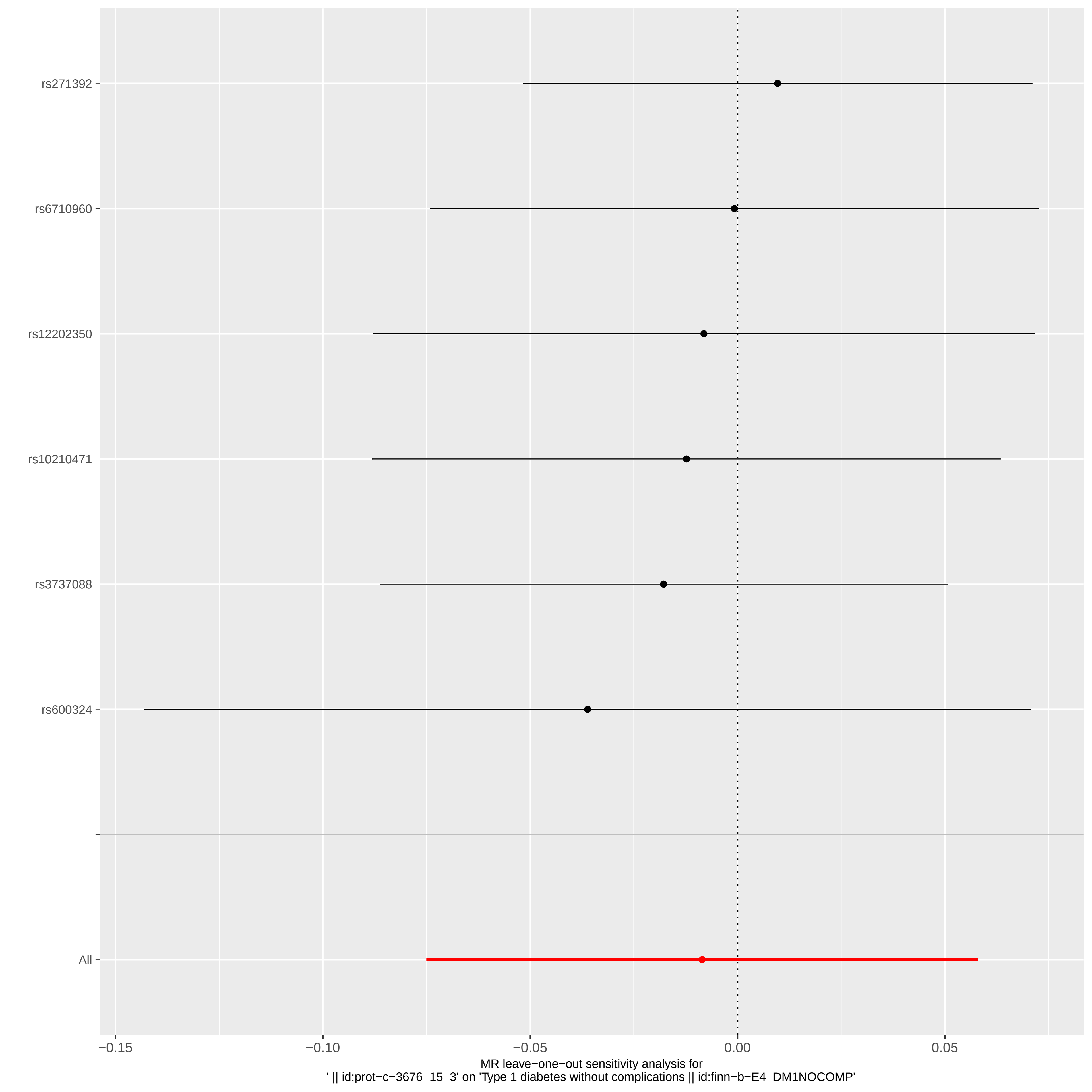

rs271392
rs6710960
rs12202350
rs10210471
rs3737088
rs600324
All
−0.15
−0.10
−0.05
0.00
0.05
MR leave−one−out sensitivity analysis for
' || id:prot−c−3676_15_3' on 'Type 1 diabetes without complications || id:finn−b−E4_DM1NOCOMP'

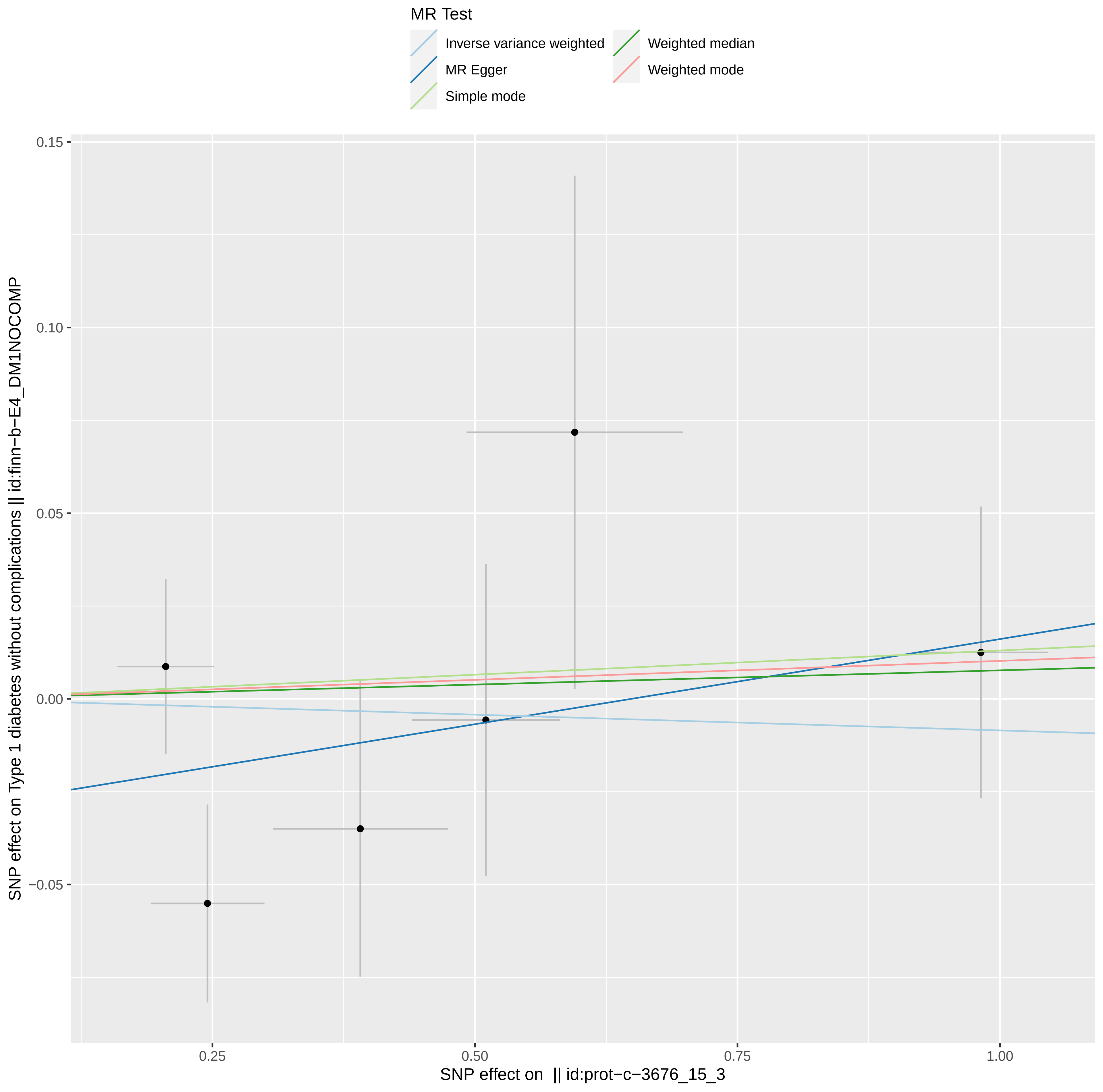

MR Test
Inverse variance weighted
MR Egger
Simple mode
Weighted median
Weighted mode
0.15
0.10
0.05
0.00
−0.05
0.25
0.50
0.75
1.00
SNP effect on Type 1 diabetes without complications || id:finn−b−E4_DM1NOCOMP
SNP effect on || id:prot−c−3676_15_3

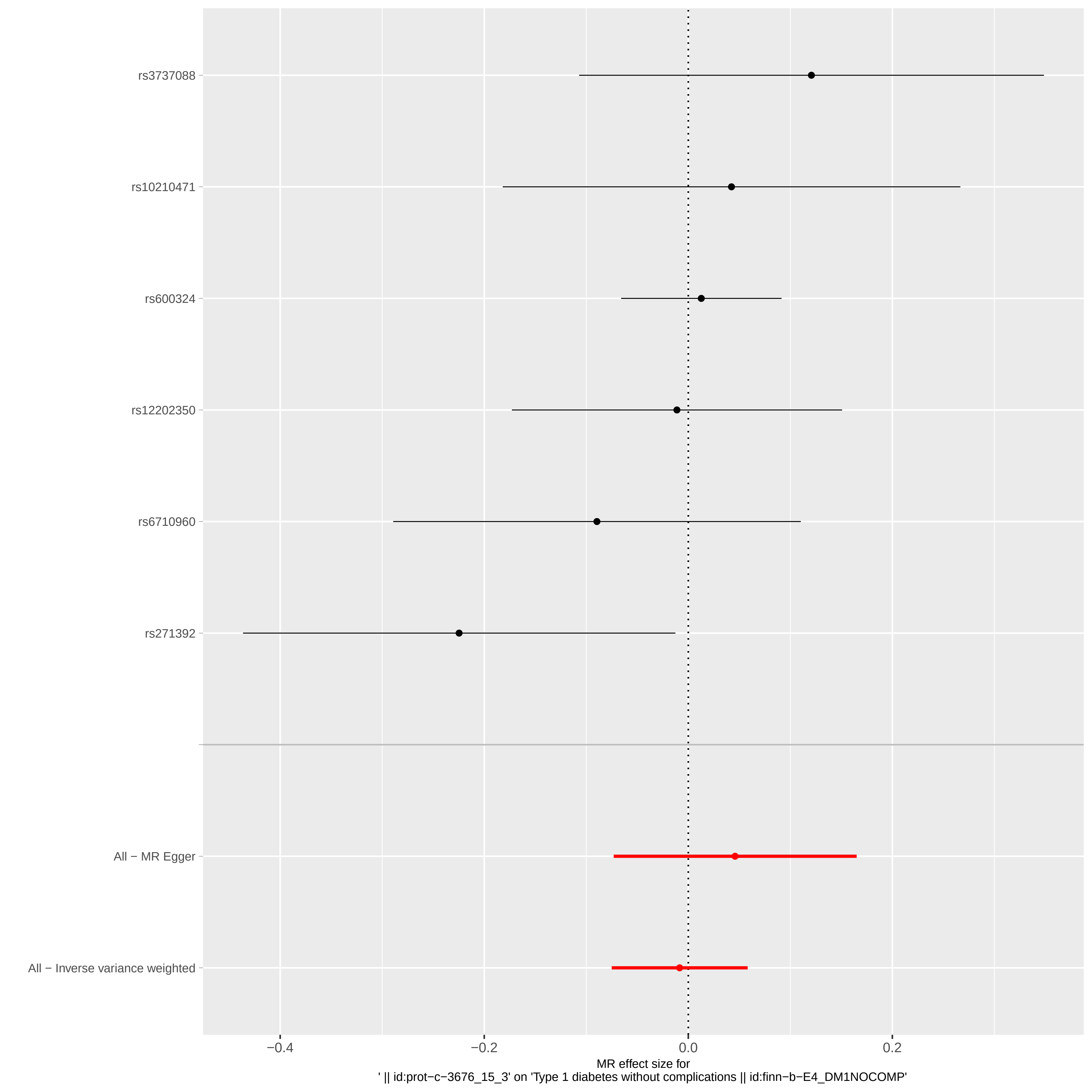
rs3737088
rs10210471
rs600324
rs12202350
rs6710960
rs271392
All − MR Egger
All − Inverse variance weighted
−0.4
−0.2
0.0
0.2
MR effect size for
' || id:prot−c−3676_15_3' on 'Type 1 diabetes without complications || id:finn−b−E4_DM1NOCOMP'

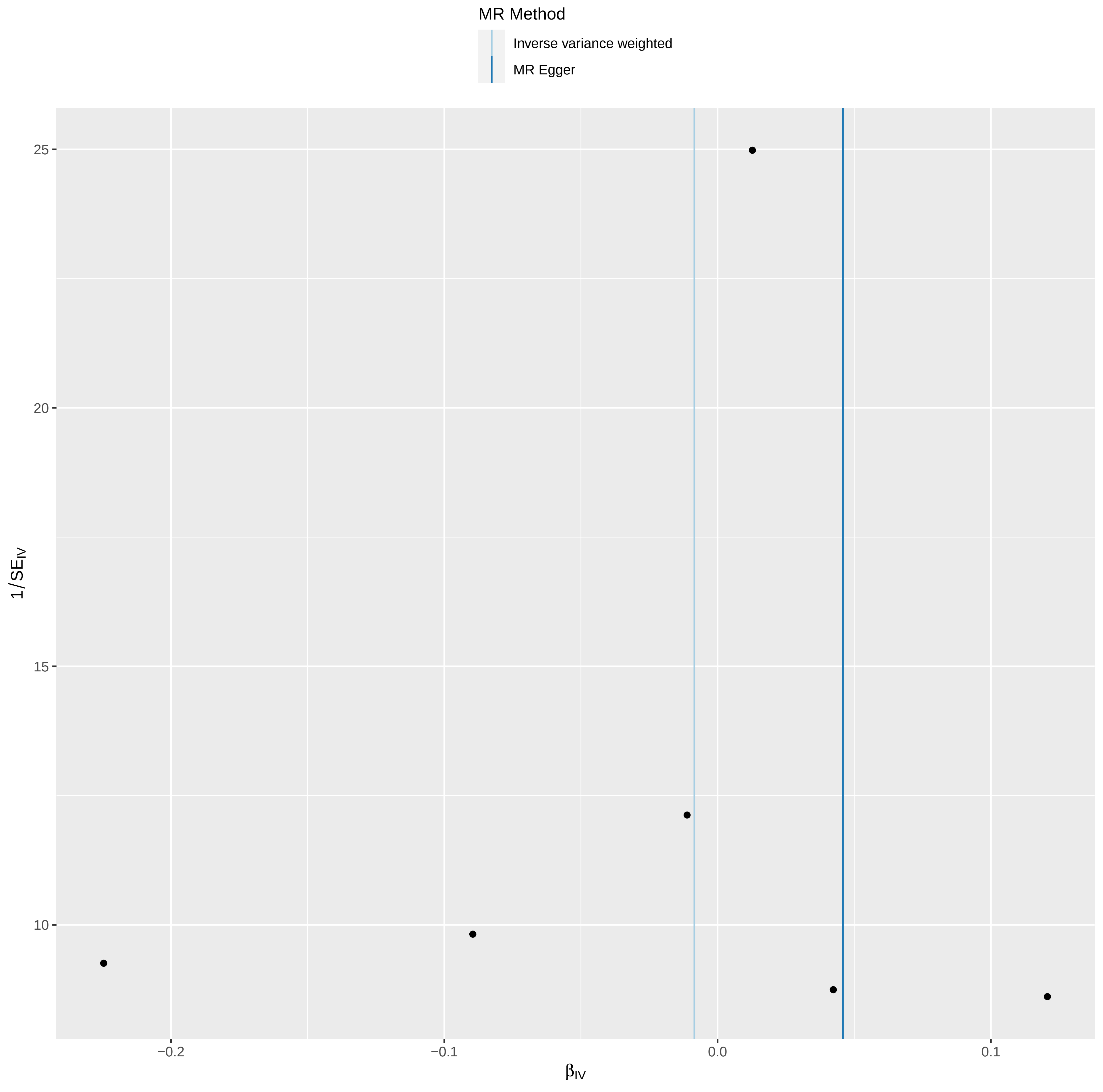

MR Method
Inverse variance weighted
MR Egger
25
20
15
10
1/SE_IV
−0.2
−0.1
0.0
0.1
β_IV

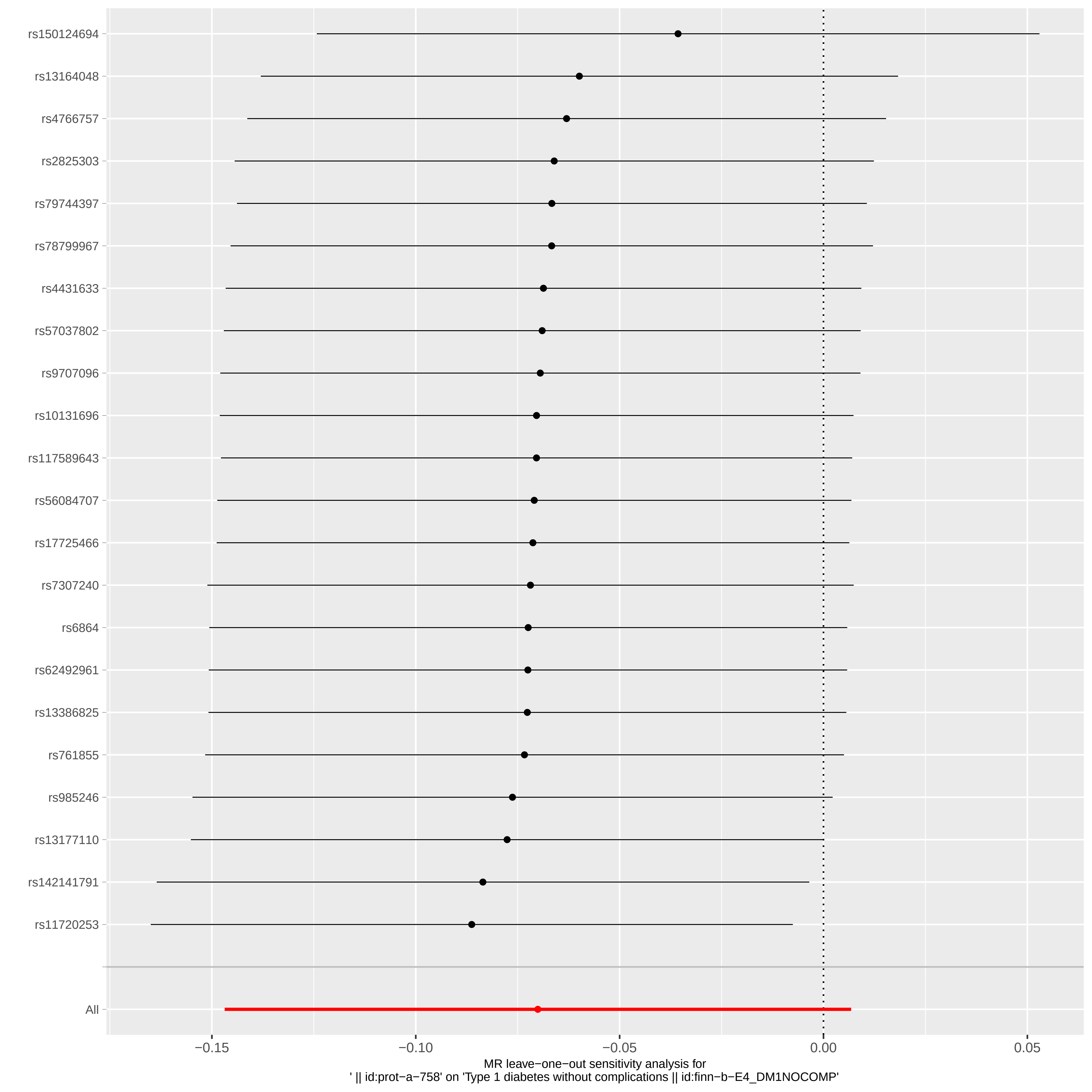
rs150124694
rs13164048
rs4766757
rs2825303
rs79744397
rs78799967
rs4431633
rs57037802
rs9707096
rs10131696
rs117589643
rs56084707
rs17725466
rs7307240
rs6864
rs62492961
rs13386825
rs761855
rs985246
rs13177110
rs142141791
rs11720253
All
−0.15
−0.10
−0.05
0.00
0.05
MR leave−one−out sensitivity analysis for
' || id:prot−a−758' on 'Type 1 diabetes without complications || id:finn−b−E4_DM1NOCOMP'

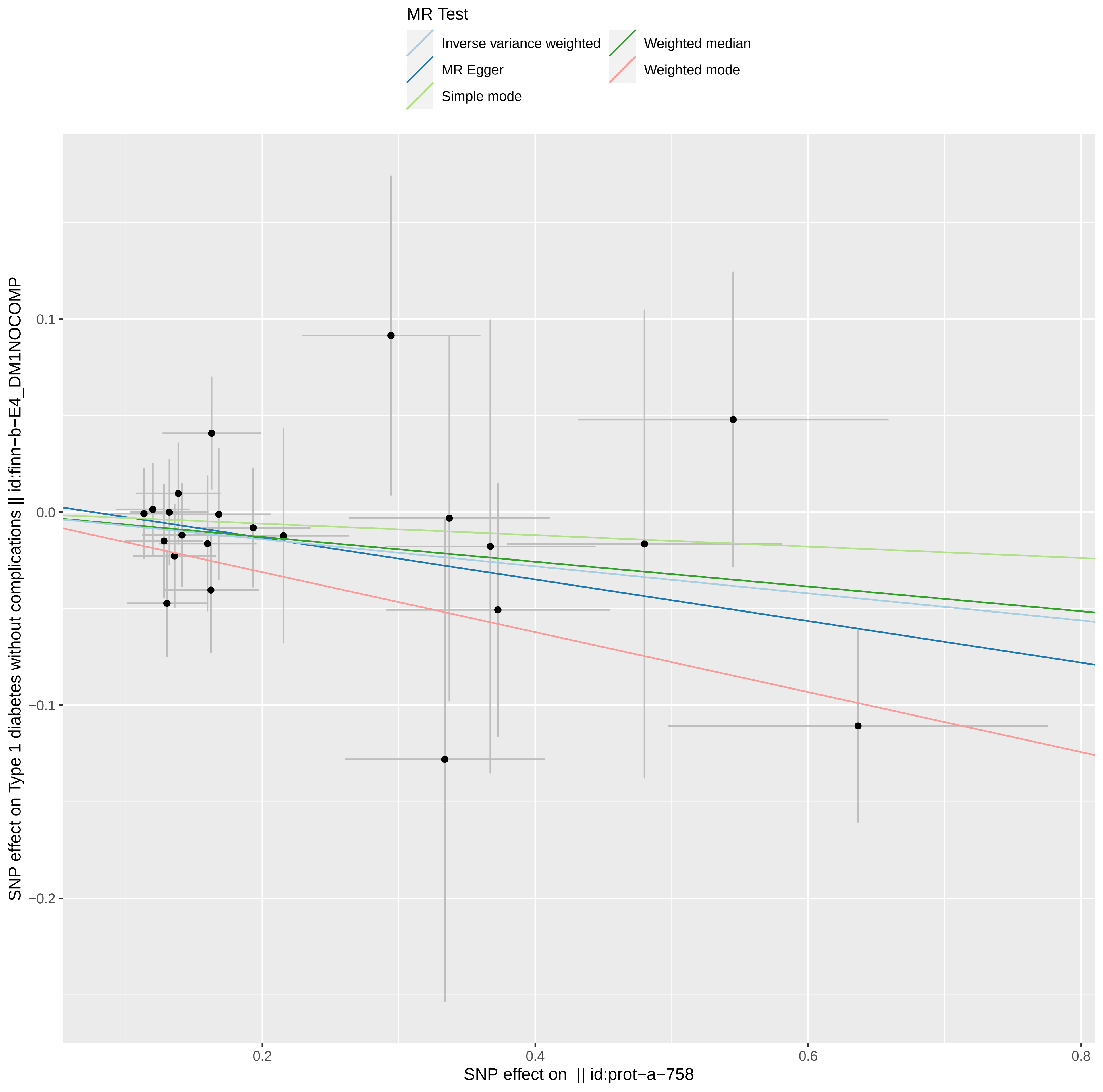

MR Test
Inverse variance weighted
MR Egger
Simple mode
Weighted median
Weighted mode
SNP effect on Type 1 diabetes without complications || id:finn-b-E4_DM1NOCOMP
0.1
0.0
−0.1
−0.2
0.2
0.4
0.6
0.8
SNP effect on || id:prot-a-758

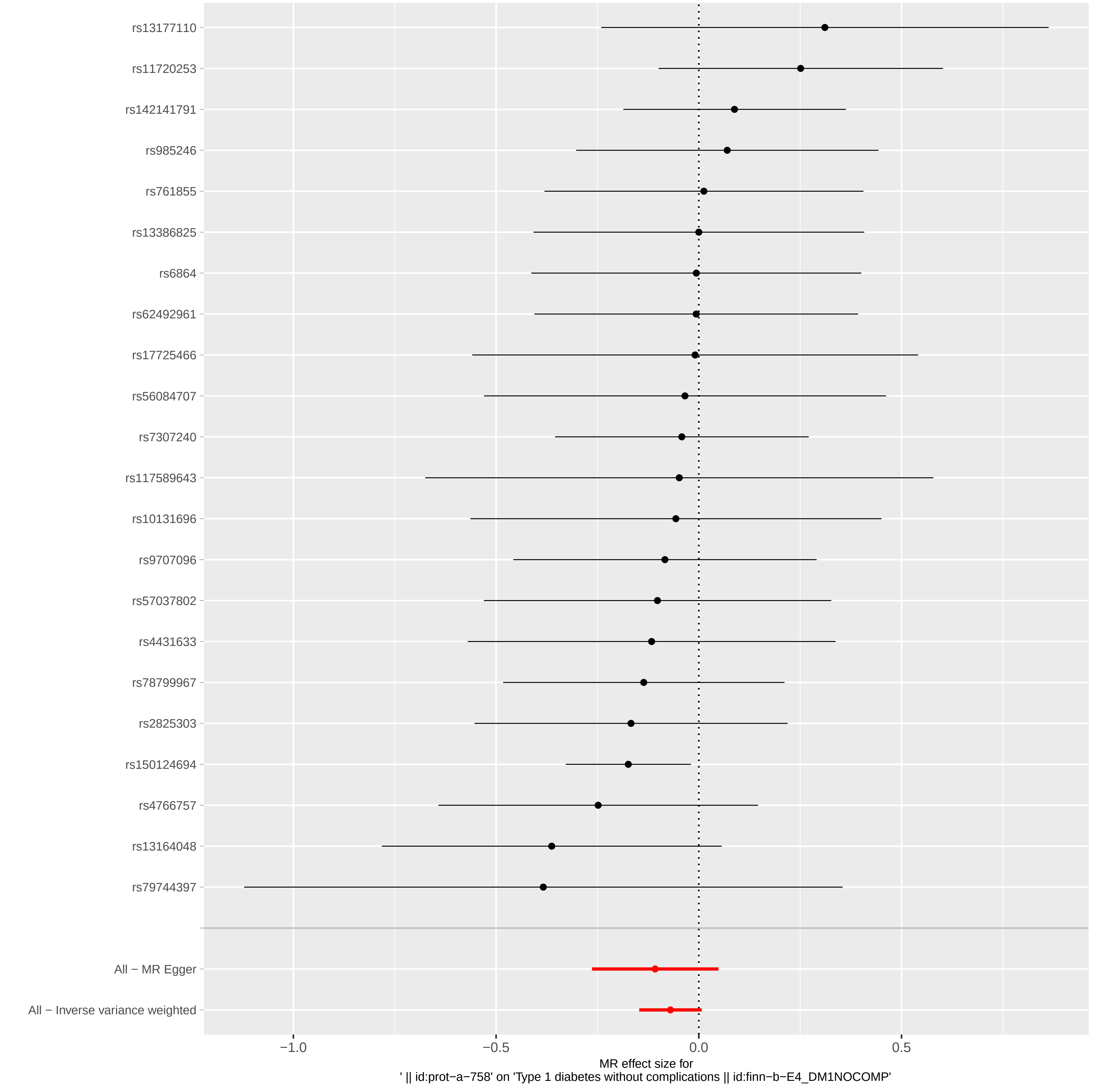
rs13177110
rs11720253
rs142141791
rs985246
rs761855
rs13386825
rs6864
rs62492961
rs17725466
rs56084707
rs7307240
rs117589643
rs10131696
rs9707096
rs57037802
rs4431633
rs78799967
rs2825303
rs150124694
rs4766757
rs13164048
rs79744397
All − MR Egger
All − Inverse variance weighted
−1.0
−0.5
0.0
0.5
MR effect size for
' || id:prot−a−758' on 'Type 1 diabetes without complications || id:finn−b−E4_DM1NOCOMP'

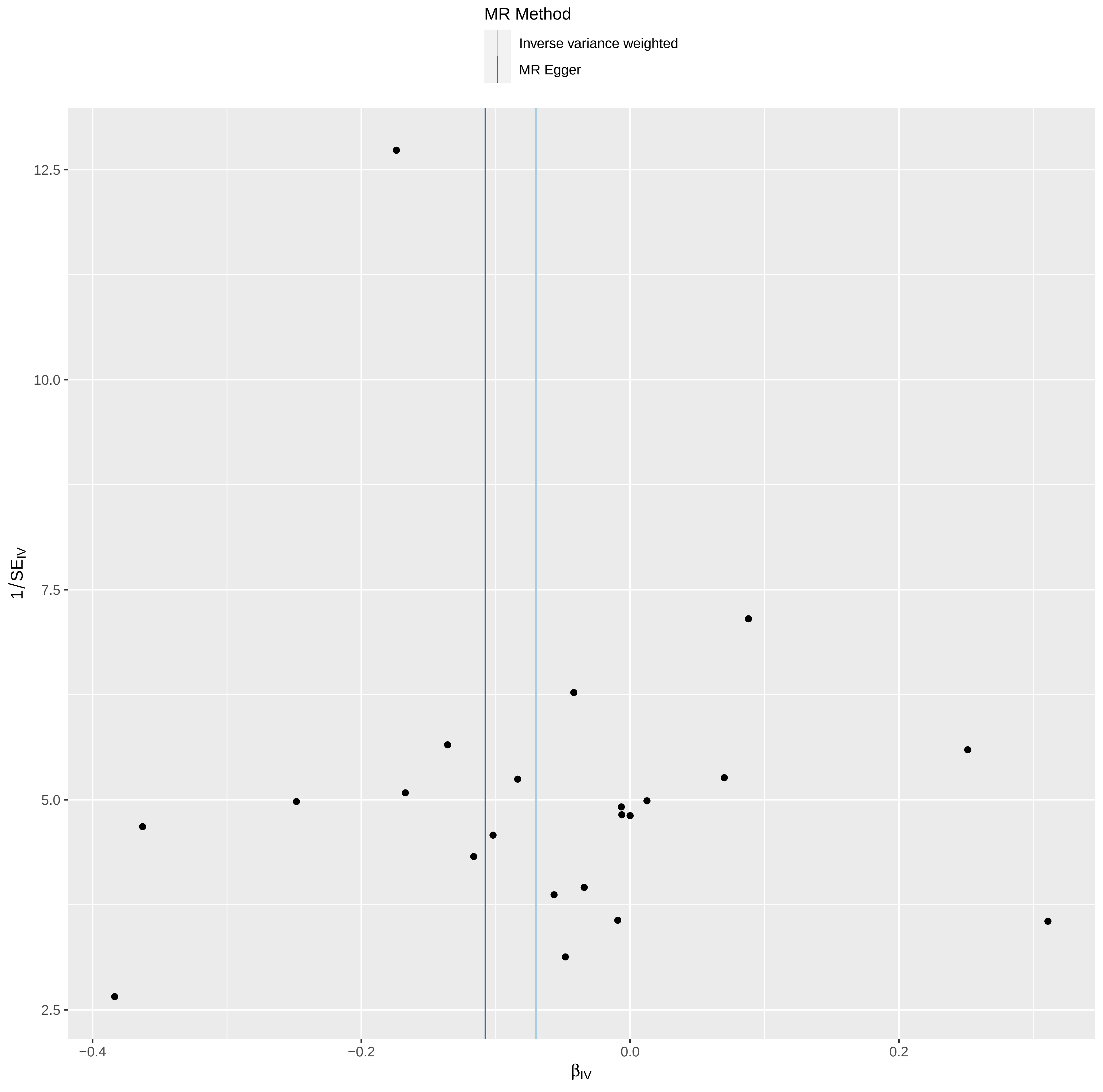

MR Method
Inverse variance weighted
MR Egger
12.5
10.0
7.5
5.0
2.5
$1/SE_{IV}$
−0.4
−0.2
0.0
0.2
β_{IV}
