# Supplementary material for: Mendelian randomization analysis demonstrates the causal effects of IGF family members in diabetes
Source: Front Med (Lausanne). 2024 Feb 5;11:1332162. doi: 10.3389/fmed.2024.1332162 (PMC10875044; doi:10.3389/fmed.2024.1332162)
Supplement: Supplementary file 2 [file Data_Sheet_2.PDF]

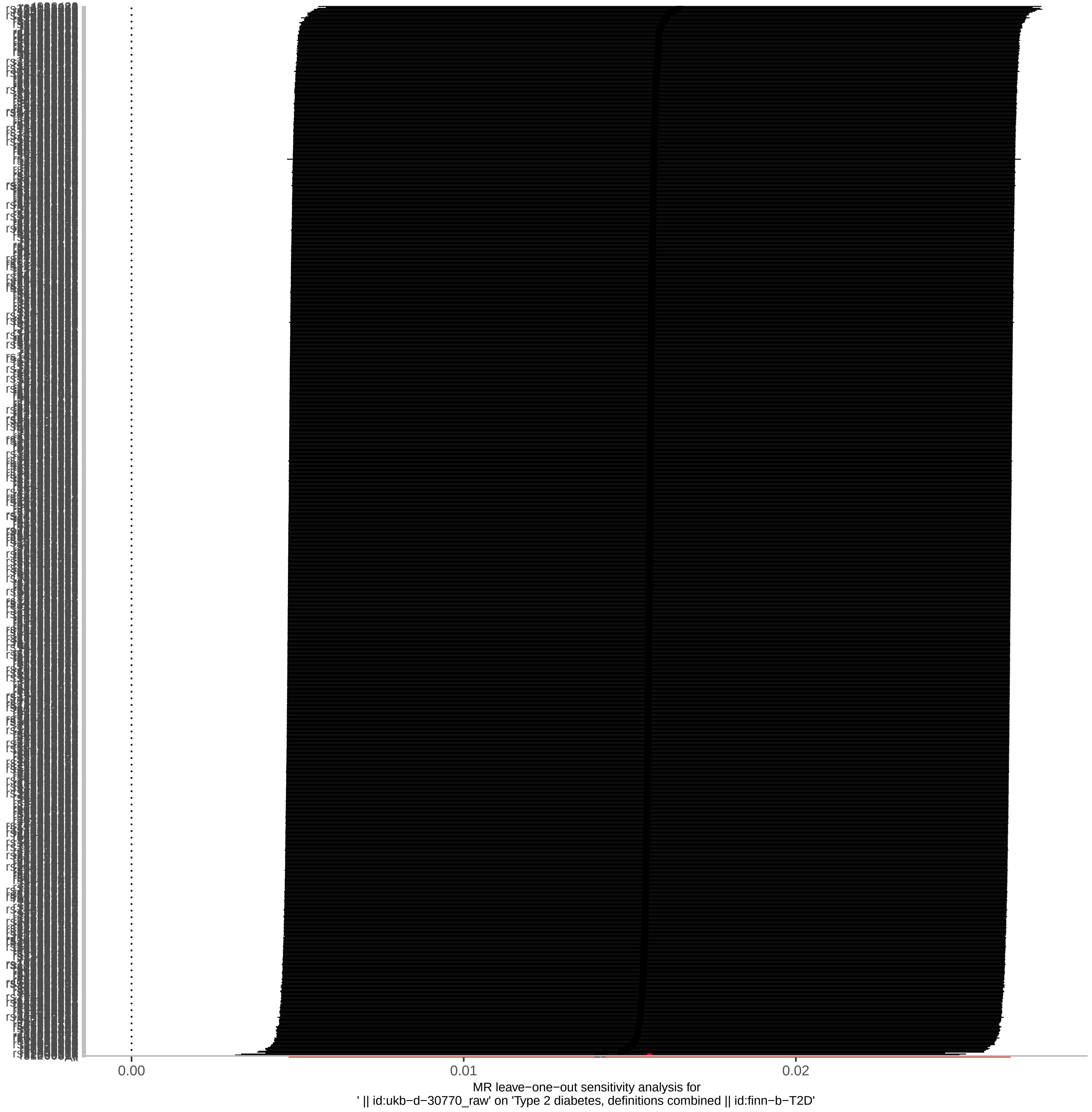

0.00
0.01
0.02
MR leave−one−out sensitivity analysis for
' || id:ukb−d−30770_raw' on 'Type 2 diabetes, definitions combined || id:finn−b−T2D'

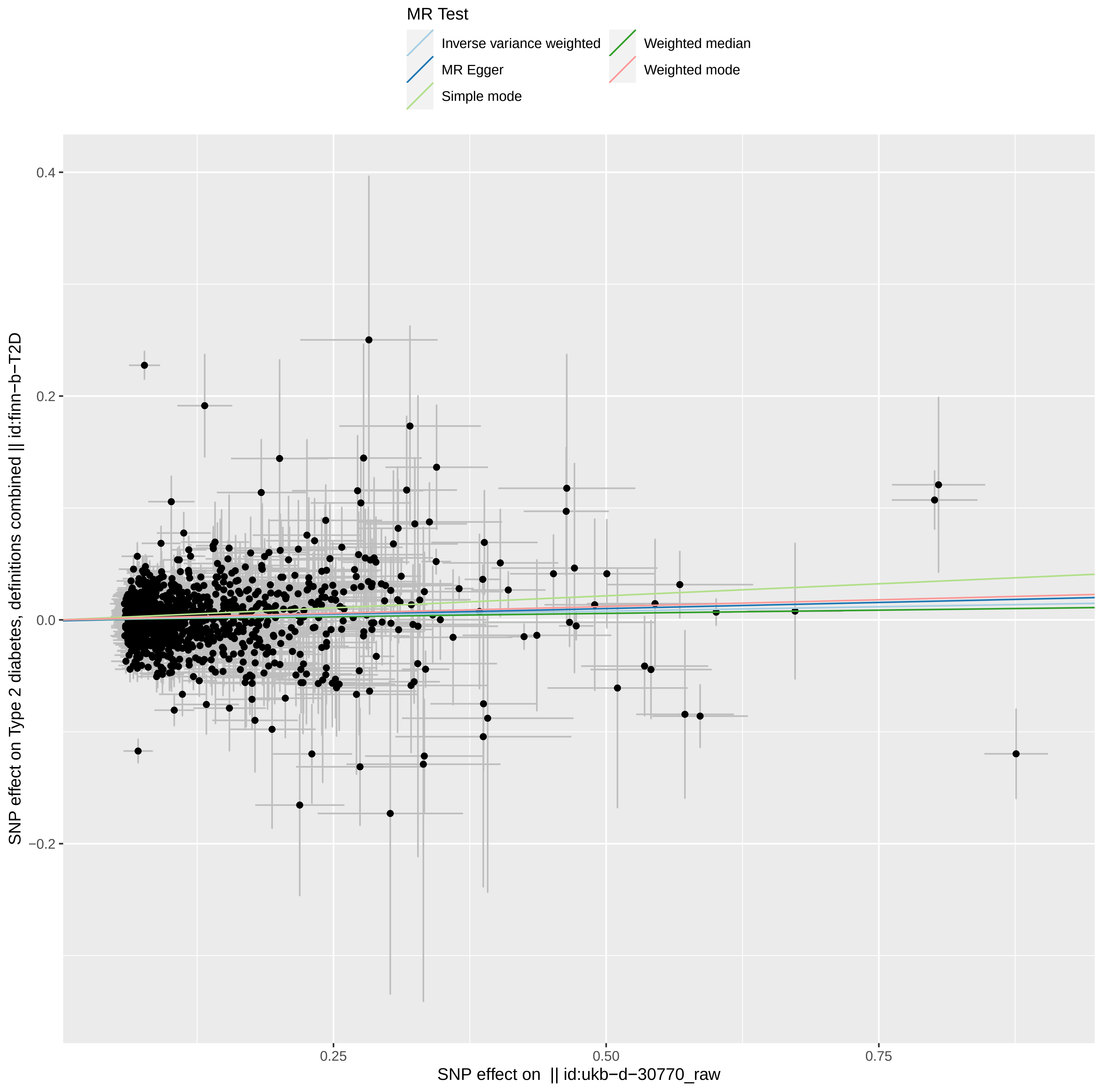

MR Test
Inverse variance weighted
MR Egger
Simple mode
Weighted median
Weighted mode
0.4
0.2
0.0
−0.2
0.25
0.50
0.75
SNP effect on Type 2 diabetes, definitions combined || id:finn−b−T2D
SNP effect on || id:ukb−d−30770_raw

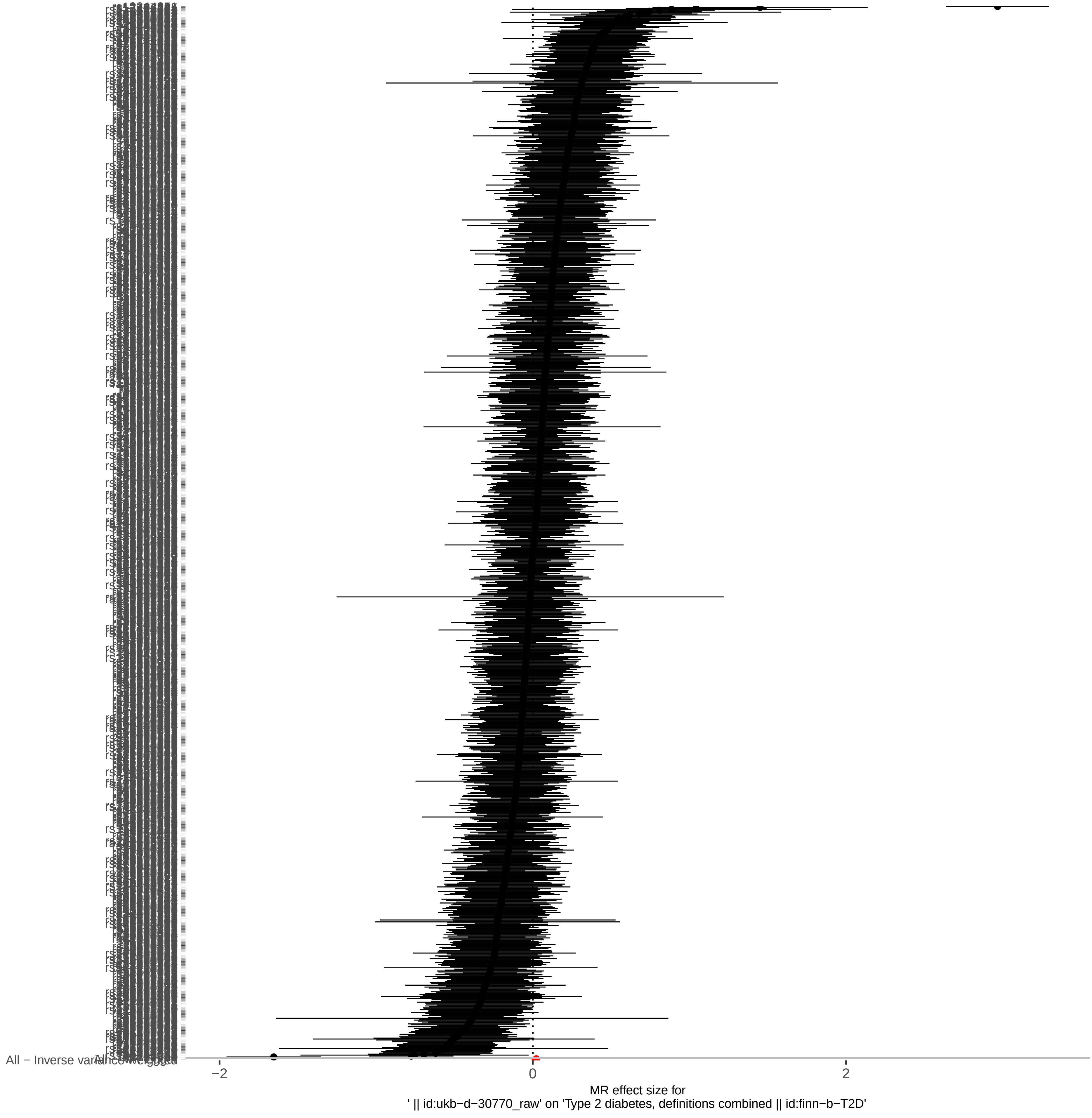

All − Inverse variance weighted
−2
0
2
MR effect size for
' || id:ukb−d−30770_raw' on 'Type 2 diabetes, definitions combined || id:finn−b−T2D'

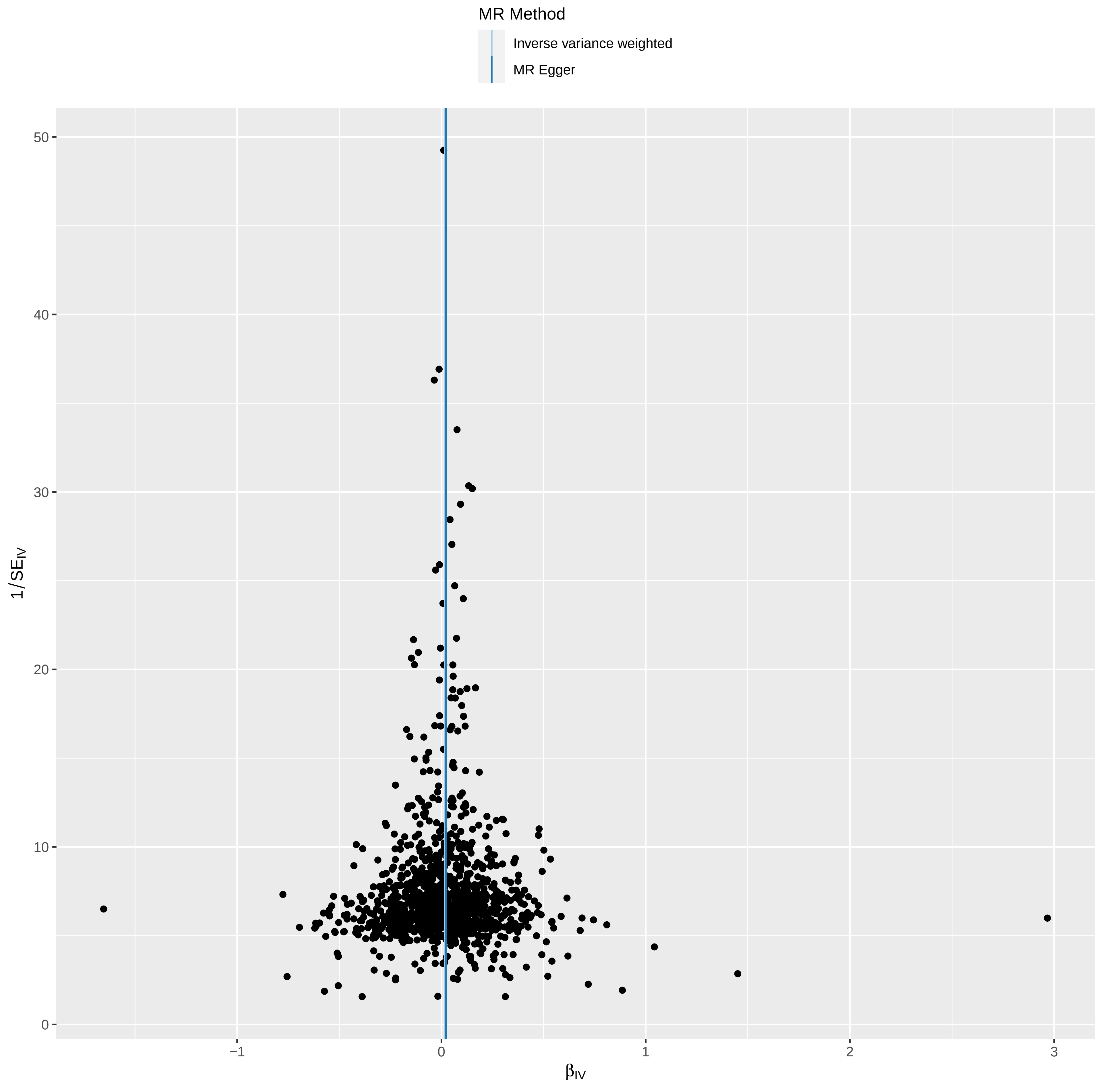
MR Method
Inverse variance weighted
MR Egger
1/SE_IV
β_IV
50
40
30
20
10
0
−1
0
1
2
3

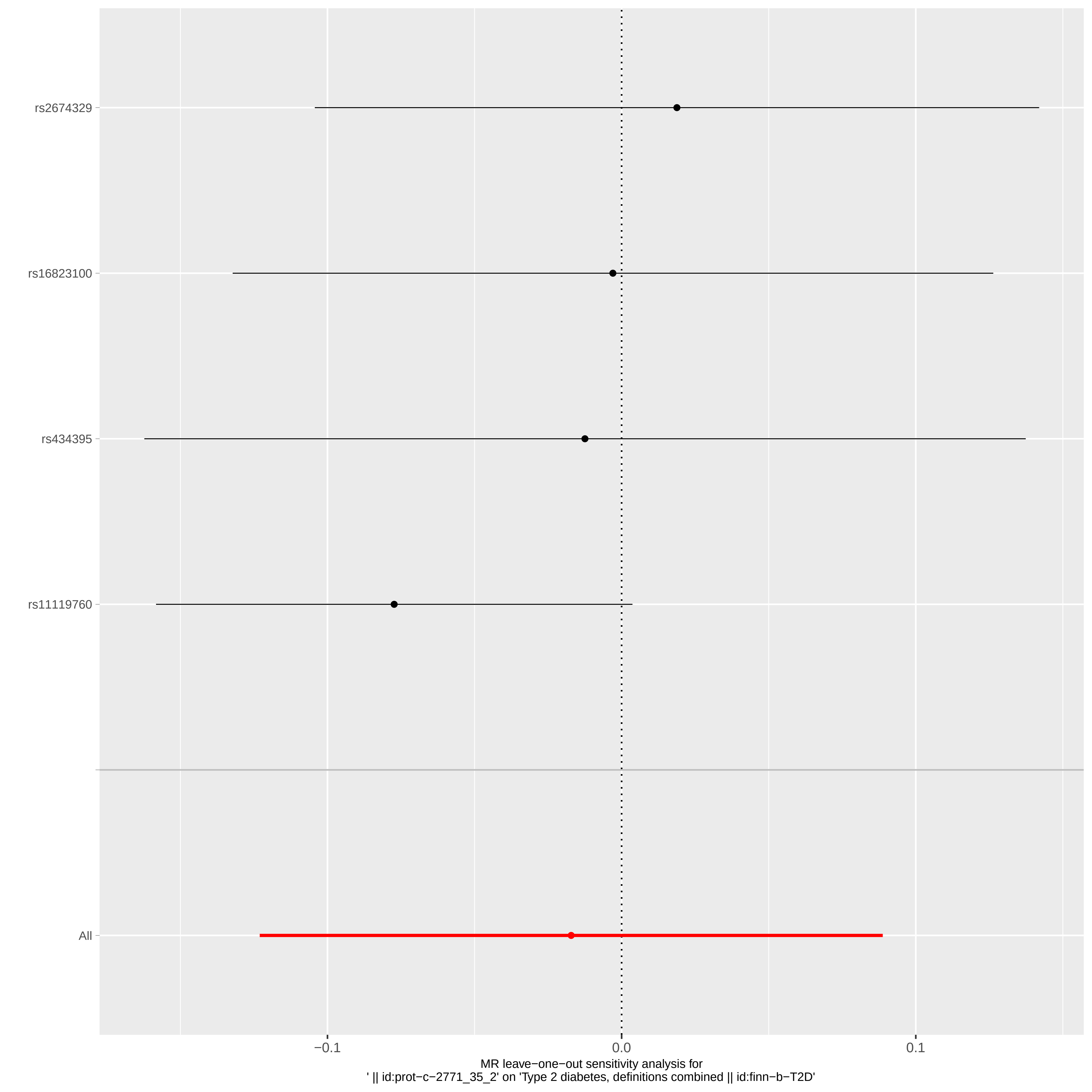
rs2674329
rs16823100
rs434395
rs11119760
All
−0.1
0.0
0.1
MR leave−one−out sensitivity analysis for
' || id:prot−c−2771_35_2' on 'Type 2 diabetes, definitions combined || id:finn−b−T2D'

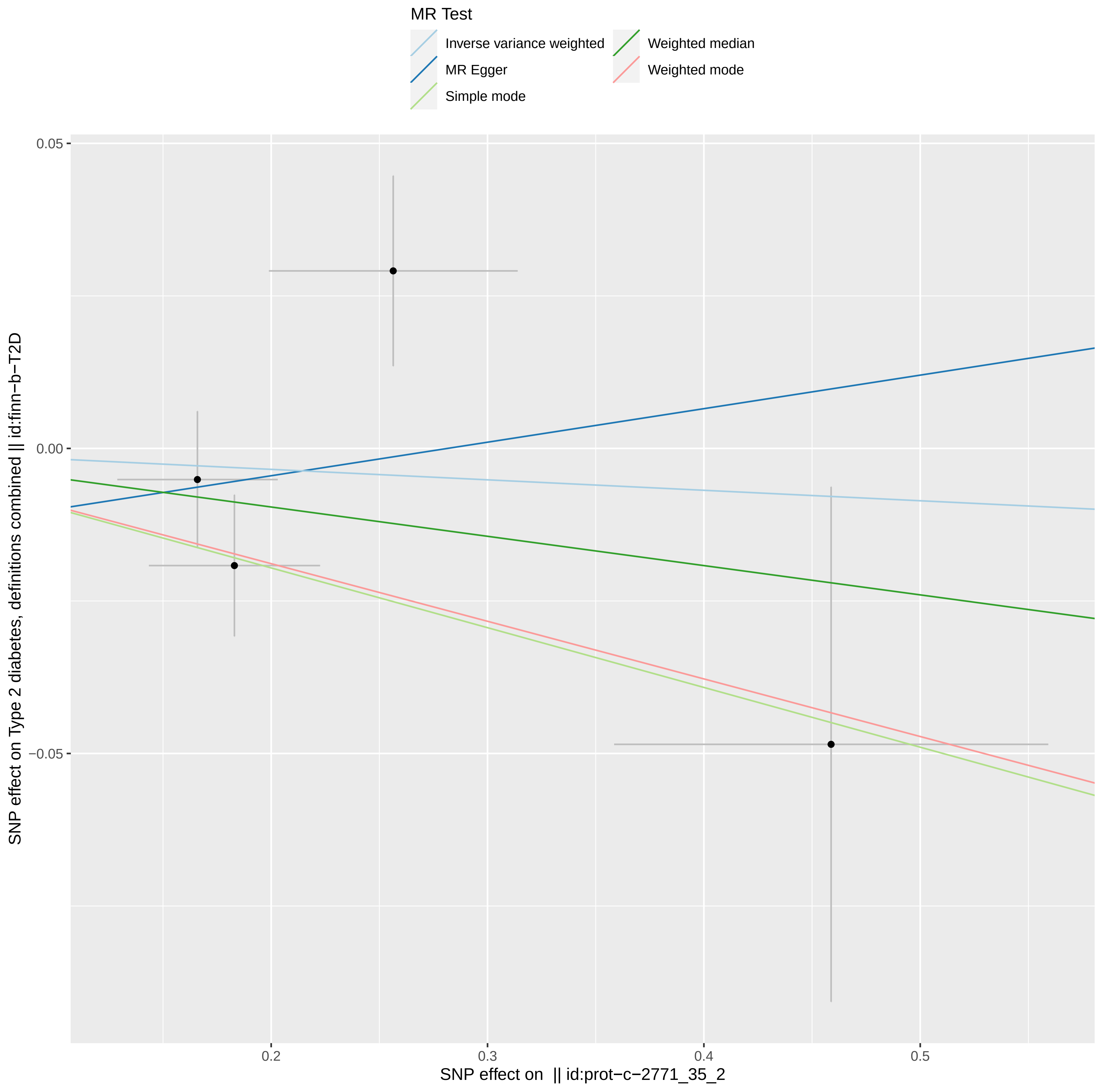

MR Test
Inverse variance weighted
MR Egger
Simple mode
Weighted median
Weighted mode
0.05
0.00
−0.05
0.2
0.3
0.4
0.5
SNP effect on Type 2 diabetes, definitions combined || id:finn−b−T2D
SNP effect on || id:prot−c−2771_35_2

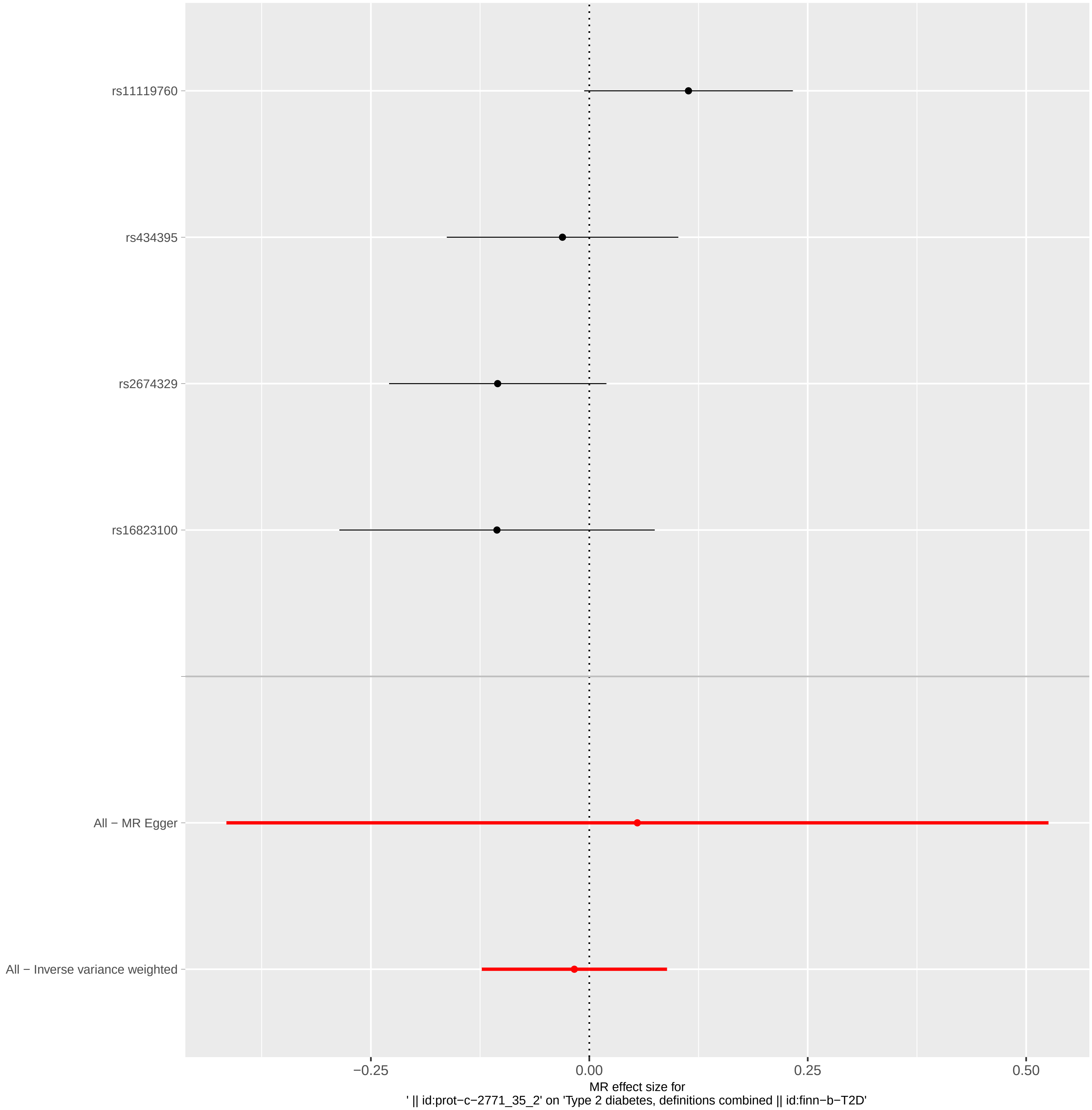
rs11119760
rs434395
rs2674329
rs16823100
All − MR Egger
All − Inverse variance weighted
−0.25
0.00
0.25
0.50
MR effect size for
' || id:prot−c−2771_35_2' on 'Type 2 diabetes, definitions combined || id:finn−b−T2D'

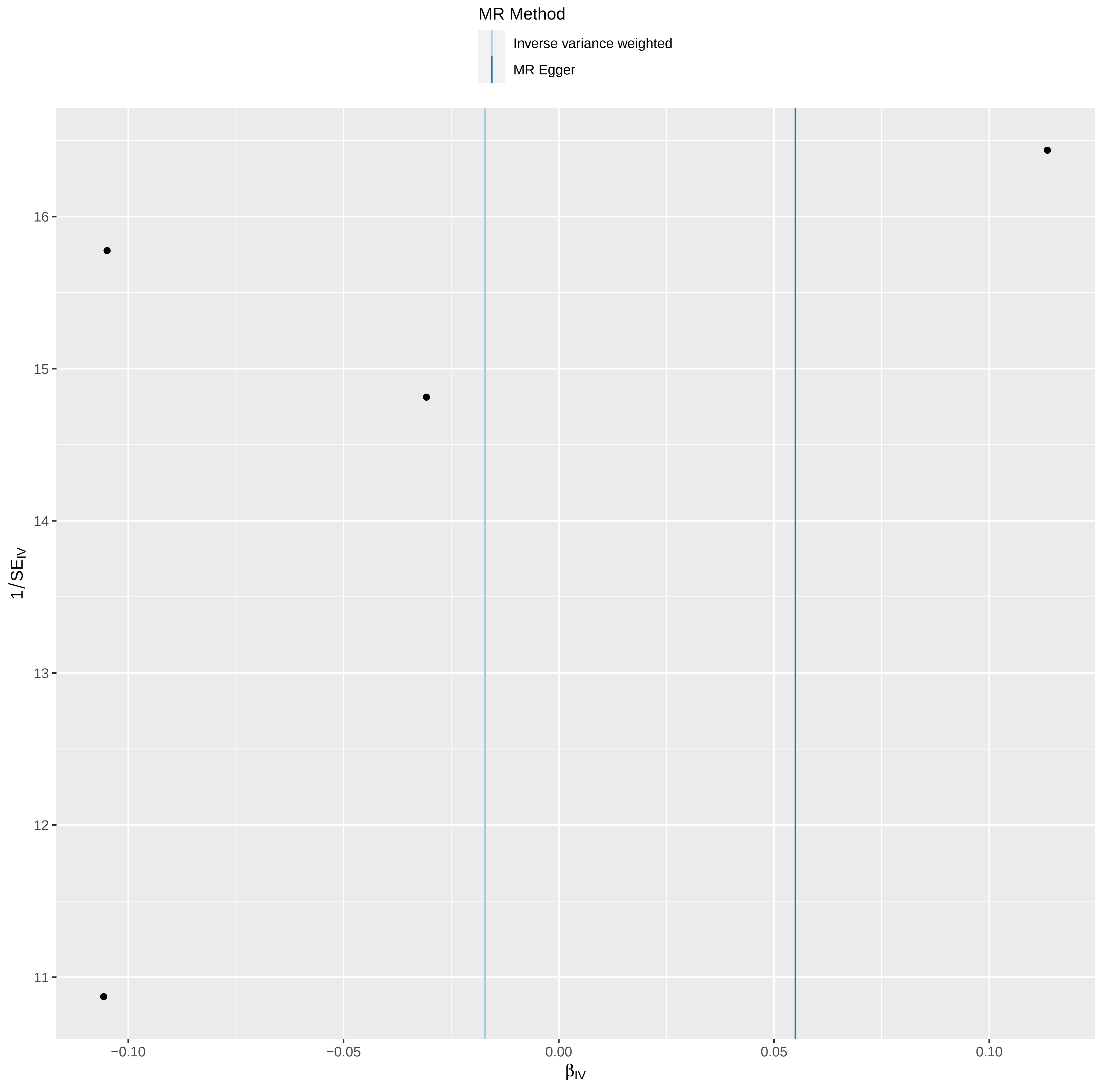

MR Method
Inverse variance weighted
MR Egger
16
15
14
13
12
11
1/SE_IV
−0.10
−0.05
0.00
0.05
0.10
β_IV

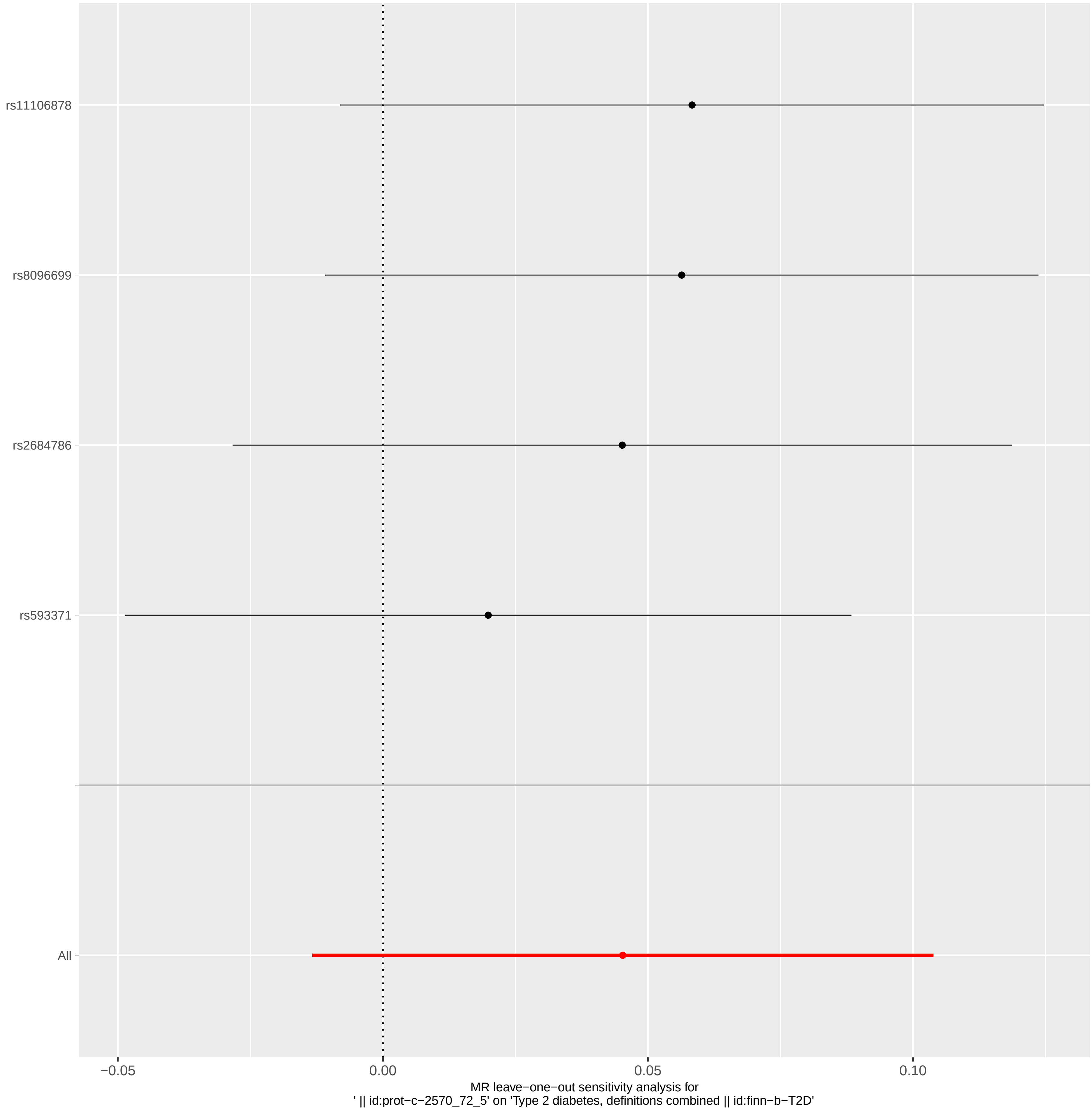

rs11106878
rs8096699
rs2684786
rs593371
All
−0.05
0.00
0.05
0.10
MR leave−one−out sensitivity analysis for
' || id:prot−c−2570_72_5' on 'Type 2 diabetes, definitions combined || id:finn−b−T2D'

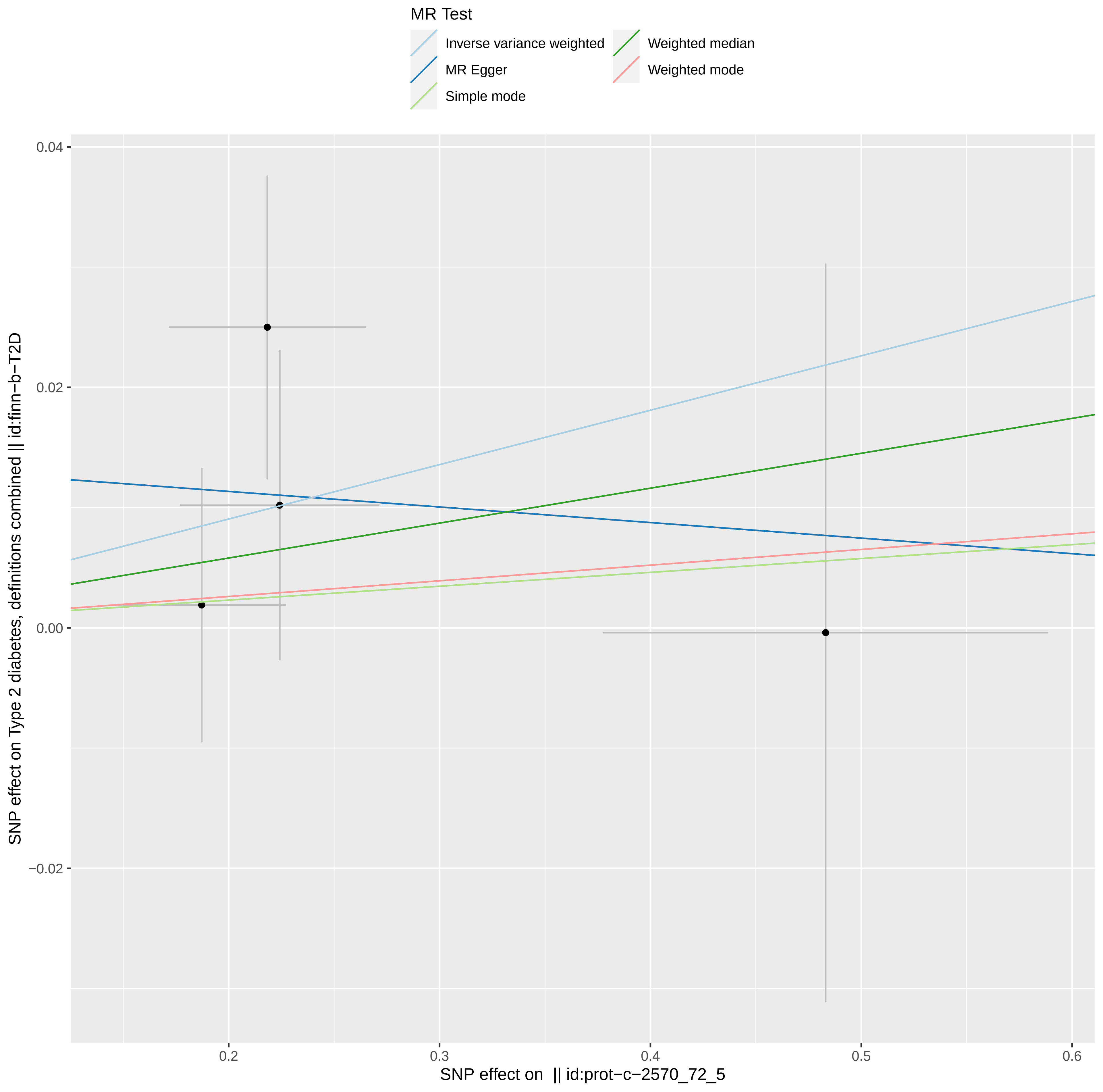
MR Test
Inverse variance weighted
MR Egger
Simple mode
Weighted median
Weighted mode
0.04
0.02
0.00
−0.02
0.2
0.3
0.4
0.5
0.6
SNP effect on Type 2 diabetes, definitions combined || id:finn−b−T2D
SNP effect on || id:prot−c−2570_72_5

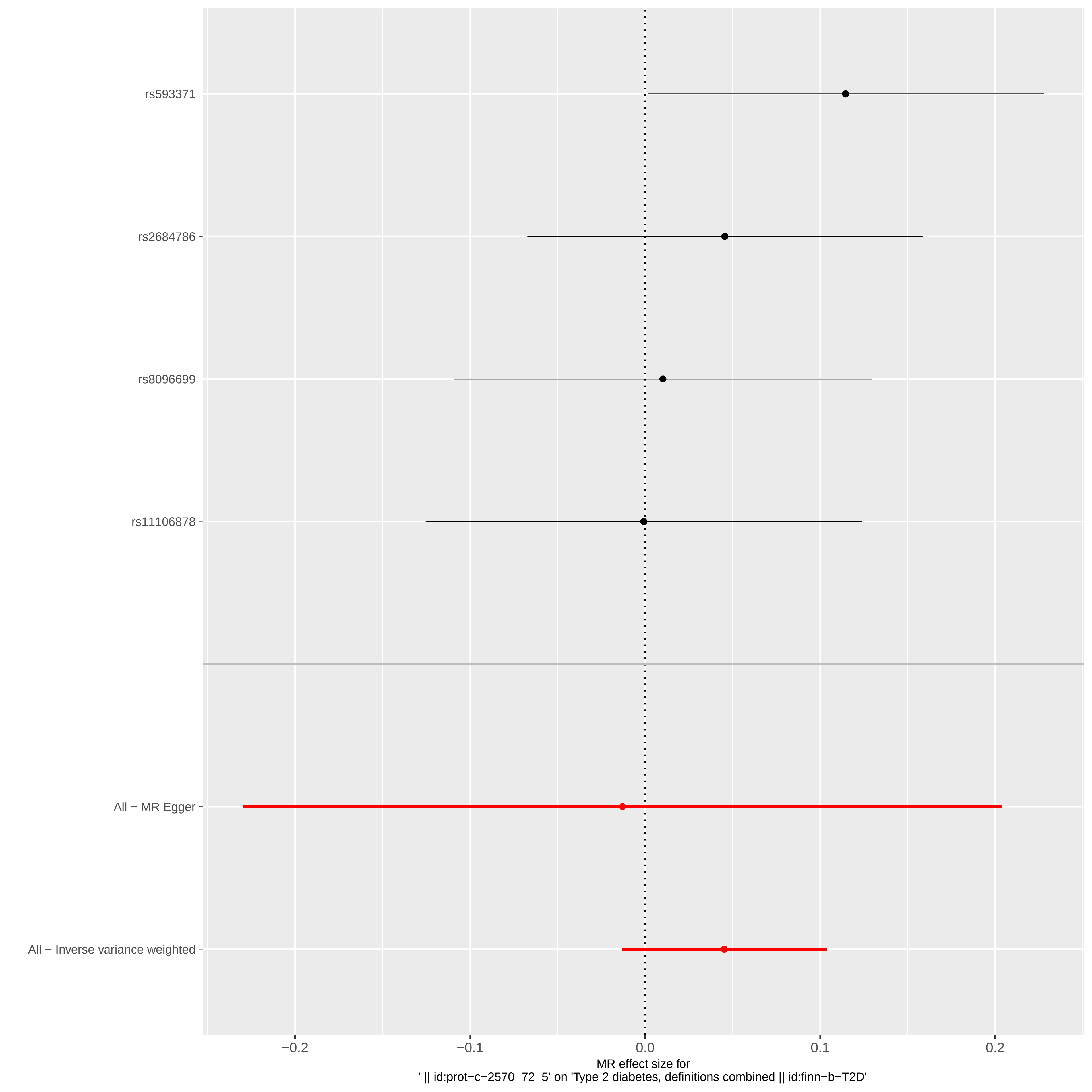
rs593371
rs2684786
rs8096699
rs11106878
All − MR Egger
All − Inverse variance weighted
−0.2
−0.1
0.0
0.1
0.2
MR effect size for
' || id:prot−c−2570_72_5' on 'Type 2 diabetes, definitions combined || id:finn−b−T2D'

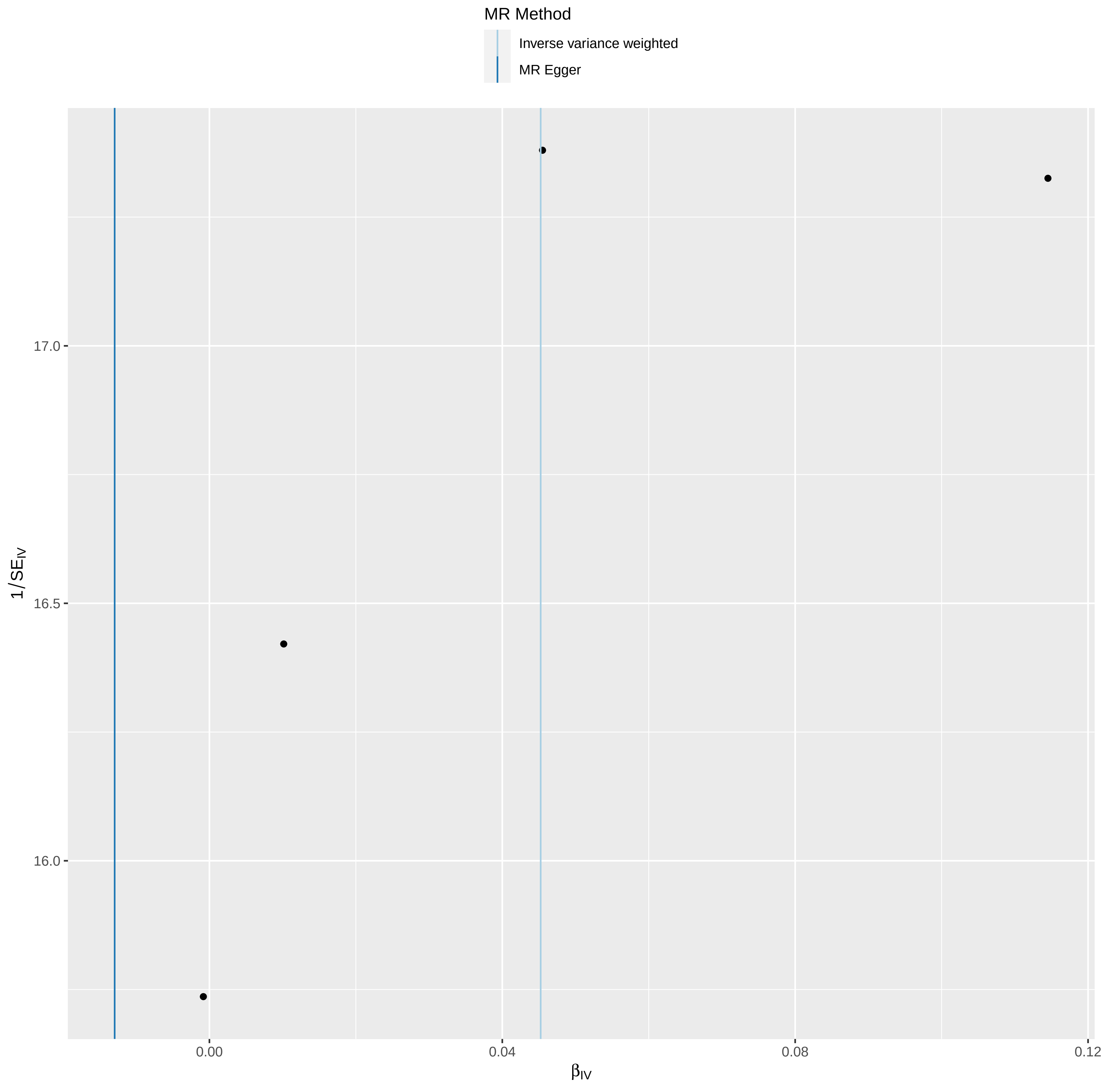

MR Method
Inverse variance weighted
MR Egger
17.0
16.5
16.0
1/SE_IV
0.00
0.04
0.08
0.12
β_IV

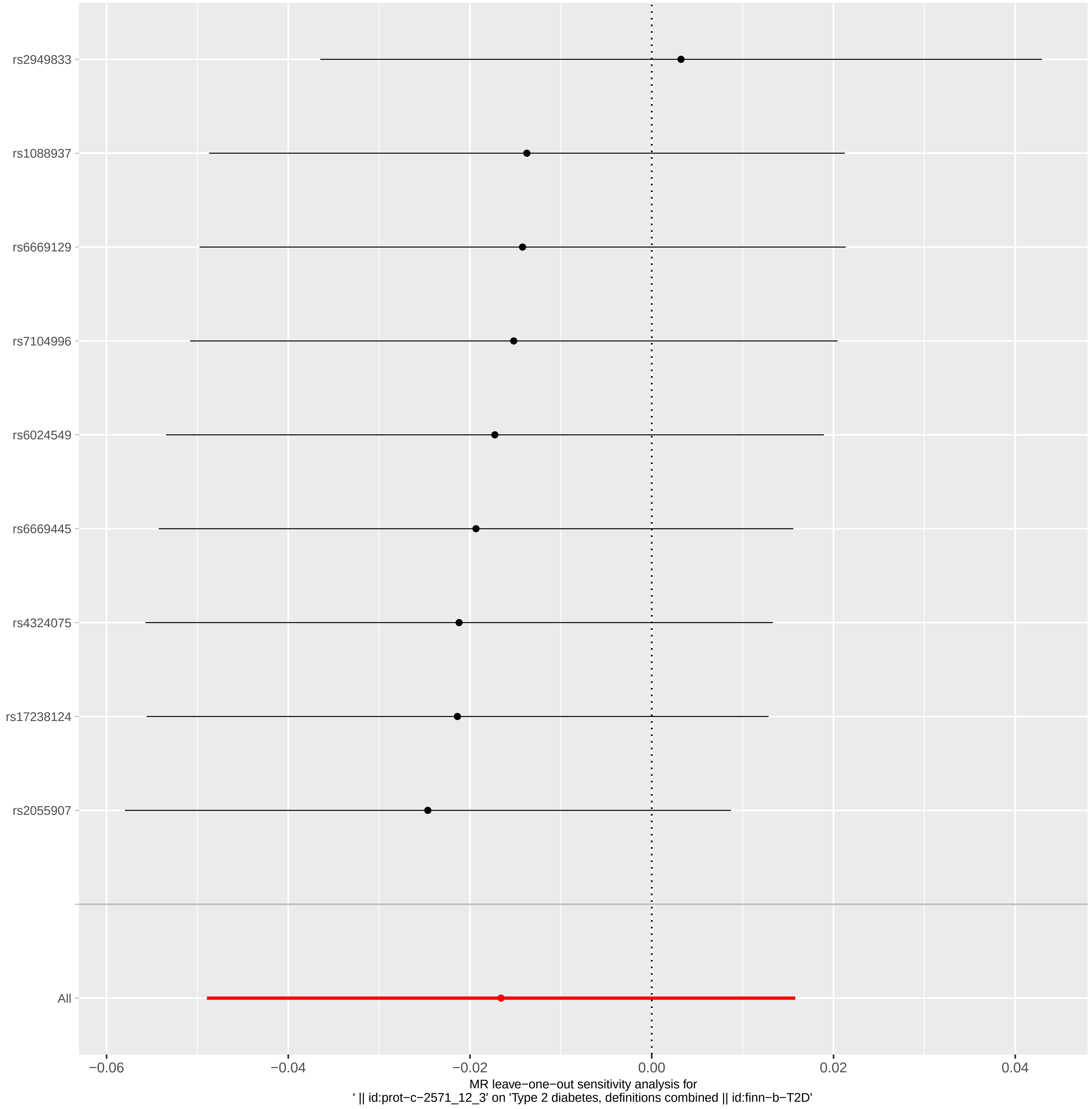
rs2949833
rs1088937
rs6669129
rs7104996
rs6024549
rs6669445
rs4324075
rs17238124
rs2055907
All
−0.06
−0.04
−0.02
0.00
0.02
0.04
MR leave−one−out sensitivity analysis for
' || id:prot−c−2571_12_3' on 'Type 2 diabetes, definitions combined || id:finn−b−T2D'

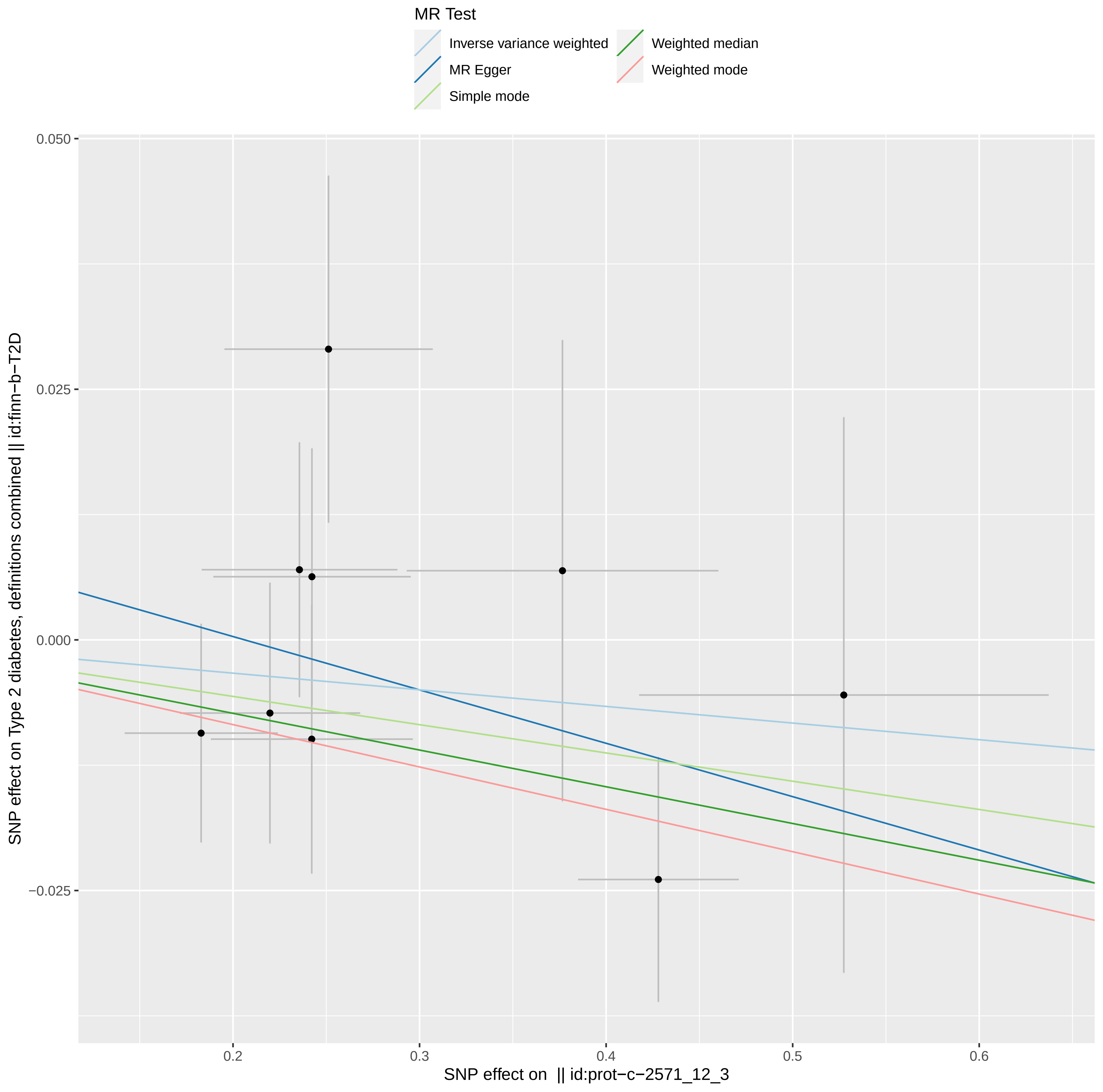

MR Test
Inverse variance weighted
MR Egger
Simple mode
Weighted median
Weighted mode
0.050
0.025
0.000
−0.025
0.2
0.3
0.4
0.5
0.6
SNP effect on || id:prot−c−2571_12_3
SNP effect on Type 2 diabetes, definitions combined || id:finn−b−T2D

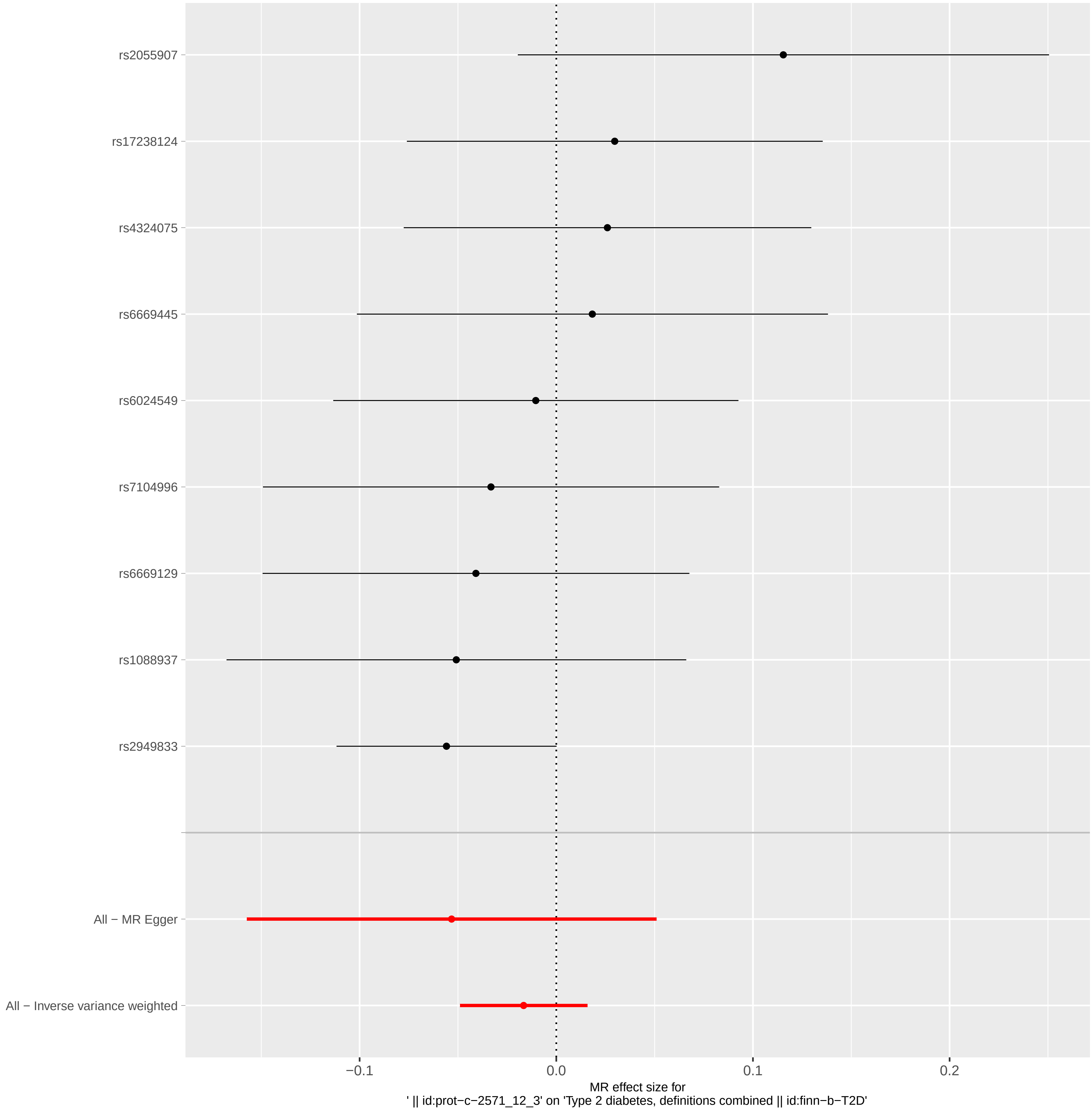

rs2055907
rs17238124
rs4324075
rs6669445
rs6024549
rs7104996
rs6669129
rs1088937
rs2949833
All − MR Egger
All − Inverse variance weighted
−0.1
0.0
0.1
0.2
MR effect size for
' || id:prot−c−2571_12_3' on 'Type 2 diabetes, definitions combined || id:finn−b−T2D'

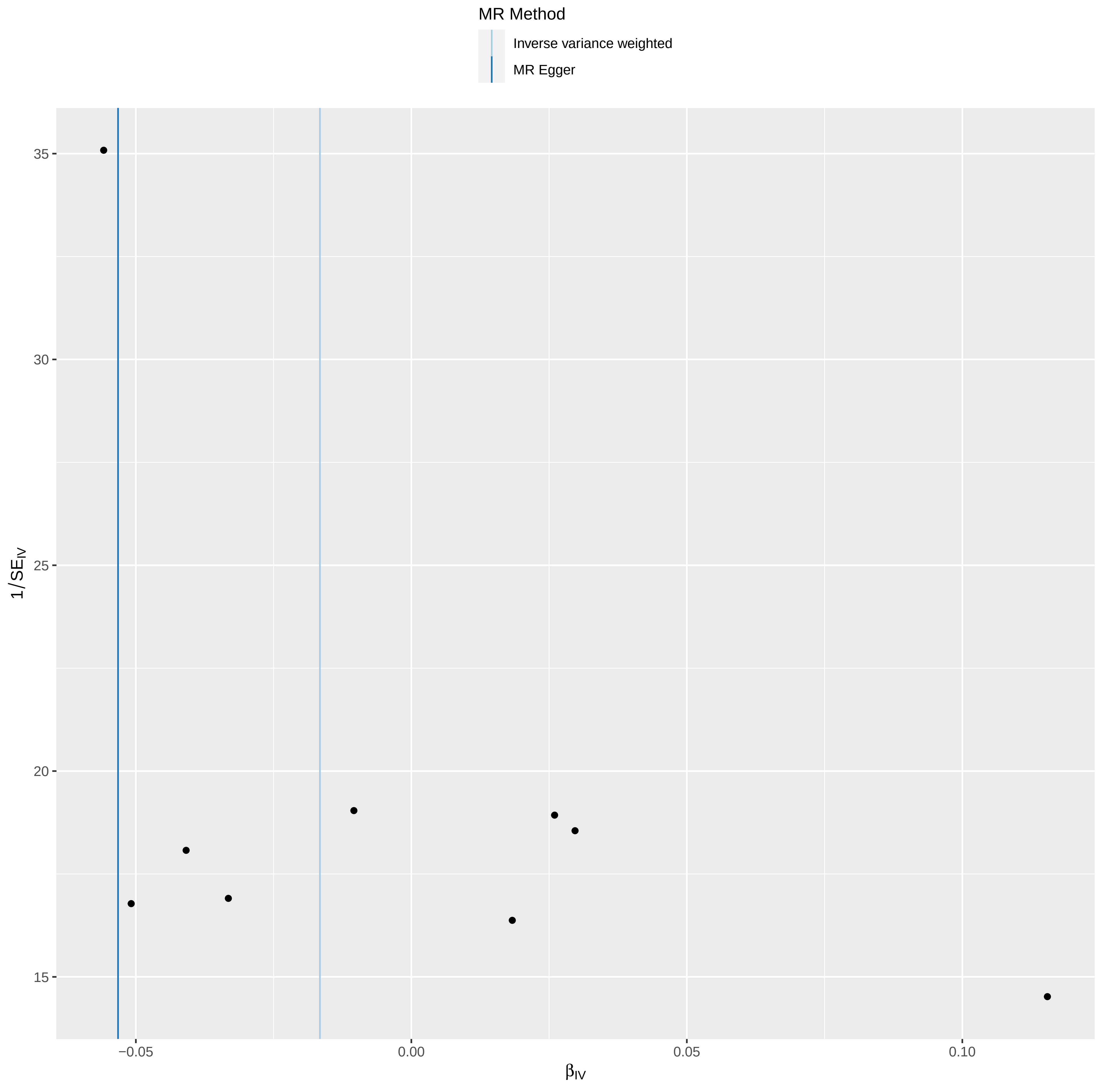
MR Method
Inverse variance weighted
MR Egger
35
30
25
20
15
1/SE$_{IV}$
−0.05
0.00
0.05
0.10
β$_{IV}$

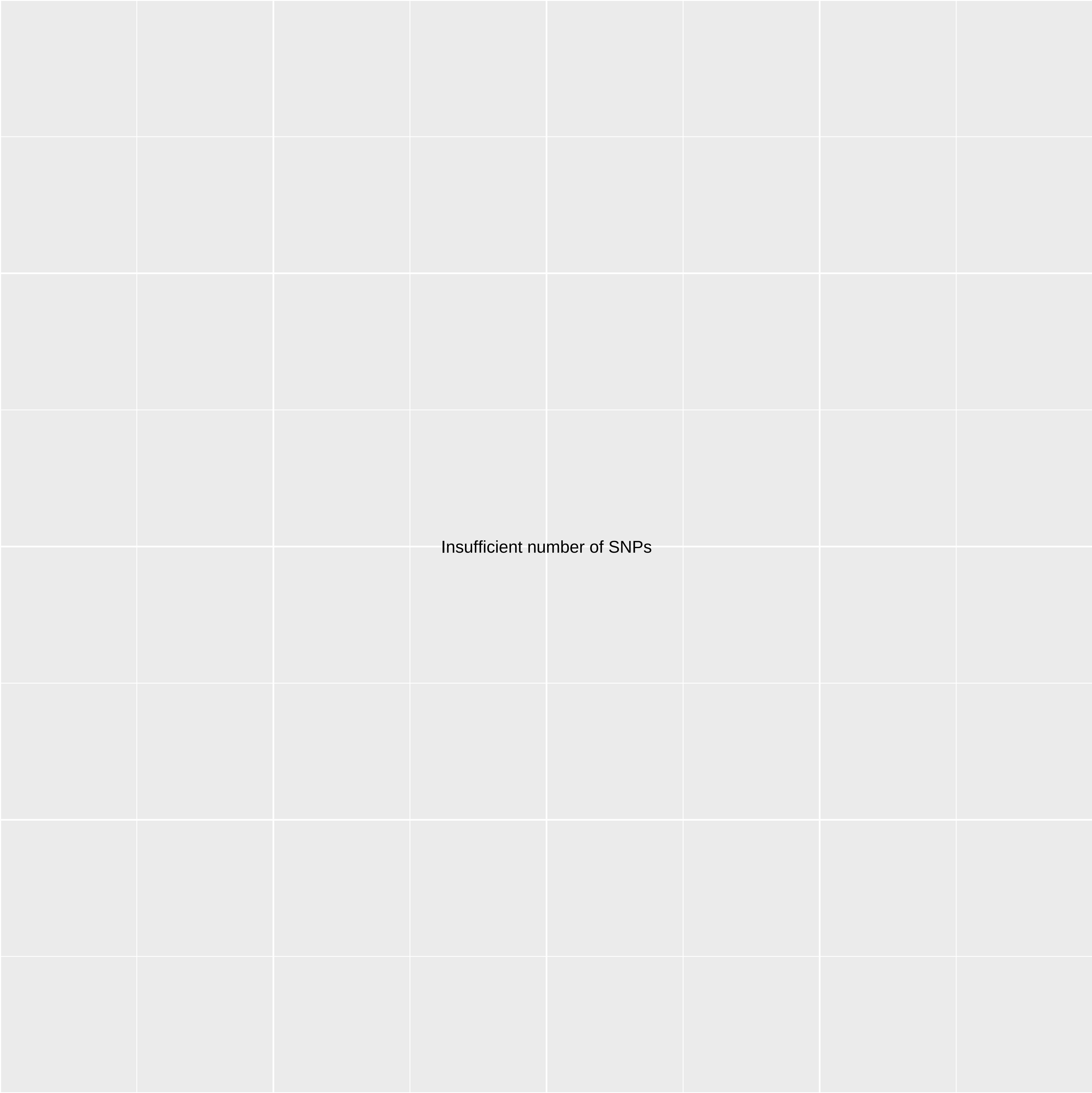
Insufficient number of SNPs

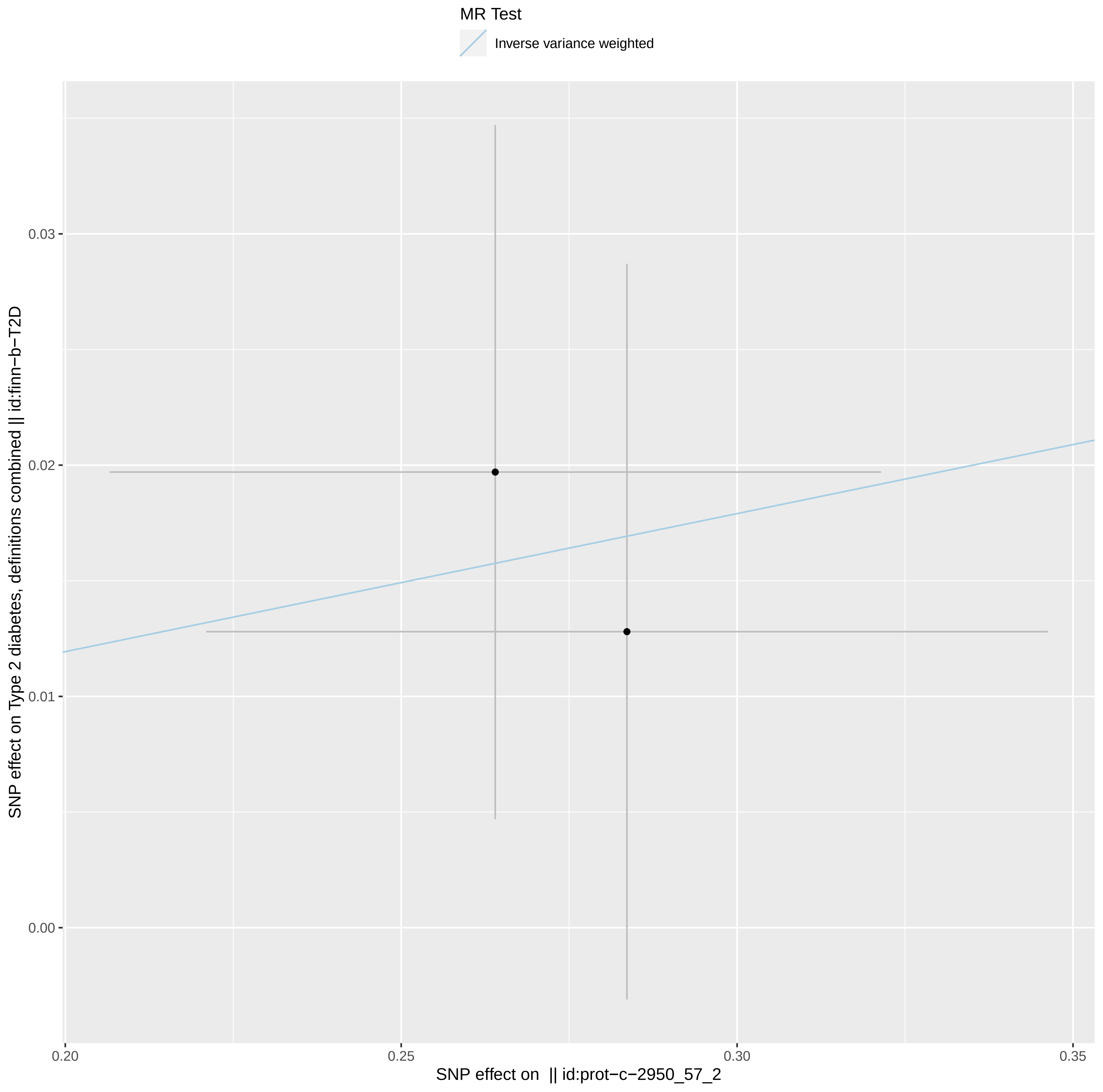
MR Test
Inverse variance weighted
0.03
0.02
0.01
0.00
SNP effect on Type 2 diabetes, definitions combined || id:finn-b-T2D
0.20
0.25
0.30
0.35
SNP effect on || id:prot-c-2950_57_2

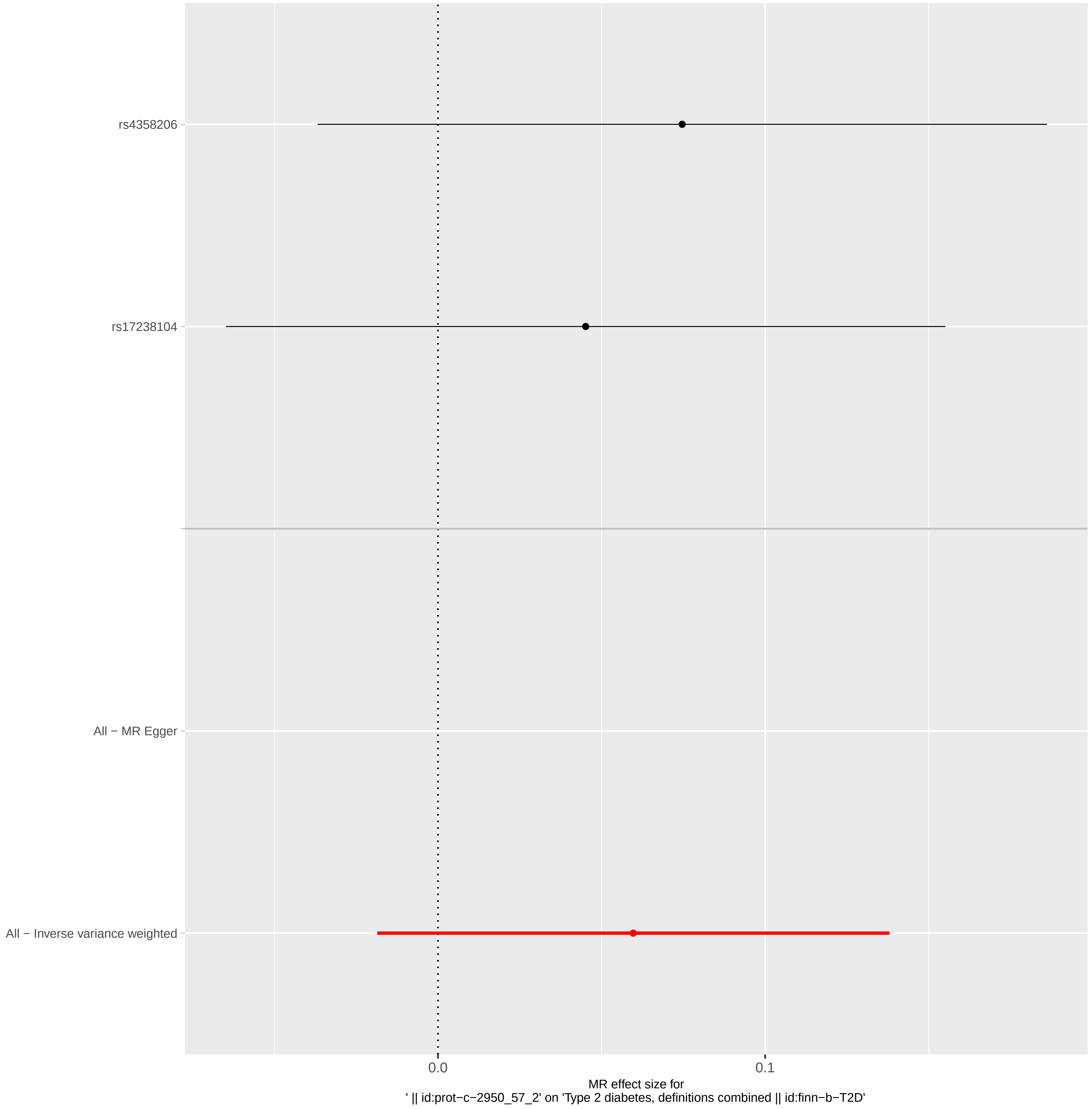
rs4358206
rs17238104
All − MR Egger
All − Inverse variance weighted
0.0
0.1
MR effect size for
' || id:prot−c−2950_57_2' on 'Type 2 diabetes, definitions combined || id:finn−b−T2D'

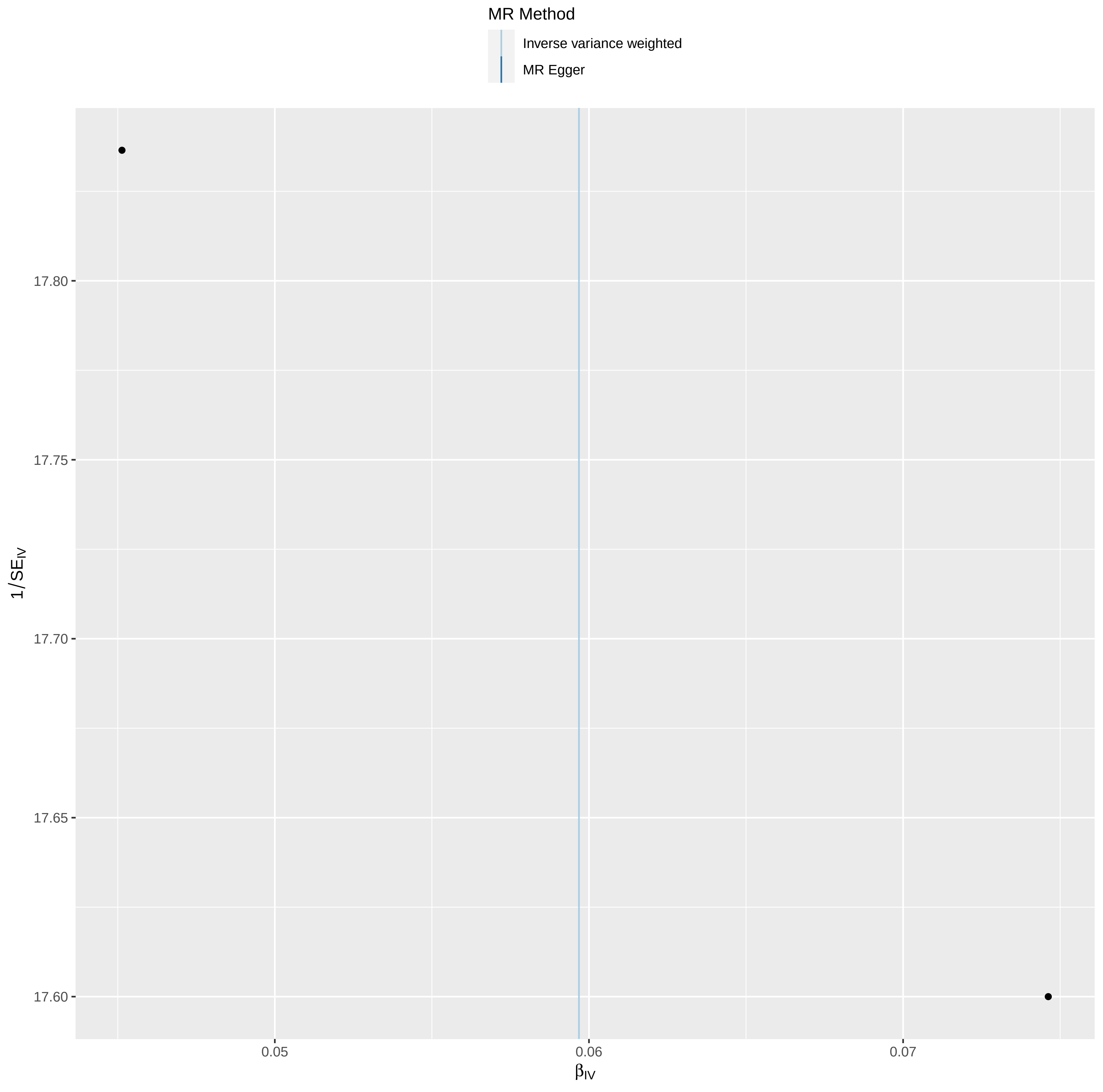
MR Method
Inverse variance weighted
MR Egger
17.80
17.75
17.70
17.65
17.60
1/SE_IV
0.05
0.06
0.07
β_IV

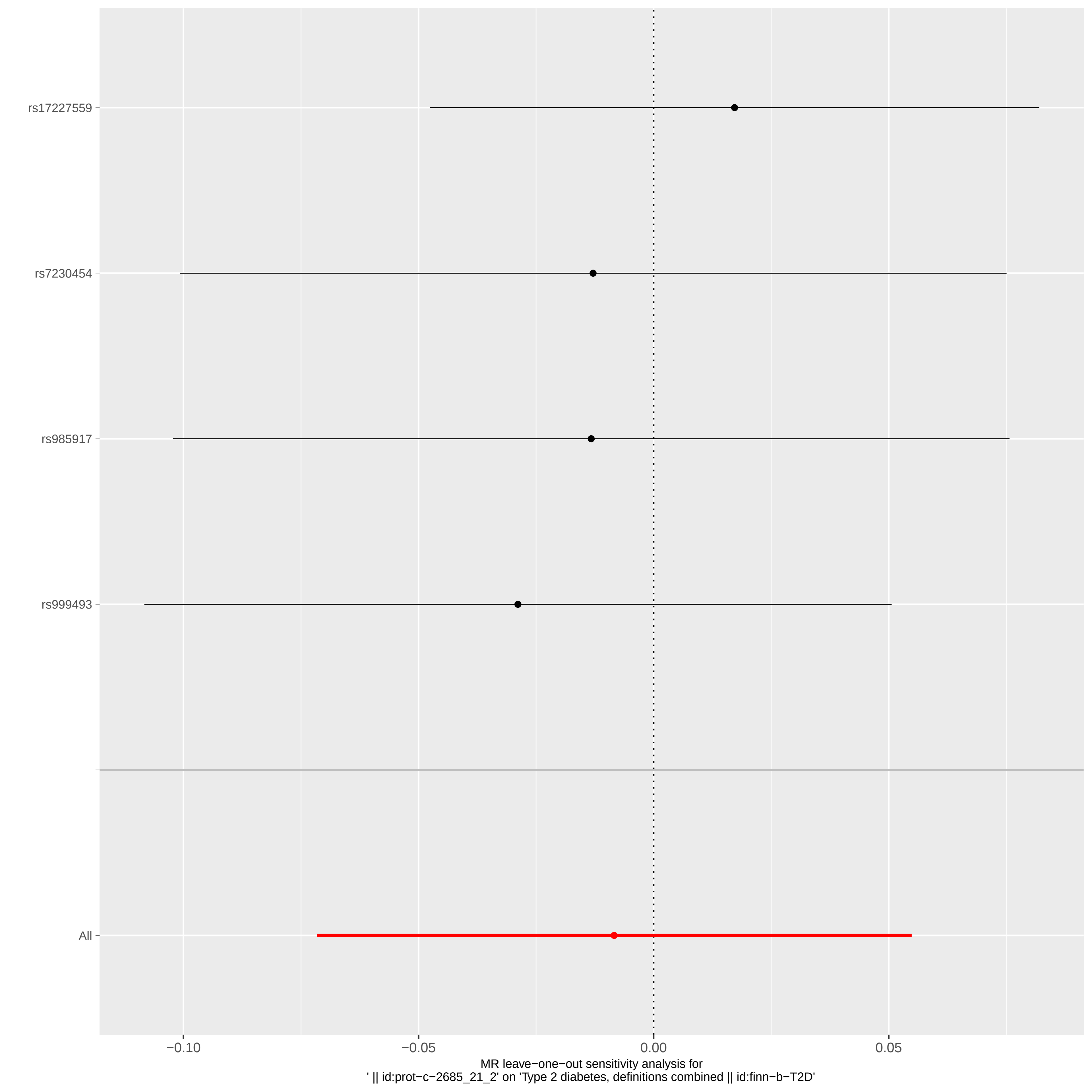
rs17227559
rs7230454
rs985917
rs999493
All
−0.10
−0.05
0.00
0.05
MR leave−one−out sensitivity analysis for
' || id:prot−c−2685_21_2' on 'Type 2 diabetes, definitions combined || id:finn−b−T2D'

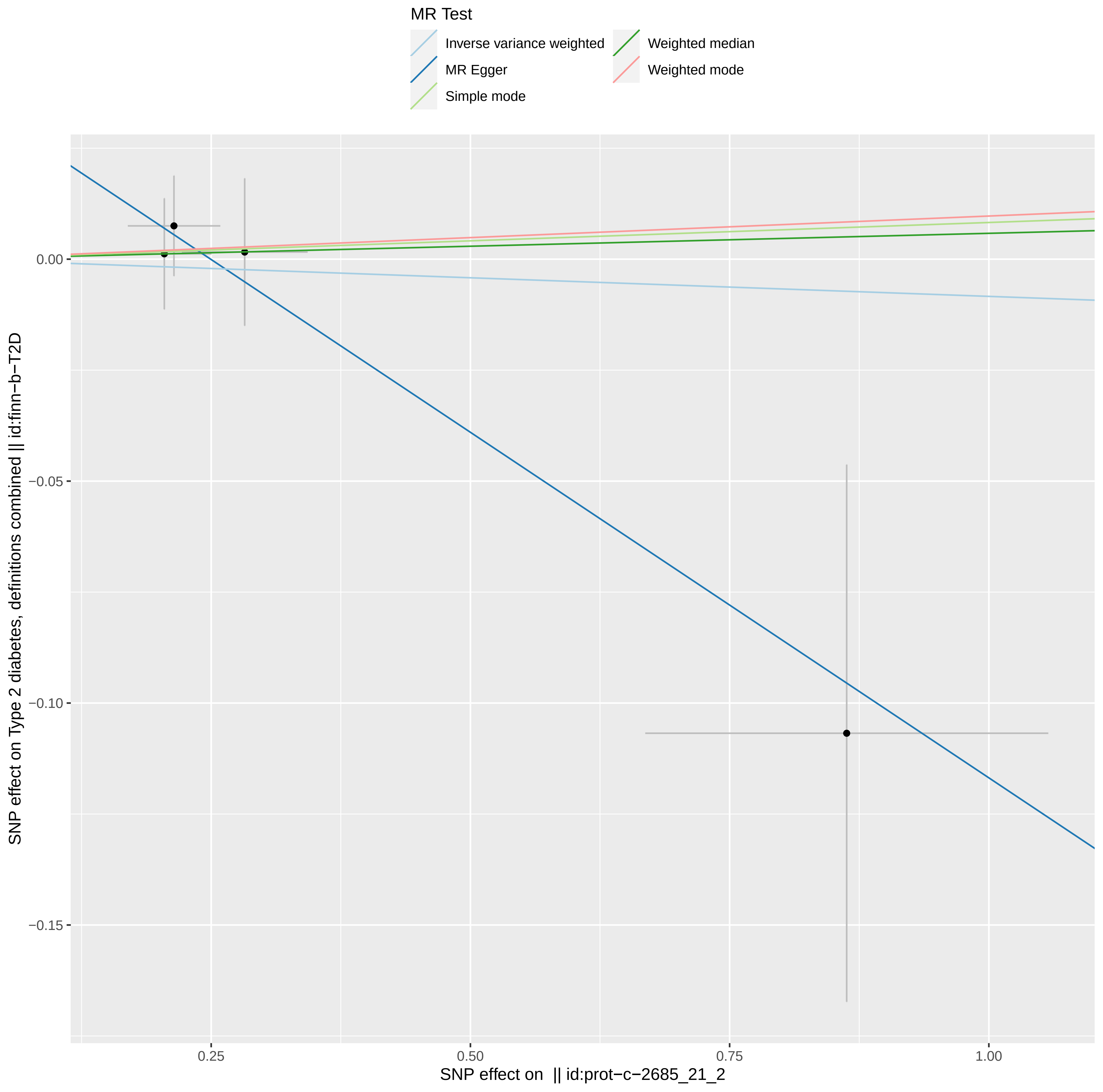
MR Test
Inverse variance weighted
MR Egger
Simple mode
Weighted median
Weighted mode
0.00
−0.05
−0.10
−0.15
0.25
0.50
0.75
1.00
SNP effect on Type 2 diabetes, definitions combined || id:finn−b−T2D
SNP effect on || id:prot−c−2685_21_2

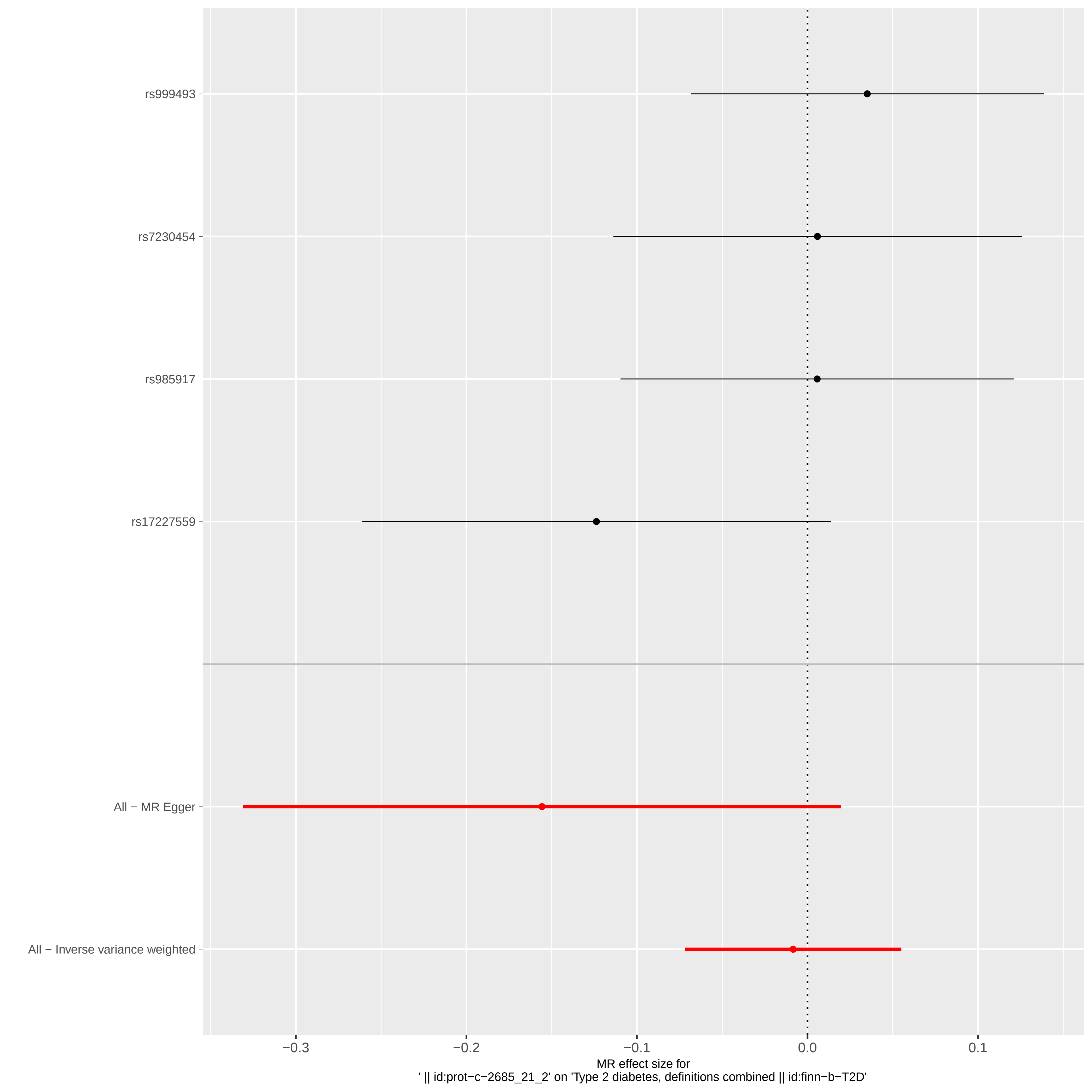
rs999493
rs7230454
rs985917
rs17227559
All − MR Egger
All − Inverse variance weighted
−0.3
−0.2
−0.1
0.0
0.1
MR effect size for
' || id:prot−c−2685_21_2' on 'Type 2 diabetes, definitions combined || id:finn−b−T2D'

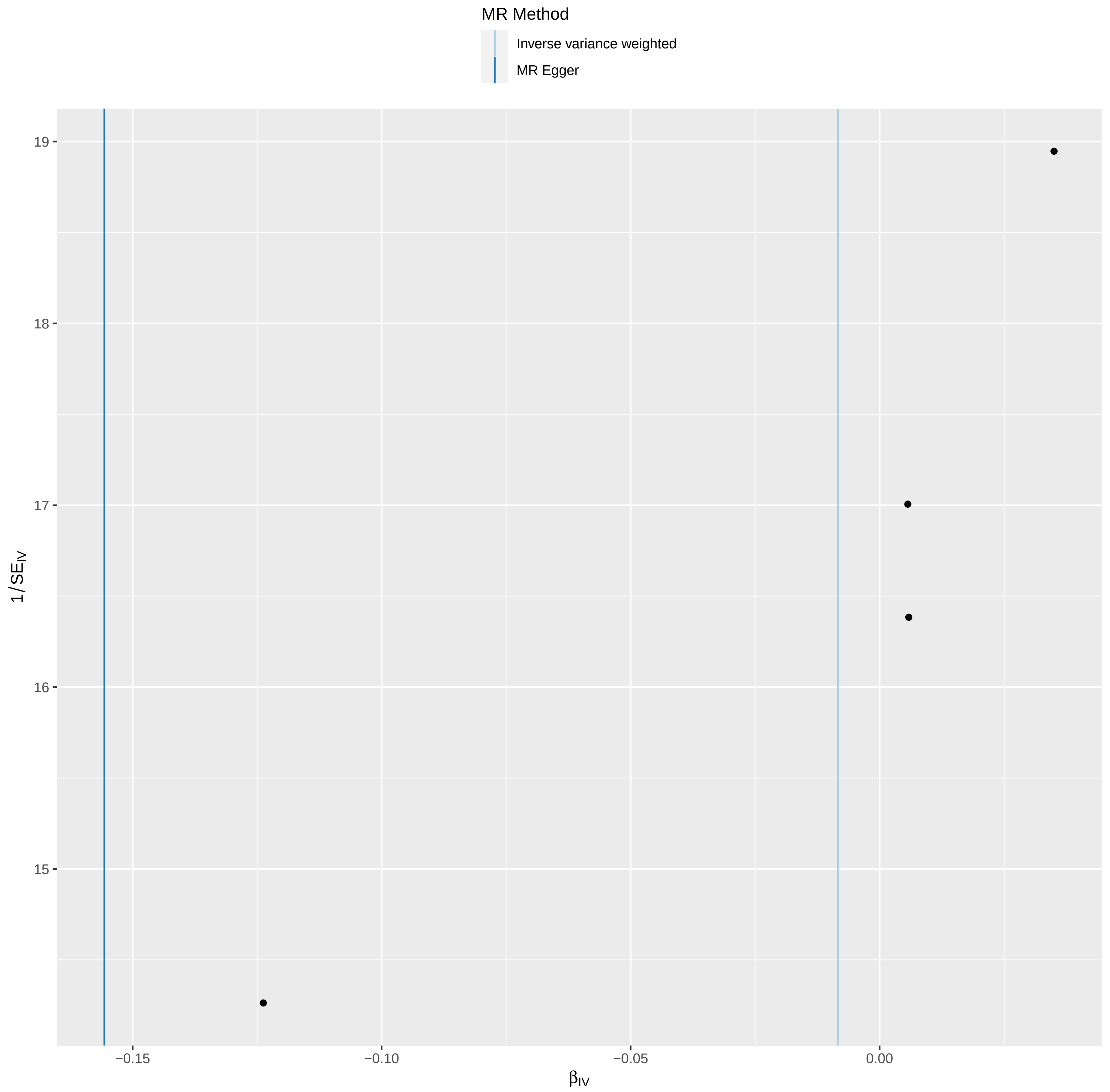
MR Method
Inverse variance weighted
MR Egger
19
18
17
16
15
1/SE_IV
−0.15
−0.10
−0.05
0.00
β_IV

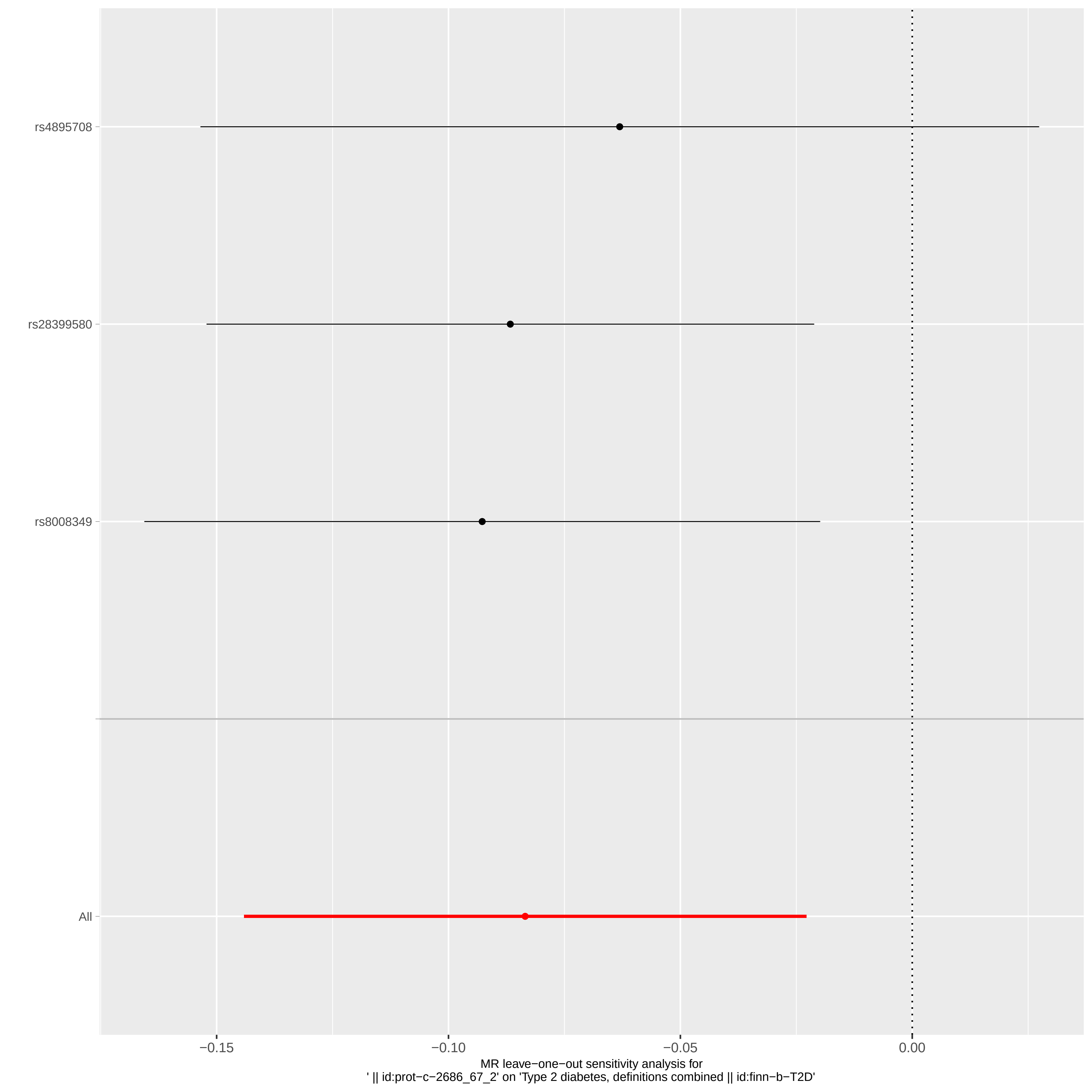
rs4895708
rs28399580
rs8008349
All
−0.15
−0.10
−0.05
0.00
MR leave−one−out sensitivity analysis for
' || id:prot−c−2686_67_2' on 'Type 2 diabetes, definitions combined || id:finn−b−T2D'

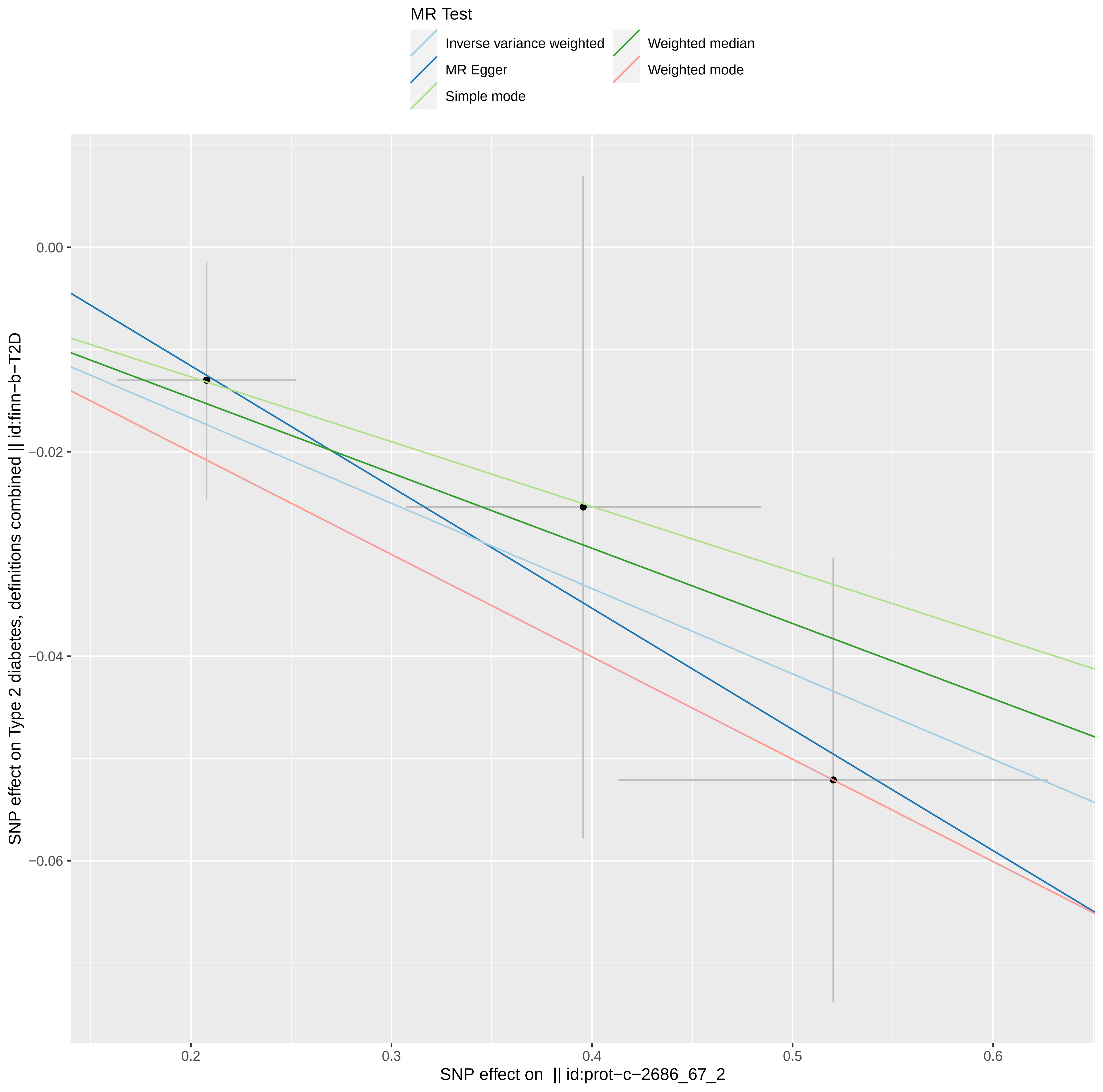

MR Test
Inverse variance weighted
MR Egger
Simple mode
Weighted median
Weighted mode
0.00
−0.02
−0.04
−0.06
0.2
0.3
0.4
0.5
0.6
SNP effect on Type 2 diabetes, definitions combined || id:finn−b−T2D
SNP effect on || id:prot−c−2686_67_2

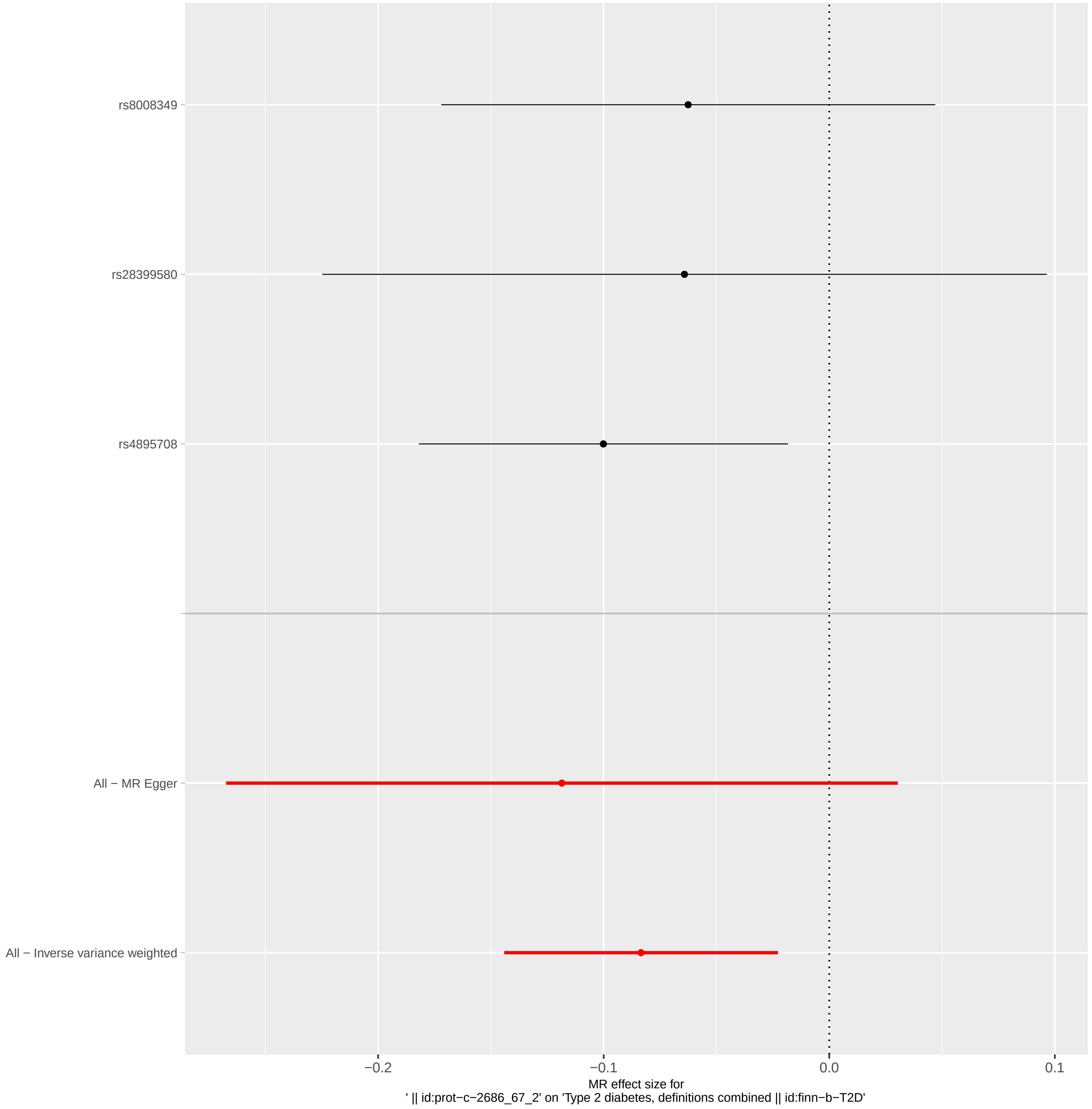
rs8008349
rs28399580
rs4895708
All − MR Egger
All − Inverse variance weighted
−0.2
−0.1
0.0
0.1
MR effect size for
' || id:prot−c−2686_67_2' on 'Type 2 diabetes, definitions combined || id:finn−b−T2D'

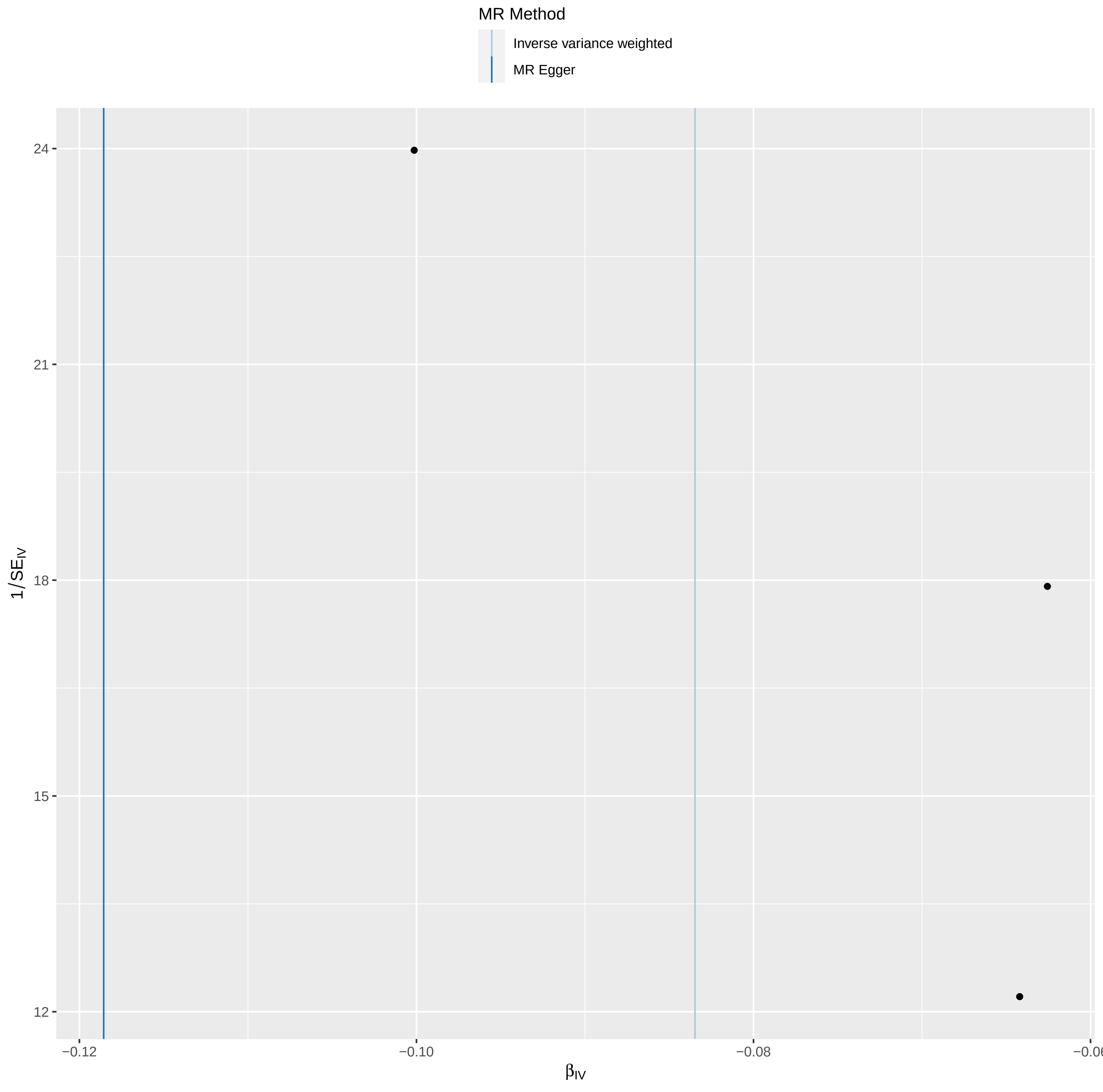

MR Method
Inverse variance weighted
MR Egger
24
21
18
15
12
1/SE$_{IV}$
−0.12
−0.10
−0.08
β$_{IV}$

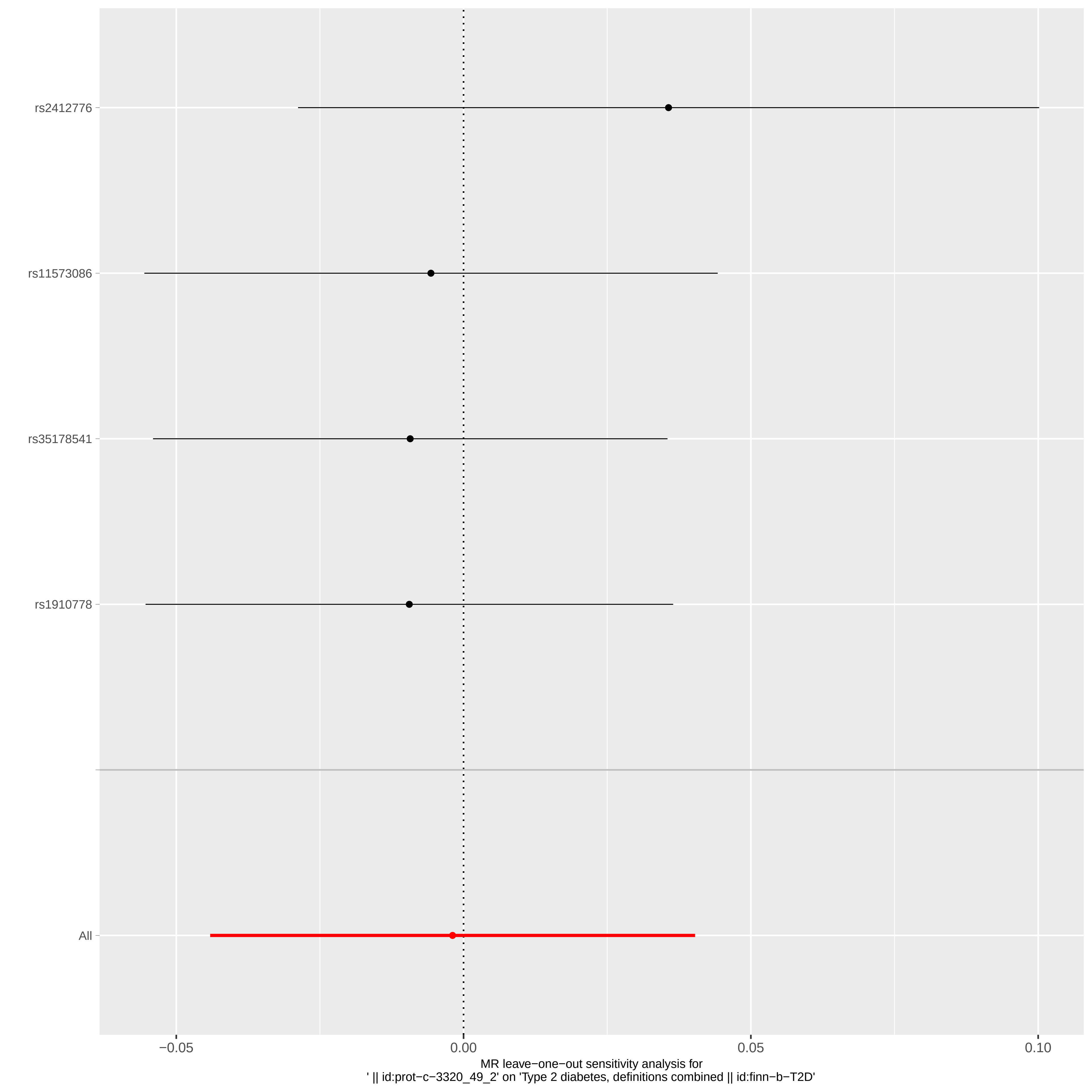
rs2412776
rs11573086
rs35178541
rs1910778
All
−0.05
0.00
0.05
0.10
MR leave−one−out sensitivity analysis for
' || id:prot−c−3320_49_2' on 'Type 2 diabetes, definitions combined || id:finn−b−T2D'

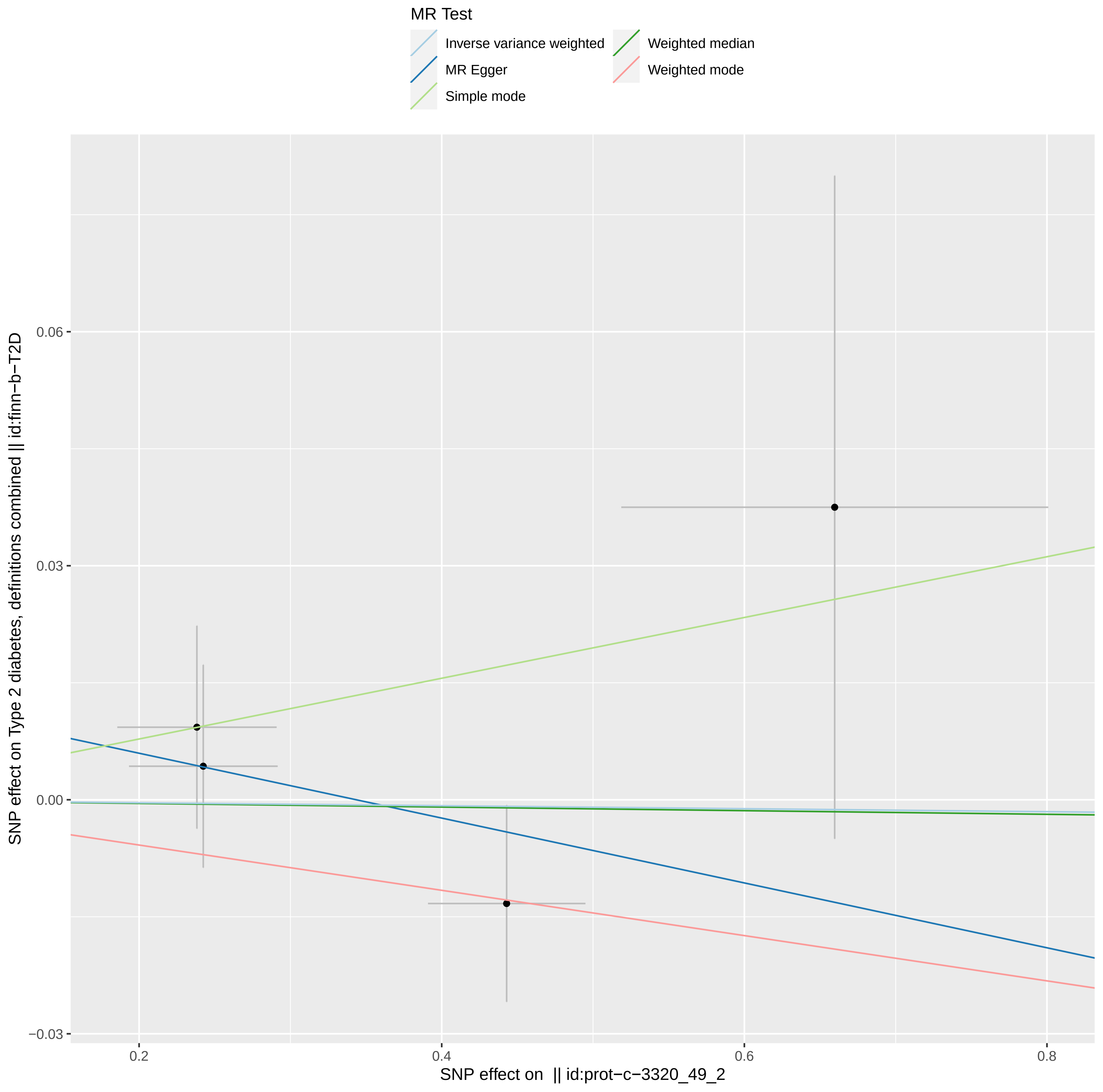
MR Test
Inverse variance weighted
MR Egger
Simple mode
Weighted median
Weighted mode
SNP effect on Type 2 diabetes, definitions combined || id:finn-b-T2D
0.06
0.03
0.00
−0.03
0.2
0.4
0.6
0.8
SNP effect on || id:prot-c-3320_49_2

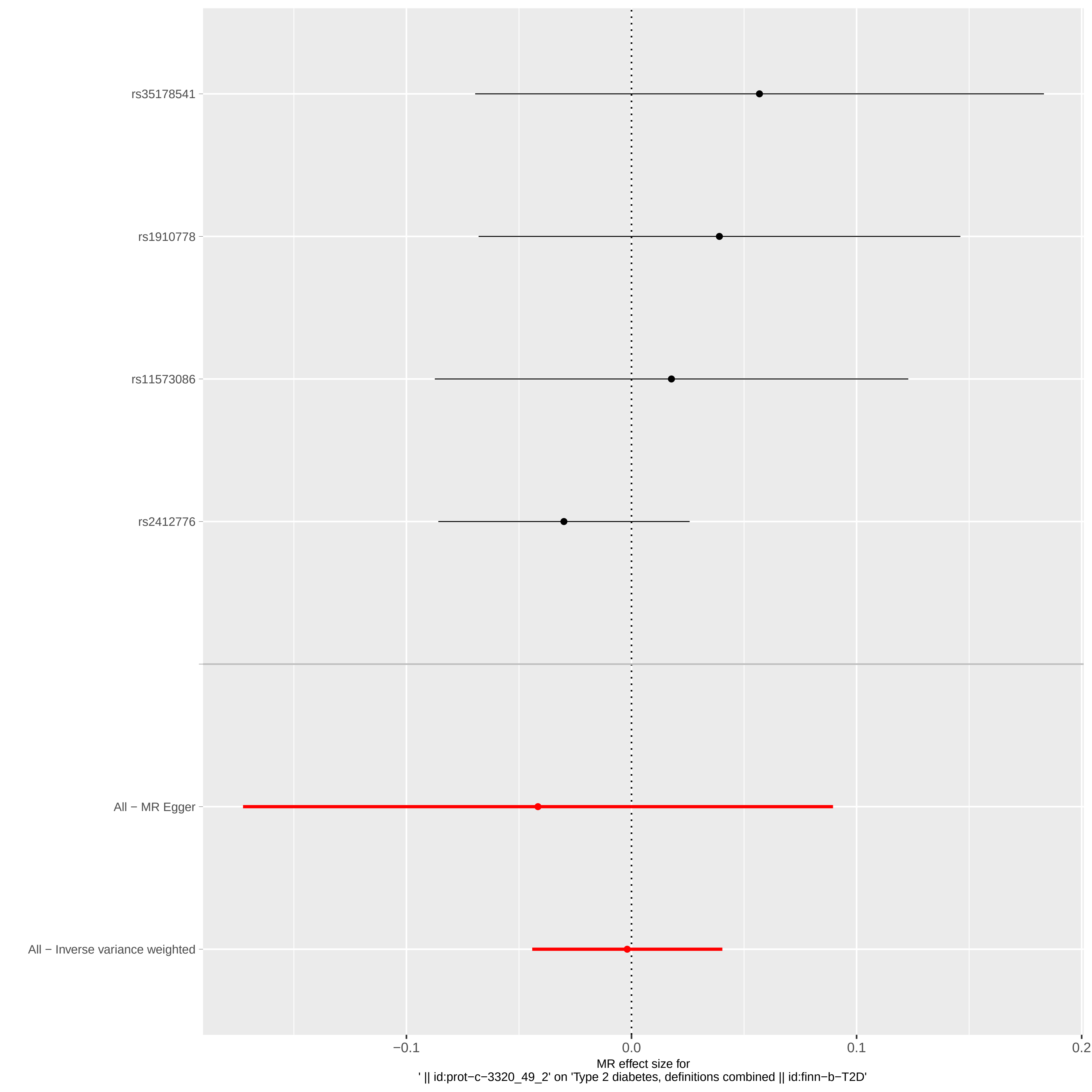
rs35178541
rs1910778
rs11573086
rs2412776
All − MR Egger
All − Inverse variance weighted
−0.1
0.0
0.1
0.2
MR effect size for
' || id:prot−c−3320_49_2' on 'Type 2 diabetes, definitions combined || id:finn−b−T2D'

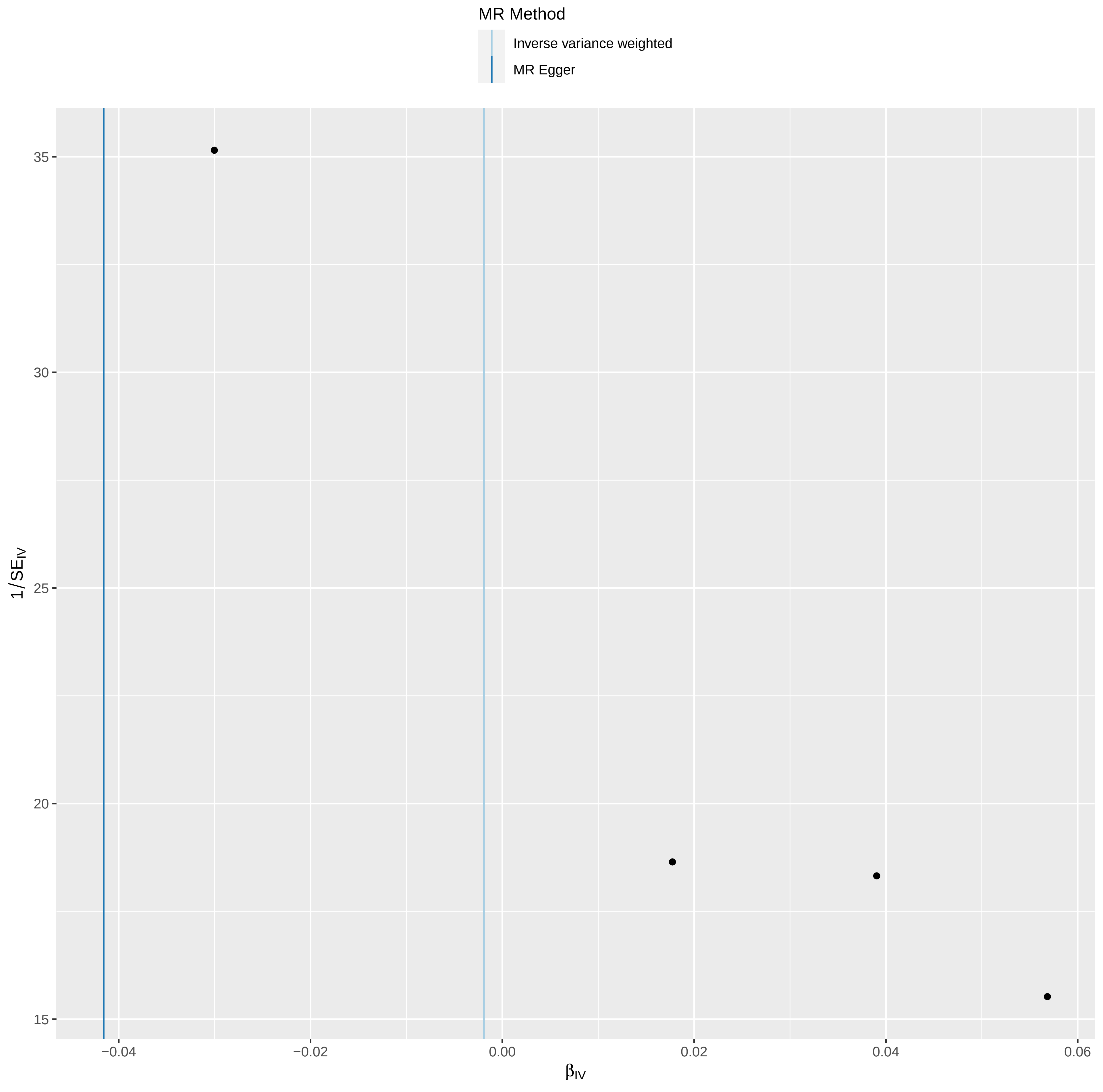

MR Method
Inverse variance weighted
MR Egger
35
30
25
20
15
1/SE$_{IV}$
−0.04
−0.02
0.00
0.02
0.04
0.06
β$_{IV}$

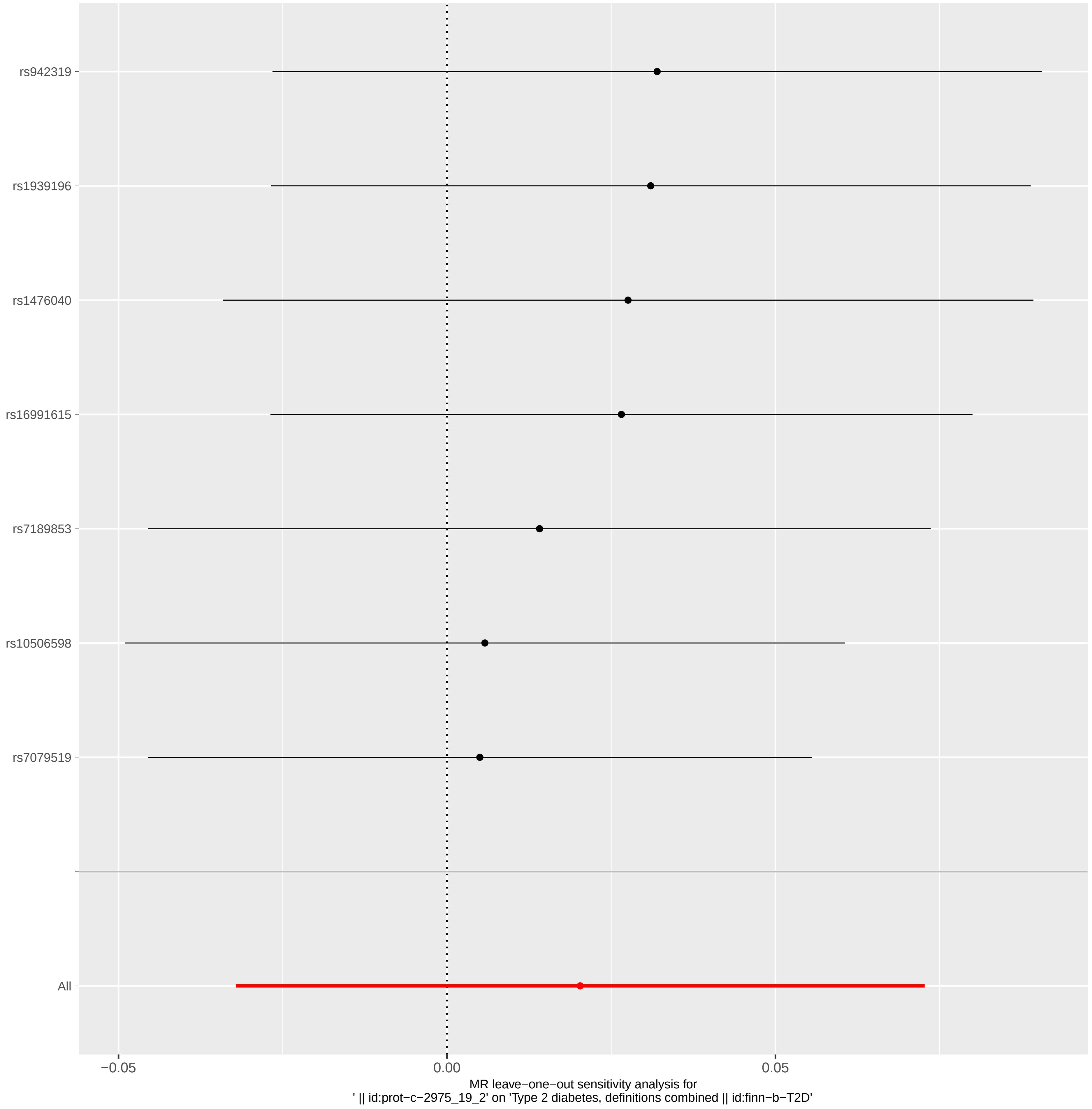
rs942319
rs1939196
rs1476040
rs16991615
rs7189853
rs10506598
rs7079519
All
−0.05
0.00
0.05
MR leave−one−out sensitivity analysis for
' || id:prot−c−2975_19_2' on 'Type 2 diabetes, definitions combined || id:finn−b−T2D'

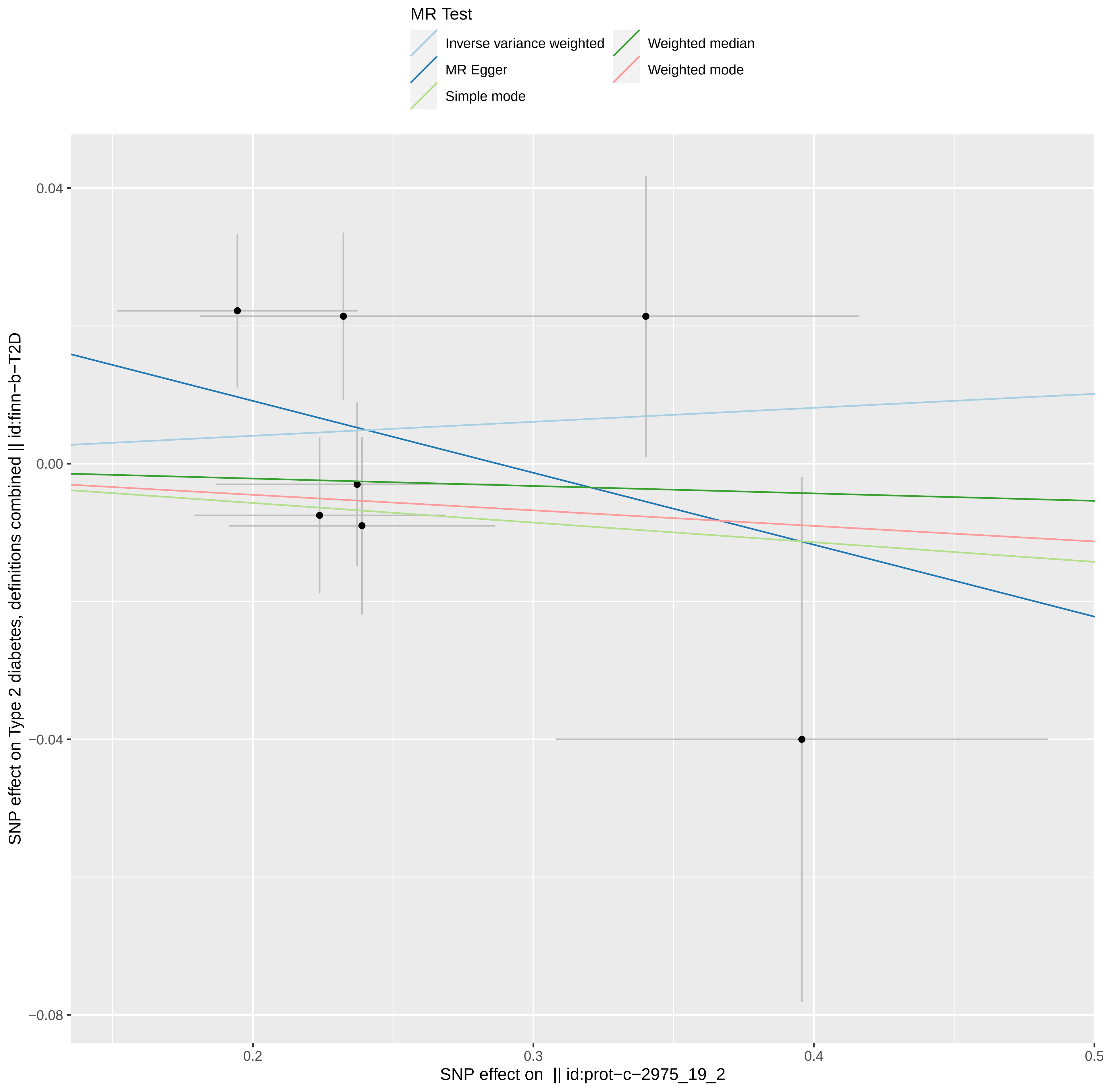

MR Test
Inverse variance weighted
MR Egger
Simple mode
Weighted median
Weighted mode
0.04
0.00
−0.04
−0.08
SNP effect on Type 2 diabetes, definitions combined || id:finn−b−T2D
0.2
0.3
0.4
0.5
SNP effect on || id:prot−c−2975_19_2

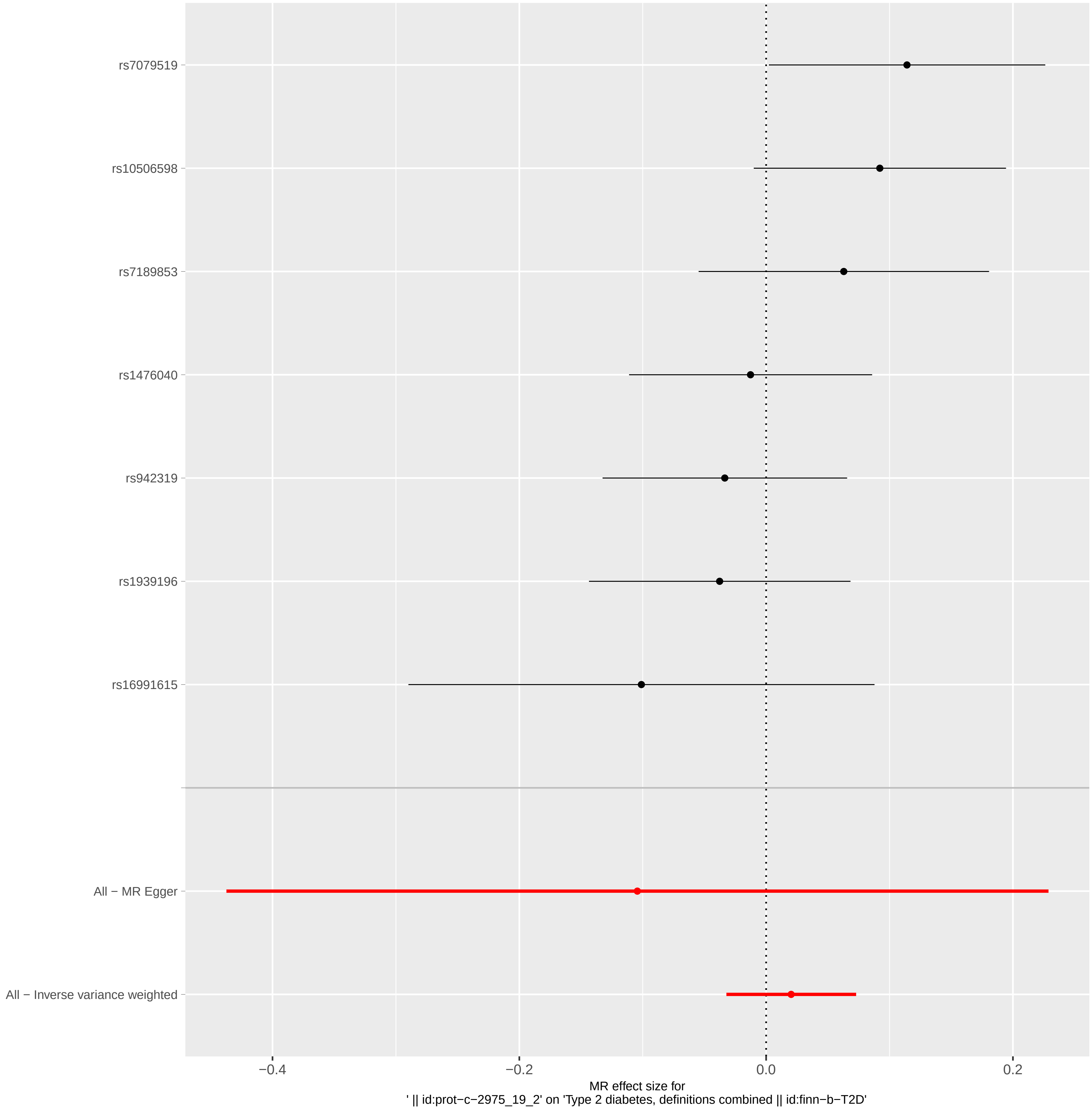

rs7079519
rs10506598
rs7189853
rs1476040
rs942319
rs1939196
rs16991615
All − MR Egger
All − Inverse variance weighted
−0.4
−0.2
0.0
0.2
MR effect size for
' || id:prot−c−2975_19_2' on 'Type 2 diabetes, definitions combined || id:finn−b−T2D'

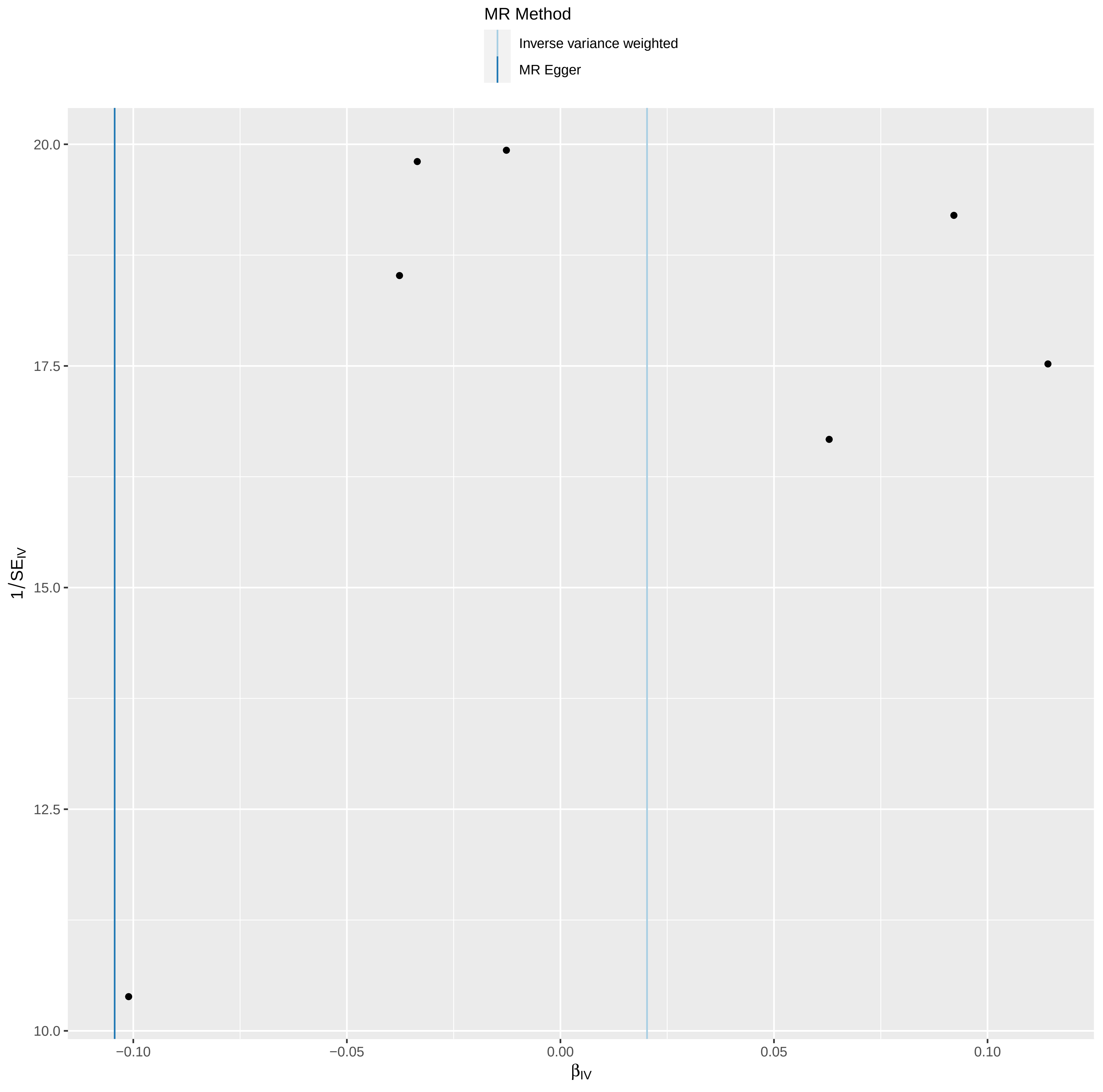

MR Method
Inverse variance weighted
MR Egger
20.0
17.5
15.0
12.5
10.0
1/SE_IV
−0.10
−0.05
0.00
0.05
0.10
β_IV

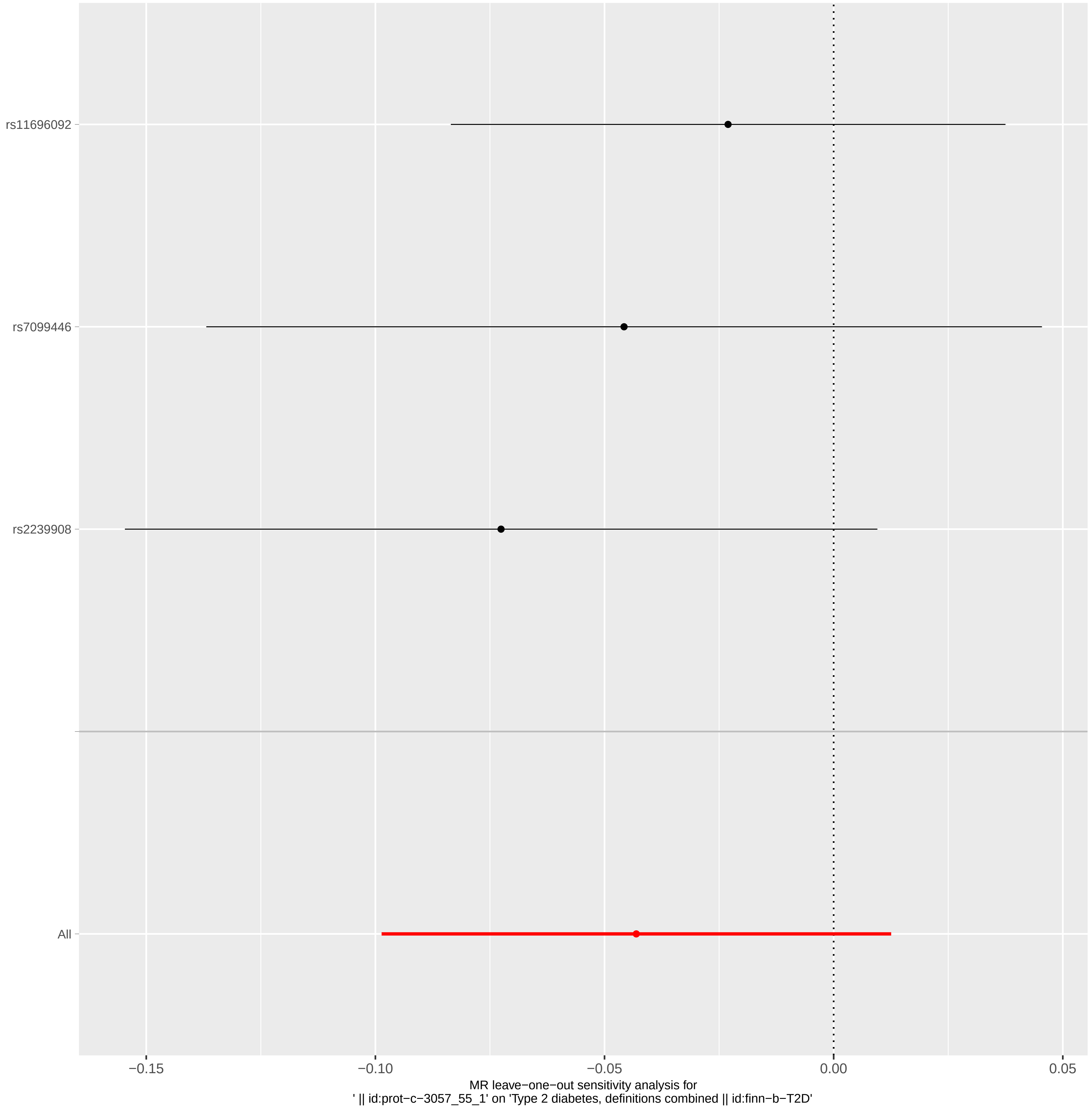

rs11696092
rs7099446
rs2239908
All
−0.15
−0.10
−0.05
0.00
0.05
MR leave−one−out sensitivity analysis for
' || id:prot−c−3057_55_1' on 'Type 2 diabetes, definitions combined || id:finn−b−T2D'

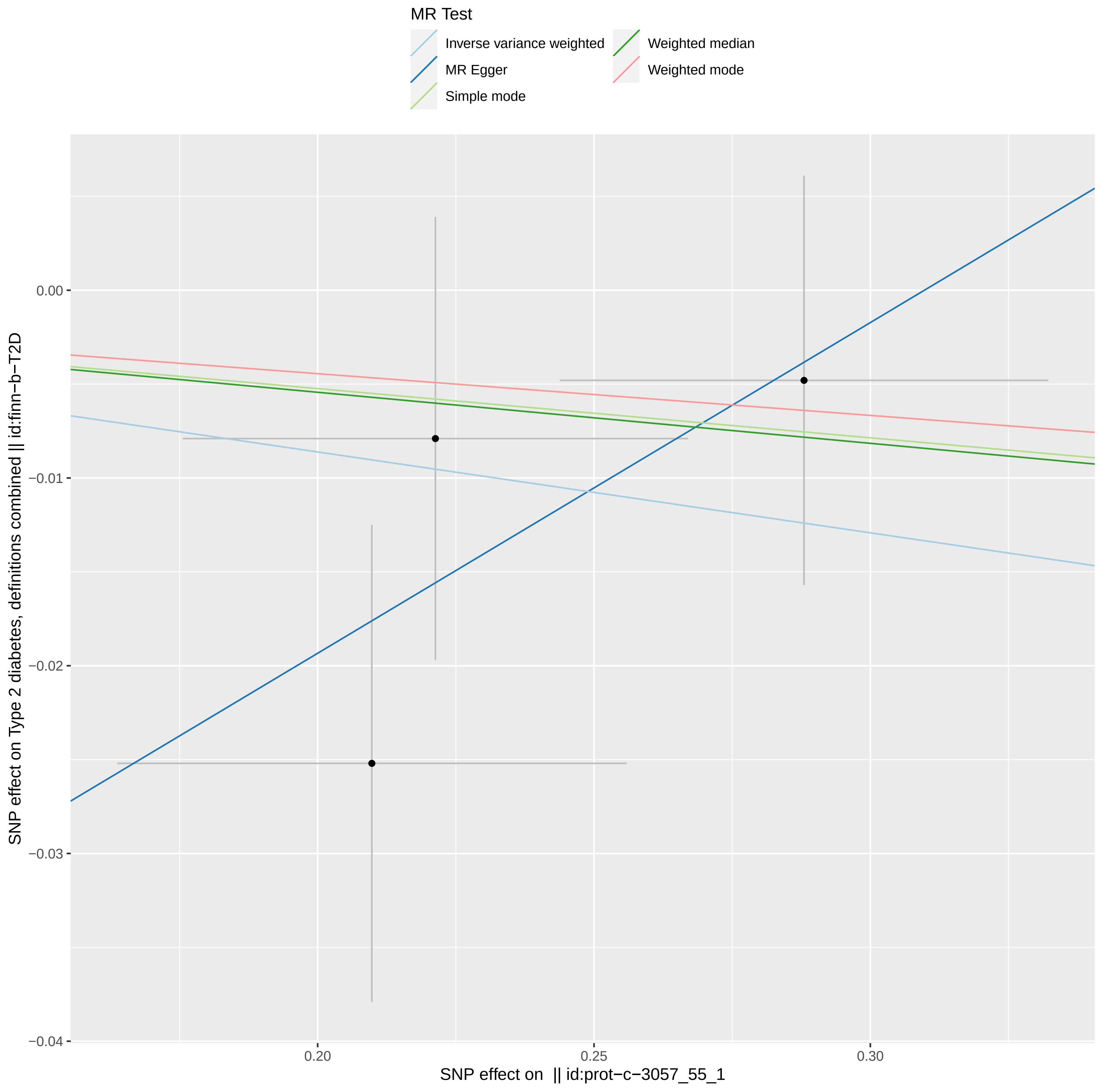

MR Test
Inverse variance weighted
MR Egger
Simple mode
Weighted median
Weighted mode
0.00
−0.01
−0.02
−0.03
−0.04
0.20
0.25
0.30
SNP effect on Type 2 diabetes, definitions combined || id:finn−b−T2D
SNP effect on || id:prot−c−3057_55_1

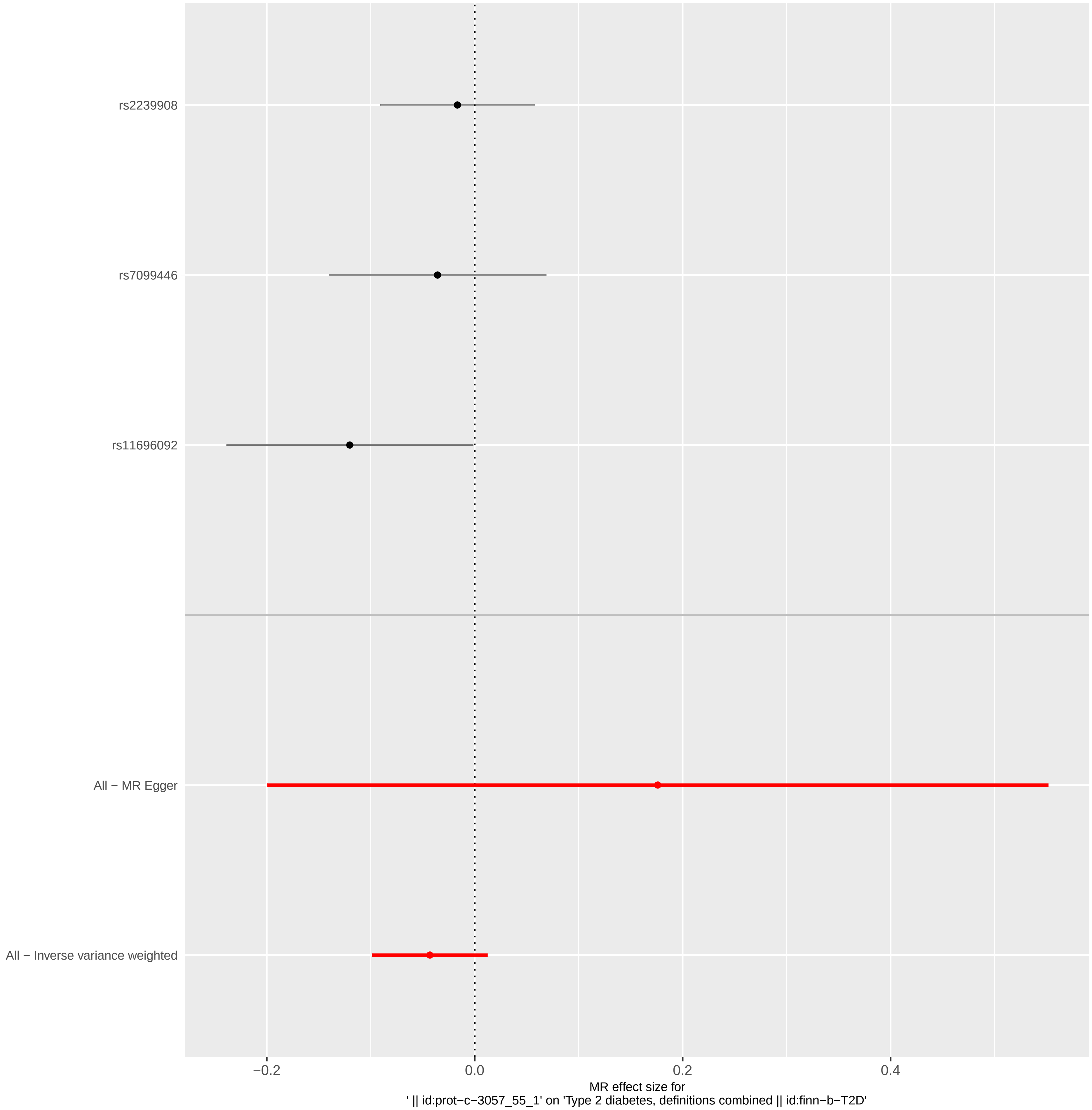
rs2239908
rs7099446
rs11696092
All − MR Egger
All − Inverse variance weighted
−0.2
0.0
0.2
0.4
MR effect size for
' || id:prot−c−3057_55_1' on 'Type 2 diabetes, definitions combined || id:finn−b−T2D'

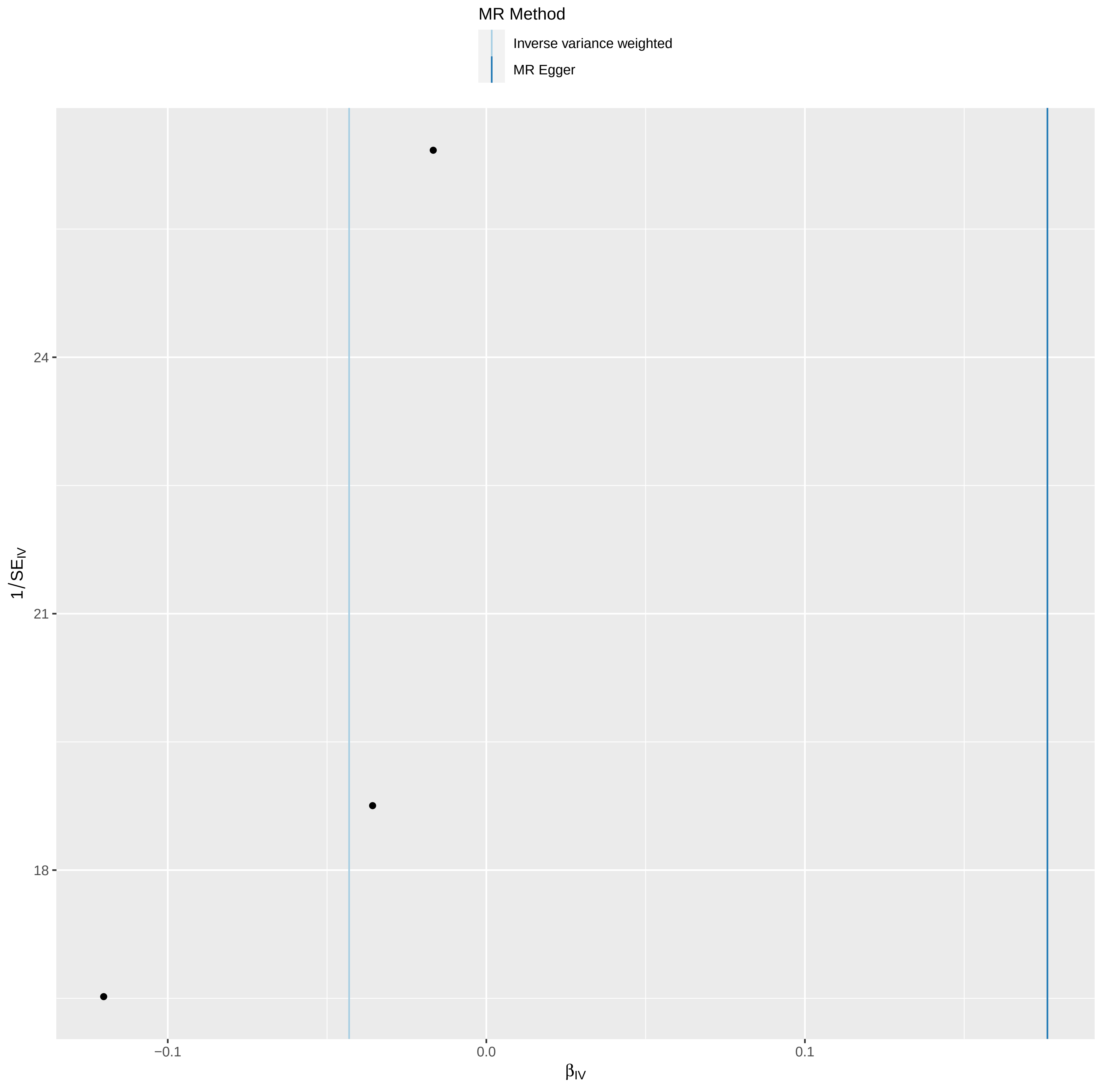

MR Method
Inverse variance weighted
MR Egger
24
21
18
1/SE$_{IV}$
−0.1
0.0
0.1
β$_{IV}$

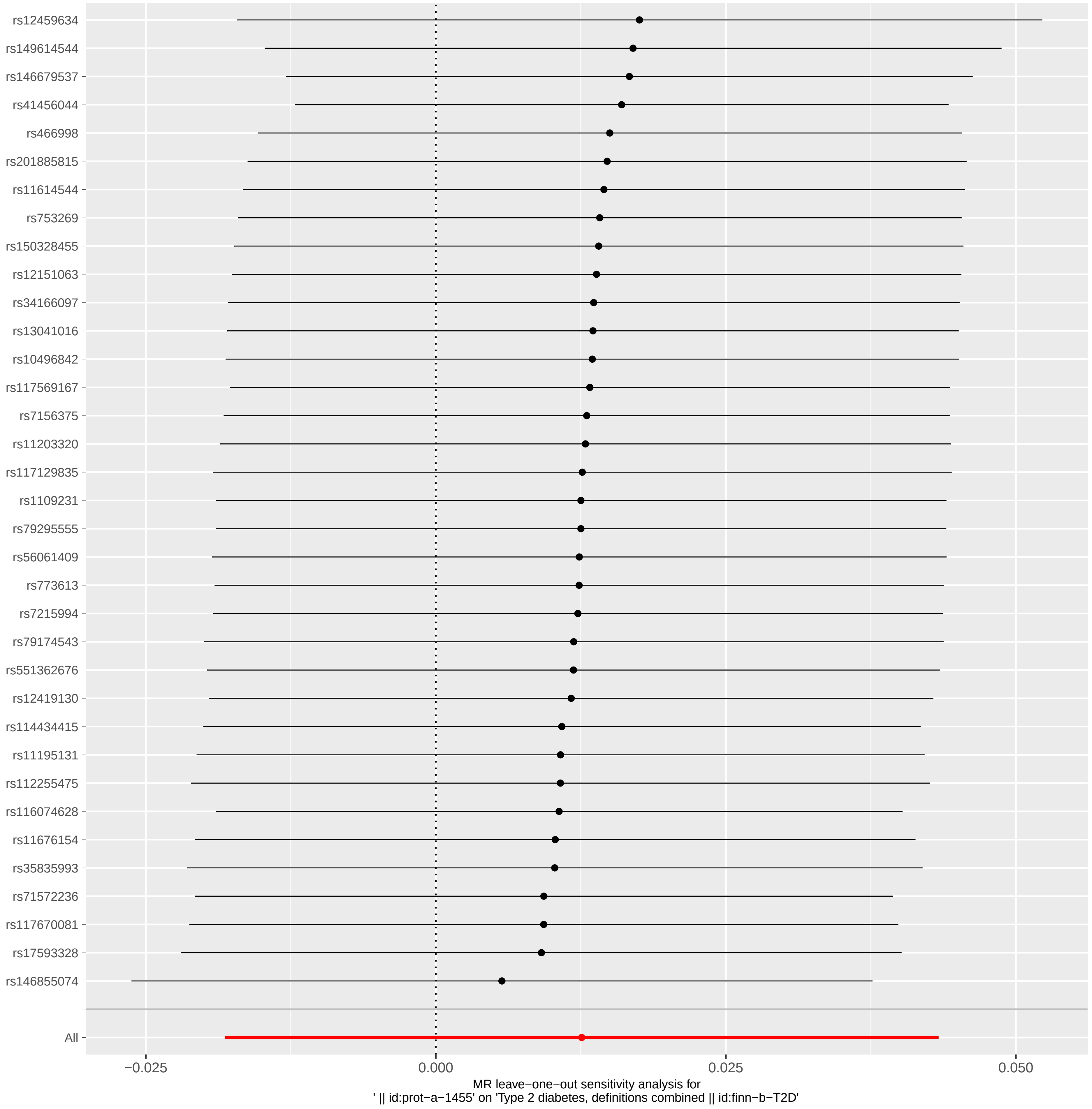

rs12459634
rs149614544
rs146679537
rs41456044
rs466998
rs201885815
rs11614544
rs753269
rs150328455
rs12151063
rs34166097
rs13041016
rs10496842
rs117569167
rs7156375
rs11203320
rs117129835
rs1109231
rs79295555
rs56061409
rs773613
rs7215994
rs79174543
rs551362676
rs12419130
rs114434415
rs11195131
rs112255475
rs116074628
rs11676154
rs35835993
rs71572236
rs117670081
rs17593328
rs146855074
All
−0.025
0.000
0.025
0.050
MR leave−one−out sensitivity analysis for
' || id:prot−a−1455' on 'Type 2 diabetes, definitions combined || id:finn−b−T2D'

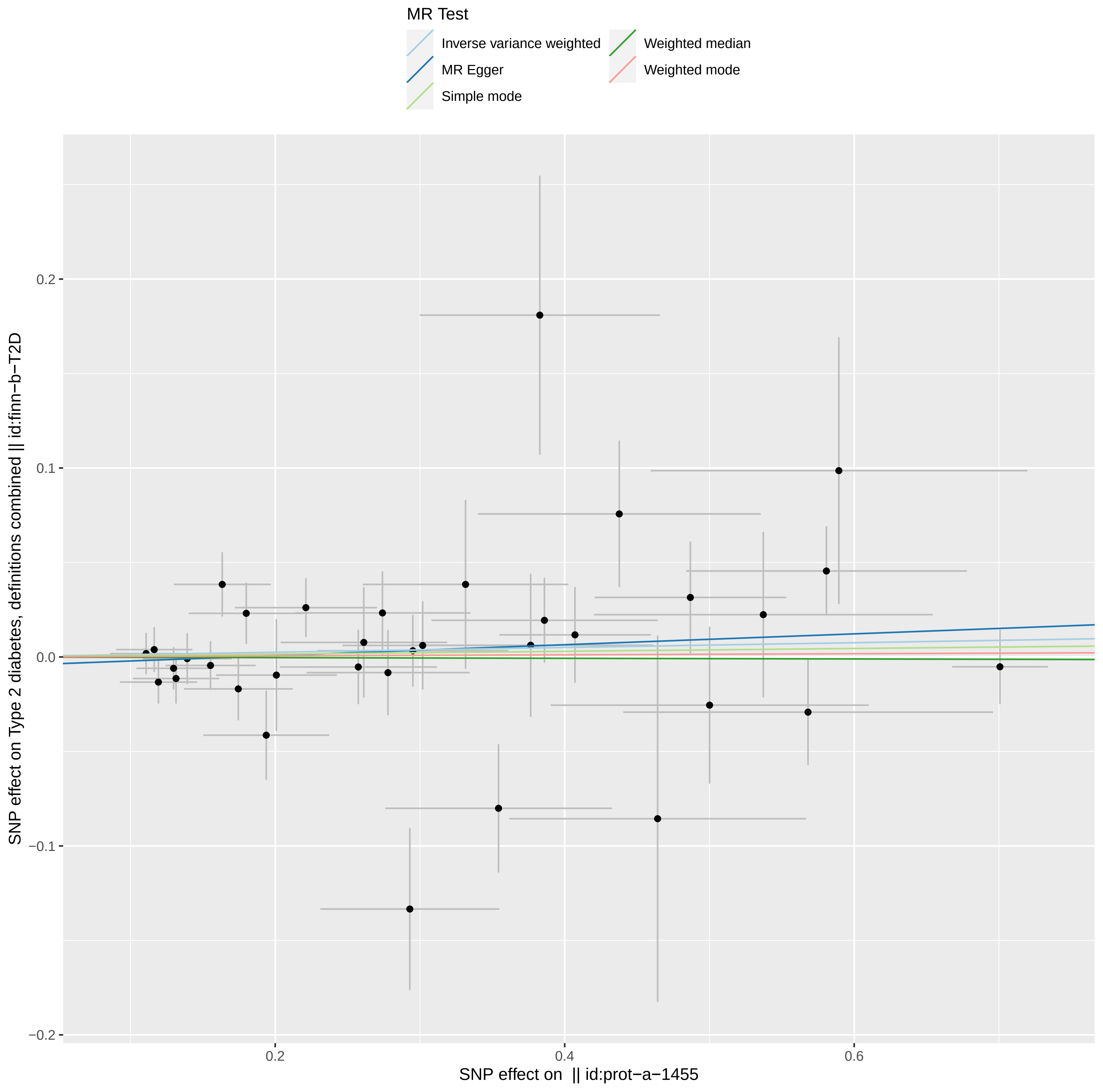

MR Test
Inverse variance weighted
MR Egger
Simple mode
Weighted median
Weighted mode
0.2
0.1
0.0
−0.1
−0.2
SNP effect on Type 2 diabetes, definitions combined || id:finn−b−T2D
0.2
0.4
0.6
SNP effect on || id:prot−a−1455

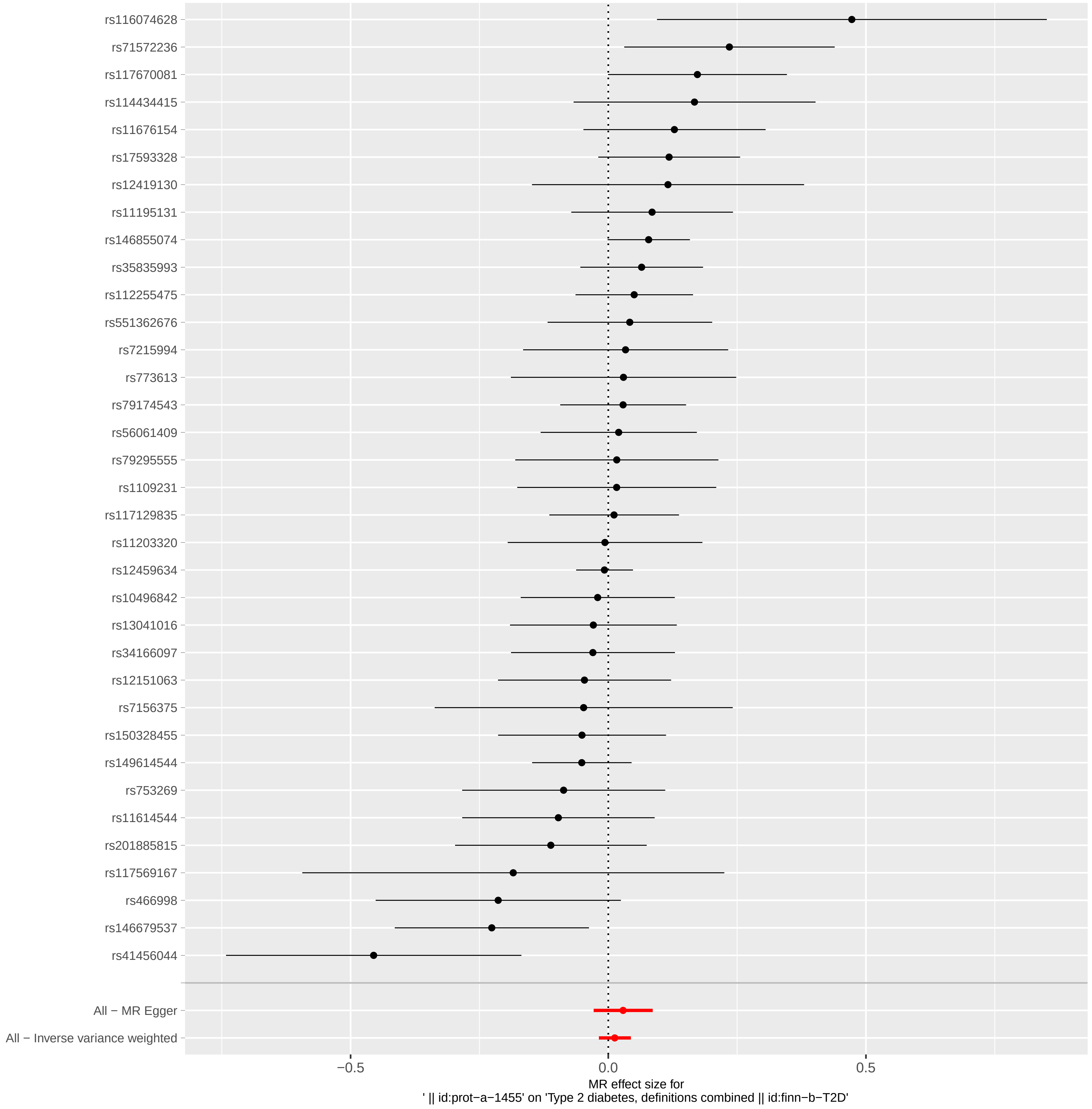
rs116074628
rs71572236
rs117670081
rs114434415
rs11676154
rs17593328
rs12419130
rs11195131
rs146855074
rs35835993
rs112255475
rs551362676
rs7215994
rs773613
rs79174543
rs56061409
rs79295555
rs1109231
rs117129835
rs11203320
rs12459634
rs10496842
rs13041016
rs34166097
rs12151063
rs7156375
rs150328455
rs149614544
rs753269
rs11614544
rs201885815
rs117569167
rs466998
rs146679537
rs41456044
All − MR Egger
All − Inverse variance weighted
−0.5
0.0
0.5
MR effect size for
' || id:prot−a−1455' on 'Type 2 diabetes, definitions combined || id:finn−b−T2D'

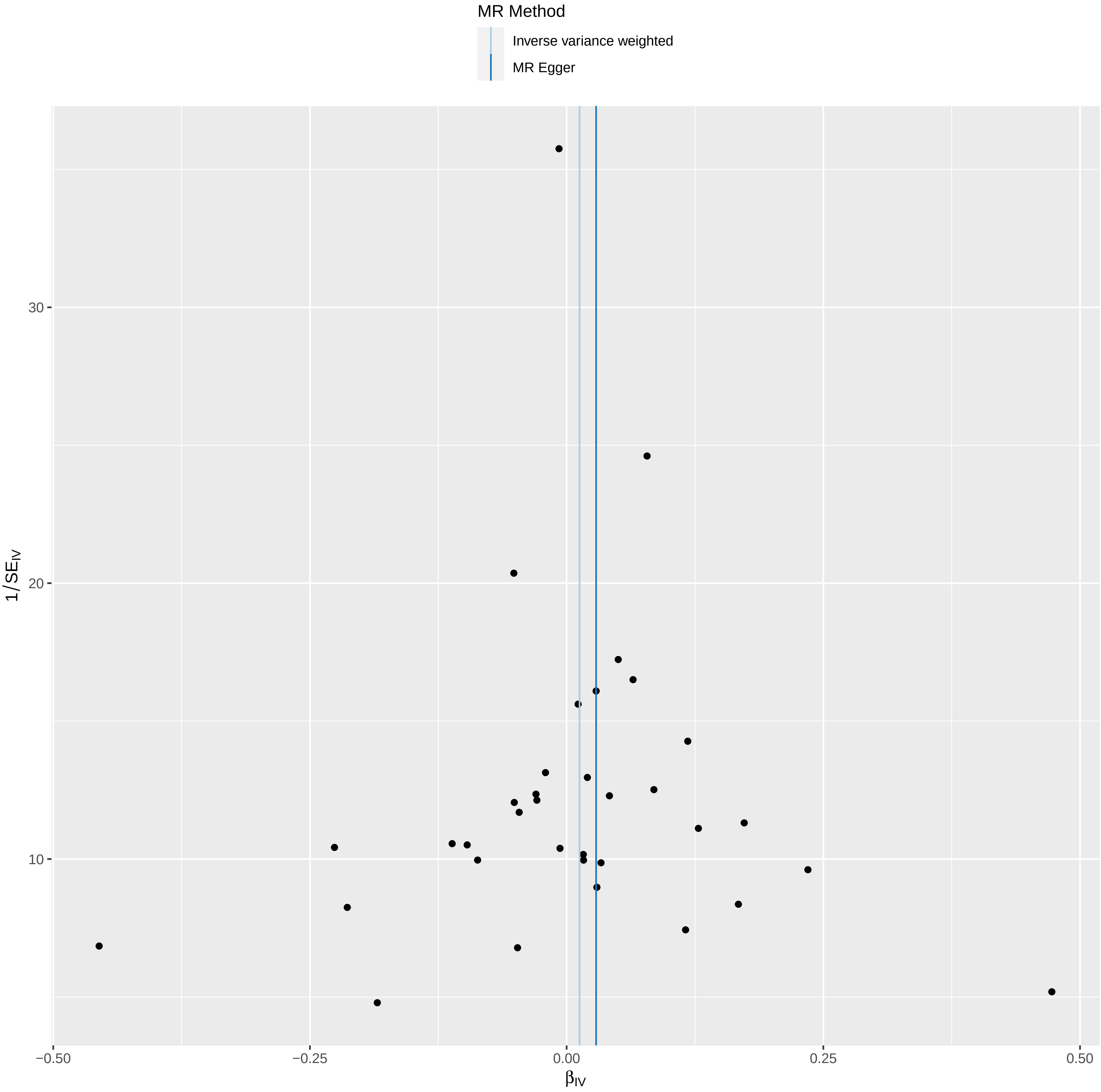
MR Method
Inverse variance weighted
MR Egger
30
20
10
1/SE$_{IV}$
−0.50
−0.25
0.00
0.25
0.50
β$_{IV}$

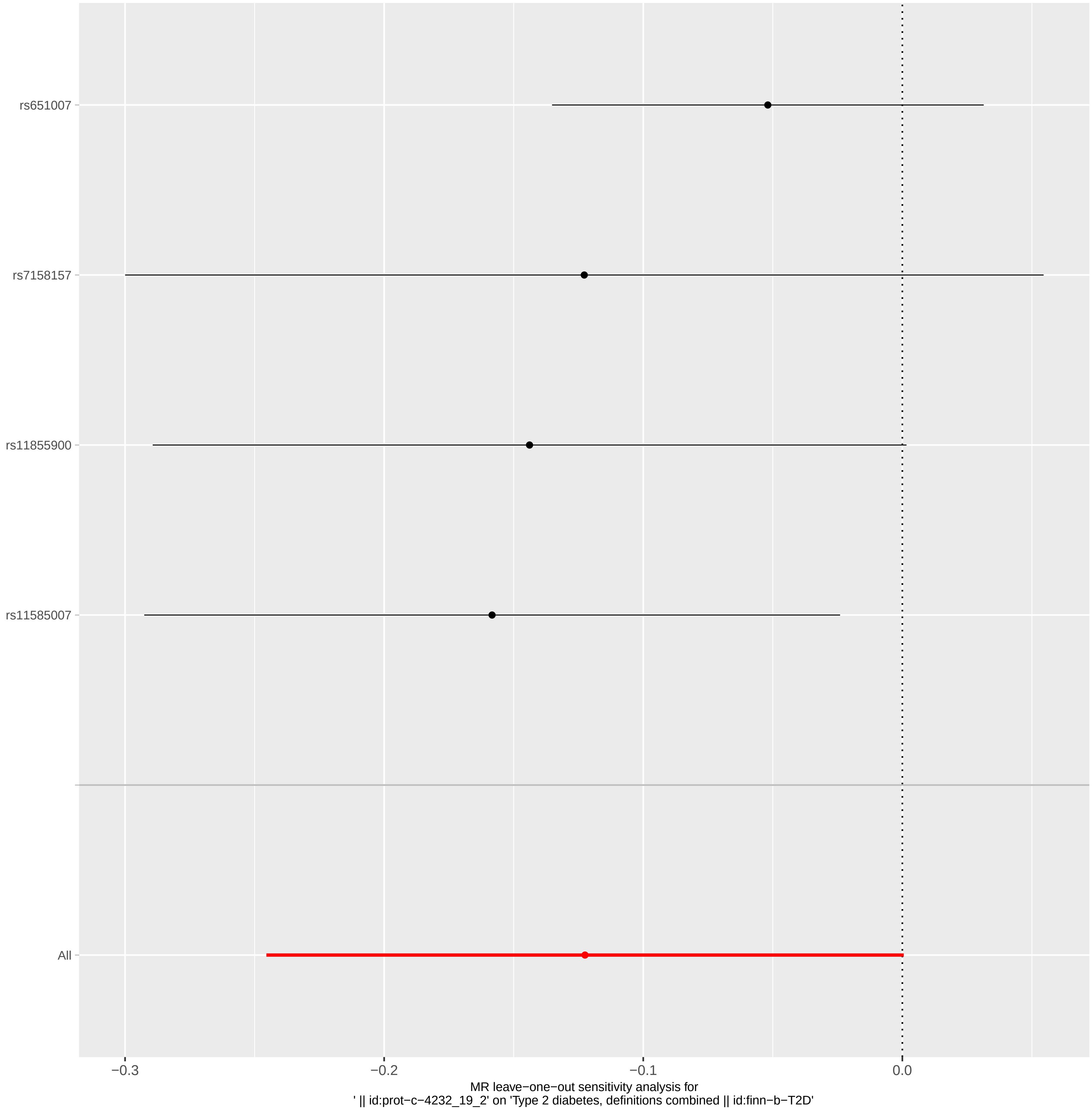
rs651007
rs7158157
rs11855900
rs11585007
All
−0.3
−0.2
−0.1
0.0
MR leave−one−out sensitivity analysis for
' || id:prot−c−4232_19_2' on 'Type 2 diabetes, definitions combined || id:finn−b−T2D'

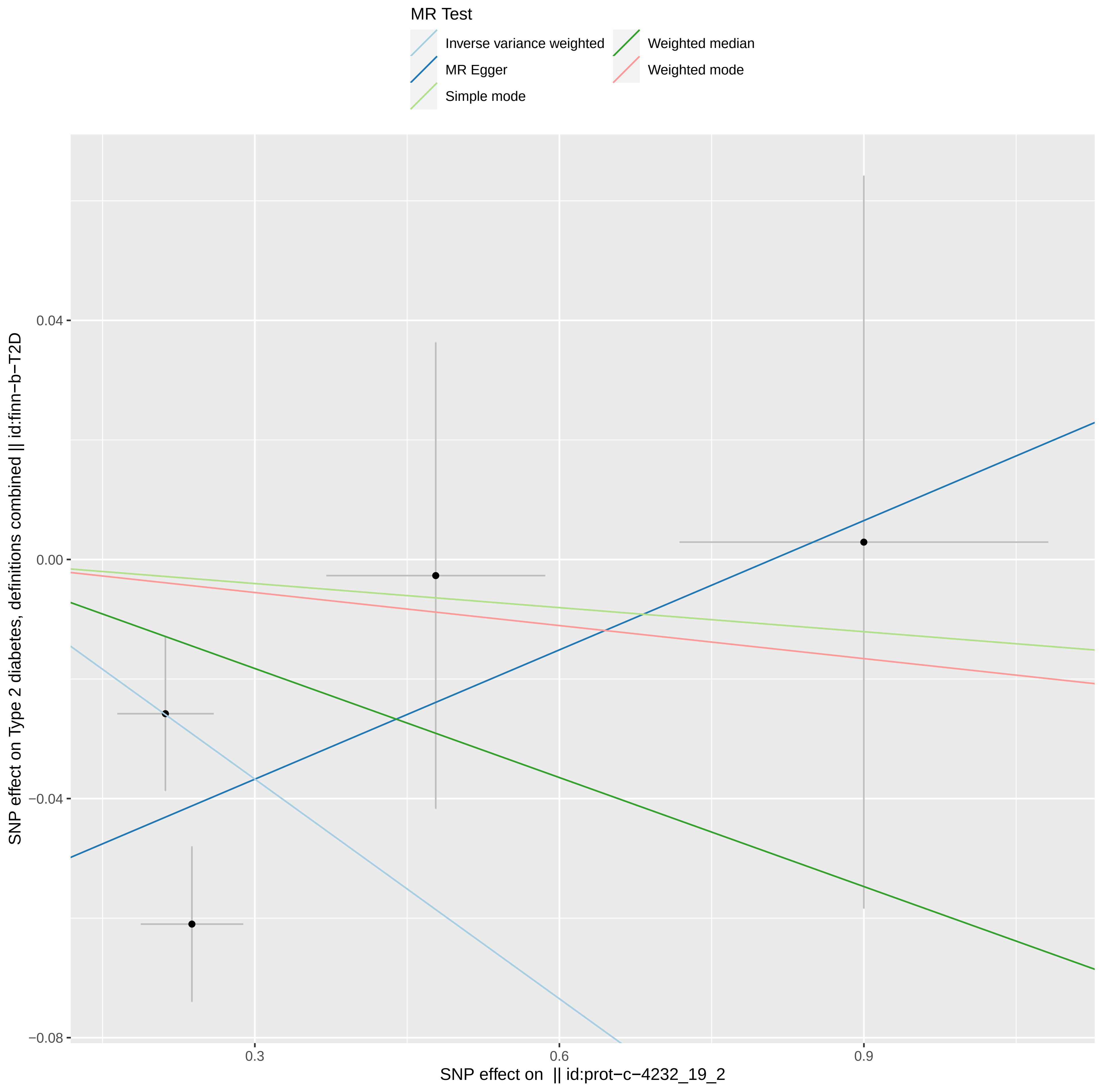

MR Test
Inverse variance weighted
MR Egger
Simple mode
Weighted median
Weighted mode
SNP effect on Type 2 diabetes, definitions combined || id:finn−b−T2D
0.04
0.00
−0.04
−0.08
0.3
0.6
0.9
SNP effect on || id:prot−c−4232_19_2

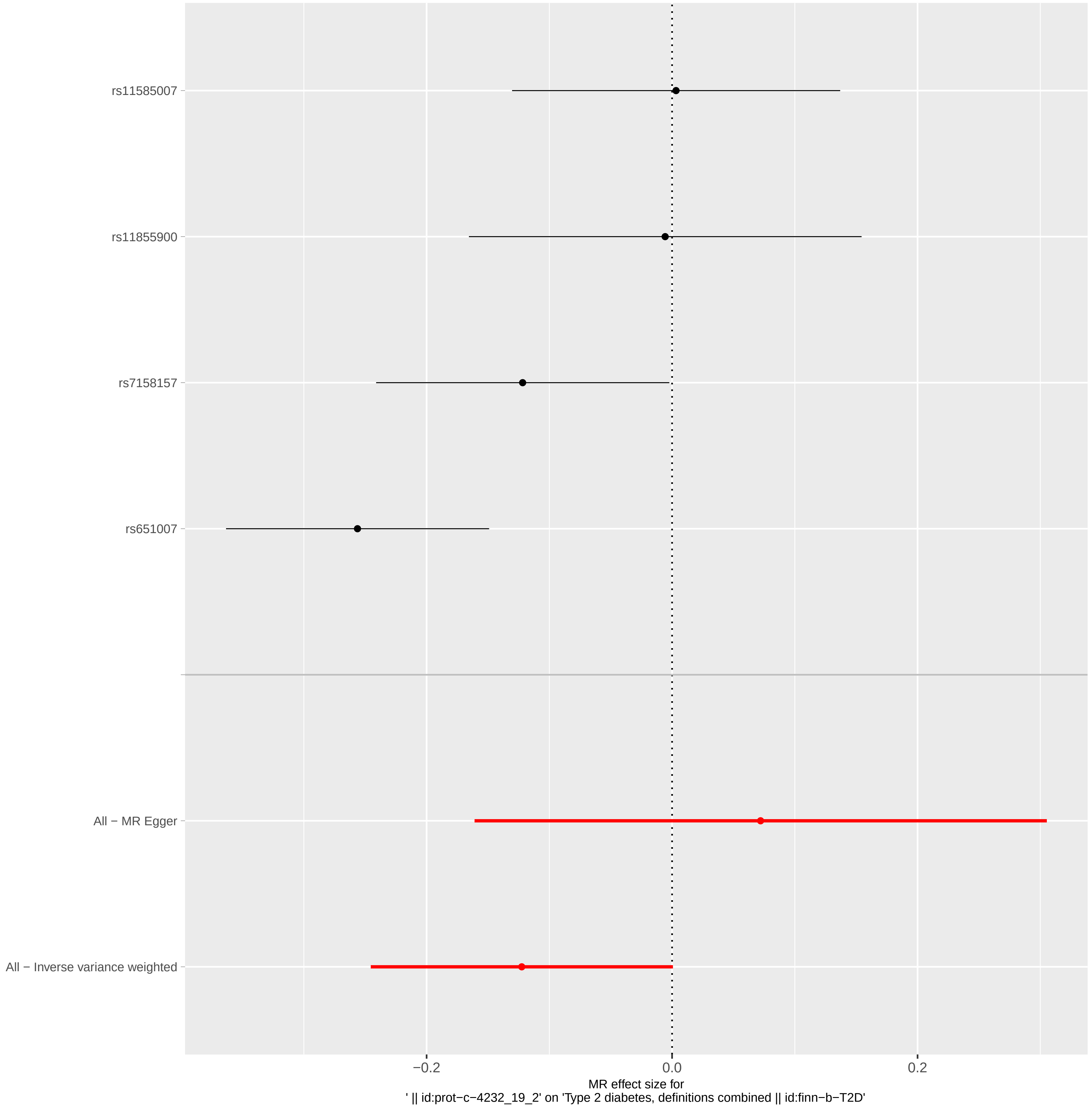

rs11585007
rs11855900
rs7158157
rs651007
All − MR Egger
All − Inverse variance weighted
−0.2
0.0
0.2
MR effect size for
' || id:prot−c−4232_19_2' on 'Type 2 diabetes, definitions combined || id:finn−b−T2D'

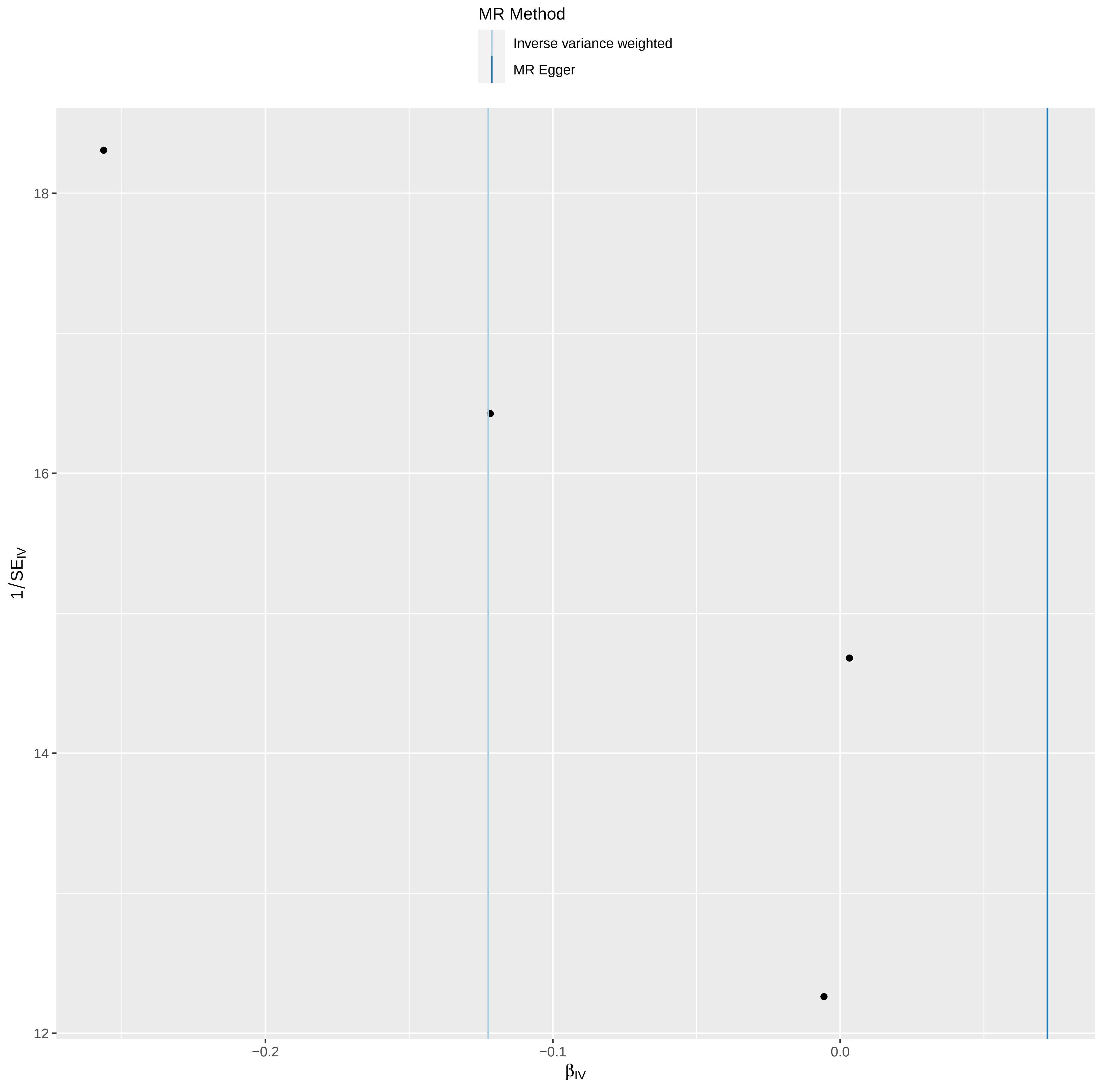

MR Method
Inverse variance weighted
MR Egger
18
16
14
12
1/SE_IV
−0.2
−0.1
0.0
β_IV

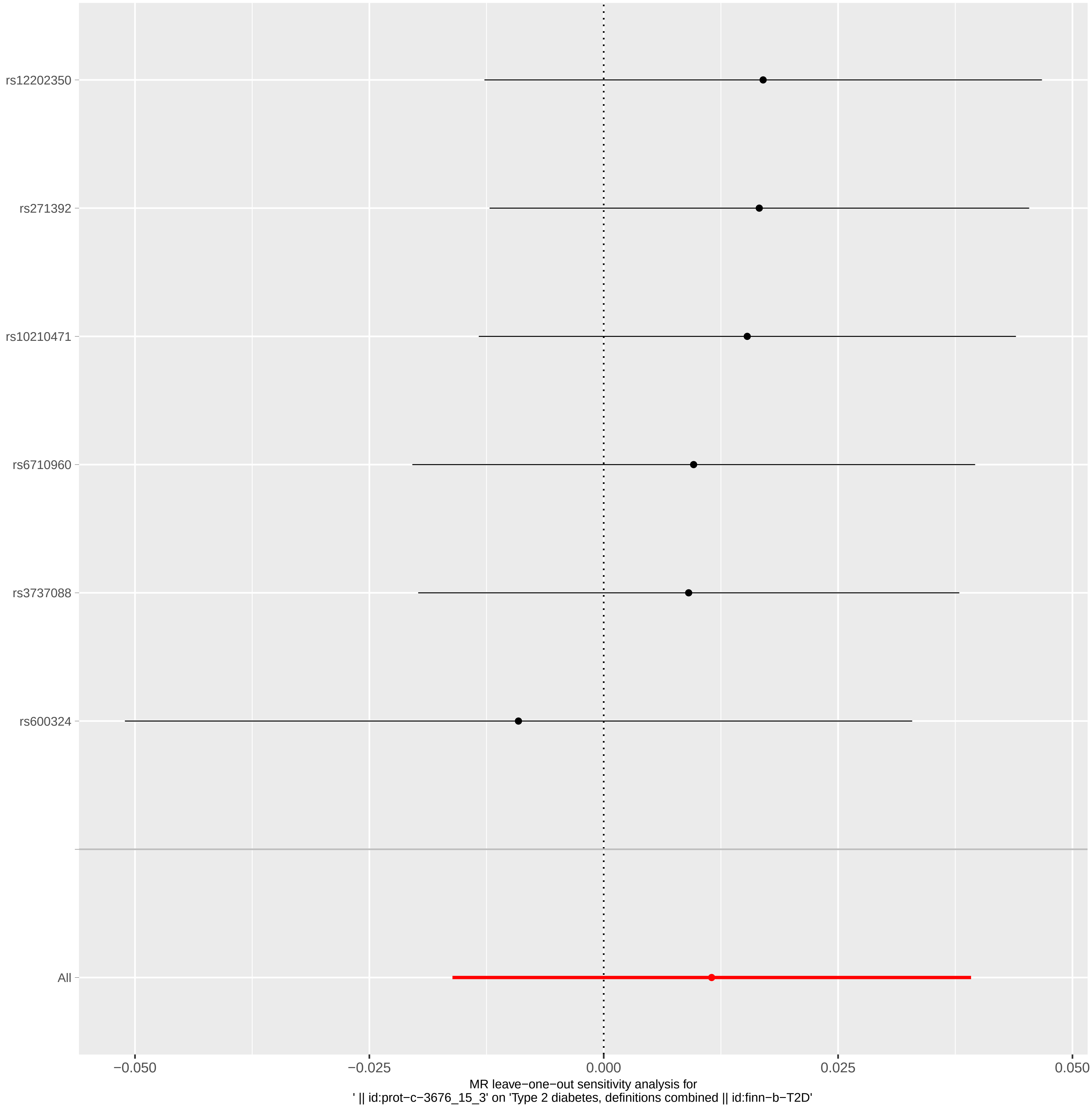
rs12202350
rs271392
rs10210471
rs6710960
rs3737088
rs600324
All
−0.050
−0.025
0.000
0.025
0.050
MR leave−one−out sensitivity analysis for
' || id:prot−c−3676_15_3' on 'Type 2 diabetes, definitions combined || id:finn−b−T2D'

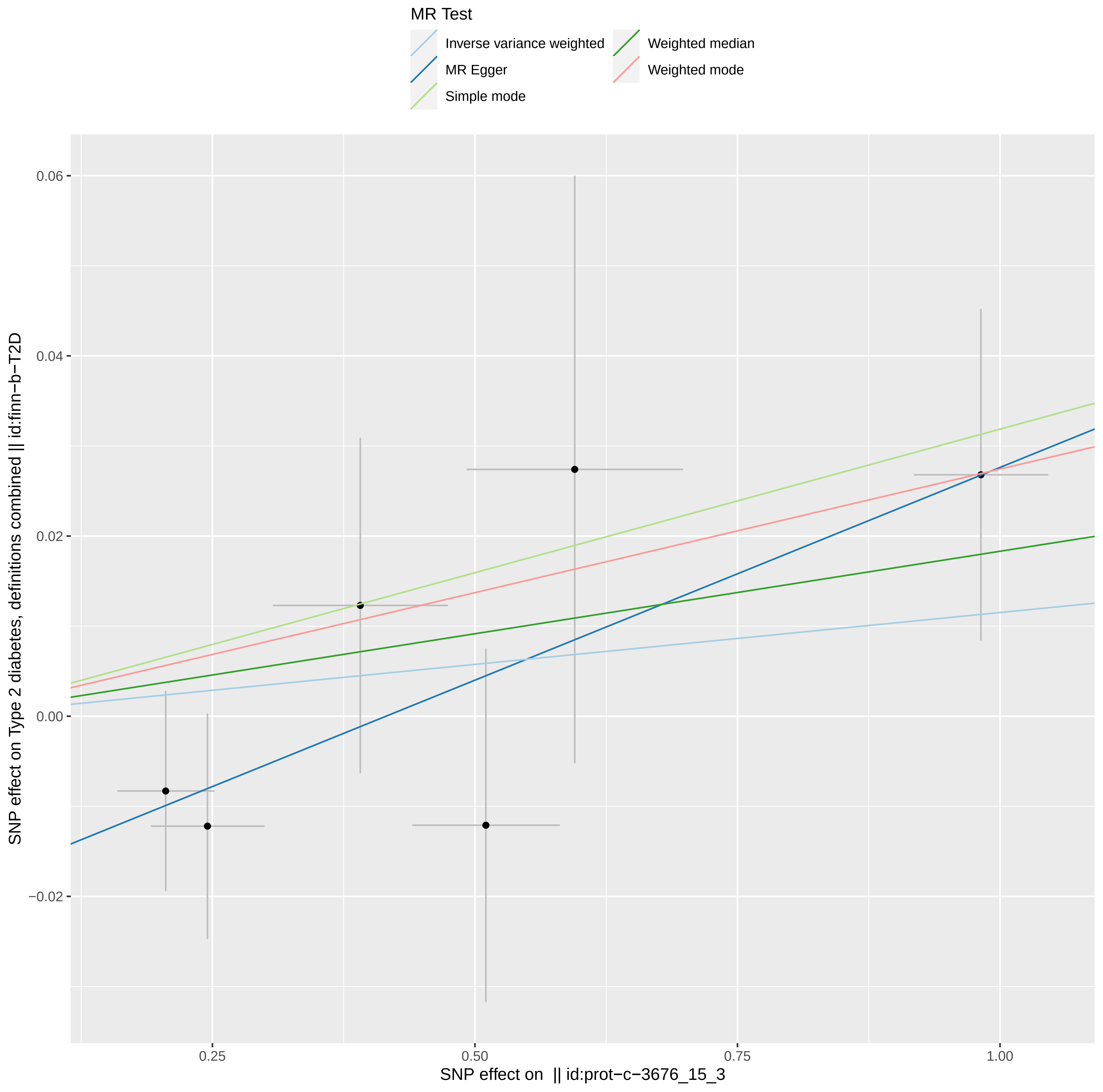

MR Test
Inverse variance weighted
MR Egger
Simple mode
Weighted median
Weighted mode
0.06
0.04
0.02
0.00
−0.02
0.25
0.50
0.75
1.00
SNP effect on Type 2 diabetes, definitions combined || id:finn−b−T2D
SNP effect on || id:prot−c−3676_15_3

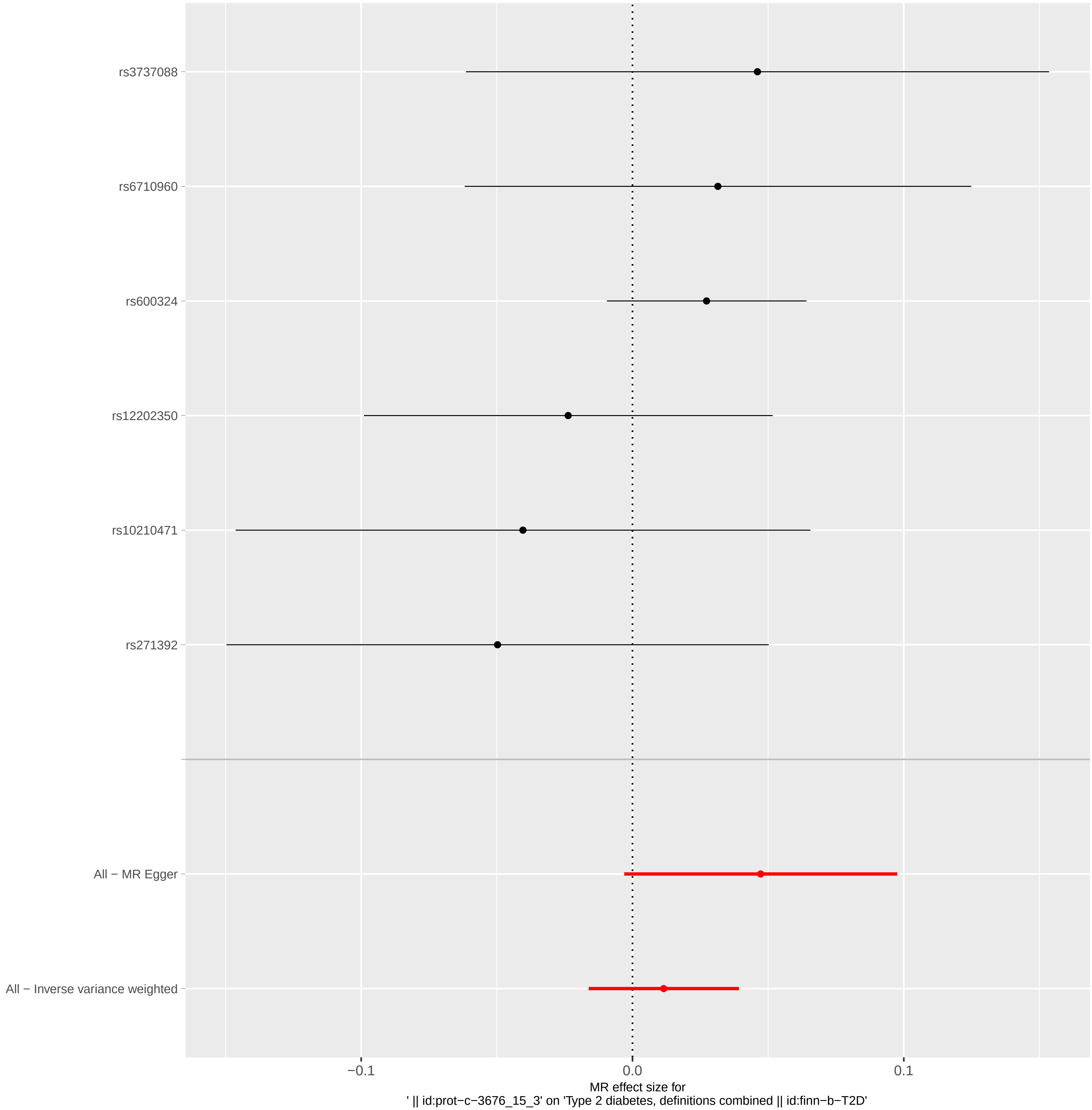
rs3737088
rs6710960
rs600324
rs12202350
rs10210471
rs271392
All − MR Egger
All − Inverse variance weighted
−0.1
0.0
0.1
MR effect size for
' || id:prot−c−3676_15_3' on 'Type 2 diabetes, definitions combined || id:finn−b−T2D'

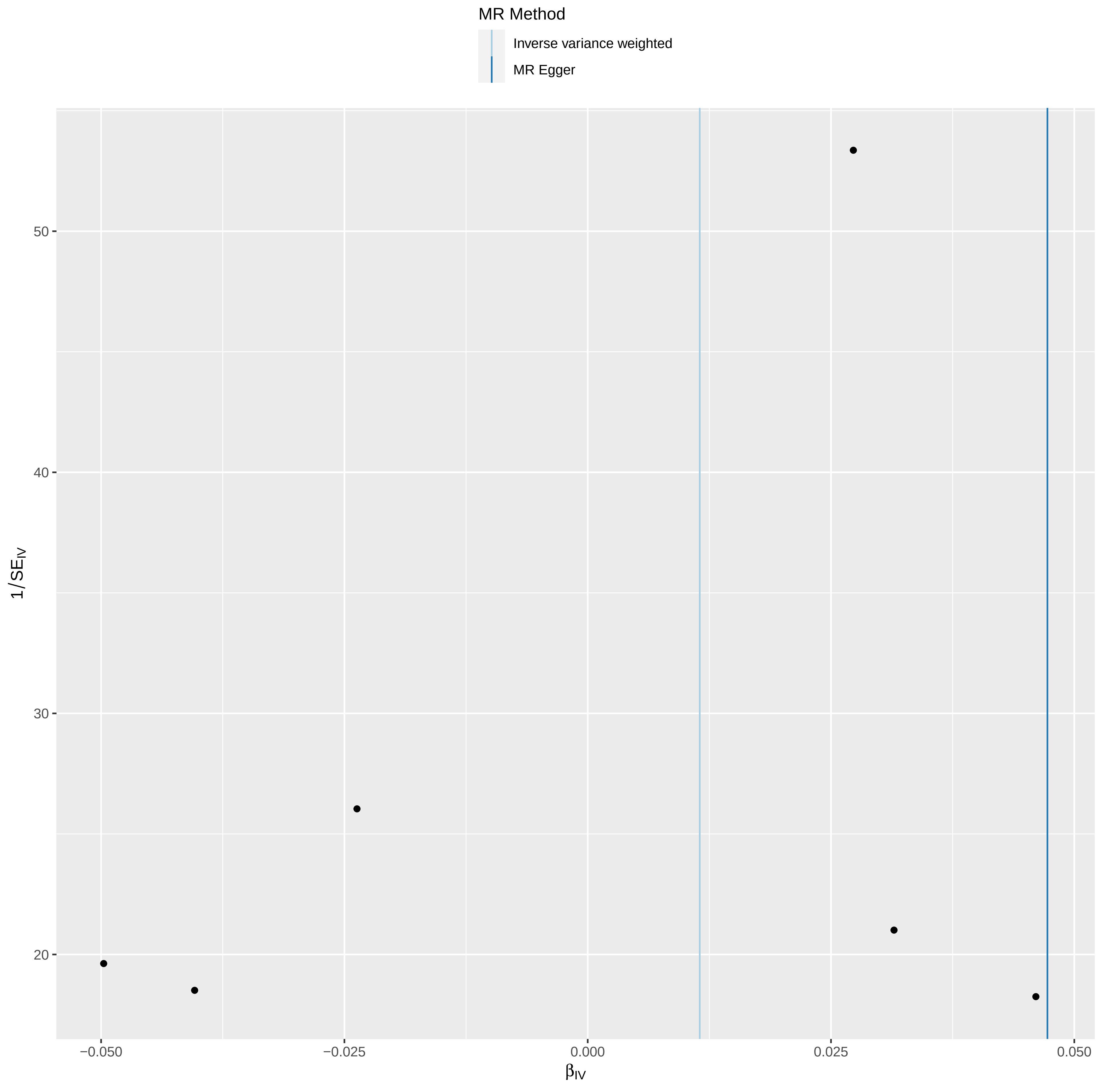

MR Method
Inverse variance weighted
MR Egger
50
40
30
20
1/SE$_{IV}$
−0.050
−0.025
0.000
0.025
0.050
β$_{IV}$

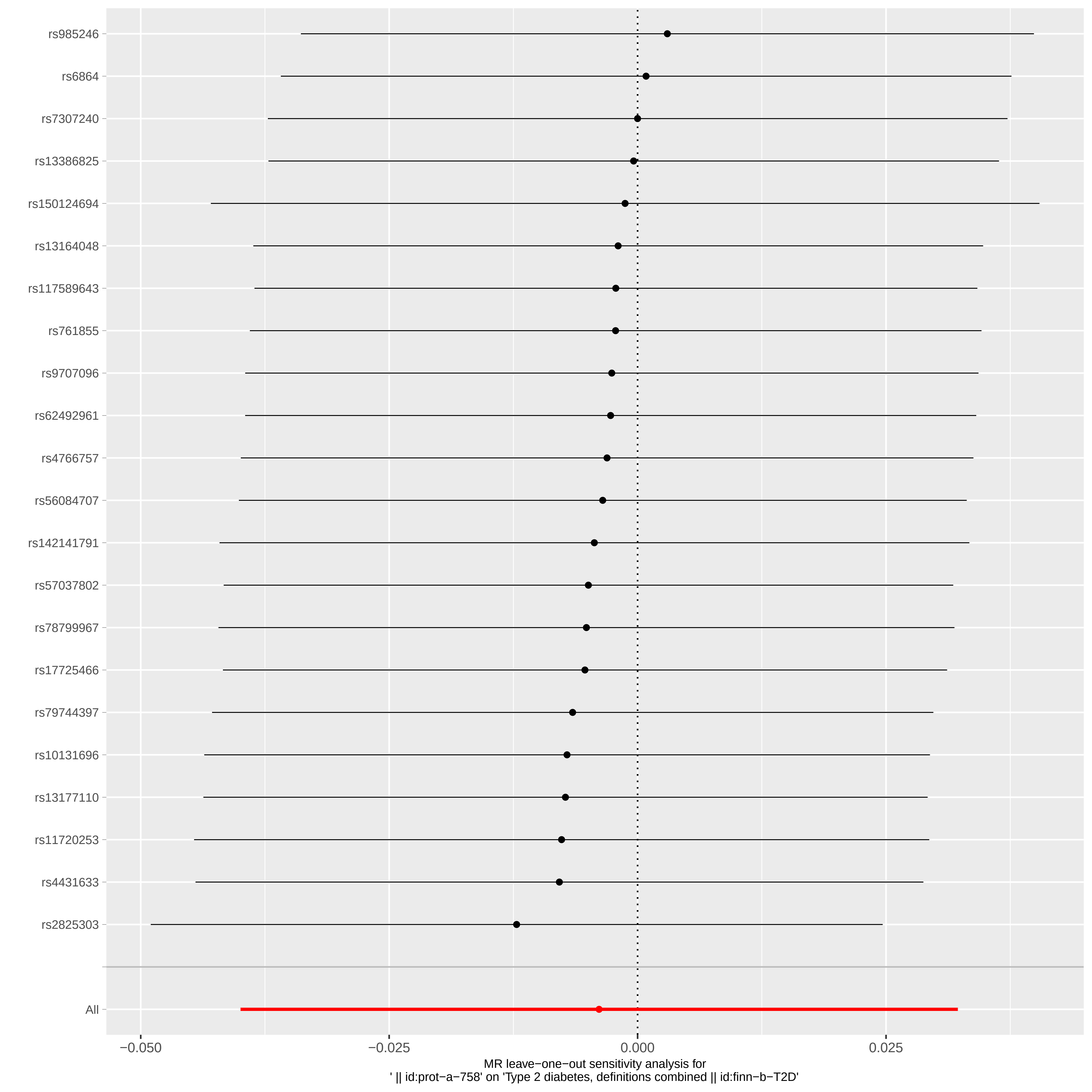
rs985246
rs6864
rs7307240
rs13386825
rs150124694
rs13164048
rs117589643
rs761855
rs9707096
rs62492961
rs4766757
rs56084707
rs142141791
rs57037802
rs78799967
rs17725466
rs79744397
rs10131696
rs13177110
rs11720253
rs4431633
rs2825303
All
−0.050
−0.025
0.000
0.025
MR leave−one−out sensitivity analysis for
' || id:prot−a−758' on 'Type 2 diabetes, definitions combined || id:finn−b−T2D'

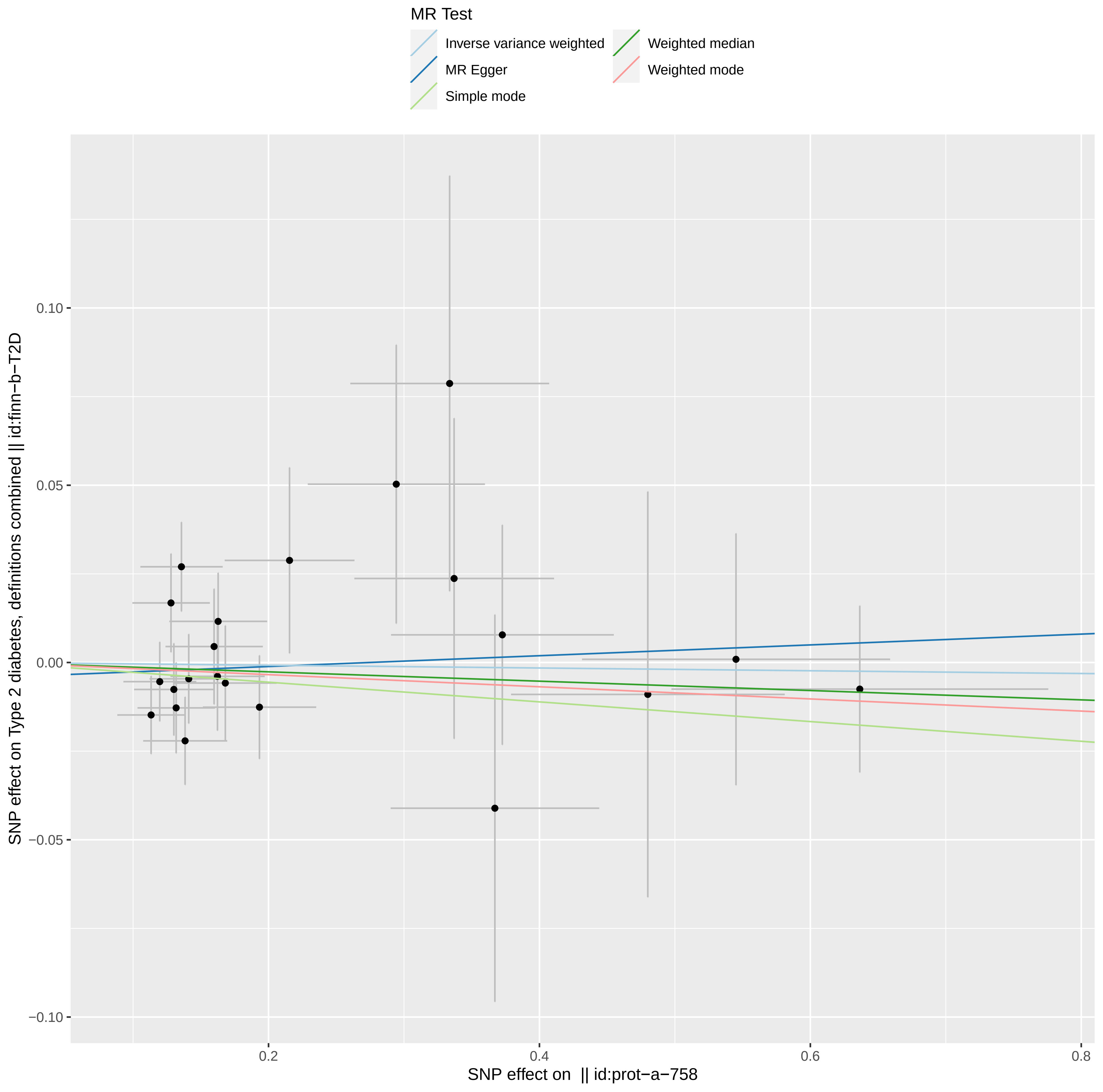

MR Test
Inverse variance weighted
MR Egger
Simple mode
Weighted median
Weighted mode
0.10
0.05
0.00
−0.05
−0.10
0.2
0.4
0.6
0.8
SNP effect on Type 2 diabetes, definitions combined || id:finn−b−T2D
SNP effect on || id:prot−a−758

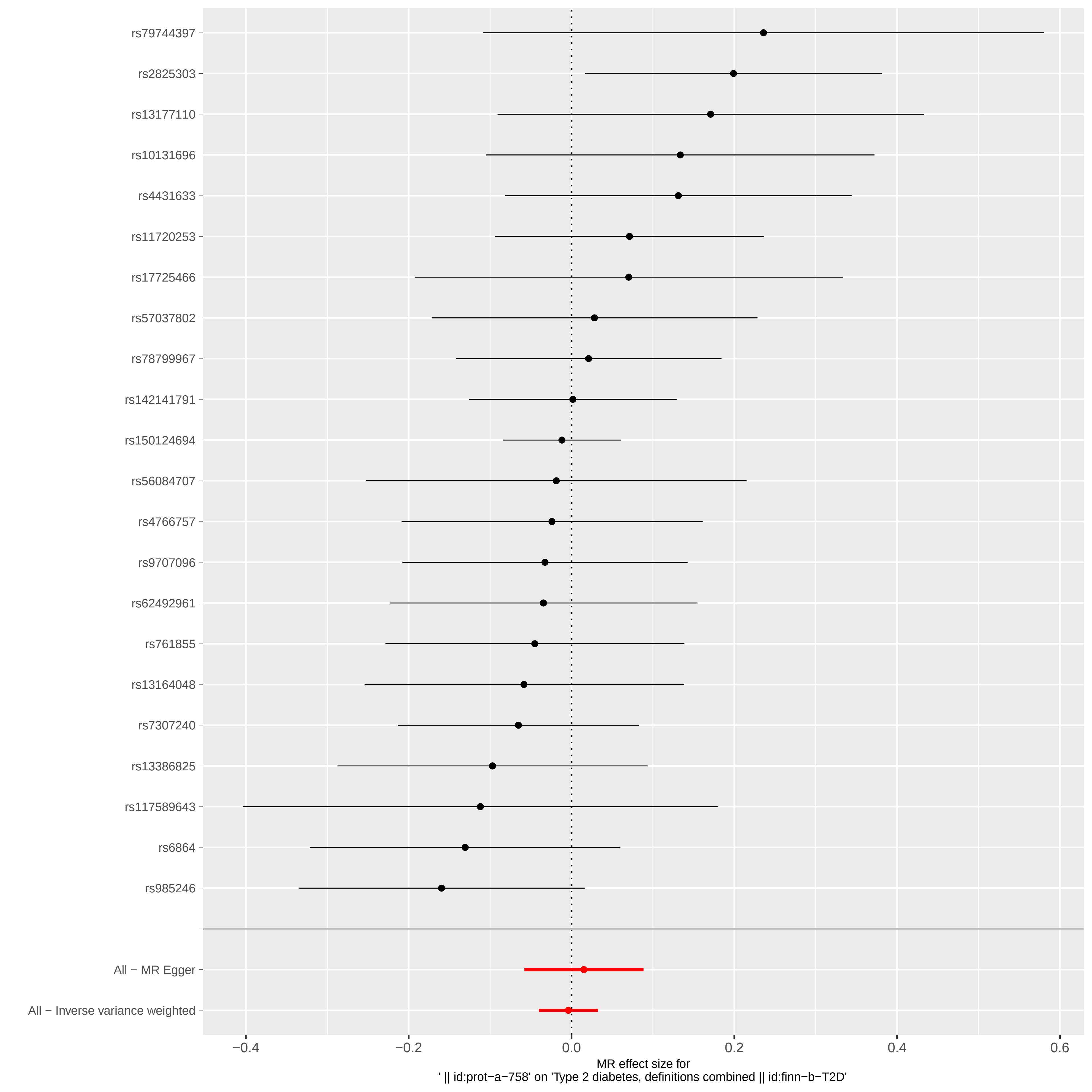

rs79744397
rs2825303
rs13177110
rs10131696
rs4431633
rs11720253
rs17725466
rs57037802
rs78799967
rs142141791
rs150124694
rs56084707
rs4766757
rs9707096
rs62492961
rs761855
rs13164048
rs7307240
rs13386825
rs117589643
rs6864
rs985246
All − MR Egger
All − Inverse variance weighted
−0.4
−0.2
0.0
0.2
0.4
0.6
MR effect size for
' || id:prot−a−758' on 'Type 2 diabetes, definitions combined || id:finn−b−T2D'

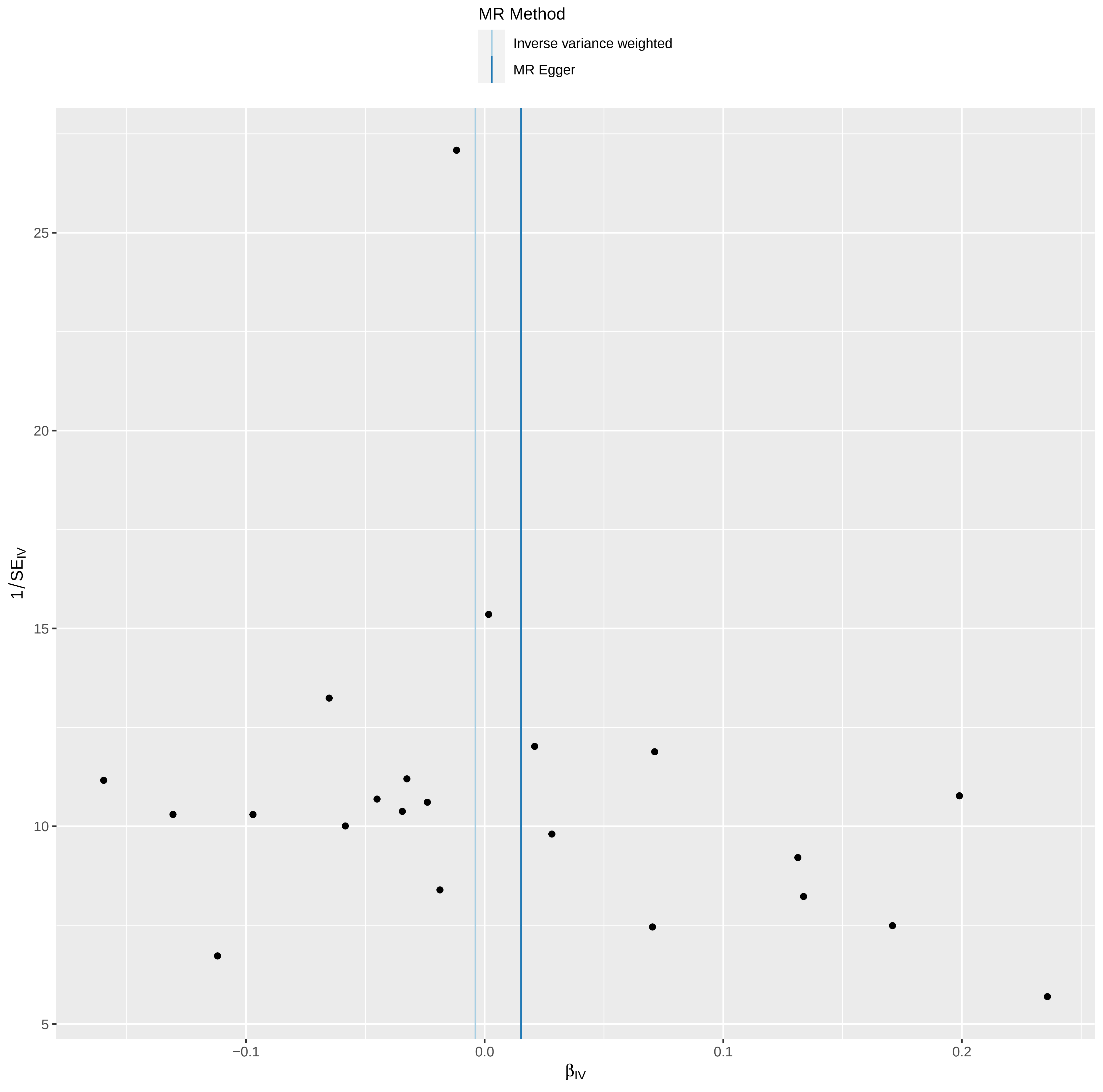
MR Method
Inverse variance weighted
MR Egger
25
20
15
10
5
1/SE_IV
−0.1
0.0
0.1
0.2
β_IV
