# Supplementary material for: Mendelian randomization analysis demonstrates the causal effects of IGF family members in diabetes
Source: Front Med (Lausanne). 2024 Feb 5;11:1332162. doi: 10.3389/fmed.2024.1332162 (PMC10875044; doi:10.3389/fmed.2024.1332162)
Supplement: Supplementary file 3 [file Data_Sheet_3.PDF]

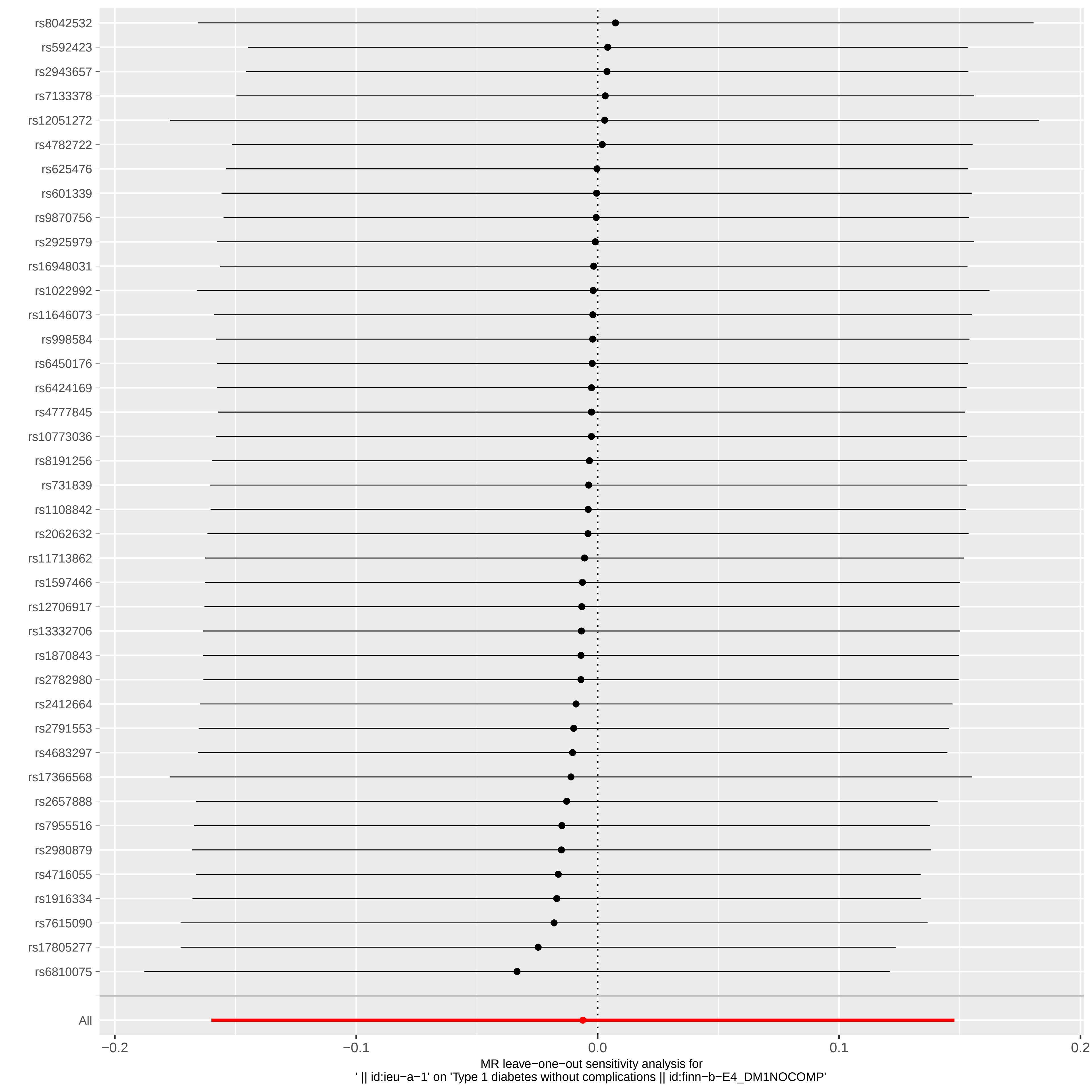

rs8042532
rs592423
rs2943657
rs7133378
rs12051272
rs4782722
rs625476
rs601339
rs9870756
rs2925979
rs16948031
rs1022992
rs11646073
rs998584
rs6450176
rs6424169
rs4777845
rs10773036
rs8191256
rs731839
rs1108842
rs2062632
rs11713862
rs1597466
rs12706917
rs13332706
rs1870843
rs2782980
rs2412664
rs2791553
rs4683297
rs17366568
rs2657888
rs7955516
rs2980879
rs4716055
rs1916334
rs7615090
rs17805277
rs6810075
All
−0.2
−0.1
0.0
0.1
0.2
MR leave−one−out sensitivity analysis for
' || id:ieu−a−1' on 'Type 1 diabetes without complications || id:finn−b−E4_DM1NOCOMP'

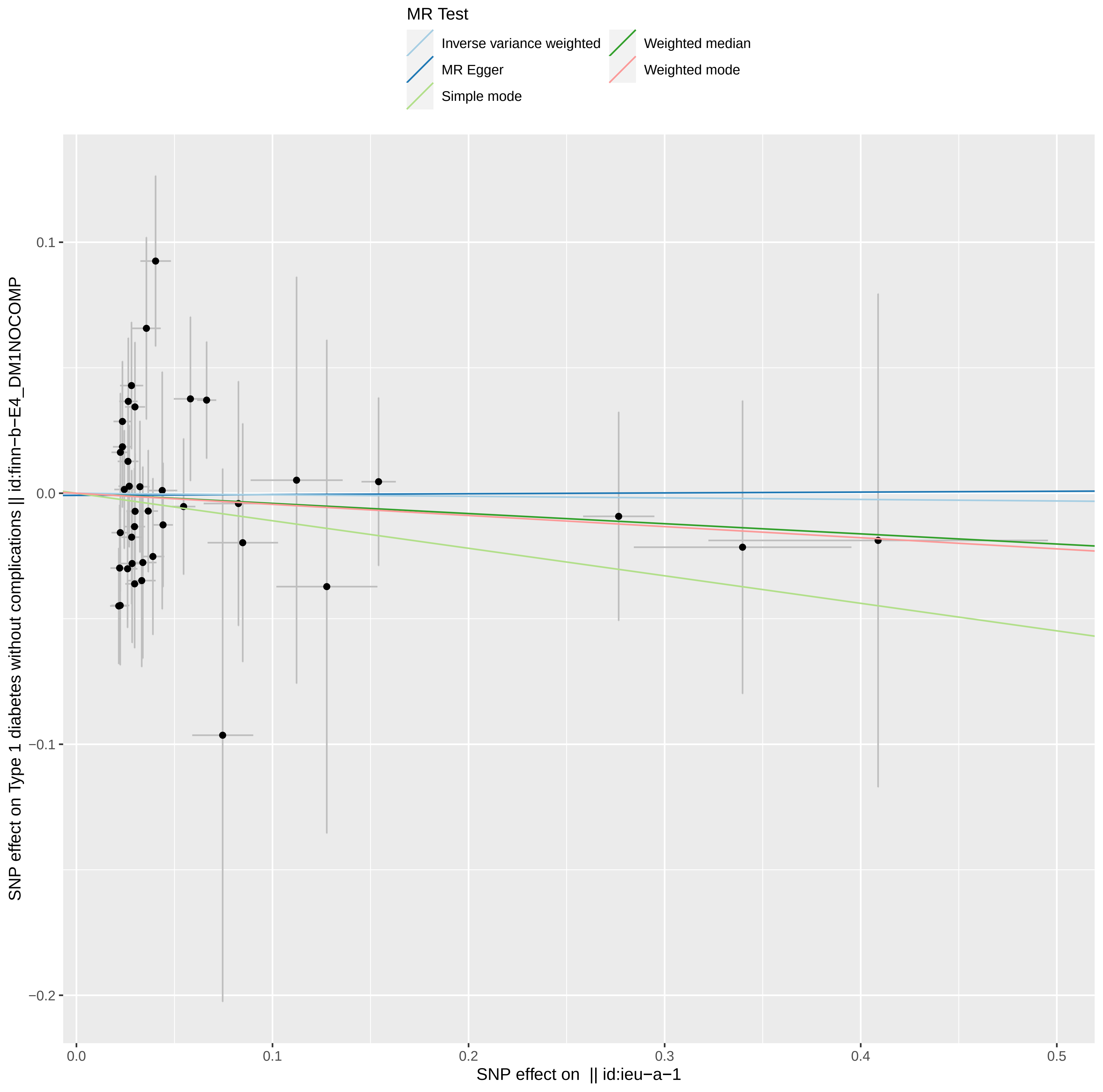
MR Test
Inverse variance weighted
MR Egger
Simple mode
Weighted median
Weighted mode
SNP effect on Type 1 diabetes without complications || id:finn-b-E4_DM1NOCOMP
0.1
0.0
−0.1
−0.2
0.0
0.1
0.2
0.3
0.4
0.5
SNP effect on || id:ieu−a−1

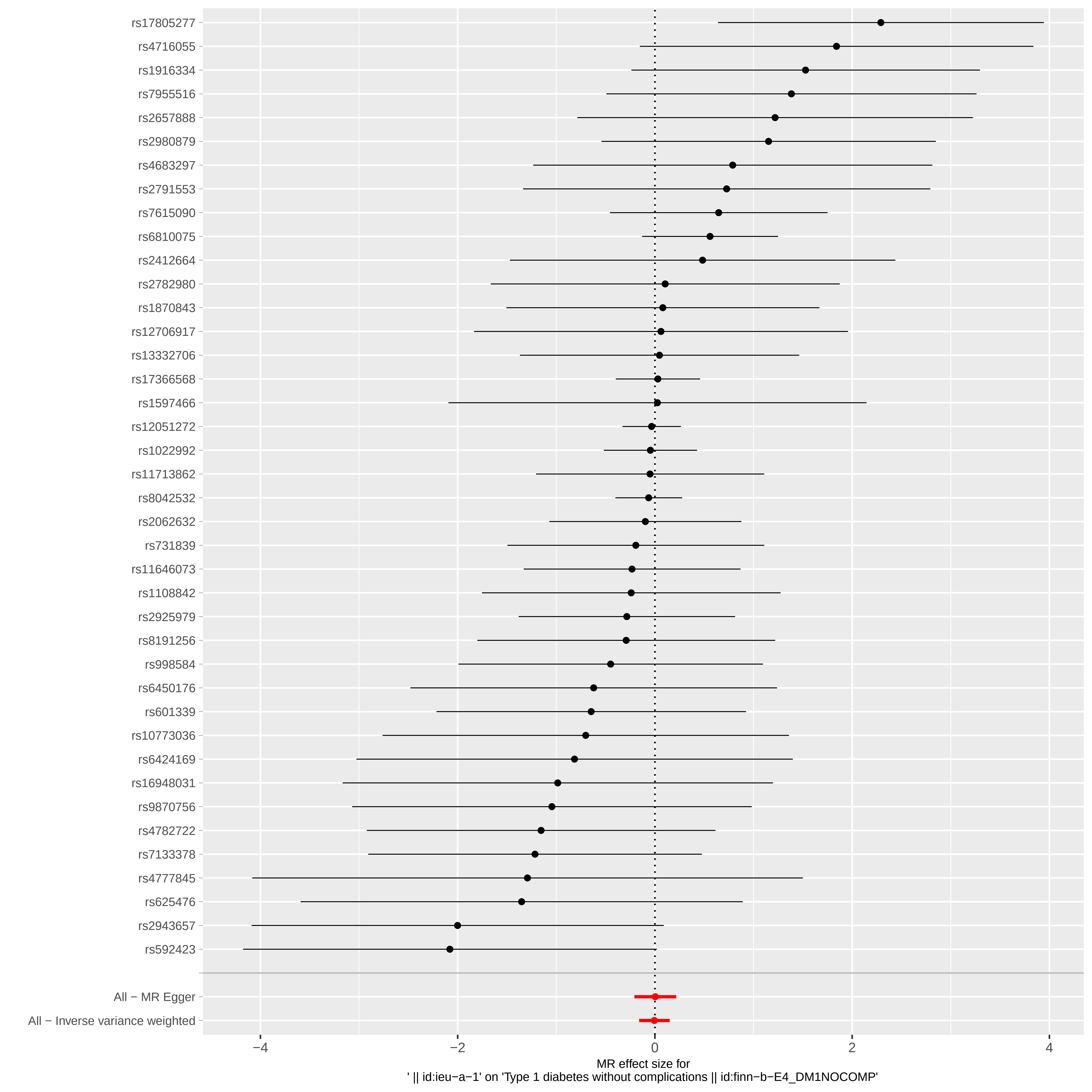
rs17805277
rs4716055
rs1916334
rs7955516
rs2657888
rs2980879
rs4683297
rs2791553
rs7615090
rs6810075
rs2412664
rs2782980
rs1870843
rs12706917
rs13332706
rs17366568
rs1597466
rs12051272
rs1022992
rs11713862
rs8042532
rs2062632
rs731839
rs11646073
rs1108842
rs2925979
rs8191256
rs998584
rs6450176
rs601339
rs10773036
rs6424169
rs16948031
rs9870756
rs4782722
rs7133378
rs4777845
rs625476
rs2943657
rs592423
All − MR Egger
All − Inverse variance weighted
−4
−2
0
2
4
MR effect size for
' || id:ieu−a−1' on 'Type 1 diabetes without complications || id:finn−b−E4_DM1NOCOMP'

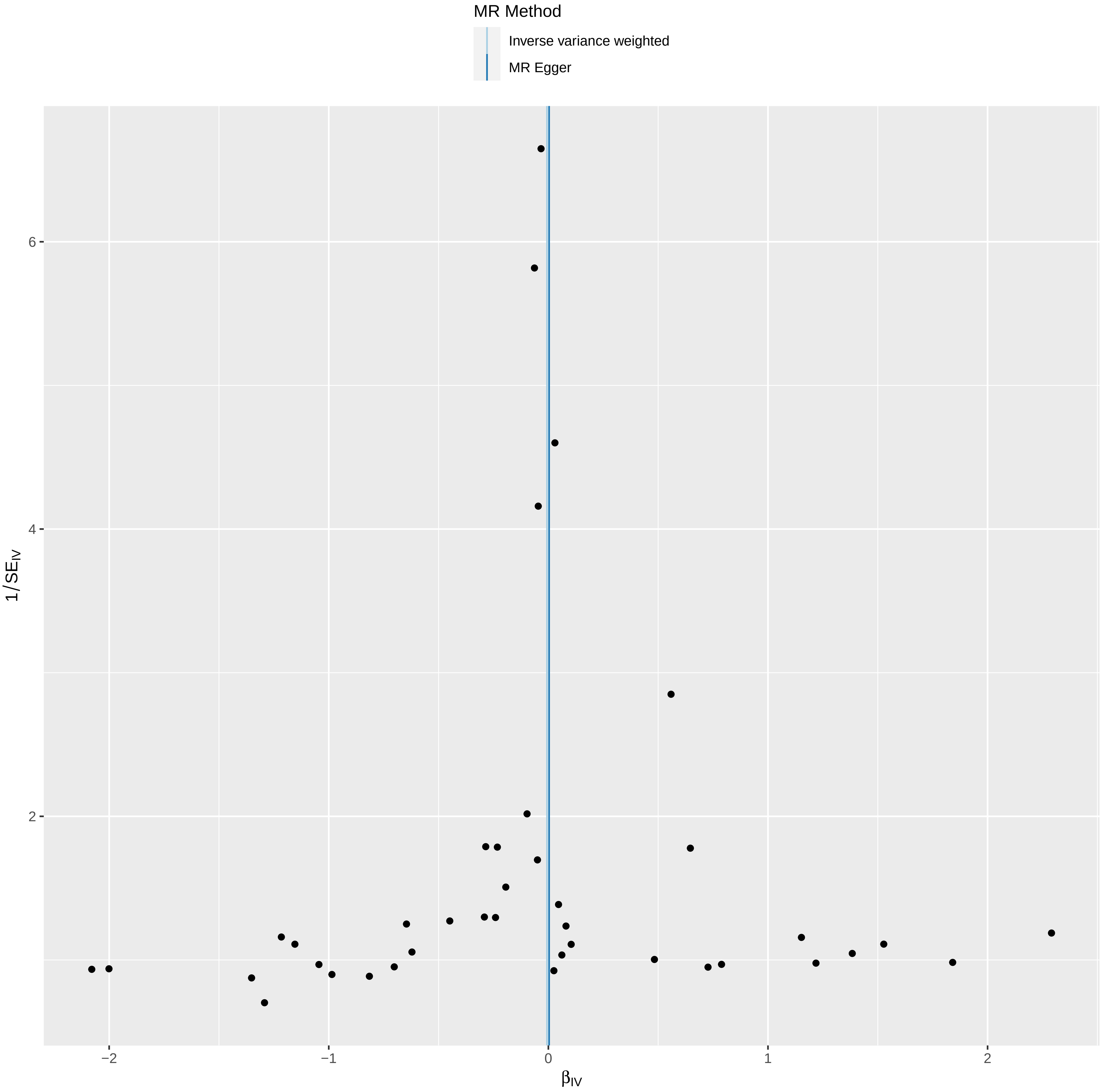
MR Method
Inverse variance weighted
MR Egger
$1/SE_{IV}$
β_{IV}
6
4
2
−2
−1
0
1
2

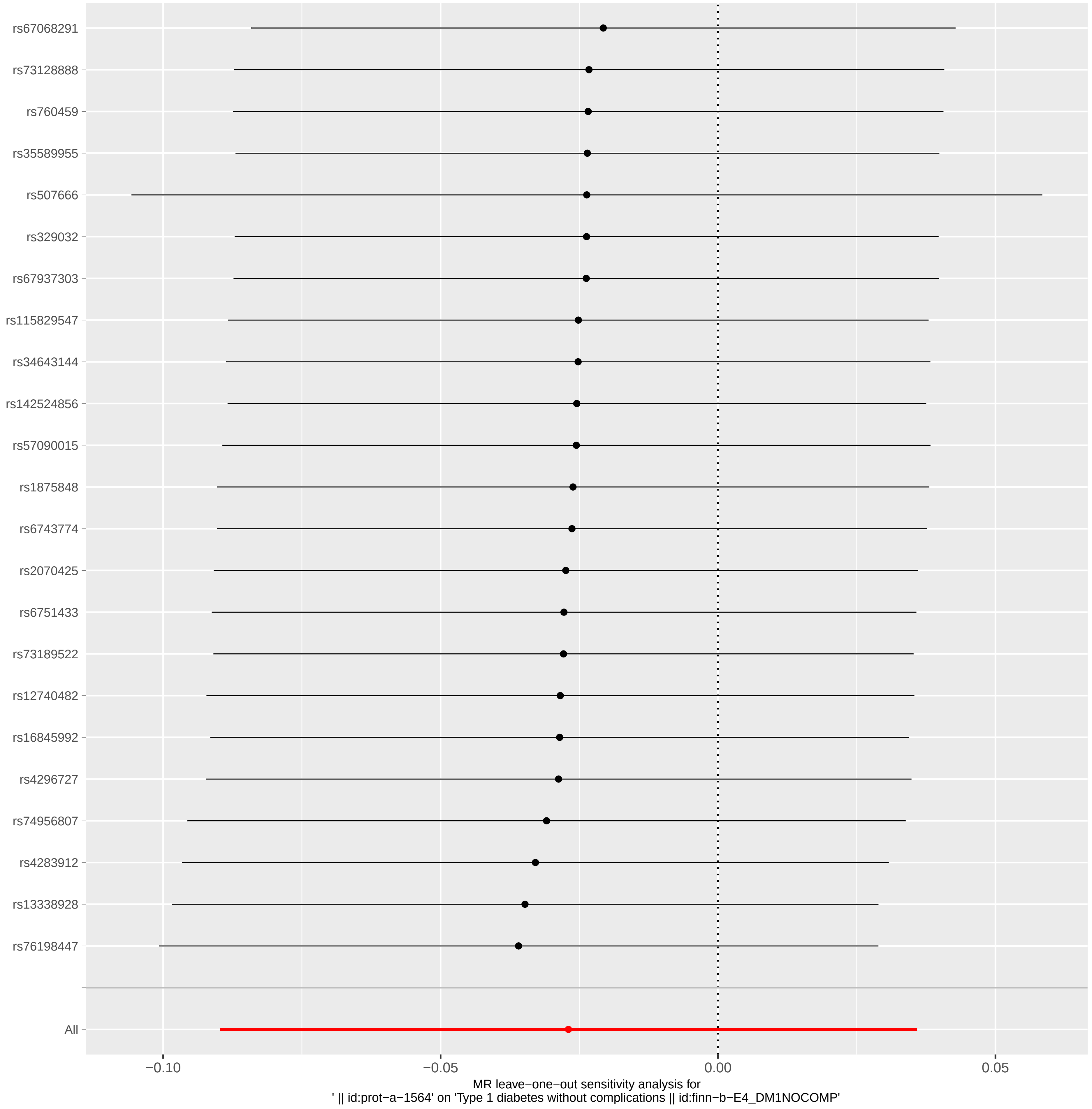

rs67068291
rs73128888
rs760459
rs35589955
rs507666
rs329032
rs67937303
rs115829547
rs34643144
rs142524856
rs57090015
rs1875848
rs6743774
rs2070425
rs6751433
rs73189522
rs12740482
rs16845992
rs4296727
rs74956807
rs4283912
rs13338928
rs76198447
All
−0.10
−0.05
0.00
0.05
MR leave−one−out sensitivity analysis for
' || id:prot−a−1564' on 'Type 1 diabetes without complications || id:finn−b−E4_DM1NOCOMP'

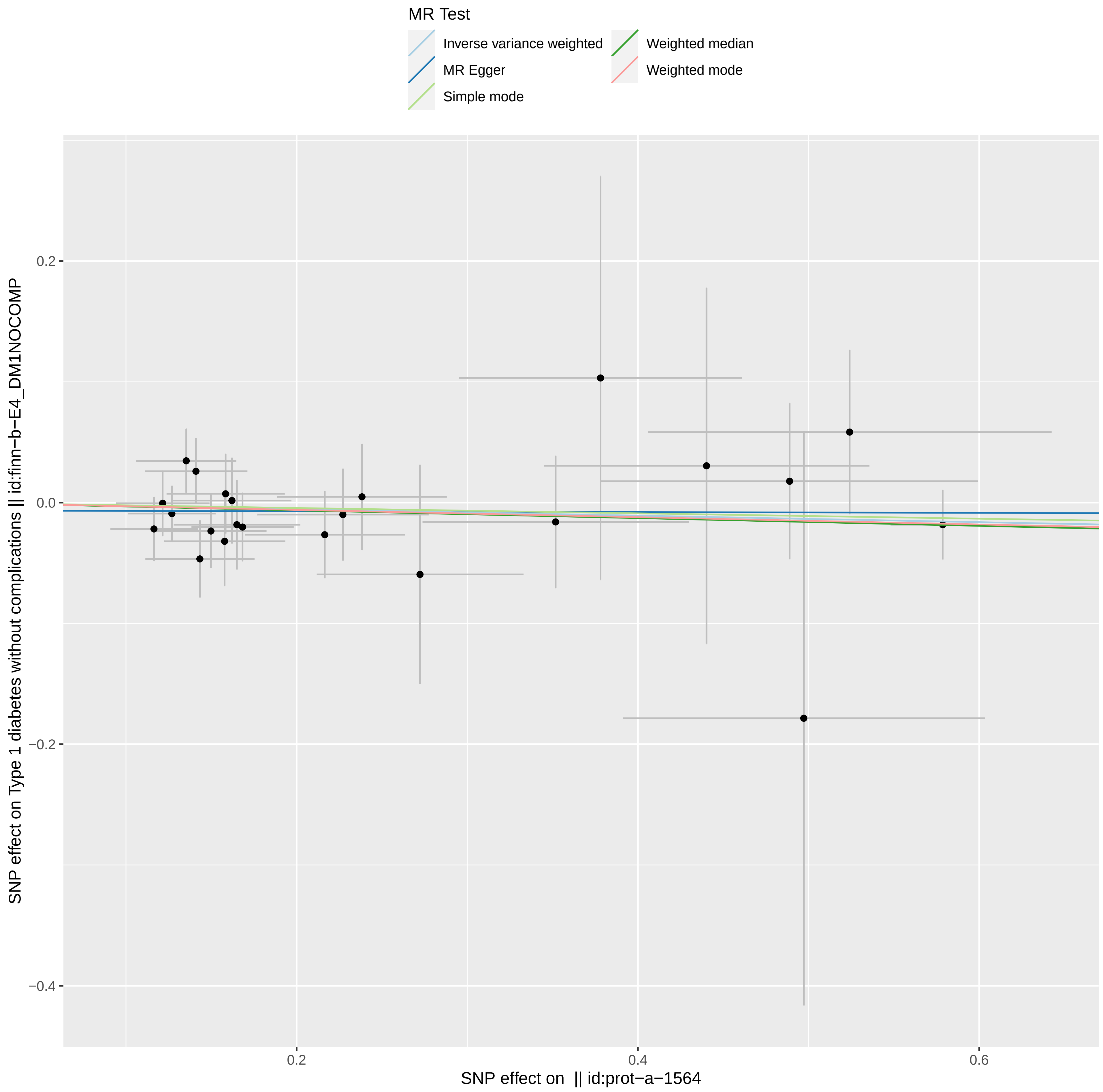

MR Test
Inverse variance weighted
MR Egger
Simple mode
Weighted median
Weighted mode
0.2
0.0
−0.2
−0.4
0.2
0.4
0.6
SNP effect on Type 1 diabetes without complications || id:finn−b−E4_DM1NOCOMP
SNP effect on || id:prot−a−1564

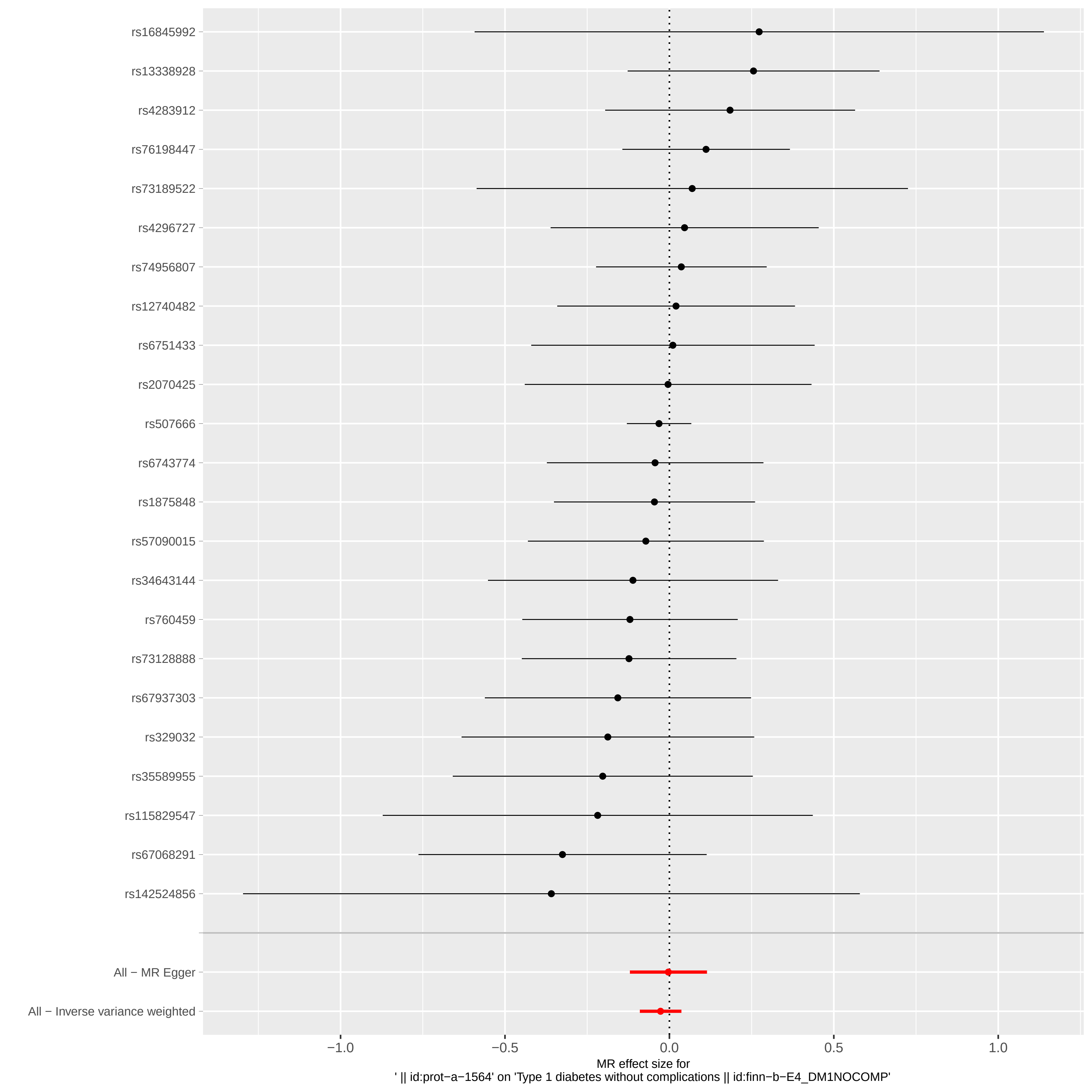
rs16845992
rs13338928
rs4283912
rs76198447
rs73189522
rs4296727
rs74956807
rs12740482
rs6751433
rs2070425
rs507666
rs6743774
rs1875848
rs57090015
rs34643144
rs760459
rs73128888
rs67937303
rs329032
rs35589955
rs115829547
rs67068291
rs142524856
All − MR Egger
All − Inverse variance weighted
−1.0
−0.5
0.0
0.5
1.0
MR effect size for
' || id:prot−a−1564' on 'Type 1 diabetes without complications || id:finn−b−E4_DM1NOCOMP'

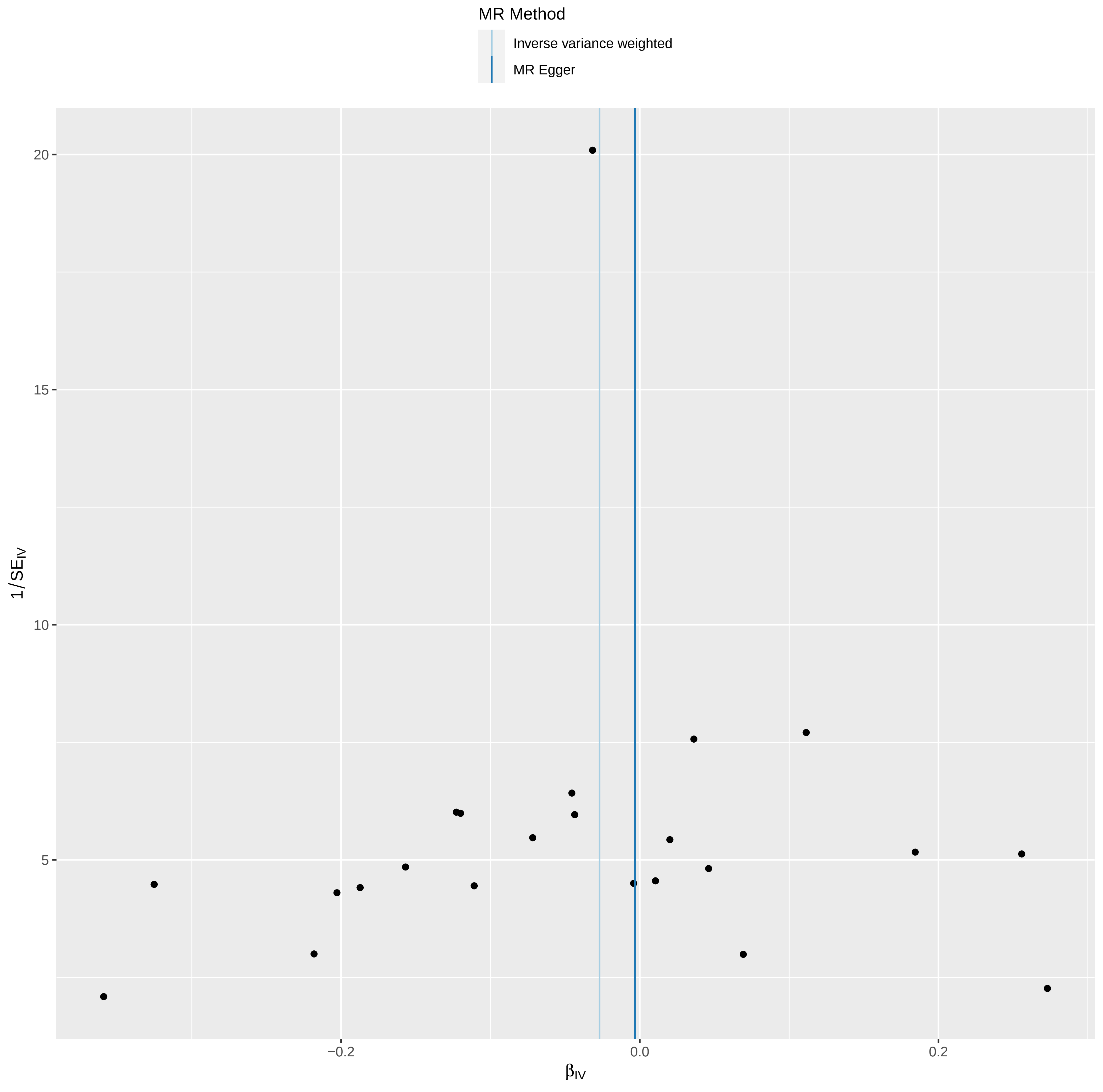
MR Method
Inverse variance weighted
MR Egger
20
15
10
5
1/SE_IV
−0.2
0.0
0.2
β_IV
