# Supplementary material for: Mendelian randomization analysis demonstrates the causal effects of IGF family members in diabetes
Source: Front Med (Lausanne). 2024 Feb 5;11:1332162. doi: 10.3389/fmed.2024.1332162 (PMC10875044; doi:10.3389/fmed.2024.1332162)
Supplement: Supplementary file 4 [file Data_Sheet_4.PDF]

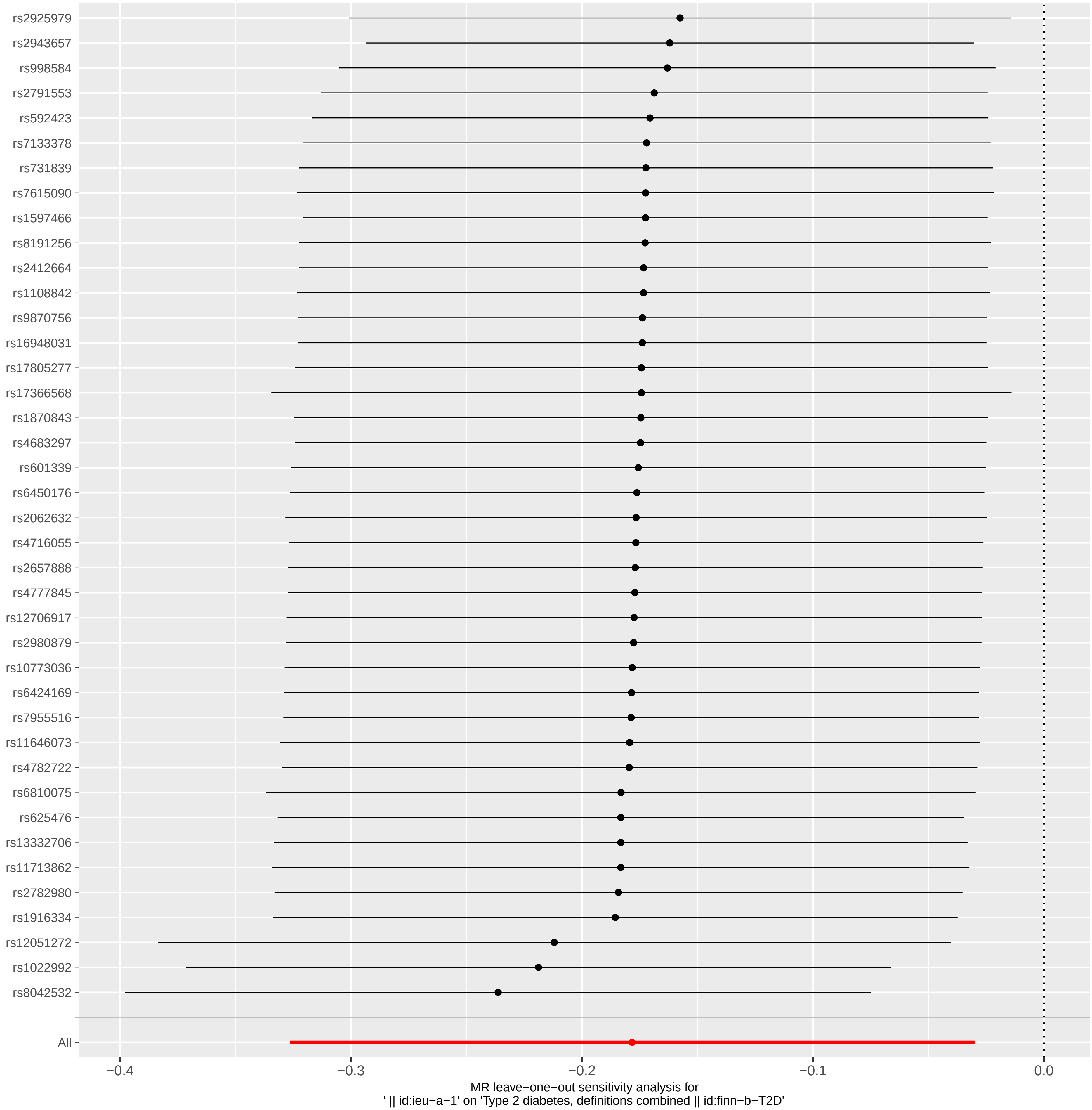
rs2925979
rs2943657
rs998584
rs2791553
rs592423
rs7133378
rs731839
rs7615090
rs1597466
rs8191256
rs2412664
rs1108842
rs9870756
rs16948031
rs17805277
rs17366568
rs1870843
rs4683297
rs601339
rs6450176
rs2062632
rs4716055
rs2657888
rs4777845
rs12706917
rs2980879
rs10773036
rs6424169
rs7955516
rs11646073
rs4782722
rs6810075
rs625476
rs13332706
rs11713862
rs2782980
rs1916334
rs12051272
rs1022992
rs8042532
All
−0.4
−0.3
−0.2
−0.1
0.0
MR leave−one−out sensitivity analysis for
' || id:ieu−a−1' on 'Type 2 diabetes, definitions combined || id:finn−b−T2D'

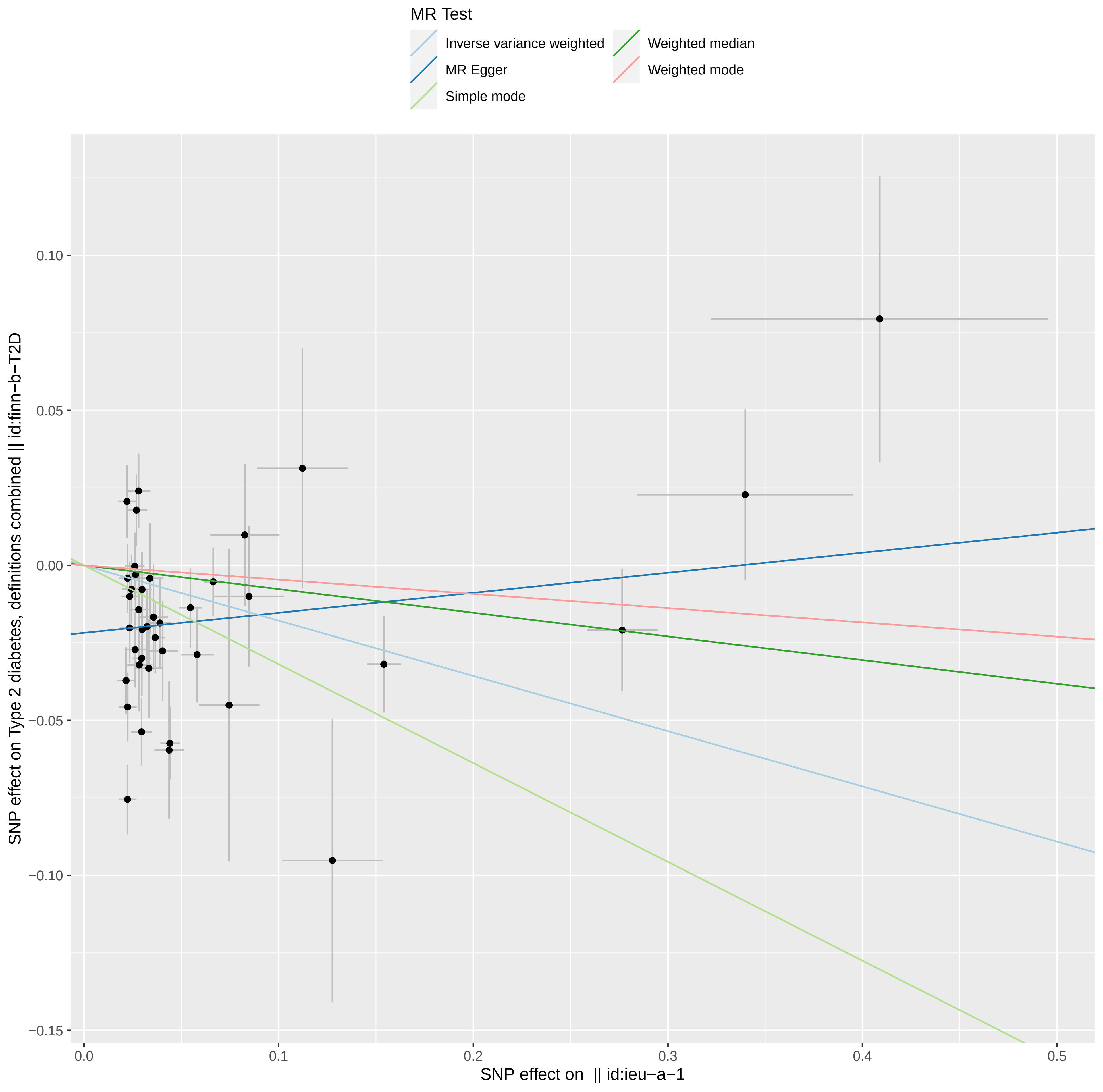

MR Test
Inverse variance weighted
MR Egger
Simple mode
Weighted median
Weighted mode
0.10
0.05
0.00
−0.05
−0.10
−0.15
0.0
0.1
0.2
0.3
0.4
0.5
SNP effect on Type 2 diabetes, definitions combined || id:finn−b−T2D
SNP effect on || id:ieu−a−1

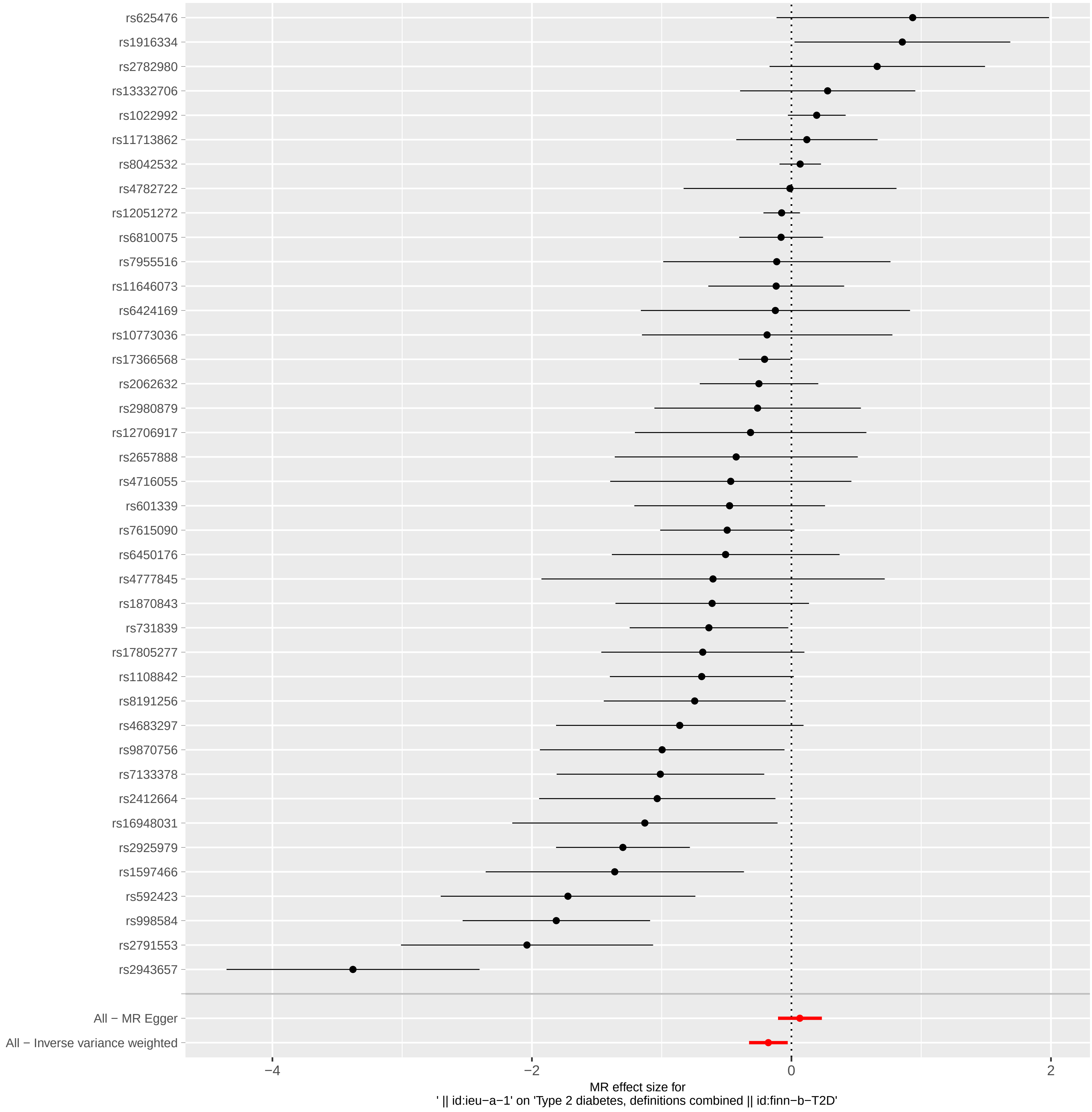
rs625476
rs1916334
rs2782980
rs13332706
rs1022992
rs11713862
rs8042532
rs4782722
rs12051272
rs6810075
rs7955516
rs11646073
rs6424169
rs10773036
rs17366568
rs2062632
rs2980879
rs12706917
rs2657888
rs4716055
rs601339
rs7615090
rs6450176
rs4777845
rs1870843
rs731839
rs17805277
rs1108842
rs8191256
rs4683297
rs9870756
rs7133378
rs2412664
rs16948031
rs2925979
rs1597466
rs592423
rs998584
rs2791553
rs2943657
All − MR Egger
All − Inverse variance weighted
−4
−2
0
2
MR effect size for
' || id:ieu−a−1' on 'Type 2 diabetes, definitions combined || id:finn−b−T2D'

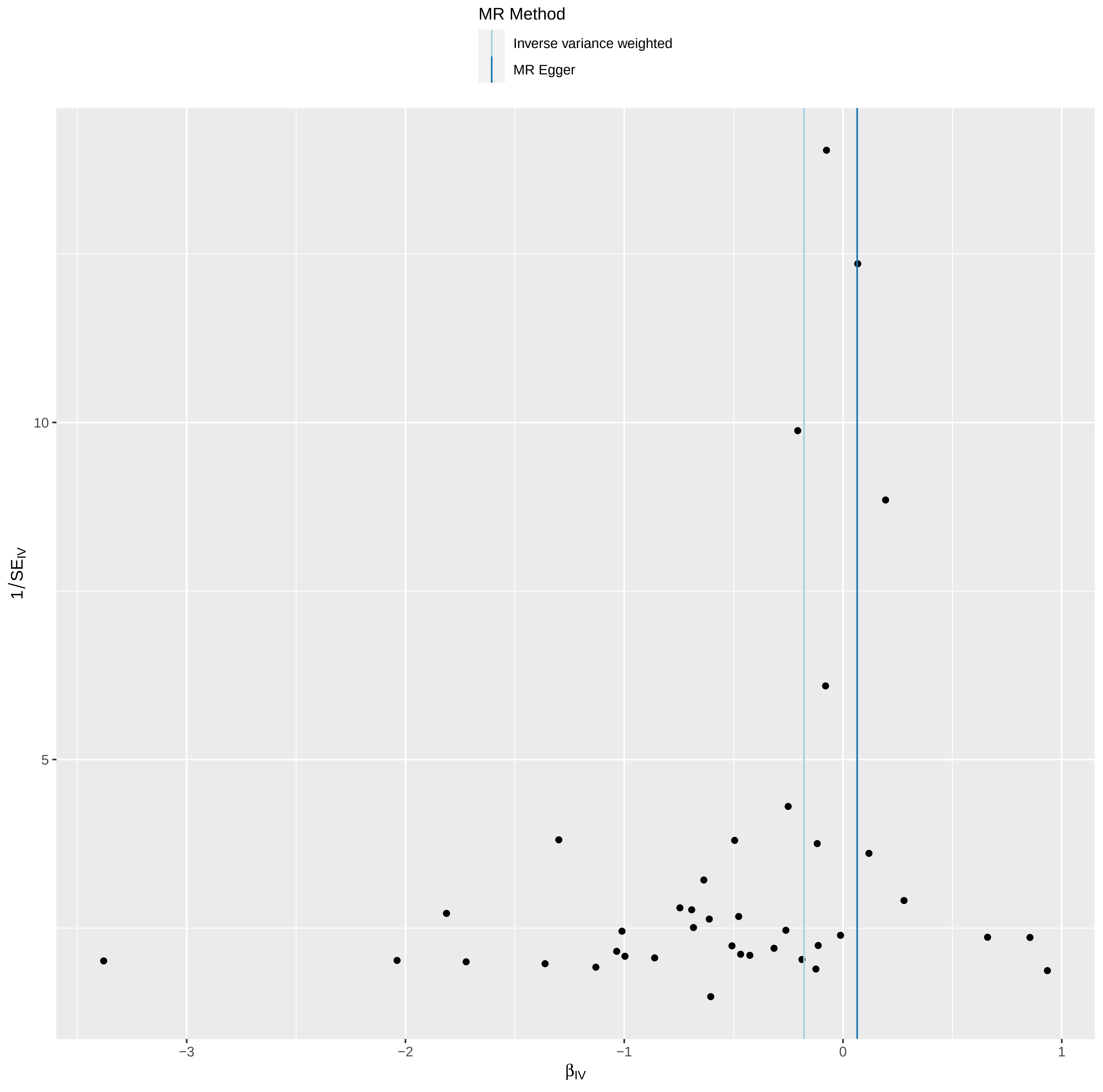

MR Method
Inverse variance weighted
MR Egger
10
5
1/SE_IV
−3
−2
−1
0
1
β_IV

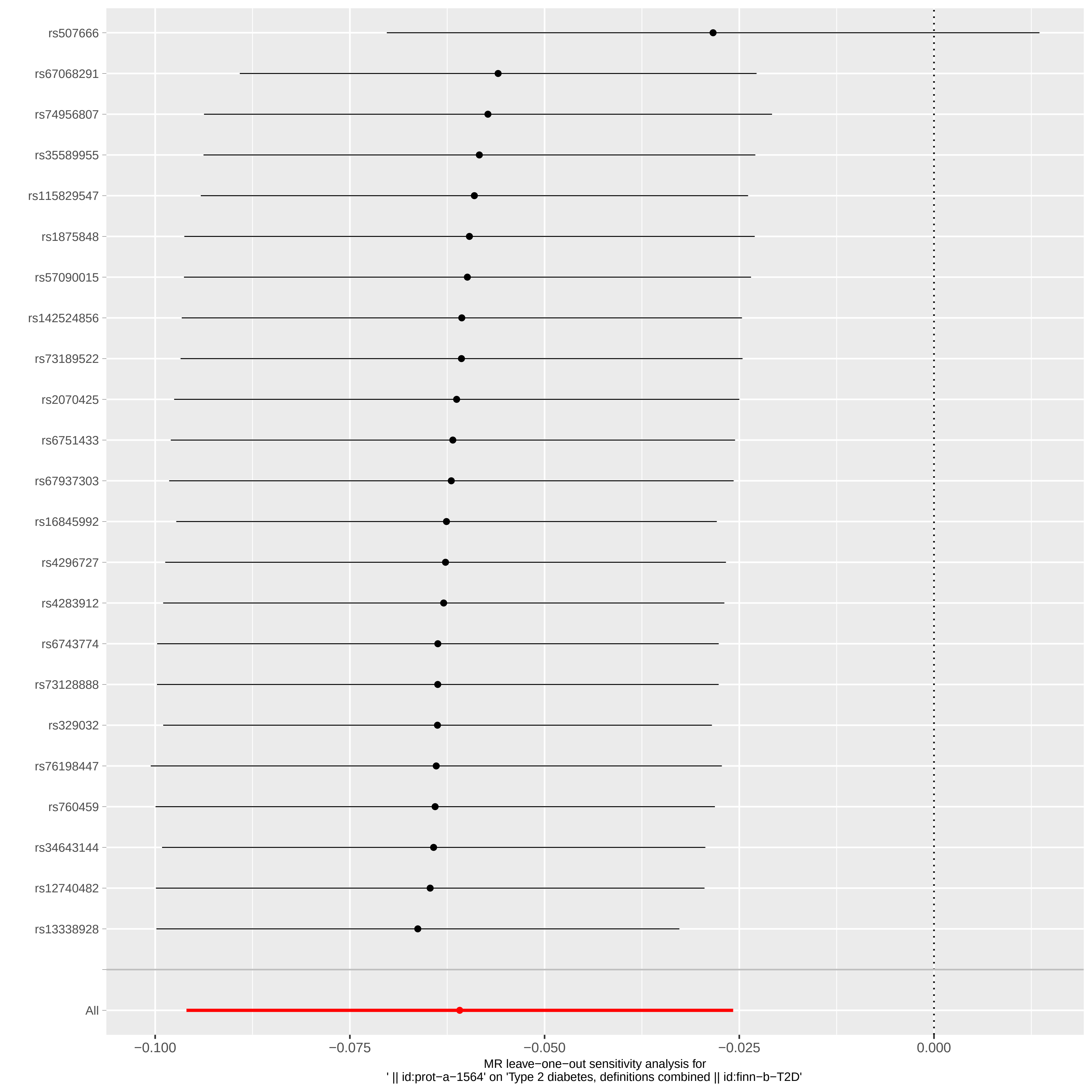
rs507666
rs67068291
rs74956807
rs35589955
rs115829547
rs1875848
rs57090015
rs142524856
rs73189522
rs2070425
rs6751433
rs67937303
rs16845992
rs4296727
rs4283912
rs6743774
rs73128888
rs329032
rs76198447
rs760459
rs34643144
rs12740482
rs13338928
All
−0.100
−0.075
−0.050
−0.025
0.000
MR leave−one−out sensitivity analysis for
' || id:prot−a−1564' on 'Type 2 diabetes, definitions combined || id:finn−b−T2D'

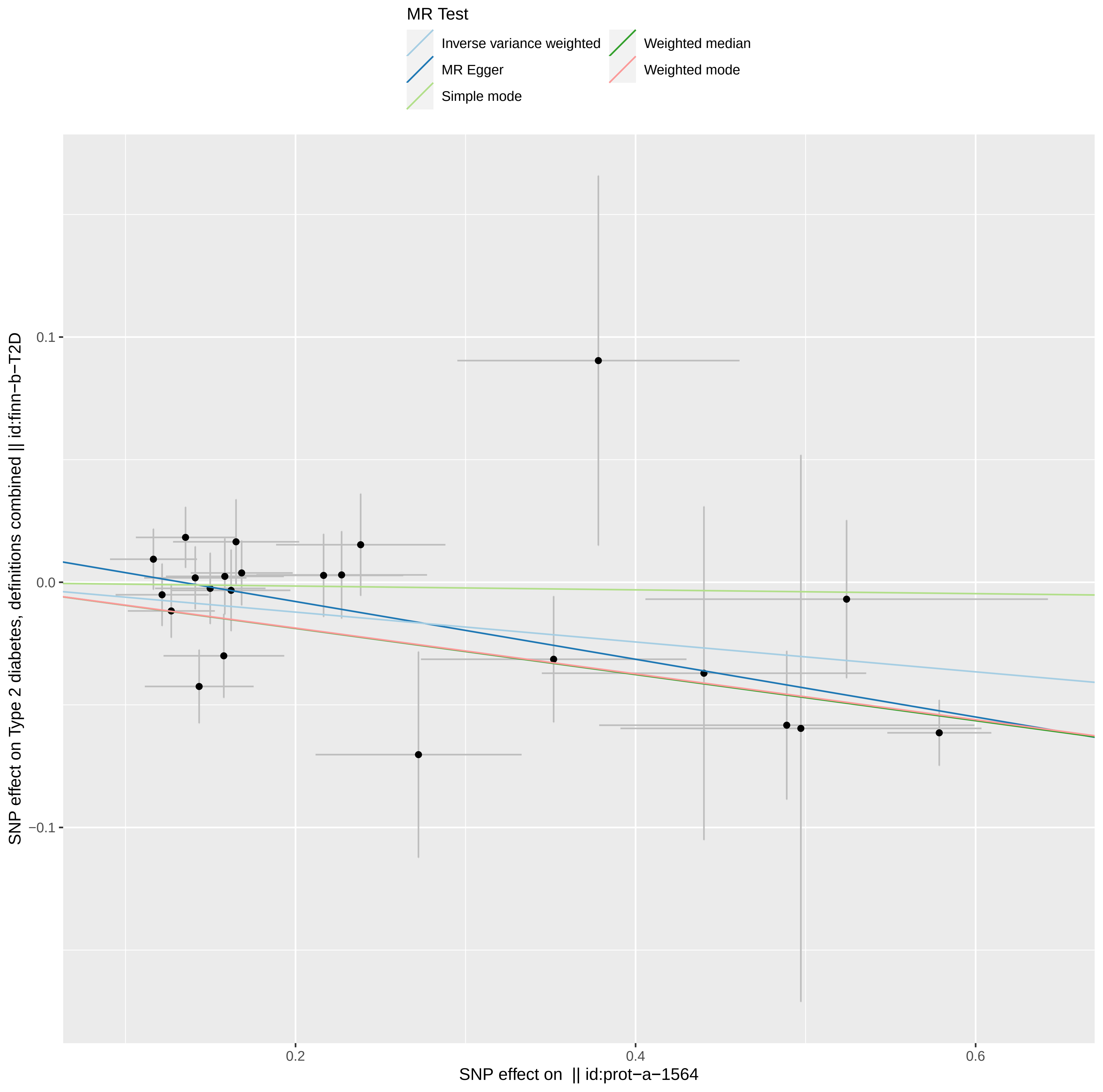
MR Test
Inverse variance weighted
MR Egger
Simple mode
Weighted median
Weighted mode
0.1
0.0
−0.1
0.2
0.4
0.6
SNP effect on Type 2 diabetes, definitions combined || id:finn−b−T2D
SNP effect on || id:prot−a−1564

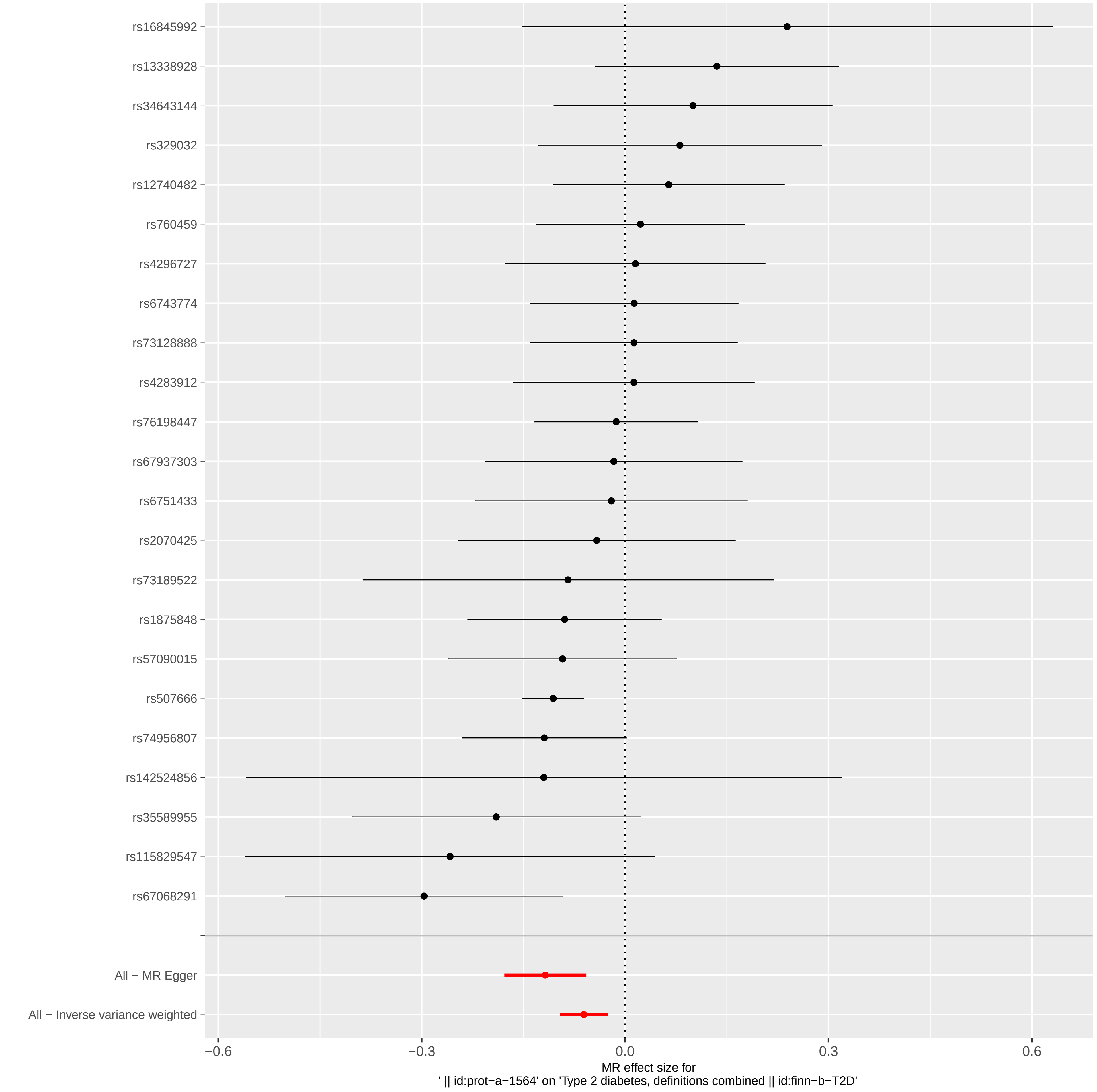
rs16845992
rs13338928
rs34643144
rs329032
rs12740482
rs760459
rs4296727
rs6743774
rs73128888
rs4283912
rs76198447
rs67937303
rs6751433
rs2070425
rs73189522
rs1875848
rs57090015
rs507666
rs74956807
rs142524856
rs35589955
rs115829547
rs67068291
All − MR Egger
All − Inverse variance weighted
−0.6
−0.3
0.0
0.3
0.6
MR effect size for
' || id:prot−a−1564' on 'Type 2 diabetes, definitions combined || id:finn−b−T2D'

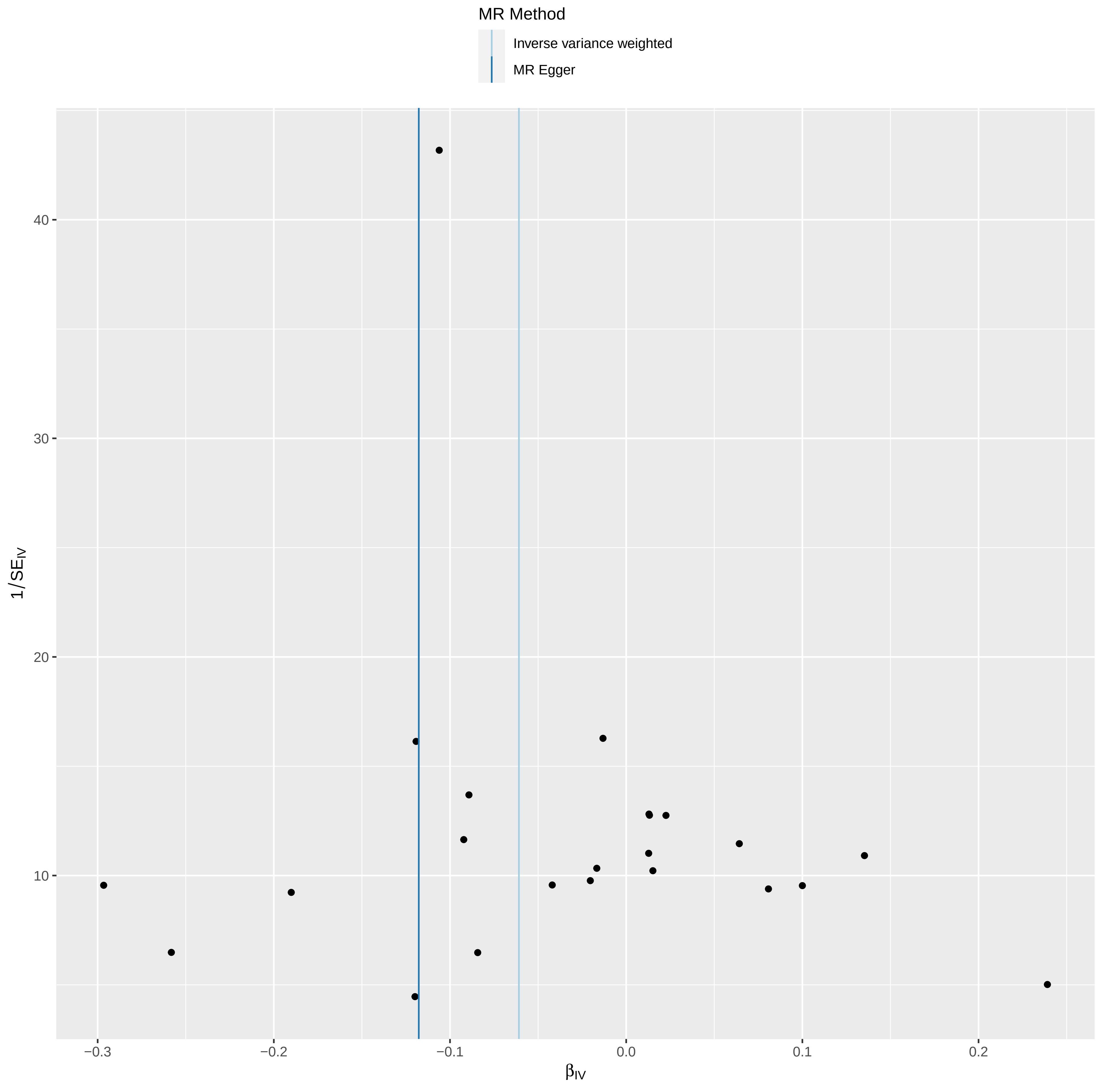

MR Method
Inverse variance weighted
MR Egger
40
30
20
10
1/SE_IV
−0.3
−0.2
−0.1
0.0
0.1
0.2
β_IV
